# Centrosome amplification fine tunes tubulin acetylation to differentially control intracellular organization

**DOI:** 10.15252/embj.2022112812

**Published:** 2023-07-05

**Authors:** Pedro Monteiro, Bongwhan Yeon, Samuel S Wallis, Susana A Godinho

**Affiliations:** ^1^ Centre for Cancer Cell and Molecular Biology, Barts Cancer Institute Queen Mary University of London London UK; ^2^ Institut Curie, Paris Sciences and Lettres Research University Centre National de la Recherche Scientifique, UMR144 Paris France

**Keywords:** ATAT1, centrosome amplification, microtubules, kinesin‐1, tubulin acetylation, Post-translational Modifications & Proteolysis

## Abstract

Intracellular organelle organization is conserved in eukaryotic cells and is primarily achieved through active transport by motor proteins along the microtubule cytoskeleton. Microtubule post‐translational modifications (PTMs) can contribute to microtubule diversity and differentially regulate motor‐mediated transport. Here, we show that centrosome amplification, commonly observed in cancer and shown to promote aneuploidy and invasion, induces a global change in organelle positioning towards the cell periphery and facilitates nuclear migration through confined spaces. This reorganization requires kinesin‐1 and is analogous to the loss of dynein. Cells with amplified centrosomes display increased levels of acetylated tubulin, a PTM that could enhance kinesin‐1‐mediated transport. Depletion of α‐tubulin acetyltransferase 1 (αTAT1) to block tubulin acetylation rescues the displacement of centrosomes, mitochondria, and vimentin but not Golgi or endosomes. Analyses of the distribution of total and acetylated microtubules indicate that the polarized distribution of modified microtubules, rather than levels alone, plays an important role in the positioning of specific organelles, such as the centrosome. We propose that increased tubulin acetylation differentially impacts kinesin‐1‐mediated organelle displacement to regulate intracellular organization.

## Graphical abstract

Kinesin‐1 and polarized distribution of acetylated microtubules converge to regulate organelle distribution and cell migration through confined spaces.

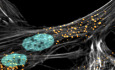

## Introduction

Eukaryotic cells display a conserved interconnected arrangement of the main cellular compartments and organelles. For metazoans, extensive changes in cell shape that occur during differentiation or migration are accompanied by organelle repositioning to maintain the functional relationship between organelles (Bornens, 2008). Thus, the ability of cells to continuously adapt and respond to physiological cues requires individual organelles to be relocated. Intracellular organelle organization is primarily achieved through active transport by motor proteins along cytoskeleton filaments (Barlan & Gelfand, 2017). The microtubule cytoskeleton, composed of αβ tubulin dimers, is intrinsically polarized, with the minus‐end of microtubules generally located at the center of the cell, and plus‐end located towards the cell periphery. This polarity and distinct distribution covering most of the cytoplasm makes the microtubule cytoskeleton ideally suited to orchestrate intracellular bidirectional transport of organelles. This is important for organelle distribution and is mediated by two classes of microtubule motor proteins; minus‐end directed dynein and plus‐end directed kinesins (Bryantseva & Zhapparova, 2012; Barlan & Gelfand, 2017). Dynein and kinesin motors generate opposing pulling and pushing forces on organelles to maintain their characteristic cellular distribution, often referred to as *tug‐of‐war* (Sweeney & Holzbaur, 2018). Changes in the direction of transport occur when one motor wins over the other, usually in response to cellular and environmental signals (Bryantseva & Zhapparova, 2012; Barlan & Gelfand, 2017; Monzon *et al*, 2020).

Different tubulin isotypes, association with various microtubule‐associated proteins, and tubulin post‐translational modifications (PTMs) contribute to the microtubule diversity and create different preferences for molecular motors (Janke & Magiera, 2020). Microtubules undergo numerous PTMs, including detyrosination, acetylation, phosphorylation, palmitoylation, polyglutamylation, and polyglycylation (Janke & Magiera, 2020). Diversity of tubulin isotypes and associated carboxy‐terminal tail PTMs have been shown to differentially regulate several molecular motors *in vitro* using chemically modified yeast tubulin (Sirajuddin *et al*, 2014). Moreover, in cells, detyrosination and acetylation can affect the binding and motility of kinesin‐1 motors (Liao & Gundersen, 1998; Reed *et al*, 2006; Balabanian *et al*, 2017; Ravindran *et al*, 2017; Tas *et al*, 2017). Thus, microtubule PTMs could play a role in organelle distribution and overall intracellular organization. Consistent with this idea, endoplasmic reticulum (ER) distribution is mediated by both tubulin acetylation and glutamylation, which regulates ER‐mitochondria interactions and cytoplasm distribution, respectively (Friedman *et al*, 2010; Zheng *et al*, 2022). However, it remains unclear how these PTMs regulate the net distribution of multiple organelles and, in particular, how different organelles respond to the same modifications.

The centrosome, which is the main microtubule organizing center in somatic cells, occupies a very characteristic position at the cell center and in close contact with the nucleus (Bornens, 1977, 2008). This close contact is, in part, regulated by the interaction between centrosomal microtubules and the Linker of Nucleoskeleton and Cytoskeleton (LINC) complex, composed of nesprins and SUN proteins, at the nuclear envelope (Gundersen & Worman, 2013). In addition, centrosome positioning at the cell's centroid is actively maintained by the radial distribution of microtubules and dynein pulling forces and also responds to anisotropic distribution of the actin network, particularly in enucleated cells (cytoplasts; Koonce *et al*, 1999; Burakov *et al*, 2003; Jimenez *et al*, 2021). Numerical centrosome abnormalities, such as centrosome amplification, can be found in cancer cells and play direct roles in tumorigenesis (Nigg & Holland, 2018; Goundiam & Basto, 2021). Centrosome amplification can directly promote cell invasion, partially due to increased microtubule nucleation (Godinho *et al*, 2014). Thus, it is possible that some of the oncogenic potentials of centrosome abnormalities could be due to microtubule alterations. However, how centrosome amplification impacts the microtubule cytoskeleton remains largely unknown.

In this study we discovered that inducing centrosome amplification leads to a global change in the distribution of intracellular compartments towards the cell periphery, in a process that requires kinesin‐1, suggesting it results from an imbalance of forces that favors plus‐end directed motors. Cells with amplified centrosomes display increased tubulin acetylation, a microtubule PTM previously shown to facilitate kinesin‐1‐mediated motility (Reed *et al*, 2006; Ravindran *et al*, 2017; Tas *et al*, 2017). Systematic analyses of several intracellular compartments revealed that changes in acetylated tubulin levels differentially impact intracellular organization in cells, in particular centrosomes, mitochondria, and vimentin. Surprisingly, we found that not only the increase in acetylated tubulin, but also the polarized distribution of acetylated microtubules plays an important role in the distribution of specific organelles, such as the centrosome. This supports a model whereby force distribution of microtubule motors, due to changes in PTMs, could differentially influence the position of individual organelles. In addition, we observed that cells with amplified centrosomes have increased nuclear deformability and migrate more proficiently through small pores, which also requires tubulin acetylation. Taken together, these findings demonstrate that tubulin acetylation differentially regulates the positioning of individual intracellular compartments and that intracellular reorganization could facilitate invasion in cells with amplified centrosomes.

## Results

### Centrosome amplification leads to kinesin‐1‐mediated centrosome displacement

**Figure 1:**
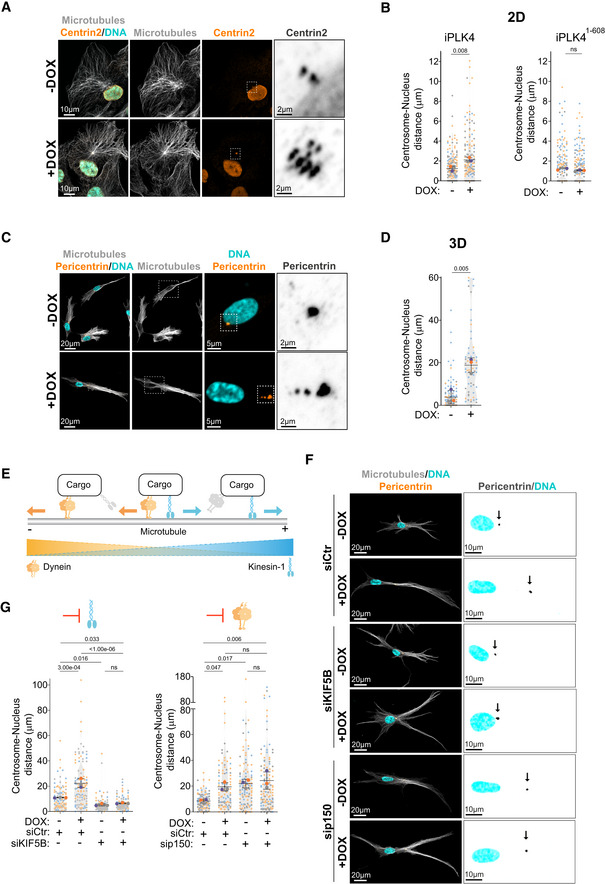
Increased centrosome displacement downstream of centrosome amplification requires kinesin‐1 Representative images of cells stained for centrosomes (Centrin2, orange), microtubules (α‐tubulin, gray), and DNA (Hoechst, cyan). Scale bar: 10 μm; inset scale bar: 2 μm.Quantification of centrosome‐nucleus distance in cells upon induction of PLK4 (Left panel; *n*_(−DOX)_ = 174; *n*_(+DOX)_ = 162) or PLK4^1‐608^ overexpression (Right panel; *n*_(−DOX)_ = 192; *n*_(+DOX)_ = 203).Representative images of cells embedded in a 3D collagen matrix and stained for centrosomes (Pericentrin, orange), microtubules (α‐tubulin, gray), and DNA (Hoechst, cyan). Scale bar: 20 μm; inset DNA/Pericentrin scale bar: 5 μm; inset Pericentrin scale bar: 2 μm.Quantification of centrosome‐nucleus distance (*n*_(−DOX)_ = 100; *n*_(+DOX)_ = 62).Scheme recapitulating the balance forces mediated by kinesin‐1 (blue) and dynein (orange) along microtubules.Representative images of cells embedded in a 3D collagen matrix and stained for centrosomes (Pericentrin, orange), microtubules (α‐tubulin, gray), and DNA (Hoechst, cyan) treated with siRNA control (Ctr), siRNA KIF5B or siRNA p150. Scale bar: 20 μm. Black arrows indicate the position of the centrosome(s). Inset scale bar: 10 μm.Left panel, Quantification of centrosome‐nucleus distance upon KIF5B depletion (number of cells: *n*_(−DOX siCtr)_ = 96; *n*_(+DOX siCtr)_ = 108; *n*_(−DOX siKIF5B)_ = 111; *n*_(+DOX siKIF5B)_ = 91); Right panel, Quantification of centrosome‐nucleus distance upon p150 depletion (*n*_(−DOX siCtr)_ = 102; *n*_(+DOX siCtr)_ = 96; *n*_(−DOX sip150)_ = 115; *n*_(+DOX sip150)_ = 117). Data information: For all graphs, error bars represent mean ± SD from three independent experiments. *P*‐values are described in the graphs, ns = not significant (*P* > 0.05). The following statistics were applied: unpaired *t*‐test for graphs in (B) and (D) and one‐way ANOVA with Tukey's *post hoc* test for graphs in (G). *n* = number of cells analyzed. Source data are available online for this figure.

**Figure EV1:**
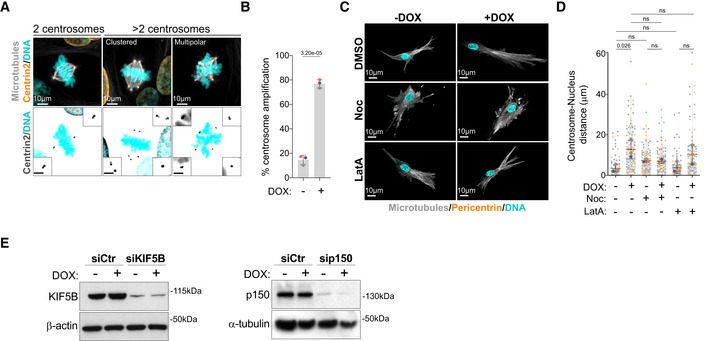
Increased centrosome displacement in cells with extra centrosomes requires microtubules Representative images of cells stained for centrosomes (Centrin2, orange), microtubules (α‐tubulin, gray), and DNA (Hoechst, cyan). Scale bar: 10 μm; inset scale bar: 2 μm.Quantification of metaphase cells with extra centrosomes (*n*_(−DOX)_ = 337; *n*_(+DOX)_ = 339).Representative images of cells embedded in a 3D collagen matrix and stained for centrosomes (Pericentrin, orange), microtubules (α‐tubulin, gray), and DNA (Hoechst, cyan) treated with nocodazole (Noc, 10 μM) or latrunculin‐A (LatA, 100 nM). Scale bar: 10 μm.Quantification of centrosome‐nucleus distance (*n*_(−DOX)_ = 90; *n*_(+DOX)_ = 114; *n*_(−DOX Noc)_ = 112; *n*_(+DOX Noc)_ = 101; *n*_(−DOX LatA)_ = 110; *n*_(+DOX LatA)_ = 108).Left panel; immunoblot of KIF5B and β‐actin in cells after KIF5B siRNA for 48 h. Right panel; immunoblot of p150 and α‐tubulin in cells after p150 siRNA for 48 h. Data information: For all graphs, error bars represent mean ± SD from three independent experiments. *P*‐values are described in the graphs, ns = not significant (*P* > 0.05). The following statistics were applied: unpaired *t*‐test for graph in (B) and one‐way ANOVA with Tukey's *post hoc* test for graph in (D). *n* = number of cells analyzed. Source data are available online for this figure.

In interphase cells, centrosomes localize in close proximity to the nucleus, with an average 1‐ to 2‐μm distance in most cells (Rezaul *et al*, 2016). Unexpectedly, we found that induction of centrosome amplification, by transiently overexpressing Polo‐like kinase 4 (PLK4) using doxycycline (DOX)‐inducible system (Arnandis *et al*, 2018) (Fig EV1A and B), led to the displacement of clustered centrosomes away from the nucleus and towards the cell periphery (~1.7 fold) in RPE‐1 cells (RPE‐1.iPLK4) (Fig 1A and B). This phenotype is not due to unspecific effects of DOX treatment or PLK4 overexpression since DOX‐induced overexpression of a PLK4 truncated mutant, PLK4^1‐608^, which is catalytically active but does not localize to the centrosomes and cannot induce centrosome amplification (Guderian *et al*, 2010), did not lead to centrosome displacement (Fig 1B). To test whether increasing cell polarization further exacerbated this phenotype, RPE‐1.iPLK4 cells were embedded in a 3D collagen‐I matrix to promote cell elongation and polarization. Indeed, the distance between extra centrosomes and the nucleus was further enhanced in cells plated in 3D (~4.1 fold) (Fig 1C and D). These results indicate that increasing centrosome numbers is sufficient to displace centrosomes away from the nucleus.

Microtubule depolymerization by nocodazole led to a small increase in centrosome‐nucleus distance in cells with normal centrosome number, which is consistent with loss of centrosome‐nucleus attachment (Salpingidou *et al*, 2007). However, no further increase was observed in cells with amplified centrosomes, suggesting that microtubules play a key role in this process (Fig EV1C and D). By contrast, F‐actin depolymerization by latrunculin‐A did not prevent centrosome displacement in cells with amplified centrosomes (Fig EV1C and D). Organelle positioning is often dictated by a balance of forces mediated by minus‐end directed dynein and plus‐end directed kinesin‐1 motors (Hancock, 2014; Belyy *et al*, 2016) (Fig 1E). Therefore, we asked whether the displacement of centrosomes towards the cell periphery was due to imbalanced forces that favored kinesin‐1. To test this, we depleted the ubiquitously expressed kinesin‐1 Kinesin Family Member 5B (KIF5B) by siRNA in cells plated in 3D collagen‐I matrices. We found that upon KIF5B depletion, supernumerary centrosomes remained closely associated with the nucleus, suggesting that pushing forces on the centrosomes are mediated by kinesin‐1 (Figs 1F and G, and EV1E). Consistent with dynein's role in counteracting kinesin‐1 pushing forces to maintain centrosome positioning (Splinter *et al*, 2010; Stiff *et al*, 2020), inhibition of dynein by depleting the p150^glued^ subunit of the dynactin complex led to centrosome displacement in control cells but had no impact on centrosome displacement in cells with amplified centrosomes (Figs 1F and G, and EV1E). Taken together, these results demonstrate that unbalanced forces that favor kinesin‐1 mediate centrosome displacement in cells with amplified centrosomes.

### Centrosome amplification leads to global intracellular reorganization

**Figure 2:**
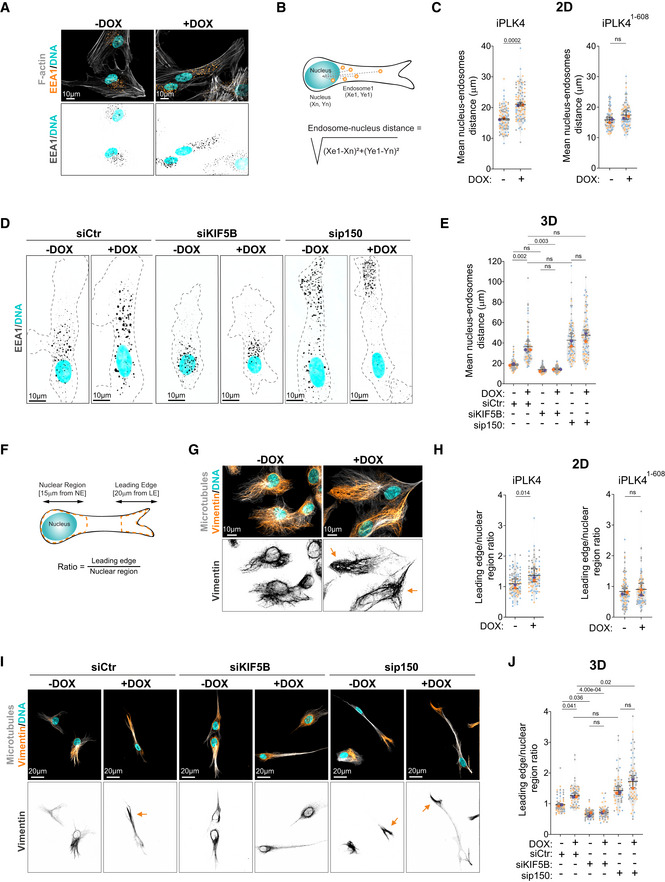
Kinesin‐1 mediates the displacement of endosomes and intermediate filaments in cells with amplified centrosomes Representative images of cells stained for early endosomes (EEA1, orange), F‐actin (phalloidin, gray), and DNA (Hoechst, cyan). Scale bar: 10 μm.Representing scheme of nucleus‐endosomes distance quantification.Quantification of nucleus‐endosomes distance upon induction of PLK4 (Left panel: *n*_(−DOX)_ = 103; *n*_(+DOX)_ = 100) or PLK4^1‐608^ overexpression (Right panel: *n*_(−DOX)_ = 79; *n*_(+DOX)_ = 83).Representative images of cells embedded in a 3D collagen matrix and stained for early endosomes (EEA1, gray) and DNA (Hoechst, cyan). Dark dotted line represents cell contour. Scale bar: 10 μm.Quantification of nucleus‐endosomes distance upon depletion of KIF5B and p150 (*n*_(−DOX siCtr)_ = 84; *n*_(+DOX siCtr)_ = 81; *n*_(−DOX siKIF5B)_ = 84; *n*_(+DOX siKIF5B)_ = 83; *n*_(−DOX sip150)_ = 82; *n*_(+DOX sip150)_ = 83).Representative scheme of vimentin displacement quantification.Representative images of cells stained for vimentin (orange), microtubules (α‐tubulin, gray), and DNA (Hoechst, cyan). Orange arrows indicate the displacement of vimentin towards cell periphery. Scale bar: 10 μm.Quantification of vimentin leading edge/nuclear ratio upon induction of PLK4 (*n*_(−DOX)_ = 102; *n*_(+DOX)_ = 79) or PLK4^1‐608^ overexpression (Right panel; *n*_(−DOX)_ = 104; *n*_(+DOX)_ = 101).Representative images of cells embedded in a 3D collagen matrix and stained for vimentin (orange), microtubules (α‐tubulin, gray), and DNA (Hoechst, cyan) upon depletion of KIF5B and p150. Orange arrows indicate the displacement of vimentin towards cell periphery. Scale bar: 20 μm.Quantification of vimentin leading edge/nuclear ratio (*n*_(−DOX siCtr)_ = 68; *n*_(+DOX siCtr)_ = 63; *n*_(−DOX siKIF5B)_ = 70; *n*_(+DOX siKIF5B)_ = 64; *n*_(−DOX sip150)_ = 58; *n*_(+DOX sip150)_ = 63). Data information: For all graphs, error bars represent mean ± SD from three independent experiments. *P*‐values are described in the graphs, ns = not significant (*P* > 0.05). The following statistics were applied: unpaired *t*‐test for graphs in (C) and (H) and one‐way ANOVA with Tukey's *post hoc* test for graphs in (E) and (J). *n* = number of cells analyzed. Source data are available online for this figure.

**Figure EV2:**
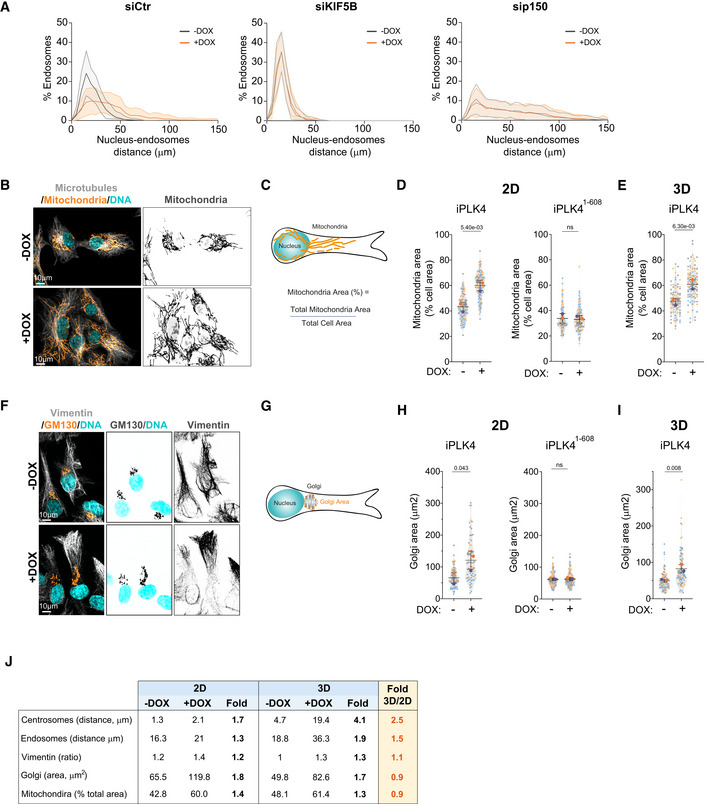
Centrosome amplification promotes mitochondria displacement and Golgi dispersion Distribution of endosomes in cells upon depletion of KIF5B and p150 (*n*_(−DOX siCtr)_ = 84; *n*_(+DOX siCtr)_ = 81; *n*_(−DOX siKIF5B)_ = 84; *n*_(+DOX siKIF5B)_ = 83; *n*_(−DOX sip150)_ = 82; *n*_(+DOX sip150)_ = 83).Representative images of cells stained for mitochondria (MitoTracker, orange), microtubules (α‐tubulin, gray), and DNA (Hoechst, cyan). Scale bar: 10 μm.Representative scheme of mitochondria area quantification.Quantification of mitochondria area in cells plated in 2D upon induction of PLK4 (Left panel; *n*_(−DOX)_ = 113; *n*_(+DOX)_ = 114) or PLK4^1‐608^ overexpression (Right panel; *n*_(−DOX)_ = 95; *n*_(+DOX)_ = 98).Quantification of mitochondria area in cells plated in 3D (*n*_(−DOX)_ = 90; *n*_(+DOX)_ = 102).Representative images of cells stained for Golgi (GM130, orange), vimentin (gray) and DNA (Hoechst, cyan). Scale bar: 10 μm.Representative scheme of Golgi area quantification.Quantification of Golgi area upon induction of PLK4 (Left panel; *n*_(−DOX)_ = 94; *n*_(+DOX)_ = 70) or PLK4^1‐608^ overexpression (Right panel; *n*_(−DOX)_ = 146; *n*_(+DOX)_ = 133).Quantification of Golgi area in cells plated in 3D (*n*_(−DOX)_ = 91; *n*_(+DOX)_ = 83).Table summarizing the fold change between 2D and 3D conditions for the intracellular compartments analyzed. Data information: For all graphs, error bars represent mean ± SD from three independent experiments. *P*‐values are described in the graphs, ns = not significant (*P* > 0.05). The following statistics were applied: unpaired *t*‐test for all graphs. *n* = number of cells analyzed. Source data are available online for this figure.

Since other organelles and cellular components rely on dynein‐kinesin balance for their positioning (Barlan & Gelfand, 2017), we next investigated whether centrosome amplification played a global role in organelle positioning in cells plated in 2D and 3D collagen‐I matrices. Using the early endosomal antigen 1 (EEA1) marker, we assessed the distribution of early endosomes in cells with normal (−DOX) and amplified (+DOX) centrosomes. Indeed, similar to centrosomes, the distance between the nucleus and endosomes increased in cells with amplified centrosomes, but not in cells overexpressing PLK4^1‐608^ (Fig 2A–C), and also mirrored the levels of endosome dispersion in p150^glued^‐depleted control cells (Marchesin *et al*, 2015; Figs 2D and E, and EV2A). Depletion of KIF5B in control cells and cells with amplified centrosomes resulted in the repositioning of endosomes near the nucleus (Figs 2D and E, and EV2A). We also examined the intracellular distribution of the intermediate filament component vimentin, since it relies on kinesin‐1 to be transported towards the leading edge (Gyoeva & Gelfand, 1991; Liao & Gundersen, 1998; Leduc & Etienne‐Manneville, 2017). To do so, we quantified vimentin's distribution ratio (leading edge/nucleus), where a ratio > 1 indicates a dispersal towards the leading edge (Fig 2F) (Leduc & Etienne‐Manneville, 2017). While in control cells (−DOX) and in cells overexpressing PLK4^1‐608^ vimentin remains mostly around the nucleus, in cells with amplified centrosomes (+DOX), vimentin is further displaced towards the cell periphery (Fig 2G and H). Dispersion of vimentin in cells with amplified centrosomes also required KIF5B, and mirrored what is observed in control cells upon depletion of p150^glued^ (Fig 2I and J). Additionally, we observed that both the mitochondria (labeled with MitoTracker) and the Golgi (using GM130 as marker) were also displaced in cells with amplified centrosomes in 2D and 3D cultures, but not in cells overexpressing PLK4^1‐608^ (Fig EV2B–I). Interestingly, from all intracellular compartments we analyzed, centrosome displacement is the most sensitive to increased cell elongation/polarization in cells plated in 3D (Fig EV2J). Taken together, these data indicate an unprecedented role for centrosome amplification in organelle organization, in a process that is dependent on the kinesin‐1 KIF5B.

### Cells with extra centrosomes exhibit increased tubulin acetylation levels

**Figure 3:**
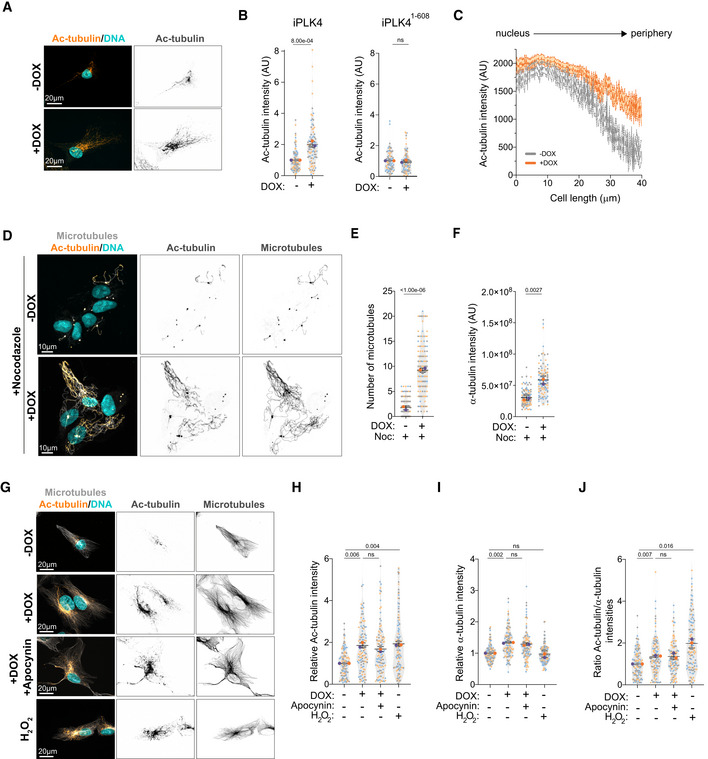
Tubulin acetylation is increased in cells with amplified centrosomes Representative images of cells stained for acetylated tubulin (Ac‐tubulin, orange) and DNA (Hoechst, cyan). Scale bar: 20 μm.Quantification of acetylated tubulin fluorescence intensity upon induction of PLK4 (Left panel: *n*_(−DOX)_ = 98; *n*_(+DOX)_ = 89) or PLK4^1‐608^ overexpression (Right panel: *n*_(−DOX)_ = 78; *n*_(+DOX)_ = 85).Distribution of acetylated tubulin fluorescence intensity throughout the cell length (nucleus to periphery) (*n*_(−DOX)_ = 37; *n*_(+DOX)_ = 42).Representative images of cells stained for microtubules (α‐tubulin, gray), tubulin acetylation (Ac‐tubulin, orange) and DNA (Hoechst, cyan) upon nocodazole treatment (Noc, 2 μM). Scale bar: 10 μm.Quantification of the number of microtubules (*n*_(−DOX Noc)_ = 185; *n*_(+DOX Noc)_ = 149).Quantification total α‐tubulin fluorescence intensity (*n*_(−DOX Noc)_ = 128; *n*_(+DOX Noc)_ = 105).Representative images of cells stained for microtubules (α‐tubulin, gray), acetylated tubulin (Ac‐tubulin, orange) and DNA (Hoechst, cyan) treated with Apocynin (0.5 mM) or H_2_O_2_ (75 μM). Scale bar: 20 μm.Quantification of acetylated tubulin fluorescence intensity (*n*_(−DOX)_ = 118; *n*_(+DOX)_ = 111; *n*_(+DOX Apocynin)_ = 110; *n*_(−DOX H2O2)_ = 118).Quantification of total α‐tubulin fluorescence intensity (*n*_(−DOX)_ = 118; *n*_(+DOX)_ = 111; *n*_(+DOX Apocynin)_ = 110; *n*_(−DOX H2O2)_ = 118).Ratio of acetylated tubulin intensity relative to total α‐tubulin intensity (*n*_(−DOX)_ = 118; *n*_(+DOX)_ = 111; *n*_(+DOX Apocynin)_ = 110; *n*_(−DOX H2O2)_ = 118). Data information: For all graphs, error bars represent mean ± SD from three independent experiments. *P*‐values are described in the graphs, ns = not significant (*P* > 0.05). The following statistics were applied: unpaired *t*‐test for graphs in (B, E and F). For graphs in (H, I, and J), a one sample *t*‐test was used for comparisons with normalized −DOX condition (using a hypothetical mean of 1) and an unpaired *t*‐test to compare +DOX and +DOX + Apocynin conditions. *n* = number of cells analyzed. Source data are available online for this figure.

**Figure EV3:**
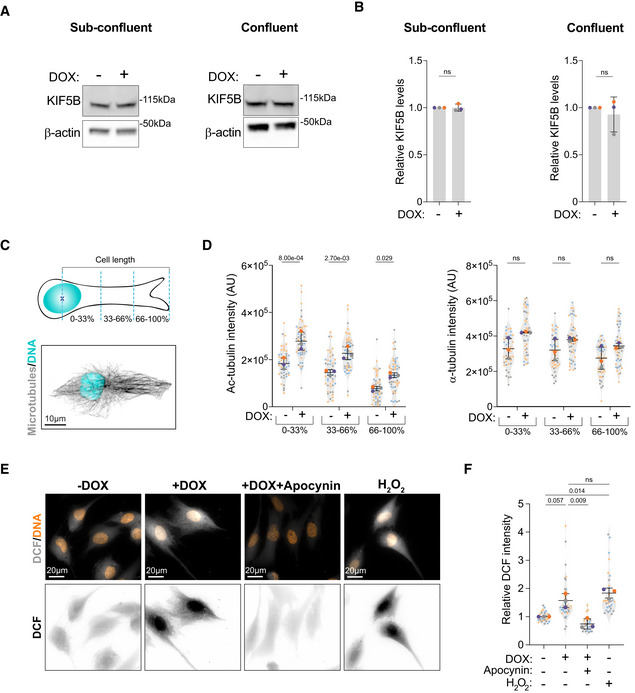
Distribution of acetylated microtubules and ROS levels in cells with amplified centrosomes Immunoblots of KIF5B and β‐actin in cells without (−DOX) and with amplified centrosomes (+DOX) under sub‐confluent (Left panel) and confluent (Right panel) conditions.Quantification of KIF5B total levels in cell lysates under sub‐confluent (Left panel) and confluent (Right panel) conditions.Top panel: Representative scheme of the quantification of intracellular distribution of acetylated tubulin. Bottom panel: Representative image of a control cell stained for microtubules (α‐tubulin, gray) and DNA (Hoechst, cyan). Scale bar: 10 μm.Left panel: Quantification of intracellular distribution of acetylated tubulin across the length of the cell; Right panel: Quantification of intracellular distribution of total tubulin across the length of the cell (*n*_(−DOX)_ = 47; *n*_(+DOX)_ = 52).Representative images of cells stained for DNA (Hoechst, orange) and DCF (gray) treated with Apocynin (0.5 mM) and H_2_O_2_ (75 μM). Scale bar: 20 μm.Quantification of total DCF fluorescence intensity (*n*_(−DOX)_ = 30; *n*_(+DOX)_ = 36; *n*_(+DOX Apocynin)_ = 27; *n*_(−DOX H2O2)_ = 30). Data information: For all graphs, error bars represent mean ± SD from three independent experiments. *P*‐values are described in the graphs, ns = not significant (*P* > 0.05). The following statistics were applied: unpaired *t*‐test for graphs in (B), one sample *t*‐test was used for comparisons with normalized −DOX condition (using a hypothetical mean of 1) and unpaired *t*‐test to compare +DOX and +DOX + Apocynin conditions for graph in (F) and two‐way ANOVA with Sidak's multiple test comparison for graph in (D). *n* = number of cells analyzed. Source data are available online for this figure.

Our data demonstrate that the extent of centrosome displacement between cells with amplified centrosomes and control cells depleted of dynein is very similar. This suggests that either a loss of dynein activity or increased kinesin‐1 activity in cells with extra centrosomes could be responsible for this phenotype. We reason that decreased dynein activity is unlikely to be the cause, as both control cells and cells with amplified centrosomes display a significant decrease in centrosome‐nucleus distance after KIF5B depletion (Fig 1G), which is consistent with functional dynein activity. We postulated that differences in the expression levels of KIF5B could explain these phenotypes. However, we did not observe any alteration in the total levels of KIF5B between control and cells with extra centrosomes (Fig EV3A and B). Therefore, we asked whether microtubule PTMs could be responsible for the enhanced kinesin‐1‐mediated transport. Tubulin acetylation has been shown to positively influence kinesin‐1 transport in cells, although to date the evidence suggesting that acetylated microtubules promote kinesin‐1 transport is still scarce (Reed *et al*, 2006; Ravindran *et al*, 2017; Tas *et al*, 2017). Thus, we decided to test whether displacement of the intracellular compartments observed in cells with amplified centrosomes was driven by changes in tubulin acetylation. Firstly, we measured the levels of tubulin acetylation by immunofluorescence in single cells and found that cells with extra centrosomes have a ~2‐fold increase in tubulin acetylation (Fig 3A and B). By contrast, overexpression of PLK4^1‐608^ had no impact on the levels of tubulin acetylation (Fig 3B). When compared to control cells, cells with amplified centrosomes showed a marked increase in tubulin acetylation levels throughout the cell and near the leading edge (Figs 3C and EV3C and D). These differences cannot be explained by changes in the levels of total α‐tubulin as these remain higher throughout the cell (Fig EV3D). Tubulin acetylation has been previously associated with long‐lived, nocodazole‐resistant microtubules and proposed to protect microtubules against mechanical aging (Portran *et al*, 2017; Xu *et al*, 2017). We found that cells with extra centrosomes retain a significantly increased population of nocodazole‐resistant microtubules that are acetylated, suggesting that increase tubulin acetylation could be a consequence of microtubule stabilization in these cells (Fig 3D–F).

To understand the source of increased tubulin acetylation in cells with extra centrosomes, we first assessed the role of reactive oxygen species (ROS) in this process. We have previously shown that cells with amplified centrosomes have increased levels of intracellular ROS (Arnandis *et al*, 2018; Adams *et al*, 2021) and it has been recently demonstrated that hydrogen peroxide (H_2_O_2_) can damage the microtubule lattice, resulting in increased tubulin acetylation (Goldblum *et al*, 2021). We confirmed that RPE‐1.iPLK4 cells with extra centrosomes (+DOX) displayed higher ROS levels, as measured by the levels of Dichlorodihydrofluorescein (DCF) that results from ROS‐mediated oxidation of hydrolyzed H_2_DCFDA. Increased intracellular ROS can be blocked by treating cells with the broad NADPH oxidase inhibitor Apocynin (Fig EV3E and F). While we observed that low doses of H_2_O_2_ can induce a similar increase in acetylated tubulin levels to cells with extra centrosomes, blocking ROS production with Apocynin in cells with amplified centrosomes did not prevent increased tubulin acetylation (Fig 3G and H). Because centrosome amplification can enhance microtubule nucleation (Godinho *et al*, 2014), we next tested whether increased total tubulin could account for the higher levels of acetylated tubulin in these cells. Quantification of α‐tubulin immunofluorescence intensity demonstrated that the presence of extra centrosomes leads to increased total tubulin in steady‐state cells, although this was not observed in H_2_O_2_‐treated cells (Fig 3I). Normalizing tubulin acetylation to total tubulin almost completely equalized the ratio of acetylated tubulin in cells with and without amplified centrosomes, although small significant differences can still be observed (Fig 3J). These results suggest that increased acetylated microtubules could result from higher levels of total tubulin or increased tubulin nucleation in cells with amplified centrosomes (Godinho *et al*, 2014). *In vitro*, αTAT1 can access the microtubule lattice through its ends where it acetylates α‐tubulin (Coombes *et al*, 2016). It is plausible that increased microtubule nucleation at the centrosomes, could improve access to microtubules and provide an explanation for the accumulation of acetylated tubulin around the centrosomes. However, more work needs to be done to fully understand how tubulin acetylation is regulated in these cells.

### Acetylated tubulin differentially regulates intracellular reorganization

**Figure 4:**
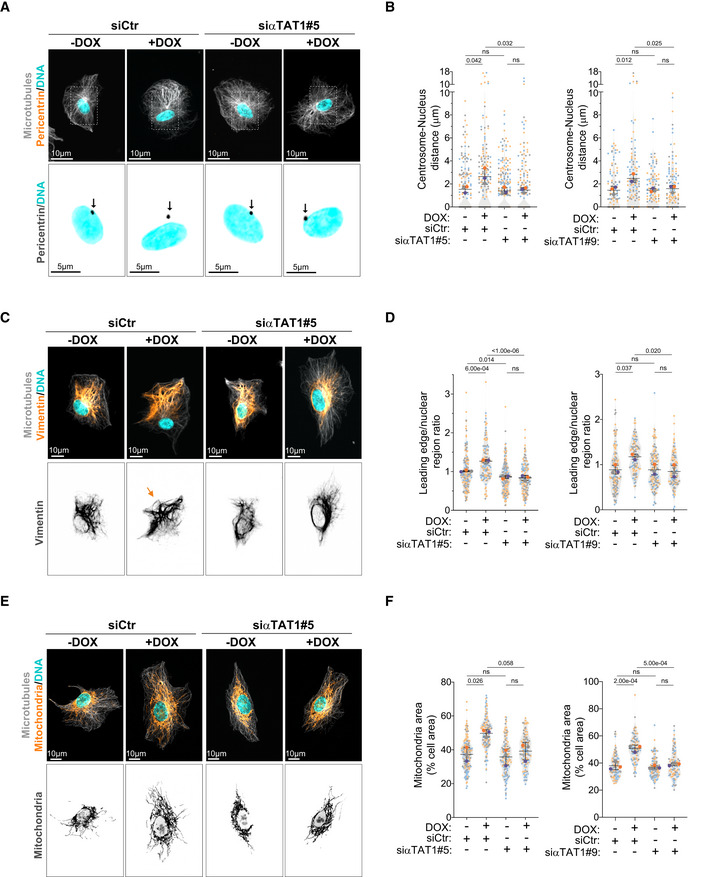
αTAT1‐dependent microtubule acetylation controls the displacement of centrosomes, vimentin, and mitochondria downstream of centrosome amplification Representative images of cells stained for centrosomes (Pericentrin, orange), microtubules (α‐tubulin, gray), and DNA (Hoechst, cyan) upon depletion of αTAT1. Scale bar: 10 μm. Black arrows indicate the position of the centrosome(s). Scale bar: 5 μm.Quantification of centrosome‐nucleus distance (Left panel: *n*_(−DOX siCtr)_ = 164; *n*_(+DOX siCtr)_ = 158; *n*_(−DOX siαTAT1#5)_ = 235; *n*_(+DOX siαTAT1#5)_ = 173; Right panel: *n*_(−DOX siCtr)_ = 107; *n*_(+DOX siCtr)_ = 119; *n*_(−DOX siαTAT1#9)_ = 110; *n*_(+DOX siαTAT1#9)_ = 118).Representative images of cells stained for vimentin (orange), microtubules (α‐tubulin, gray), and DNA (Hoechst, cyan) upon depletion of αTAT1. Orange arrows indicate the displacement of vimentin towards cell periphery. Scale bar: 10 μm.Quantification of vimentin leading edge/nuclear ratio (Left panel: *n*_(−DOX siCtr)_ = 164; *n*_(+DOX siCtr)_ = 123; *n*_(−DOX siαTAT1#5)_ = 179; *n*_(+DOX siαTAT1#5)_ = 137; Right panel: *n*_(−DOX siCtr)_ = 144; *n*_(+DOX siCtr)_ = 109; *n*_(−DOX siαTAT1#9)_ = 130; *n*_(+DOX siαTAT1#9)_ = 117).Representative images of cells stained for mitochondria (MitoTracker, orange), microtubules (α‐tubulin, gray), and DNA (Hoechst, cyan) upon depletion of αTAT1. Scale bar: 10 μm.Quantification of mitochondria area (Left panel: *n*_(−DOX siCtr)_ = 169; *n*_(+DOX siCtr)_ = 125; *n*_(−DOX siαTAT1#5)_ = 130; *n*_(+DOX siαTAT1#5)_ = 134; Right panel: *n*_(−DOX siCtr)_ = 91; *n*_(+DOX siCtr)_ = 89; *n*_(−DOX siαTAT1#9)_ = 101; *n*_(+DOX siαTAT1#9)_ = 89). Data information: For all graphs, error bars represent mean ± SD from three independent experiments. *P*‐values are described in the graphs, ns = not significant (*P* > 0.05). The following statistics were applied: one‐way ANOVA with Tukey's *post hoc* test for all graphs. *n* = number of cells analyzed. Source data are available online for this figure.

**Figure EV4:**
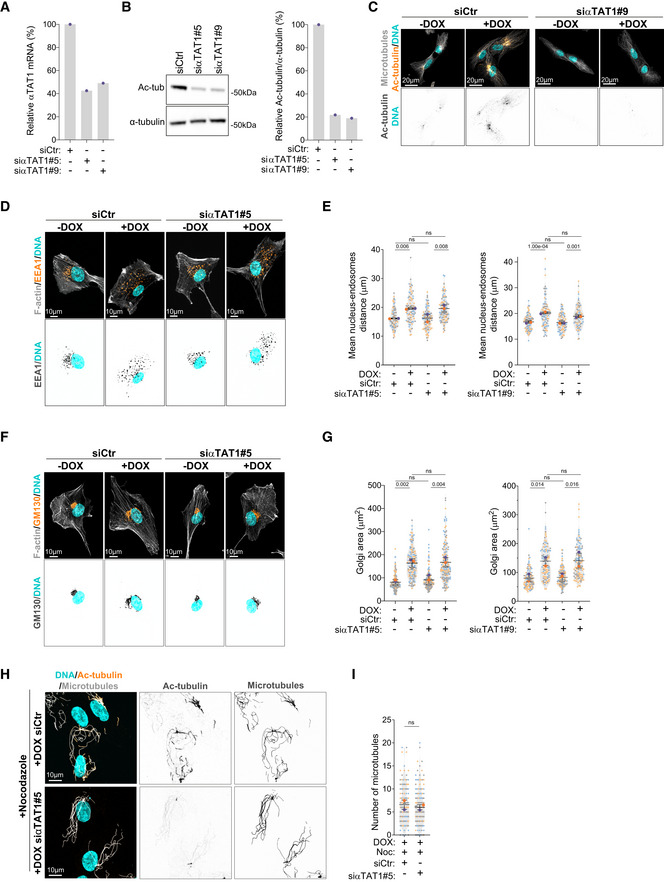
Endosome displacement and Golgi dispersion do not rely on tubulin acetylation Quantification of αTAT1 mRNA expression in cells treated with siRNA against αTAT1 (two independent siRNAs; #5 and #9).Left panel; immunoblot for α‐tubulin and acetylated tubulin (Ac‐tub) upon αTAT1 depletion (two independent siRNAs; #5 and #9). Right panel; percentage of acetylated tubulin relative to total α‐tubulin.Representative images of cells stained for microtubules (α‐tubulin; gray), acetylated tubulin (Ac‐tubulin, orange) and DNA (Hoechst, cyan) upon αTAT1 depletion. Scale bar: 20 μm.Representative images of cells stained for early endosomes (EEA1, orange), F‐actin (phalloidin, gray), and DNA (Hoechst, cyan) upon αTAT1 depletion. Scale bar: 10 μm.Quantification of endosome‐nucleus distance (Left panel: *n*_(−DOX siCtr)_ = 88; *n*_(+DOX siCtr)_ = 90; *n*_(−DOX siαTAT1#5)_ = 91; _(+DOX siαTAT1#5)_ = 98; Right panel: *n*_(−DOX siCtr)_ = 70; *n*_(+DOX siCtr)_ = 64; *n*_(−DOX siαTAT1#9)_ = 69; _(+DOX siαTAT1#9)_ = 67).Representative images of cells stained for Golgi (GM130, orange), F‐actin (phalloidin, gray), and DNA (Hoechst, cyan) upon αTAT1 depletion. Scale bar: 10 μm.Quantification of Golgi area (Left panel: *n*_(−DOX siCtr)_ = 177; *n*_(+DOX siCtr)_ = 147; *n*_(−DOX siαTAT1#5)_ = 172; *n*_(+DOX siαTAT1#5)_ = 156; Right panel: *n*_(−DOX siCtr)_ = 103; *n*_(+DOX siCtr)_ = 103; *n*_(−DOX siαTAT1#9)_ = 128; *n*_(+DOX siαTAT1#9)_ = 109).Representative images of cells treated with siRNA against αTAT1, stained for microtubules (α‐tubulin, cyan), acetylated tubulin (Ac‐tubulin, orange) and DNA (Hoechst, gray) upon nocodazole treatment (Noc, 2 μM). Scale bar: 10 μm.Quantification of microtubule numbers (*n*_(+DOX siCtr+Noc)_ = 145; *n*_(+DOX siαTAT1+Noc)_ = 159). Data information: For all graphs, error bars represent mean ± SD from three independent experiments. *P*‐values are described in the graphs, ns = not significant (*P* > 0.05). The following statistics were applied: one‐way ANOVA with Tukey's *post hoc* test for graphs in (E) and (G) and unpaired *t*‐test for graph in (I). *n* = number of cells analyzed. Source data are available online for this figure.

We next investigated whether tubulin acetylation plays a role in the displacement of intracellular compartments observed in cells with amplified centrosomes by targeting the main tubulin acetyltransferase in mammalian cells, αTAT1, which acetylates lysine 40 (K40) on α‐tubulin (Akella *et al*, 2010; Shida *et al*, 2010). Using two independent siRNAs against αTAT1 (#5 and #9), we greatly reduced the levels of αTAT1 mRNA and more importantly, tubulin acetylation was efficiently blocked (Fig EV4A–C). Depletion of αTAT1 rescued centrosome displacement in cells with amplified centrosomes (Fig 4A and B). Similarly, vimentin and mitochondria displacement towards the cell periphery were also suppressed following αTAT1 depletion (Fig 4C–F). However, not all membrane‐bound organelles were sensitive to tubulin acetylation. EEA1‐positive endosomes and Golgi displacement were not prevented by αTAT1 depletion (Fig EV4D–G), indicating that displacement of these organelles in response to centrosome amplification is regulated by a different mechanism that does not involve tubulin acetylation. Notably, in control cells, αTAT1 depletion had no impact on the distribution of any of the intracellular compartments measured, which is consistent with the low levels of acetylated tubulin observed in these cells. This is in striking contrast to what is observed upon depletion of KIF5B and p150, which significantly impact intracellular organization (Figs 1G and 2E and J). Thus, it is crucial to consider that the role of αTAT1 and tubulin acetylation might be context dependent or in response to specific challenges/stresses. We also observed that αTAT1 depletion did not prevent the formation of stable, nocodazole‐resistant microtubules in cells with amplified centrosomes (Fig EV4H and I). Thus, the role of tubulin acetylation in kinesin‐1‐mediated organelle displacement is independent of increased microtubule stabilization.

**Figure 5:**
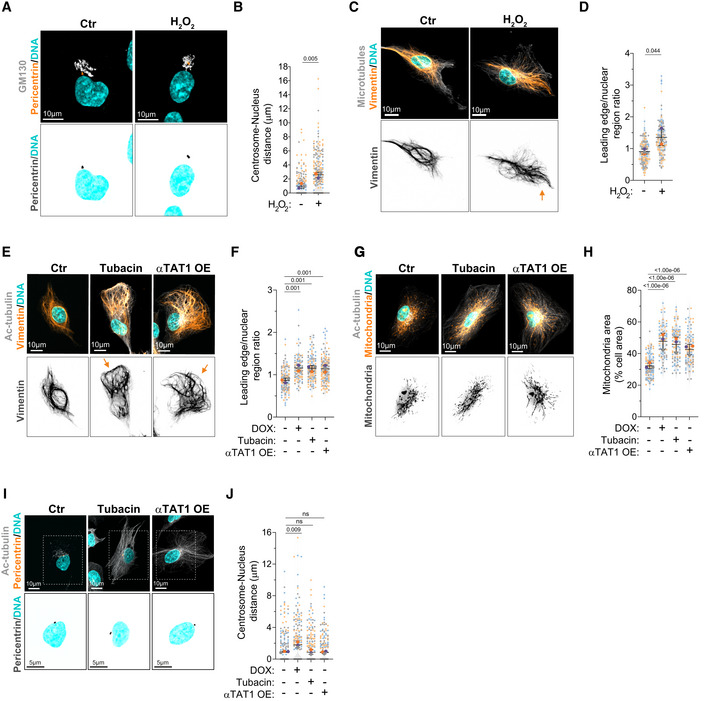
Increased tubulin acetylation is sufficient to promote vimentin and mitochondria displacement independently of centrosome amplification Representative images of cells stained for centrosomes (Pericentrin, orange), Golgi (GM130, gray), and DNA (Hoechst, cyan) treated with H_2_O_2_ (75 μM). Scale bar: 10 μm.Quantification of centrosome‐nucleus distance (*n*_(Ctr)_ = 192; *n*_(H2O2)_ = 211).Representative images of cells stained for vimentin (orange), microtubules (α‐tubulin, gray), and DNA (Hoechst, cyan) treated with H_2_O_2_. Orange arrows indicate the displacement of vimentin towards cell periphery. Scale bar: 10 μm.Quantification of vimentin leading edge/nuclear ratio (*n*_(Ctr)_ = 132; *n*_(H2O2)_ = 117).Representative images of cells stained for vimentin (orange), acetylated tubulin (Ac‐tubulin, gray), and DNA (Hoechst, cyan) treated with Tubacin (5 μM) or overexpressing eGFP‐αTAT1 (αTAT1 OE). Orange arrows indicate the displacement of vimentin towards cell periphery. Scale bar: 10 μm.Quantification of vimentin leading edge/nuclear ratio (*n*_(−DOX)_ = 108; *n*_(+DOX)_ = 95; *n*_(Tubacin)_ = 137; *n*_(αTAT1 OE)_ = 123).Representative images of cells stained for mitochondria (MitoTracker, orange), acetylated tubulin (Ac‐tubulin, gray), and DNA (Hoechst, cyan) treated with Tubacin or overexpressing eGFP‐αTAT1 (αTAT1 OE). Scale bar: 10 μm.Quantification of mitochondria area (*n*_(−DOX)_ = 102; *n*_(+DOX)_ = 86; *n*_(Tubacin)_ = 90; *n*_(αTAT1 OE)_ = 101).Representative images of cells stained for centrosomes (Pericentrin, orange), acetylated tubulin (Ac‐tubulin, gray), and DNA (Hoechst, cyan) treated with Tubacin or overexpressing eGFP‐αTAT1 (αTAT1 OE). Scale bar: 10 μm. Inset scale bar: 5 μm.Quantification of centrosome‐nucleus distance (*n*_(−DOX)_ = 270; *n*_(+DOX)_ = 222; *n*_(Tubacin)_ = 248; *n*_(αTAT1 OE)_ = 288). Data information: For all graphs, error bars represent mean ± SD from three independent experiments. *P*‐values are described in the graphs, ns = not significant (*P* > 0.05). The following statistics were applied: unpaired *t*‐test for graphs in (B) and (D) and one‐way ANOVA with Tukey's *post hoc* test for graphs in (F), (H), and (J). *n* = number of cells analyzed. Source data are available online for this figure.

**Figure 6:**
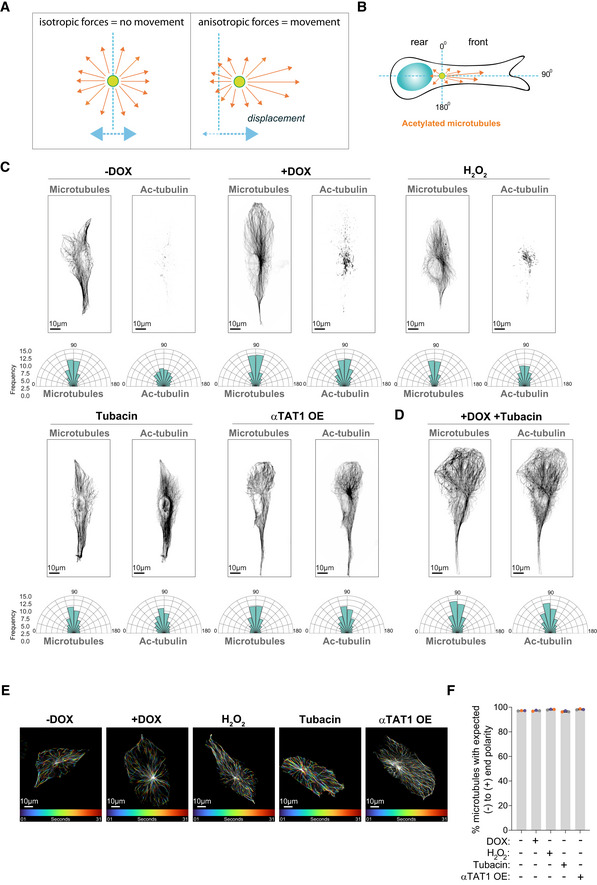
Polarization of total and acetylated microtubules in cells with increased levels of acetylated microtubules Scheme representing how distribution of forces could impact movement/displacement of the centrosome.Scheme depicting how orientation of total and acetylated microtubules was determined in cells.Top: Representative images of total and acetylated microtubules (gray) in control cells (−DOX), cells with amplified centrosomes (+DOX), treated with H_2_O_2_ or Tubacin and overexpressing eGFP‐αTAT1 (αTAT1 OE). Scale bar: 10 μm. Bottom panel: Rose plots displaying the frequency of total and acetylated microtubules orientation in the cell front (*n*_(−DOX)_ = 61; *n*_(+DOX)_ = 48; *n*_(H2O2)_ = 84; *n*_(Tubacin)_ = 89; *n*_(αTAT1 OE)_ = 70).Top: Representative images of total and acetylated microtubules (gray) in cells with amplified centrosomes treated with Tubacin (+DOX + Tubacin). Bottom panel: Rose plots displaying the frequency of total and acetylated microtubules orientation in the cell front (*n*_(+DOX Tubacin)_ = 73).Representative images of color‐coded temporal projection of EB3‐GFP comets over 30 s. Scale bar: 10 μm.Quantification of the percentage of EB3‐GFP comets per cell that follow normal (−) to (+) end polarity assessed by live‐cell imaging (*n*_(−DOX)_ = 25; *n*_(+DOX)_ = 23; *n*_(H2O2)_ = 25; *n*_(Tubacin)_ = 23; *n*_(αTAT1 OE)_ = 42). Data information: For the graph in (F), error bars represent mean ± SD from three independent experiments. *n* = number of cells analyzed. Source data are available online for this figure.

**Figure 7:**
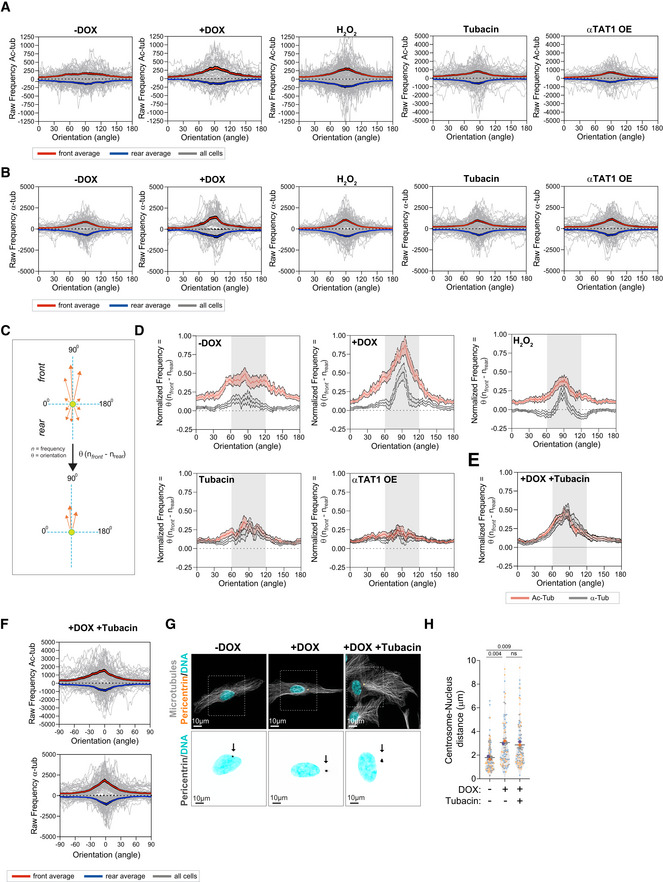
Polarized distribution of total and acetylated tubulin correlates with centrosome displacement Raw frequency of the distribution of acetylated microtubules orientation towards cell front (+ve values) and rear (−ve values) for all individual cells (gray lines). Red line represents the average for front orientations and blue line the averages for rear orientations for all cells (*n*_(−DOX)_ = 61; *n*_(+DOX)_ = 48; *n*_(H2O2)_ = 84; *n*_(Tubacin)_ = 89; *n*_(αTAT1 OE)_ = 70).Raw frequency of the distribution of total microtubules orientation towards cell front (+ve values) and rear (−ve values) for all individual cells (gray lines). Red line represents the average for front orientations and blue line the averages for rear orientations for all cells (*n*_(−DOX)_ = 61; *n*_(+DOX)_ = 48; *n*_(H2O2)_ = 84; *n*_(Tubacin)_ = 89; *n*_(αTAT1 OE)_ = 70).Scheme depicting how subtraction of rear from front values was determined and how this could be used as a measure for polarized microtubule distribution.Quantification of the distribution of acetylated microtubule orientation upon subtracting rear values from front values (*n*_(−DOX)_ = 61; *n*_(+DOX)_ = 48; *n*_(H2O2)_ = 84; *n*_(Tubacin)_ = 89; *n*_(αTAT1 OE)_ = 70).Quantification of the distribution of acetylated microtubule orientation upon subtracting rear values from front values (*n*_(+DOX Tubacin)_ = 73).Raw frequency of the distribution of acetylated microtubules (Left) and total microtubules (Right) orientation towards cell front (+ve values) and rear (−ve values) for all individual cells (gray lines). Red line represents the average for front orientations and blue line the averages for rear orientations for all cells (*n*_(+DOX Tubacin)_ = 73).Representative images of cells stained for centrosomes (Pericentrin, orange), microtubules (α‐tubulin, gray), and DNA (Hoechst, cyan). Scale bar: 10 μm. Black arrows indicate the position of the centrosome(s). Inset scale bar: 10 μm.Quantification of nucleus‐centrosome distance (*n*_(−DOX)_ = 108; *n*_(+DOX)_ = 87; *n*_(+DOX+Tubacin)_ = 97). Data information: For the graph in (H), error bars represent mean ± SD from three independent experiments. *P*‐values are described in the graphs, ns = not significant (*P* > 0.05). The following statistics was applied: one‐way ANOVA with Tukey's *post hoc*. *n* = number of cells analyzed. Source data are available online for this figure.

**Figure EV5:**
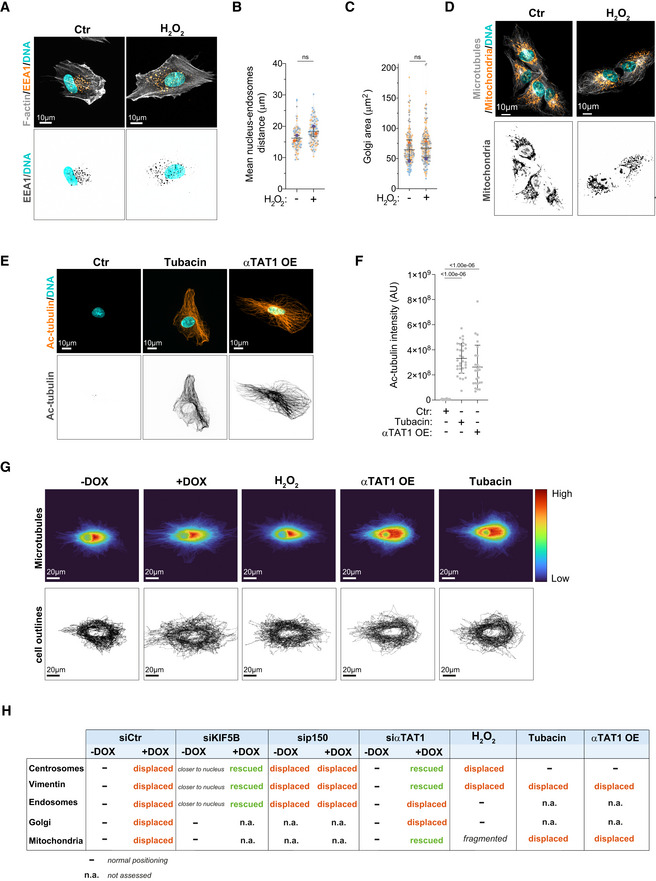
H_2_O_2_‐treated cells do not display endosome displacement or Golgi dispersion Representative images of cells stained for early endosomes (EEA1, orange), F‐actin (phalloidin, gray), and DNA (Hoechst, cyan) treated with H_2_O_2_. Scale bar: 10 μm.Quantification of endosomes‐nucleus distance (*n*_(Ctr)_ = 82; *n*_(H2O2)_ = 79).Quantification of Golgi area (*n*_(Ctr)_ = 151; *n*_(H2O2)_ = 157).Representative images of cells stained for mitochondria (MitoTracker, orange), microtubules (α‐tubulin, gray), and DNA (Hoechst, cyan) treated with H_2_O_2_ (75 μM). Scale bar: 10 μm.Representative images of cells stained for acetylated tubulin (Ac‐tubulin, orange) and DNA (Hoechst, cyan) treated with Tubacin (5 μM) or overexpressing eGFP‐αTAT1 (αTAT1 OE). Scale bar: 10 μm.Quantification of acetylated tubulin fluorescence intensity (*n*_(Ctr)_ = 29; *n*_(Tubacin)_ = 32; *n*_(αTAT1 OE)_ = 25).Top: Heat map of α‐tubulin distribution in 50 cells from each condition. Bottom: Outline of all cells (based on α‐tubulin signal). Cells were superimposed using the center of the nucleus as reference point. Scale bar: 20 μm.Table summarizing the effect of different treatments on intracellular organization. Data information: For all graphs, error bars represent mean ± SD from three independent experiments. *P*‐values are described in the graphs, ns = not significant (*P* > 0.05). The following statistics were applied: unpaired *t*‐test for graphs in (B) and (C) and one‐way ANOVA with Tukey's *post hoc* test for graph in (F). *n* = number of cells analyzed. Source data are available online for this figure.

To test whether microtubule acetylation was sufficient to promote intracellular reorganization, we treated cells with low doses of H_2_O_2_, which led to similar levels of tubulin acetylation observed in cells with amplified centrosomes without changing total tubulin levels (Fig 3H and I). H_2_O_2_ treatment also promoted centrosome and vimentin displacement (Fig 5A–D), but no effect was observed on endosome displacement or Golgi dispersion (Fig EV5A–D). Interestingly, even in cells that exhibited centrosome displacement, Golgi positioning was unchanged (Fig 5A), further demonstrating that these phenotypes are not co‐dependent. Because H_2_O_2_ treatment induces mitochondria fragmentation (Fan *et al*, 2010), we were unable to assess mitochondria displacement in this condition (Fig EV5D). Additionally, we tested whether inducing higher levels of tubulin acetylation by treating cells with Tubacin, an inhibitor of the deacetylase HDAC6, or by overexpressing αTAT1 (Fig EV5E and F) could induce similar phenotypes (Haggarty *et al*, 2003; Shida *et al*, 2010). Indeed, cells treated with Tubacin or overexpressing αTAT1 (αTAT1 OE) showed similar displacement of vimentin and mitochondria to cells with amplified centrosomes (Fig 5E–H). Vimentin displacement is an unlikely consequence of cell shape alterations. Superimposing either all cells stained for total tubulin or the outlines of all cells analyzed demonstrates that no major changes in cell polarization, shape, or size are observed (Fig EV5G). Surprisingly, however, centrosomes remained closely associated with the nuclear envelope, suggesting that high levels of tubulin acetylation alone may not be sufficient to promote their displacement (Figs 5I and J, and EV5H). What could explain this difference? We hypothesized that, in addition to increased levels, the distribution or orientation of acetylated microtubules could differentially impact centrosome positioning. As a single, discrete organelle from where microtubules emanate, centrosomes are uniquely surrounded by microtubules and thus we postulated that centrosome displacement would likely be dependent on the distribution or polarization of motor‐mediated pushing forces. For example, an isotropic distribution of pushing forces around the centrosome would cancel each other, thereby preventing its displacement (Fig 6A). By contrast, if the microtubule network, and specifically acetylated microtubules, became polarized then this could lead to the anisotropic distributions of forces required to displace the centrosome (Fig 6A). This model could also explain why other intracellular components, such as vimentin and mitochondria, which are usually transported along single microtubules (Friedman *et al*, 2010; Hookway *et al*, 2015), would be less sensitive to the distribution of pushing forces. To evaluate this, we divided the cell as rear and front based on the centrosome position and quantified the distribution of orientation frequencies for both total and acetylated microtubules (Schindelin *et al*, 2012; preprint: Li *et al*, 2022; Fig 6B and C). From these analyses, and most clearly visualized in the rose plots, we observed that the orientation frequencies of total and acetylated microtubules are polarized towards the leading edge in all conditions, apart from control cells where tubulin acetylation levels are very low and no polarization was observed for acetylated microtubules (Fig 6C and D). Furthermore, microtubule minus‐ to plus‐end polarity, which could affect kinesin‐1‐mediated transport (Tas *et al*, 2017), is also unchanged by increased tubulin acetylation. Using EB3‐GFP or EB3‐tdTomato expressing cells to track microtubule plus ends, we found no major differences in microtubule growth patterns, with more than 97% of all EB3 comets having the expected polarity (minus‐end: cell center, plus‐end: cell periphery; Fig 6E and F, Movies EV1 and EV2). However, this analysis does not take into account the asymmetrical distribution of tubulin acetylation between the front and rear of the cell. For this, we plotted the frequency of orientation variations in the cell front (values > 0) and rear (values < 0) for all cells and saw that the distribution of total and acetylated microtubules between the front and rear varies between the different conditions (Fig 7A and B). To quantify these differences, for each orientation, we subtracted the rear normalized frequency from the matching front normalized frequency (Fig 7C). Interestingly, in all conditions, there is an increase in the frequency of orientations towards the front of the cell, which follows the distribution of total microtubules. In addition, only in cells with amplified centrosomes or treated with H_2_O_2,_ a clear and organized polarization of these frequencies towards the leading edge (~60° to 120°) can be observed (Fig 7D). Thus, with the assumption that increased tubulin acetylation results in increased kinesin‐1 pushing forces, our data fit with a model whereby an anisotropic and organized distribution of acetylated microtubules is key for centrosome displacement, which is only observed in cells with amplified centrosomes or treated with H_2_O_2_.

To further test our model, we treated cells with amplified centrosomes, which display a polarization of total and acetylated microtubules towards the leading edge (Figs 6C and 7D), with Tubacin (+DOX + Tubacin) to determine its impact on the distribution of acetylated microtubules and centrosome displacement (Fig 6D). We found that the presence of extra centrosomes is indeed sufficient to maintain the polarized distribution of total and acetylation microtubules towards the leading edge, even when most microtubules are acetylated as a result of Tubacin treatment (Fig 7E and F). Remarkably, the polarization of the microtubule network induced by centrosome amplification is sufficient to drive centrosome displacement in cells treated with Tubacin (Fig 7G and H), supporting a model where centrosome displacement requires an anisotropic and organized distribution of acetylated microtubules. These results demonstrate that the displacement of centrosomes, vimentin, and mitochondria towards the leading edge is regulated by tubulin acetylation and that, both increased and distribution of acetylated microtubules could differentially impact these phenotypes.

### Intracellular reorganization in cells with amplified centrosomes correlates with enhanced nuclear deformability

**Figure 8:**
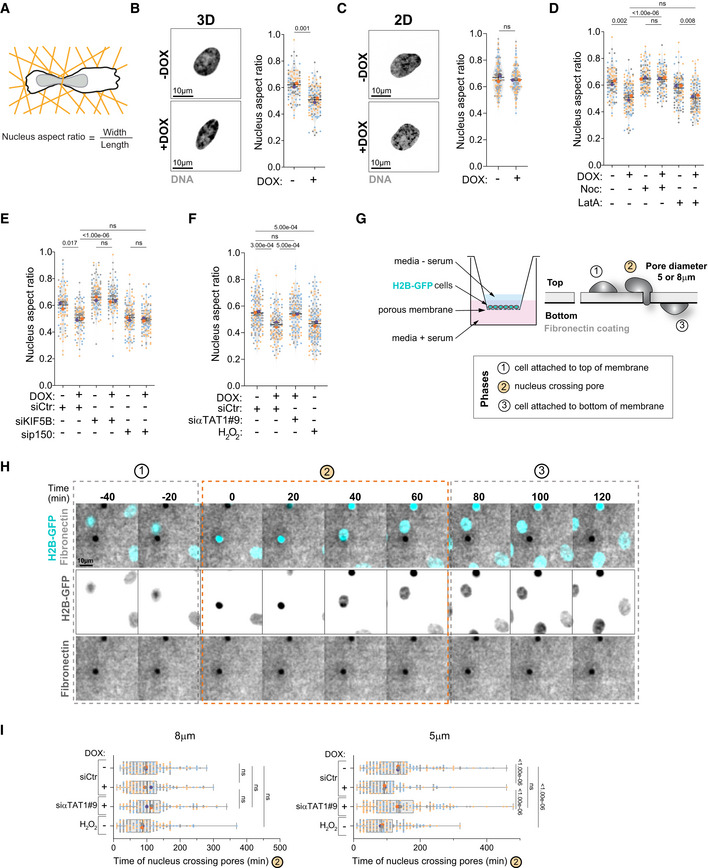
Centrosome amplification enhances nucleus deformation and promotes efficient nucleus translocation through small constrictions Representative scheme of nucleus aspect ratio quantification.Left: Representative images of the nucleus (Hoechst, gray) in control cells (−DOX) and upon induction of extra centrosomes (+DOX) in 3D. Scale bar: 10 μm. Right: Quantification of nucleus aspect ratio in 3D (*n*_(−DOX)_ = 100; *n*_(+DOX)_ = 62).Left: Representative images of the nucleus (Hoechst, gray) in control cells (−DOX) and upon induction of extra centrosomes (+DOX) in 2D. Scale bar: 10 μm. Right: Quantification of nucleus aspect ratio in 2D (*n*_(−DOX)_ = 114; *n*_(+DOX)_ = 102).Quantification of nucleus aspect ratio (*n*_(−DOX)_ = 90; *n*_(+DOX)_ = 114; *n*_(−DOX Noc)_ = 112; *n*_(+DOX Noc)_ = 101; *n*_(−DOX LatA)_ = 110; *n*_(+DOX LatA)_ = 108).Quantification of nucleus aspect ratio (*n*_(−DOX siCtr)_ = 95; *n*_(+DOX siCtr)_ = 107; *n*_(−DOX siKIF5B)_ = 111; *n*_(+DOX siKIF5B)_ = 91; *n*_(−DOX sip150)_ = 115; *n*_(+DOX sip150)_ = 117).Quantification of nucleus aspect ratio (*n*_(−DOX siCtr)_ = 148; *n*_(+DOX siCtr)_ = 129; *n*_(+DOX siαTAT1#9)_ = 134; *n*_(H2O2)_ = 154).Representative scheme of the Transwell system and nucleus crossing constrictions.Still images from live‐cell imaging depicting cell nucleus (H2B‐GFP, cyan) crossing pores on membranes coated with fibronectin (gray). Time spent in phase 2 is depicted in the graph in (I). Scale bar: 10 μm.Quantification of time spent by the nucleus to cross 5‐μm or 8‐μm diameter constrictions (phase 2) (Left panel, 8‐μm pores: *n*_(−DOX siCtr)_ = 180; *n*_(+DOX siCtr)_ = 132; *n*_(+DOX siαTAT1#9)_ = 160; *n*_(H2O2)_ = 153; Right panel, 5‐μm pores: *n*_(−DOX siCtr)_ = 178; *n*_(+DOX siCtr)_ = 150; *n*_(+DOX siαTAT1#9)_ = 196; *n*_(H2O2)_ = 172). Data information: For all graphs, error bars represent mean ± SD from three independent experiments. For graph in (I), vertical line represents the median and whiskers the minimum (left quartile) and maximum values (right quartile). *P*‐values are described in the graphs, ns = not significant (*P* > 0.05). The following statistics were applied: unpaired *t*‐test for graphs in (B) and (C) and one‐way ANOVA with Tukey's *post hoc* test for graphs in (D, E, F, and I). *n* = number of cells analyzed.

Nucleus‐associated vimentin confers a protective role to the nucleus against mechanical stress and its loss enhances nuclear deformability (Patteson *et al*, 2019a,b). Thus, we hypothesized that intracellular reorganization resulting in vimentin displacement towards the cell periphery could promote nuclear deformability. Nucleus aspect ratio was used as a proxy for deformability, where 1 = perfect circle (Fig 8A). When plated in 3D confined collagen‐I matrices, cells with amplified centrosomes displayed lower nuclear circularity compared with control cells, suggesting increased nucleus deformability (Fig 8B). The same was not observed in cells plated in 2D, indicating that only in confined environments these differences can be observed (Fig 8C). Although actin has long been proposed to play a role in mediating nuclear deformation in different cell types (Thiam *et al*, 2016), treatment with latrunculin‐A did not prevent nuclear deformability in cells with amplified centrosomes. By contrast, microtubule depolymerization with nocodazole prevented nuclear deformability (Fig 8D). Moreover, KIF5B depletion also blocked increased nuclear deformability while p150^glued^ depletion did not affect cells with extra centrosomes but was sufficient to increase nuclear deformability in control cells (Fig 8E). These results suggest that changes in the balance of microtubule motors driven by centrosome amplification enhance nucleus deformation. Supporting this idea, blocking tubulin acetylation in cells with extra centrosomes by depleting αTAT1 was sufficient to prevent nuclear deformability, whereas increasing tubulin acetylation levels in control cells with low doses of H_2_O_2_ was sufficient to promote nuclear deformability (Fig 8F).

During cell migration through confined spaces, the nucleus, which is the largest and stiffest cellular organelle, constitutes a burden for cells (Denais *et al*, 2016; Raab *et al*, 2016). Thus, we hypothesized that the increased nuclear deformability in cells with amplified centrosomes could facilitate migration through confined spaces. To test this, we utilized a Transwell assay in which cells were seeded onto a porous membrane and allowed to migrate through pores of different sizes (5 or 8 μm). Using RPE‐1.iPLK4 cells expressing H2B‐GFP to visualize the nucleus, the speed of nuclear translocation through these pores (which we named phase 2) was assessed by live‐cell imaging (Fig 8G). We found that the time for the nucleus to cross the larger 8‐μm pores was similar in cells with normal and amplified centrosomes (−DOX = 97.05 ± 2.14 min; +DOX = 102.06 ± 9.61 min). Conversely, we found that cells with amplified centrosomes migrated significantly faster than control cells (−DOX) through 5‐μm pores (−DOX = 134.25 ± 1.22 min; +DOX = 93.04 ± 1.59 min). Depletion of αTAT1 not only prevented nuclear deformability in cells with extra centrosomes but also blocked faster migration through 5‐μm pores (+DOX siRNA αTAT1 = 136.44 ± 5.16 min), suggesting that intracellular organization downstream of tubulin acetylation plays a role in this process (Fig 8H and I). Consistent with this, treatment of control cells with H_2_O_2_ was sufficient to drive faster nuclear migration through 5‐μm pores (−DOX H_2_O_2_ = 84.68 ± 3.04 min). Our results suggest that increased nuclear deformability driven by tubulin acetylation provides a migratory advantage in constrained environments (Fig 8I). Altogether, these results indicate that changes in intracellular organization in cells with extra centrosomes could enhance nuclear deformation to facilitate migration through confined spaces.

## Discussion

Here, we demonstrate that centrosome amplification is sufficient to change intracellular organization, a process that requires kinesin‐1‐mediated transport and is partly regulated by increased tubulin acetylation. This intracellular reorganization mediated by tubulin acetylation increases nuclear deformability and facilitates nuclear migration through small constrictions. The differential impact of acetylated tubulin on organelle distribution highlights a more complex sensing and response mechanisms by which organelles read the tubulin code.

The centrosome positioning at the cell center and in close proximity to the nucleus has long been proposed to result from an equilibrium of pulling and pushing forces exerted by microtubule motors (Bornens, 2008). In late G2, inhibition of dynein leads to centrosome displacement away from the nucleus, from the cell center towards cell periphery, in a kinesin‐1‐dependent manner (Splinter *et al*, 2010), demonstrating that dynein functions as a brake that counteracts kinesin‐1 forces. Interestingly, we found that induction of centrosome amplification is sufficient to drive the displacement of centrosomes towards the cell periphery, phenocopying what has been observed in cells upon dynein inhibition. Depletion of the kinesin‐1 KIF5B prevents displacement of the centrosomes, implying that pushing forces on centrosomes are mediated by KIF5B and that these forces overcome the pulling activity of dynein. We also observed that, in response to centrosome amplification, several intracellular compartments are displaced towards the cell periphery, namely endosomes, mitochondria, vimentin, and Golgi. This global reorganization is consistent with previous observations in pancreatic cancer cells showing that centrosome amplification leads to the dispersion of late endosomes/multivesicular bodies towards the cell periphery (Adams *et al*, 2021). Displacement of endosomes and vimentin have also been shown to require kinesin‐1 (Gyoeva & Gelfand, 1991; Liao & Gundersen, 1998; Nath *et al*, 2007; Schmidt *et al*, 2009), potentially highlighting a kinesin‐1‐mediated global reorganization of the cytoplasm in cells with amplified centrosomes.

Systematic analyses of different intracellular compartments revealed that reduction of acetylated tubulin levels, via depletion of αTAT1, prevented the displacement of centrosomes, vimentin, and mitochondria towards the cell periphery, indicating that enhanced tubulin acetylation plays a role in the relocation of these intracellular compartments. By contrast, depletion of αTAT1 had no significant impact on endosomes and Golgi reorganization, suggesting that other microtubule PTMs and/or adaptor proteins, which link organelles to microtubules, could specifically affect the relocation of these organelles in cells with amplified centrosomes (Akhmanova & Hammer, 2010; Barlan & Gelfand, 2017; Cross & Dodding, 2019). Importantly, while endosome displacement requires kinesin‐1 in cells with amplified centrosomes, this was independent of tubulin acetylation, demonstrating that tubulin acetylation is not a general mechanism to regulate organelle transport. This is also consistent with the observation that endosomes do not localize to acetylated microtubules (Friedman *et al*, 2010). It is also possible that, in the absence of tubulin acetylation, other plus‐end kinesin motors, such as kinesin‐3, could transport endosomes (Wedlich‐Soldner *et al*, 2002; Bielska *et al*, 2014). Exactly how tubulin acetylation, which occurs in the microtubule lumen, favors kinesin‐1‐mediated organelle transport remains largely unknown and no direct link has been described to date. Our data demonstrate that microtubule stabilization is unlikely to be the answer since αTAT1 depletion in cells with amplified centrosomes does not prevent the formation of nocodazole‐resistant microtubules but is sufficient to prevent centrosomes, mitochondria, and vimentin displacement.

Unexpectedly, we found that not only increased levels but also the distribution of acetylated microtubules could contribute to organelle displacement. High levels of tubulin acetylation induced by Tubacin or αTAT1 overexpression, as compared to untreated control cells, promotes the displacement of vimentin and mitochondria towards cell periphery, but not centrosomes. This contrasts with what we observed in cells with amplified centrosomes or treated with H_2_O_2_, which induce lower levels of tubulin acetylation. These observations led us to propose a model whereby, in addition to increased levels, the distribution of forces, through changes in tubulin acetylation, is required to displace centrosomes. Because microtubules emanate from the centrosomes in all directions, we hypothesized that centrosome displacement is likely to be more sensitive to the distribution of acetylated microtubules and motor‐mediated pushing forces. While isotropic distribution of forces would cancel each other and block centrosome displacement, an anisotropic distribution of forces, promoted by the polarized distribution of acetylated microtubules, would lead to centrosome displacement. According to this, displacement of intracellular components that move along single or bundled microtubules could be much less sensitive to the distribution or polarization of pushing forces. Indeed, and in contrast to cells treated with Tubacin or overexpressing αTAT1, we observed that in cells harboring amplified centrosomes or treated with H_2_O_2_ both acetylated and total microtubules display a polarized distribution towards the leading edge, which is correlated with centrosome displacement. In support of this idea, cells with amplified centrosomes and treated with Tubacin to promote high levels of tubulin acetylation, retained a polarized organization of total and acetylated microtubules and exhibited centrosome displacement. These data demonstrate that it is the polarization of acetylated microtubules that is key for centrosome displacement and not the amount of acetylated microtubules. These observations could also explain why centrosome displacement, unlike other intracellular compartments, is exquisitely sensitive to cells plated in 3D environments that promote cell elongation/polarization. While our data are only suggestive of such model, it raises an important issue when assessing the role of microtubule PTMs in cells, that not all conditions that increase specific PTMs may elicit the exact same phenotype and that distribution and organization of modified microtubules should be taken into consideration.

To date, direct evidence supporting a role for tubulin acetylation in kinesin‐1‐mediated transport is still limited. In neurons, where long‐distance intracellular transport is required, subpopulations of acetylated microtubules are important to drive polarized kinesin‐1‐mediated transport of cargoes, such as JNK‐interacting protein 1 (JIP1) to a subset of neurites (Reed *et al*, 2006). Furthermore, both acetylation and microtubule orientation were shown to drive kinesin‐1 transport along the axon (Tas *et al*, 2017). During SV‐40 infection, the transport of viruses from the ER to the cytosol, which is crucial for infection, is mediated by the ability of kinesin‐1 to move along acetylated microtubules (Ravindran *et al*, 2017). Thus, it is possible that the regulation of kinesin‐1 transport by acetylated microtubules occurs in specific contexts/conditions. Indeed, our data show that while loss of tubulin acetylation has no impact on intracellular organization in untreated control cells, upon induction of centrosome amplification acetylated tubulin is key to promote the transport of centrosomes, vimentin, and mitochondria towards the cell periphery.

What are the consequences of this intracellular reorganization in cells with amplified centrosomes? Extensive changes in cell shape occur as cells migrate, and this is accompanied by relocations of several organelles and cellular compartments (Bornens, 2008). Previous work demonstrated that vimentin knockout in mouse embryonic fibroblasts (MEFs) have lower perinuclear stiffness and enhanced migration through microchannels (10‐ to 20‐μm width) (Patteson *et al*, 2019a). It is therefore plausible that during migration, displacement of vimentin towards cell periphery could lead to nuclear deformation to facilitate migration through confined spaces. Consistently, cells with amplified centrosomes display increased nuclear deformability in confined 3D collagen gels and move faster through smaller pores, which can be prevented by loss of acetylated microtubules through αTAT1 depletion. Moreover, extreme nuclear deformability has also been observed in invasive MCF10A cells with extra centrosomes migrating through thin invasive protrusions (Godinho *et al*, 2014). However, complete loss of vimentin leads to extensive nuclear rupture and DNA damage in MEFs migrating through confined spaces, suggesting that vimentin may confer mechanical resistance to protect the nucleus (Patteson *et al*, 2019b). In RPE‐1 cells, depletion of vimentin led to a severe nuclear deformation phenotype, consistent with nuclear rupture observed in vimentin‐knockout MEFs (Patteson *et al*, 2019b), and defects of nuclear migration through 5‐μm pores (Appendix Fig S1). Thus, it is tantalizing to propose that vimentin displacement, rather than its loss, could help migration through confined spaces in a more controlled manner while preventing extensive nuclear rupture and DNA damage.

It has been recently proposed that the binding of the ER to glutamylated microtubules plays a role in orchestrating the movement and positioning of several organelles, in an attempt to centralize intracellular organization (Zheng *et al*, 2022). However, this is unlikely to be a general feature. While ER tubules were shown to slide preferentially along acetylated microtubules (Friedman *et al*, 2010), our findings demonstrate that not all organelles respond to the same extent to tubulin acetylation. This indicates that, depending on the context, individual organelles must have their own sensing and response mechanisms to ensure fine‐tuning of their distribution in cells. We propose that this fine‐tuning, which can be promoted by microtubule PTMs, enables cells to adapt to different stimuli and environments.

## Materials and Methods

### Cell culture

Human hTERT‐RPE‐1 (human retinal epithelial; RRID: CVCL_4388; RPE‐1) cells were grown in Dulbecco's modified Eagle's medium/nutrient mixture F‐12 Ham (DMEM‐F12; Sigma) supplemented with 10% Fetal Bovine Serum (FBS; Gibco) and 100 U/ml Penicillin/Streptomycin (P/S; Gibco) and maintained at 37°C with 5% CO_2_ atmosphere. Tetracycline‐free FBS (Gibco) was used to grow cells expressing the PLK4 tet‐inducible construct. The FBS was heat inactivated at 56°C water bath for 30 min. RPE‐1 cells were routinely tested for mycoplasma.

### Plasmids and cell lines

RPE‐1.iPLK4 and RPE‐1.iPLK4^1‐608^ cell lines were generated using pLenti‐CMV‐TetR‐Blast lentiviral vector (Addgene, 17492) and selected using Blasticidin (10 μg/ml). Postselection, cells were then infected with a lentiviral vector containing either PLK4 WT or PLK4^1‐608^ mutant cDNA, which had been previously cloned into the pLenti‐CMV/TO‐Neo‐Dest vector and selected using Geneticin (200 μg/ml; Godinho *et al*, 2014). Cells expressing the PLK4 WT and PLK4^1‐608^ mutant transgenes were then induced for 48 h using 2 μg/ml of Doxycycline. The LV‐GFP plasmid (Addgene, 25999) was used to express H2B‐GFP, and cells were selected by FACS (Beronja *et al*, 2010). eGFP‐αTAT1 was prepared from pEF5B‐FRT‐GFP‐αTAT1 (Addgene, 27099) by PCR with BamHI and SalI restriction sites on the 5′ and 3′ end, respectively, and cloned into pLV‐eGFP (Addgene, 36083). The EB3‐GFP (pKan‐CMV‐Mapre3‐GFP) and EB3‐tdTomato (pKan‐CMV‐Mapre3‐tdTomato) pLenti plasmids were kind gifts from Anne Straube (Roth *et al*, 2018). All lentiviral plasmids are amplified in One‐shot Stbl3 chemically competent *E. coli* (Thermo Fisher Scientific, C7373‐03). Primers used for cloning eGFP‐αTAT1 are listed below:

**Table:** 

Primer	Sequence
BamHI_aTAT1_eGFP_Forward	TATATGGATCCACCATGGTGAGCAAGGGCGAGGAGC
alI_aTAT1_Reverse	ATATAGTCGACTTAGTATCGACTCTCCTCAGAGCGG

### Lentiviral generation

To generate lentivirus, HEK‐293 cells were plated in antibiotic‐free medium. Transfection of the appropriate lentiviral plasmid in combination with Gag‐Pol (psPAX2, Addgene, 12260) and VSV‐G (VSV‐G: pMD2.G, Addgene, 12259) was performed using Lipofectamine 2000^®^ (Thermo Fisher Scientific), as per the manufacturer's specifications. The resultant lentivirus was harvested 24 and 48 h postinfection, passed through a 0.4‐μM syringe filter, and stored in cryovials at −80°C. For infection, the appropriate lentivirus was then mixed with 8 μg/ml polybrene before being added to the cells in a dropwise fashion. Infection was repeated the following day and antibiotic selection started 24 h after final infection.

### Chemicals

Chemicals and treatments were performed as follows: 2 μg/ml Doxycycline hyclate (DOX; Sigma) treatment for 48 h, 75 μM hydrogen peroxide (H_2_O_2_; Sigma) treatment for 4 h, 0.5 mM of Apocynin (Santa Cruz) treatment for 72 h (added at the same time as DOX), 0.1 μM latrunculin‐A (LatA; Sigma) treatment for 5 h, 10 μM nocodazole (Noc; Sigma) treatment for 5 h to completely depolymerize microtubules and 2 μM nocodazole for 30 min to assess the numbers of microtubules resistant to Noc, 5 μM of Tubacin (Sigma) was added for 4 h before fixation.

### siRNA transfection

siRNA transfection was performed in antibiotic‐free growth medium using Lipofectamine^®^ RNAi MAX (Thermo Fisher) as per the manufacturer's instructions. Briefly, cells were grown in a 6‐well plate until reaching ~60% confluency. Prior to transfection, growth medium was replaced with 2 ml of fresh growth medium without antibiotics. For each well, the transfection solution was prepared as followed: 10 μl of Lipofectamine^®^ RNAi MAX Transfection Reagent was diluted in 250 μl of Reduced Serum Medium Opti‐MEM^®^ (Thermo Fisher) in a sterile 1.5 ml microcentrifuge tube and 5 μl of siRNA at 20 μM was diluted in 250 μl of Opti‐MEM^®^ in a separate sterile 1.5 ml microcentrifuge tube. Tubes were then incubated at room temperature (RT) for 5 min for equilibration. Opti‐MEM^®^ solution containing siRNA was then added dropwise onto the tube containing the lipofectamine RNAi MAX solution and incubated for 20 min at RT to allow liposome formation. The solution was then added dropwise onto the 6‐well and incubated for 6 h. After 6 h media was refreshed, and cells were analyzed 72 h post‐transfection. siRNAs used in this study are listed below:

**Table:** 

siRNA	Reference	Sequence (target)	Company
siControl (Ctr)	1027310	AATTCTCCGAACGTGTCACGT	Qiagen
siKIF5B SmartPool	L‐008867‐00‐0005	GAACUGGCAUGAUAGAUGA CAACAGACAUGUAGCAGUU GCAGGAACGUCUAAGAGUA CAAUUGGAGUUAUAGGAAA	Dharmacon
sip150glued (DCNT1) SmartPool	L‐012874‐00‐0005	CUGGAGCGCUGUAUCGUAA GAAGAUCGAGAGACAGUUA GCUCAUGCCUCGUCUCAUU CGAGCUCACUACUGACUUA	Dharmacon
siαTAT1 #5	SI03124660	AACCGCCATGTTGTTTATATT	Qiagen
siαTAT1 #9	SI04145162	ACCGCACCAACTGGCAATTGA	Qiagen

### RNA extraction, quantification, and cDNA generation

Total RNA extraction was carried out using RNeasy kit (Qiagen) according to the manufacturer's instructions. Eluted RNA was then stored at −80°C. RNA concentration was determined using Nanodrop 1000 spectrophotometer (Thermo Fisher, USA). For cDNA generation, 500 ng of total RNA was mixed with 2 μl of random primer mix (New England Biolabs, UK) in RNase‐free PCR strips (Thermo Fisher, USA). RNase‐free water was added to a final volume of 16 μl. The mixture was heated for 3 min at 65°C in a PCR machine. Tubes were then placed on ice immediately for few minutes. After that, 2 μl of RT buffer, 1 μl of RNase inhibitor (New England Biolabs), and 1 μl of reverse transcriptase was added on top of 16 μl of the extracted RNA. Tubes were incubated at 42°C for 1 h for reverse transcriptase elongation and at 90°C for 15 min for reverse transcriptase inactivation to create a pool of cDNA. cDNA was then stored at −20°C.

### qRT–PCR

A PCR cocktail was generated by adding 9 μl of nuclease‐free water (Thermo Fisher), 30 μl of 2× Power SYBR^®^ Green PCR Master Mix (Thermo Fisher), and 3 μl of gene‐specific forward and reverse primers at 10 μM to generate a final volume of 45 μl. cDNA was diluted by adding 4.5 μl of nuclease‐free water onto 0.5 μl of cDNA to make a final volume of 5 μl. Fifteen microliter of the PCR cocktail was added onto each well in triplicate in a 96‐well plate and 5 μl of the diluted cDNA was then added to all wells in triplicate, giving a final volume of 20 μl in each well. The 96‐well plate was then sealed and centrifuged for few seconds to spin down the mixture. The Ct values acquired from the qRT–PCR reaction were analyzed by using the comparative Ct method (2‐ΔΔCt) and GAPDH was used as a housekeeping gene for normalization.

**Table:** 

Primer	Sequence
aTAT1_forward	GGCGAGAACTCTTCCAGTAT
aTAT1_reverse	TTGTTCACCTGTGGGACT
GAPDH_forward	ACAGTTGCCATGTAGACC
GAPDH_reverse	TTTTTGGTTGAGCACAGG

### Indirect immunofluorescence

1–1.5 × 10^4^ cells were seeded in a final volume of 50–80 μl of serum‐free media on an 18‐mm diameter glass coverslip in a 12‐well plate. The plate was then incubated at 37°C for 30 min to allow cell attachment. Once cells were attached, 1 ml of the growth medium was added and the plate was incubated at 37°C overnight. On the following day, growth medium was aspirated and coverslips were washed with PBS once and fixed immediately with either 4% PFA + PBS at RT for 15 min or with 99.9% ice‐cold methanol at −20°C for 10 min. After fixation, all steps were carried out at RT. Cells were incubated with permeabilization buffer (PBS +0.2% Triton X‐100) for 5 min. After permeabilization, cells were blocked with 1 ml of blocking buffer (PBS, 5% BSA, and 0.1% Triton X‐100) for 30 min. Thirty microliter of the diluted primary antibodies were added onto the coverslip and incubated for 30 min, after which another 30 μl was added for another 30 min to avoid coverslips to dry. Next, coverslips are washed twice with PBS, and secondary antibodies (Alexa Fluor conjugated; Molecular Probes) incubation was performed in the same way as the primary antibodies in the dark. Hoechst 33342 solution was used at 1:10,000 dilution to stain DNA in the dark. Coverslips were then mounted on a drop of ProLong Gold antifade reagent on a microscope slide. Primary and secondary antibodies and molecular probes used in this paper are listed below:

**Table:** 

Antibody (Clone)	Species	Catalog number	Manufacturer	RRID	Dilution	Fixation
Vimentin	Rabbit	5741	Cell Signaling	AB_10695459	1:200	Methanol (MeOH)
EEA1	Rabbit	2411S	Cell Signaling	AB_2096814	1:100	Paraformaldehyde (PFA)
Pericentrin	Rabbit	Ab4448	Abcam	AB_304461	1:1,500	MeOH
α‐tubulin (DM1A)	Mouse	T9026	Sigma Aldrich	AB_477593	1:1,000	MeOH
α‐tubulin FITC‐conjugated	Mouse	F2168	Sigma Aldrich	B_476967	1:200	MeOH
Acetylated tubulin (6‐11B‐1)	Mouse	T6793	Sigma Aldrich	AB_477585	1:2,500	MeOH
GM130	Mouse	610822	BD biosciences	AB_398141	1:100	MeOH/PFA
Centrin2N‐17‐R	Rabbit	Sc‐27793‐R	Santa Cruz	AB_2082359	1:100	MeOH
Anti‐Mouse Alexa Fluor 488	Goat	A11001	Thermo Fisher Scientific	AB_2534069	1:1,000	
Anti‐Mouse Alexa Fluor 647	Goat	A21235	Thermo Fisher Scientific	AB_2535804	1:1,000	
Anti‐Rabbit Alexa Fluor 488	Goat	A11008	Thermo Fisher Scientific	AB_143165	1:1,000	
Anti‐Mouse Alexa Fluor 568	Goat	A11004	Thermo Fisher Scientific	AB_2534072	1:1,000	

Probe/dye	Catalog number	Manufacturer	Dilution	Fixation
Alexa Fluor 568 Phalloidin	A12380	Thermo Fisher Scientific	1:500	PFA
Mitotracker	M7510	Thermo Fisher Scientific	1:10,000	PFA

### Western blotting

Cells were collected and resuspended in 100 μl of RIPA buffer (Thermo Fisher Scientific) with added protease inhibitors (Roche; 1 tablet/10 ml RIPA). Protein concentration was quantified using the Bio‐Rad DC protein assay and 15 μg of protein was loaded per well. Protein samples were resuspended in Laemmli buffer and separated on SDS–PAGE and transferred onto PVDF membranes. Western blots were developed using SRX‐101A Konica Minolta and scanned. Antibodies used for Western blot analyses are listed below.

**Table:** 

Antibody	Species	Catalog number	Manufacturer	RRID	Dilution
KIF5B	Mouse	Ab167429	Abcam	AB_2715530	1:1,000
p150^glued^/dynactin	Mouse	610474	BD Bioscience	AB_397846	1:1,000
β‐Actin	Rabbit	4970	Cell Signaling	AB_2223172	1:5,000
α‐tubulin (DM1A)	Mouse	T9026	Sigma Aldrich	AB_477593	1:3,000
Acetylated tubulin	Mouse	T6793	Sigma Aldrich	AB_477585	1:3,000
HRP anti‐rabbit secondary	Polyclonal	NA934	GE healthcare Lifesciences	AB_772206	1:1,000
HRP anti‐mouse secondary	Polyclonal	NA931	GE healthcare Lifesciences	AB_772210	1:1,000

### 3D collagen gels

Collagen gels were performed as previously described (Infante *et al*, 2018). Briefly, glass coverslips were layered with 15 μl of a 2.2 mg/ml type‐I collagen solution (bottom layer). Polymerization was induced at 37°C for 3 min. Then, a cell suspension (1.5–2.5 × 10^5^ cells/ml) was added to the bottom layer and cultures were incubated for 30 min at 37°C to allow cells to adhere to the collagen gels. Growth medium was gently removed and a 2.2 mg/ml type‐I collagen solution was polymerized on top of the cells (top layer). After polymerization at 37°C for 90 min, growth medium was added to the cultures. Z‐stacks of images were acquired with an inverted Nikon microscope coupled with a spinning disk confocal head (Andor) with a 60× objective.

### Quantifications of indirect immunofluorescence images

For centrosome number quantification, cells were stained with the DNA dye Hoechst and centrin2 (centriole marker) and a number of centrosomes were quantified in mitosis: 4 centrioles = 2 centrosomes (normal) and > 5 centrioles = amplified centrosomes. Centrosome‐nucleus distance was manually assessed using Fiji by drawing a line between nucleus edge and centrosome(s) center. Cells where centrosomes were located on top of the nucleus were excluded from this analysis. For nucleus aspect ratio quantification, cells were stained with Hoechst (DNA) and Fiji was used to assess the width and height of the nucleus. We subtracted the lowest value by the highest, since we were not assessing nucleus orientation, to ensure the maximum value obtained was 1 (ratio 1 = perfect circle). Cells containing micronuclei or extra nuclei, although only a small fraction, were excluded from this analysis. To quantify endosomes‐nucleus distance, cells were stained for EEA1 and DNA. Individual endosome distance to the nucleus was manually determined using Fiji “find maxima plugin” to determine endosome coordinates. Mean endosomes distance per cell was then calculated as the average of all endosomes for each cell. For the quantification of vimentin displacement, cells were stained for vimentin and microtubules and vimentin fluorescence intensity was determined as previously described (Leduc & Etienne‐Manneville, 2017). Briefly, vimentin fluorescence intensity was calculated as the ratio between fluorescence intensity at the leading edge (20 μm from the plasma membrane) and in a 15‐μm radius perinuclear region (ratio < 1 = vimentin associated with the nucleus; ratio > 1 = vimentin displaced towards cell leading edge). For Golgi area quantification, cells were stained for GM130 and DNA. Golgi area was manually determined using Fiji “freehand draw” and “measure” options. For mitochondria area quantification, cells were stained with MitoTracker (in order to visualize mitochondria) and with phalloidin (in order to visualize F‐actin and determine cell area and border). Mitochondria area was manually determined and mitochondria spreading was determined as the ratio between mitochondria area over total cell area. For all imaging experiments, cells were imaged with an Eclipse Ti‐E inverted microscope (Nikon) equipped with a CSU‐X1 Zyla 4.2 camera (Ti‐E, Zyla; Andor), including a Yokogawa Spinning Disk, a precision motorized stage, and Nikon Perfect Focus, all controlled by NIS‐Elements Software (Nikon). 60× 1.45‐NA oil objective was used to acquire images. Images used for all the analyses were not blinded, but all images were acquired using the DNA dye channel as reference to exclude any bias.

### Acetylated and total microtubule orientation variations

Images for −DOX, +DOX, H_2_O_2_, Tubacin, +DOX + Tubacin, and αTAT1 overexpression (αTAT1 OE) were collected using an Eclipse Ti‐E inverted microscope (Nikon) equipped with a CSU‐X1 Zyla 4.2 camera (Ti‐E, Zyla; Andor). Images of individual cells were transformed in Fiji to have all cells aligned horizontally, with the leading edge on the right and cell rear on the left. Images were thresholded for both acetylated tubulin and total α‐tubulin to remove background fluorescence. The orientations of acetylated microtubules were calculated using the OrientationJ plugin for Fiji (Rezakhaniha *et al*, 2012; Puspoki *et al*, 2016) for the front of the cell (as defined by the centrosome to the leading edge) and the cell rear (defined as the centrosome to the rear of the cell). These same regions were used to quantify the distribution of orientations for α‐tubulin. The front and rear frequencies were summed to calculate the total frequency of all orientations and used to normalize the data for each cell. The difference between front and rear orientation frequency was calculated by subtracting the rear frequency from the front frequency for each orientation.

### Quantification of microtubule polarity by live‐cell imaging

To examine microtubule polarity, RPE‐1 cells expressing EB3‐GFP (−DOX, +DOX, H_2_O_2_, Tubacin) or EB3‐tdTomato (αTAT1 OE) were seeded onto 8‐well glass bottom chambers (iBidi) overnight at low confluency (3 × 10^4^ per well). The following day, H_2_O_2_ and Tubacin conditions were treated as previously described before live imaging using an Eclipse Ti‐E inverted microscope (Nikon) equipped with a CSU‐X1 Zyla 4.2 camera (Ti‐E, Zyla; Andor), including a Yokogawa Spinning Disk, a precision motorized stage, and Nikon Perfect Focus, all controlled by NIS‐Elements Software (Nikon). The microscope was enclosed within temperature‐ and CO_2_‐controlled environments that maintained an atmosphere of 37°C and 5% humidified CO_2_ for live‐cell imaging. 60× 1.45‐NA oil objective was used to capture images every second for 30 s. EB3‐positive comets were tracked using the TrackMate plugin for Fiji (Tinevez *et al*, 2017), using automatic detection and filtering for tracks longer than 7 s to calculate the total number of EB3 comets. Identification of reverse polarity microtubules, defined as an EB3 comet traveling towards the center of the cell, was then performed manually. Temporal projections were made using the “Temporal‐Color Code” feature in Fiji and colored using the Turbo LUT.

### Reactive oxygen species (ROS) quantification by live‐cell imaging

To measure ROS levels in live cells, 4 × 10^4^ cells (−DOX and H_2_O_2_) and 5 × 10^4^ cells (+DOX and +DOX + Apocynin) were seeded overnight in 8‐well glass bottom chambers (iBidi). On the following day, cells were washed with 1× PBS twice and incubated for 20 min in dark at 37°C with 20 μM of carboxy‐H_2_DCFDA (2′,7′‐dichlorodihydrofluorescein diacetate; I36007, Thermo Fisher) diluted in serum‐free medium. After incubation with carboxy‐H_2_DCFDA, cells were incubated for 5 min in the dark at 37°C with Hoechst 33342 diluted 1:10,000 in full growth medium. Cells were then washed with 1× PBS twice and 300 μl of growth medium was added per well. Carboxy‐H_2_DCFDA gets hydrolyzed inside cells to form a nonfluorescent compound, which can be oxidized in the presence of ROS to DCF, which is fluorescence. Cells incubated with carboxy‐H_2_DCFDA were immediately imaged on an Eclipse Ti‐E inverted microscope (Nikon) as described above. 60× 1.45‐NA oil objective was used to capture images at multiple fields (~15 fields) and z‐stack images were captured with 0.5‐μm step size and the step size was calculated to minimal pixel overlapping between steps. This procedure was repeated for each condition. “nd” files containing z‐stack images were directly opened in the Fiji software. SUM projection was applied to obtain a 2D image and fluorescence intensity was quantified per cell per field. Raw integrated density of multiple cells was measured. To obtain the mean total fluorescence intensity per cell in a field, the total fluorescence intensity was divided by the total number of cells per field. Five to ten fields were analyzed to have a total number of ~30 cells per condition for each experiment.

### Quantification of acetylated tubulin

“nd” files containing z‐stack images were directly opened in the Fiji software. SUM projection was applied to obtain a 2D image. To quantify the total fluorescence intensity of single cells, the boundaries of single cells within an image were outlined using the “freehand” selection tool in the Fiji software. By using the “measure” command, raw integrated density and area of a single cell were measured. After that, a region without fluorescence outside the cell (background) was outlined and measured to obtain mean background fluorescence. Background‐corrected total fluorescence intensity of a single cell was determined using the formula = Raw integrated density − (Area of selected cells × Mean fluorescence of background reading).

To quantify the distribution of total and acetylated tubulin throughout the cell in 2D cultures, SUM projection images of individual cells were transformed in Fiji to have all cells aligned horizontally, with the leading edge on the right and cell rear on the left. Images were then thresholded for DNA (Hoechst), acetylated tubulin, and total α‐tubulin to remove background fluorescence, with background values set to “Not a Number” (NaN). A ROI was then drawn over the entire cell and the “Plot Profile” feature of Fiji was used to calculate the signal intensity over distance for all channels. The nucleus center was calculated as the mid‐point value in the DNA profile. The cell was divided into thirds based on the distance between the nucleus center and leading edge. Intensity values for each range were summed to give the fluorescence intensity for both total and acetylated tubulin in each region.

### Quantification of nocodazole‐resistant microtubules

To quantify the number of microtubules that resist nocodazole treatment we followed a previously published protocol (Xu *et al*, 2017). Briefly, cells were plated on glass coverslips overnight. The following day cells were treated with 2 μM of nocodazole in growth medium at 37°C for 30 min. Coverslips were then washed in the extraction buffer (60 mM PIPES, 25 mM HEPES, 2 mM MgCl_2_, 10 mM EGTA, pH 7.0) by rinsing quickly. To extract soluble tubulin, coverslips were immersed in the same extraction buffer containing 0.2% Triton X‐100 and 2 μM of nocodazole for 1 min at room temperature. Cells were quickly fixed in cold methanol at −20°C for 10 min. Next, normal immunofluorescence protocol to stain for microtubules, acetylated tubulin, and DNA was applied. Cells were imaged using an inverted Zeiss LS880 confocal and a 60× objective. A number of microtubules that emanate from the centrosomes were quantified manually in Fiji. Total fluorescence intensity of α‐tubulin was also quantified in 2D images obtained using SUM projection. The boundaries of single cells within an image were outlined using the “freehand” selection tool in the Fiji software. By using the “measure” command, raw integrated density of a single cell was measured.

### Transwell migration assay

RPE‐1 cells stably expressing H2B‐GFP were grown on transwell chambers (iBidi). Briefly, the bottom of the upper chamber is a cell‐permeable membrane with 5‐μm or 8‐μm diameter pore size holes allowing cells to migrate through the chamber. Cell‐permeable membrane was coated on their external side, where cells attach, with 20 μg/ml fibronectin and 10 μg/ml fluorescent conjugated fibronectin solution. 2.5 × 10^4^ cells (siCtrl‐DOX, siCtrl+DOX, siαTAT1 + DOX, and siCtrl + H_2_O_2_) were seeded in the upper chamber in serum‐free medium or with 75 μM of H_2_O_2_ in serum‐free medium for siCtrl+H_2_O_2_‐treated cells. Serum‐containing medium was added to the bottom wells to function as an attractant to cells and allow efficient cell migration through the pores. Transwells were imaged for 12–16 h on an Eclipse Ti‐E inverted microscope (Nikon) equipped with a CSU‐X1 Zyla 4.2 camera (Ti‐E, Zyla; Andor), including a Yokogawa Spinning Disk, a precision motorized stage, and Nikon Perfect Focus, all controlled by NIS‐Elements Software (Nikon). The microscope was enclosed within temperature‐ and CO_2_‐controlled environments that maintained an atmosphere of 37°C and 5% humidified CO_2_ for live‐cell imaging. Movies were acquired with a Plan Fluor 10× dry objective with a 15.2‐mm working distance. Time crossing the pores (speed of nuclear translocation) was determined as the period since a nucleus reaches a pore until it completely crosses the membrane (phase 2 in the scheme in Fig 8G).

### Statistical analysis

Graphs and statistics were generated using Prism 9 (GraphPad Software) where results are presented as mean ± standard deviation (SD) unless otherwise stated. Statistical analysis was performed on average values for each experiment using one‐way ANOVA with a Tukey's *post hoc* test, unpaired *t‐*test, one sample *t*‐test for normalized data (using a hypothetical mean of 1), and two‐way ANOVA with Sidak's multiple test comparison. Before any statistical analyses, normal distribution of the data was assessed using the Shapiro–Wilk normality test in prism. Different tests utilized are highlighted in the figure legends. *P*‐values are indicated in the graphs. ns = not significant (*P* > 0.05).

## Author contributions

**Pedro Monteiro:** Data curation; formal analysis; validation; investigation; visualization; methodology; writing – review and editing. **Bongwhan Yeon:** Data curation; formal analysis; validation; investigation; visualization; methodology; writing – review and editing. **Samuel S Wallis:** Data curation; formal analysis; validation; investigation; visualization; methodology; writing – review and editing. **Susana A Godinho:** Conceptualization; data curation; supervision; funding acquisition; investigation; visualization; writing – original draft; project administration; writing – review and editing.

## Disclosure and competing interests statement

The authors declare that they have no conflict of interest.

## Supporting information



Appendix

Expanded View Figures PDF

Movie EV1

Movie EV2

Source Data for Expanded View

PDF+

Source Data for Figure 1

Source Data for Figure 2

Source Data for Figure 3

Source Data for Figure 4

Source Data for Figure 5

Source Data for Figure 6

Source Data for Figure 7

## Data Availability

This study includes no data deposited in external repositories.
