# Supplementary material for: Centrosome amplification fine tunes tubulin acetylation to differentially control intracellular organization
Source: EMBO J. 2023 Jul 5;42(16):e112812. doi: 10.15252/embj.2022112812 (PMC10425843; doi:10.15252/embj.2022112812)
Supplement: Supplementary file 1 — Appendix [file EMBJ-42-e112812-s004.pdf]

Table of contents:

- Appendix Figure S1

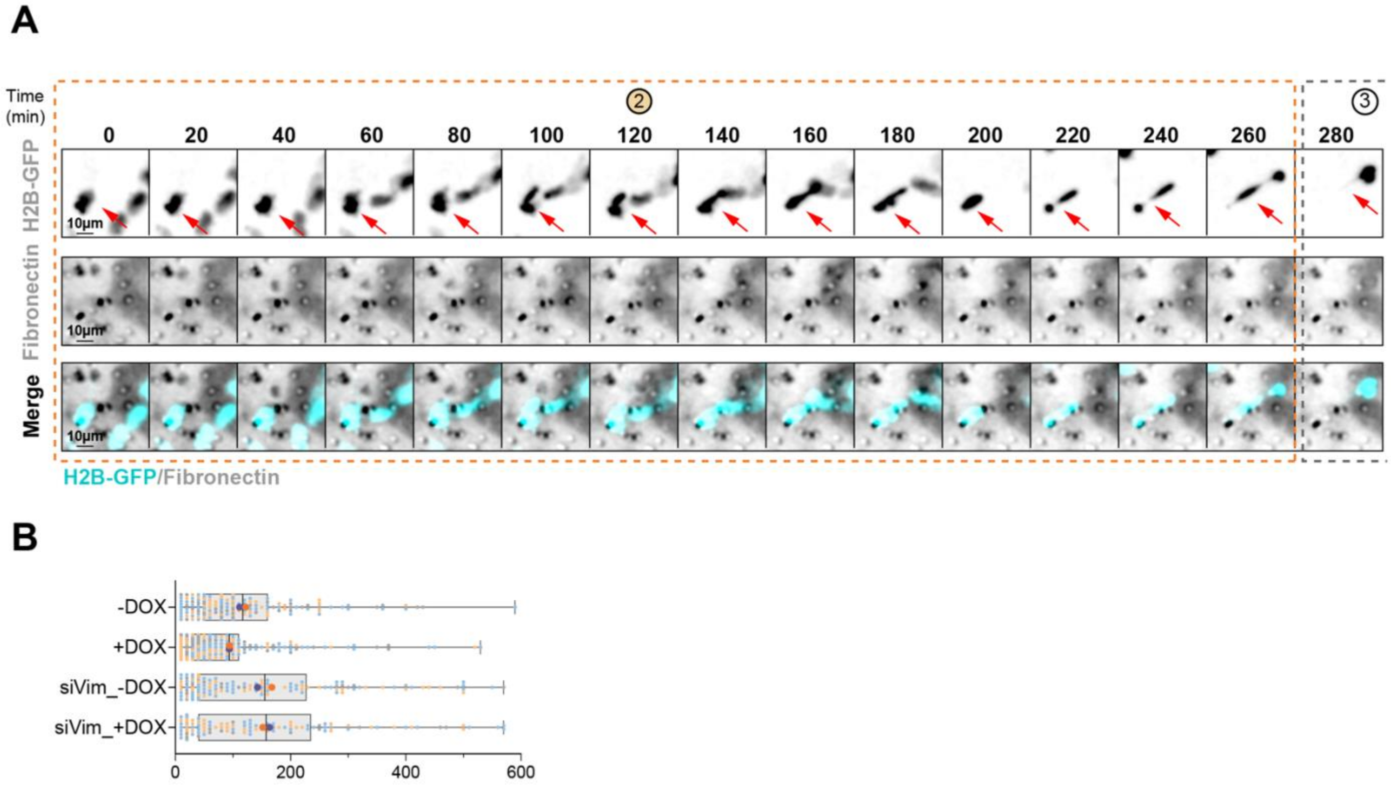

Appendix Figure S1.

A. Time lapse of RPE-1 H2B-GFP cells depleted of vimentin migrating through small pores (5 µm). Red arrow indicates a nucleus crossing a small pore. A highly deformed nucleus can be seen at 220-260 minutes due to vimentin depletion. Scale bar = 10 µm.

B. Quantification of time spent by the nucleus to cross 5 µm-diameter constrictions. $n=_{(-DOX\ siCtr)}$=145; $n=_{(+DOX\ siCtr)}$=160; $n=_{(-DOX\ siVimentin)}$=128; $n=_{(+DOX\ siVimentin)}$=105. Data represent 2 independent experiments. Vertical line represents the median and whiskers the minimum (left quartile) and maximum values (right quartile).

We observed that upon vimentin depletion, nucleus spends in average ~155±17 min (-DOX) and 157±8 min (+DOX) to cross 5 µm constrictions, when compared with control cells (-DOX; 116±7 min) or cells with amplified centrosomes (+DOX; 93±0.2 min). Thus, these data suggests that loss of vimentin leads to higher nuclear deformability in cells migrating through small constrictions that compromises efficient migration.
