# Supplementary material for: Centrosome amplification fine tunes tubulin acetylation to differentially control intracellular organization
Source: EMBO J. 2023 Jul 5;42(16):e112812. doi: 10.15252/embj.2022112812 (PMC10425843; doi:10.15252/embj.2022112812)
Supplement: Supplementary file 2 — Expanded View Figures PDF [file EMBJ-42-e112812-s011.pdf]

Expanded View Figures

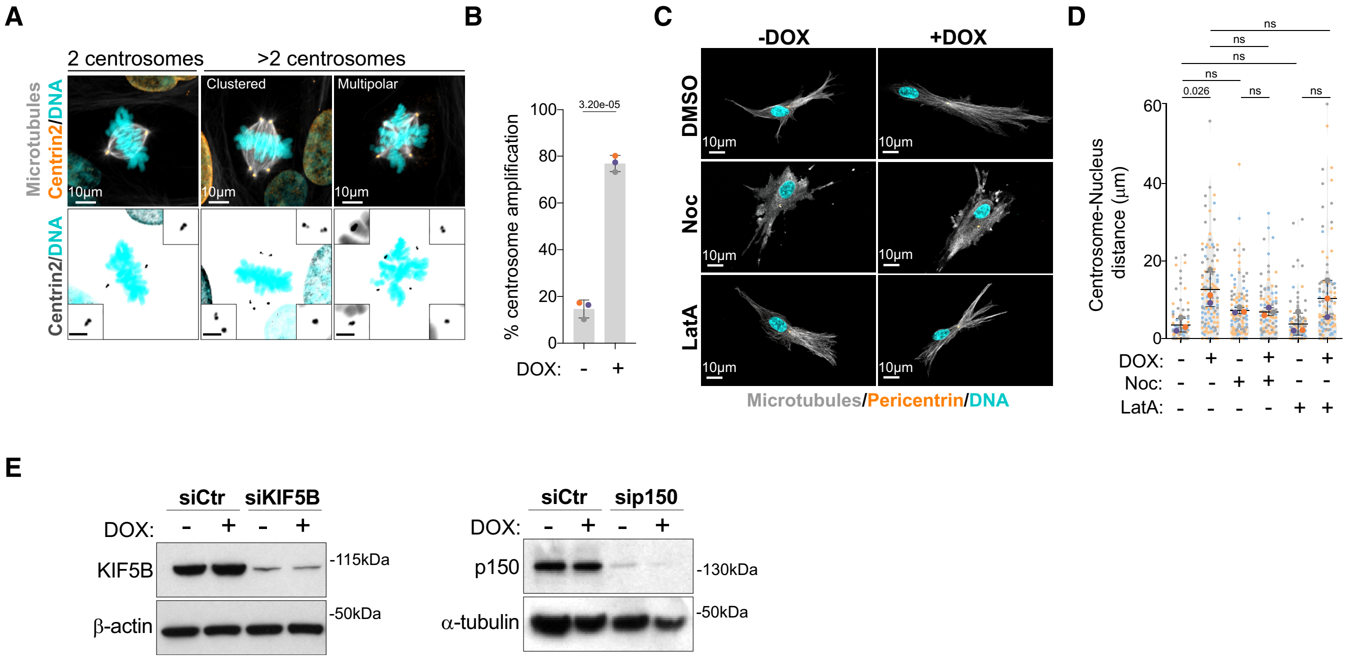

Figure EV1. Increased centrosome displacement in cells with extra centrosomes requires microtubules.

A Representative images of cells stained for centrosomes (Centrin2, orange), microtubules (α-tubulin, gray), and DNA (Hoechst, cyan). Scale bar: 10 µm; inset scale bar: 2 µm.

B Quantification of metaphase cells with extra centrosomes ($n_{(-DOX)} = 337$; $n_{(+DOX)} = 339$).

C Representative images of cells embedded in a 3D collagen matrix and stained for centrosomes (Pericentrin, orange), microtubules (α-tubulin, gray), and DNA (Hoechst, cyan) treated with nocodazole (Noc, 10 µM) or latrunculin-A (LatA, 100 nM). Scale bar: 10 µm.

D Quantification of centrosome-nucleus distance ($n_{(-DOX)} = 90$; $n_{(+DOX)} = 114$; $n_{(-DOX\ Noc)} = 112$; $n_{(+DOX\ Noc)} = 101$; $n_{(-DOX\ LatA)} = 110$; $n_{(+DOX\ LatA)} = 108$).

E Left panel; immunoblot of KIF5B and β-actin in cells after KIF5B siRNA for 48 h. Right panel; immunoblot of p150 and α-tubulin in cells after p150 siRNA for 48 h.

Data information: For all graphs, error bars represent mean ± SD from three independent experiments. *P*-values are described in the graphs, ns = not significant ($P > 0.05$). The following statistics were applied: unpaired *t*-test for graph in (B) and one-way ANOVA with Tukey's *post hoc* test for graph in (D). *n* = number of cells analyzed.
Source data are available online for this figure.

© 2023 The Authors

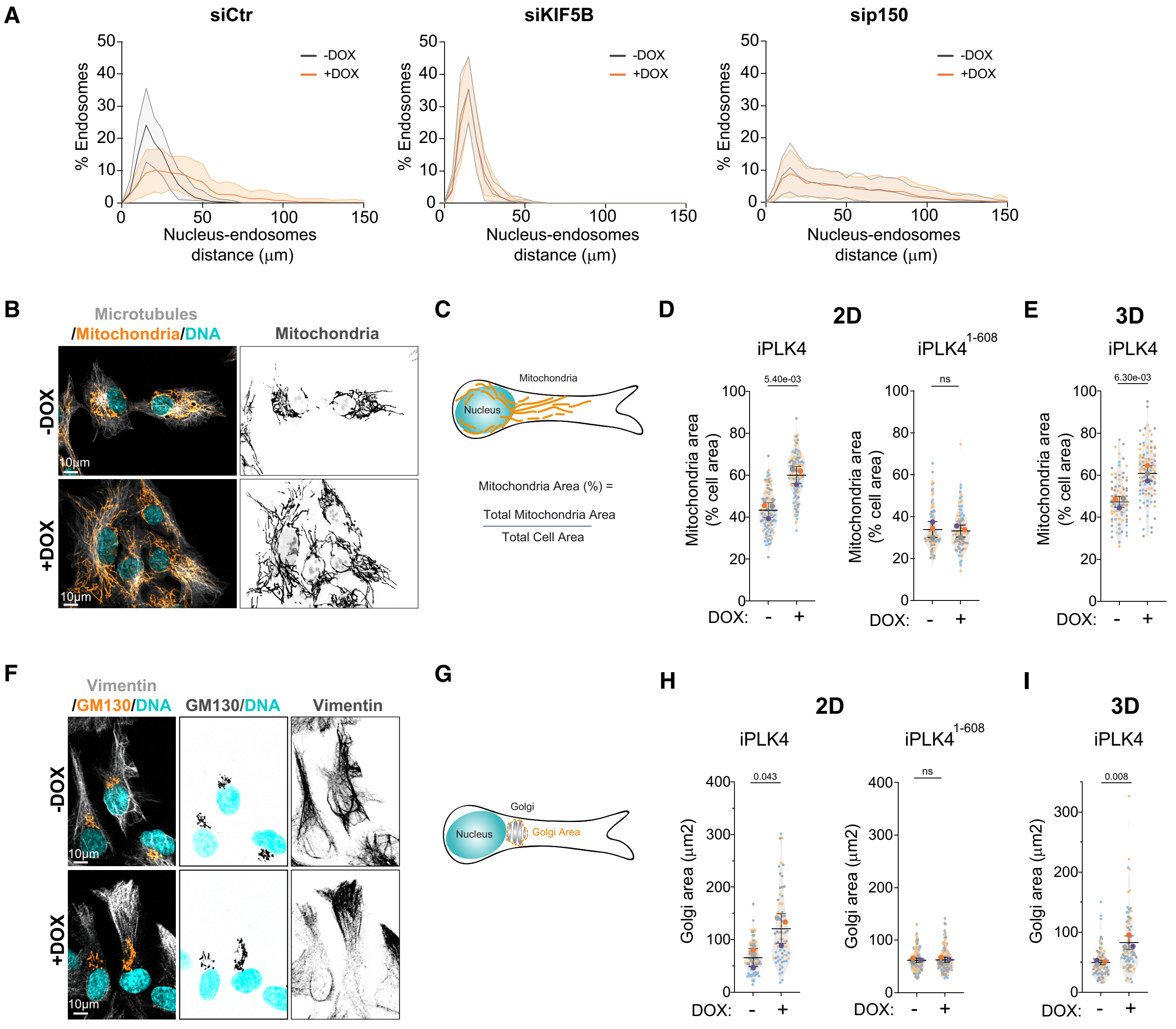

J

	2D			3D			Fold 3D/2D
	-DOX	+DOX	Fold	-DOX	+DOX	Fold	
Centrosomes (distance, μm)	1.3	2.1	**1.7**	4.7	19.4	**4.1**	**2.5**
Endosomes (distance μm)	16.3	21	**1.3**	18.8	36.3	**1.9**	**1.5**
Vimentin (ratio)	1.2	1.4	**1.2**	1	1.3	**1.3**	**1.1**
Golgi (area, μm^2)	65.5	119.8	**1.8**	49.8	82.6	**1.7**	**0.9**
Mitochondira (% total area)	42.8	60.0	**1.4**	48.1	61.4	**1.3**	**0.9**

Figure EV2.

© 2023 The Authors

Figure EV2. Centrosome amplification promotes mitochondria displacement and Golgi dispersion.

A Distribution of endosomes in cells upon depletion of KIF5B and p150 ($n_{(-DOX\ siCtr)}$ = 84; $n_{(+DOX\ siCtr)}$ = 81; $n_{(-DOX\ siKIF5B)}$ = 84; $n_{(+DOX\ siKIF5B)}$ = 83; $n_{(-DOX\ sip150)}$ = 82; $n_{(+DOX\ sip150)}$ = 83).
B Representative images of cells stained for mitochondria (MitoTracker, orange), microtubules (α-tubulin, gray), and DNA (Hoechst, cyan). Scale bar: 10 μm.
C Representative scheme of mitochondria area quantification.
D Quantification of mitochondria area in cells plated in 2D upon induction of PLK4 (Left panel; $n_{(-DOX)}$ = 113; $n_{(+DOX)}$ = 114) or $PLK4^{1\text{-}608}$ overexpression (Right panel; $n_{(-DOX)}$ = 95; $n_{(+DOX)}$ = 98).
E Quantification of mitochondria area in cells plated in 3D ($n_{(-DOX)}$ = 90; $n_{(+DOX)}$ = 102).
F Representative images of cells stained for Golgi (GM130, orange), vimentin (gray) and DNA (Hoechst, cyan). Scale bar: 10 μm.
G Representative scheme of Golgi area quantification.
H Quantification of Golgi area upon induction of PLK4 (Left panel; $n_{(-DOX)}$ = 94; $n_{(+DOX)}$ = 70) or $PLK4^{1\text{-}608}$ overexpression (Right panel; $n_{(-DOX)}$ = 146; $n_{(+DOX)}$ = 133).
I Quantification of Golgi area in cells plated in 3D ($n_{(-DOX)}$ = 91; $n_{(+DOX)}$ = 83).
J Table summarizing the fold change between 2D and 3D conditions for the intracellular compartments analyzed.

Data information: For all graphs, error bars represent mean ± SD from three independent experiments. *P*-values are described in the graphs, ns = not significant ($P > 0.05$). The following statistics were applied: unpaired *t*-test for all graphs. *n* = number of cells analyzed.
Source data are available online for this figure.

 © 2023 The Authors

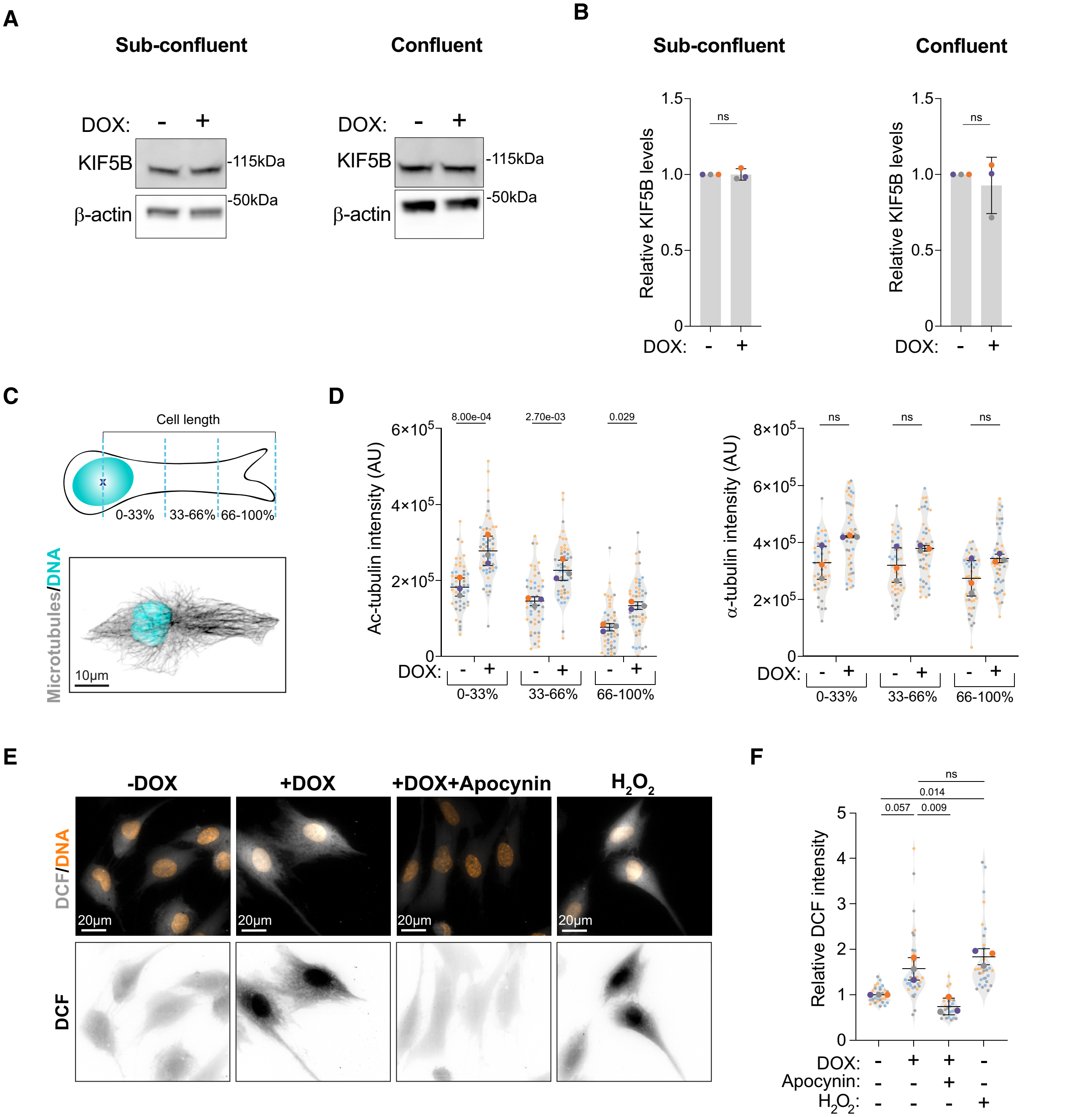
A
Sub-confluent
Confluent
DOX: - +
KIF5B
β-actin
-115kDa
-50kDa
B
Sub-confluent
Confluent
Relative KIF5B levels
ns
DOX: - +
C
Cell length
0-33%
33-66%
66-100%
Microtubules/DNA
10µm
D
8.00e-04
2.70e-03
0.029
Ac-tubulin intensity (AU)
α-tubulin intensity (AU)
ns
DOX: - +
0-33%
33-66%
66-100%
E
-DOX
+DOX
+DOX+Apocynin
H2O2
DCF/DNA
DCF
20µm
F
0.057
0.009
0.014
ns
Relative DCF intensity
DOX:
Apocynin:
H2O2:

Figure EV3.

Figure EV3. Distribution of acetylated microtubules and ROS levels in cells with amplified centrosomes.

A Immunoblots of KIF5B and β-actin in cells without (−DOX) and with amplified centrosomes (+DOX) under sub-confluent (Left panel) and confluent (Right panel) conditions.

B Quantification of KIF5B total levels in cell lysates under sub-confluent (Left panel) and confluent (Right panel) conditions.

C Top panel: Representative scheme of the quantification of intracellular distribution of acetylated tubulin. Bottom panel: Representative image of a control cell stained for microtubules (α-tubulin, gray) and DNA (Hoechst, cyan). Scale bar: 10 μm.

D Left panel: Quantification of intracellular distribution of acetylated tubulin across the length of the cell; Right panel: Quantification of intracellular distribution of total tubulin across the length of the cell ($n_{(-DOX)} = 47$; $n_{(+DOX)} = 52$).

E Representative images of cells stained for DNA (Hoechst, orange) and DCF (gray) treated with Apocynin (0.5 mM) and H_2O_2 (75 μM). Scale bar: 20 μm.

F Quantification of total DCF fluorescence intensity ($n_{(-DOX)} = 30$; $n_{(+DOX)} = 36$; $n_{(+DOX\ Apocynin)} = 27$; $n_{(-DOX\ H2O2)} = 30$).

Data information: For all graphs, error bars represent mean ± SD from three independent experiments. *P*-values are described in the graphs, ns = not significant ($P > 0.05$). The following statistics were applied: unpaired *t*-test for graphs in (B), one sample *t*-test was used for comparisons with normalized −DOX condition (using a hypothetical mean of 1) and unpaired *t*-test to compare +DOX and +DOX + Apocynin conditions for graph in (F) and two-way ANOVA with Sidak's multiple test comparison for graph in (D). *n* = number of cells analyzed.
Source data are available online for this figure.

Figure EV4. Endosome displacement and Golgi dispersion do not rely on tubulin acetylation.

A Quantification of αTAT1 mRNA expression in cells treated with siRNA against αTAT1 (two independent siRNAs; #5 and #9).

B Left panel; immunoblot for α-tubulin and acetylated tubulin (Ac-tub) upon αTAT1 depletion (two independent siRNAs; #5 and #9). Right panel; percentage of acetylated tubulin relative to total α-tubulin.

C Representative images of cells stained for microtubules (α-tubulin; gray), acetylated tubulin (Ac-tubulin, orange) and DNA (Hoechst, cyan) upon αTAT1 depletion. Scale bar: 20 μm.

D Representative images of cells stained for early endosomes (EEA1, orange), F-actin (phalloidin, gray), and DNA (Hoechst, cyan) upon αTAT1 depletion. Scale bar: 10 μm.

E Quantification of endosome-nucleus distance (Left panel: $n_{(-DOX\ siCtr)} = 88$; $n_{(+DOX\ siCtr)} = 90$; $n_{(-DOX\ si\alpha TAT1\#5)} = 91$; $_{(+DOX\ si\alpha TAT1\#5)} = 98$; Right panel: $n_{(-DOX\ siCtr)} = 70$; $n_{(+DOX\ siCtr)} = 64$; $n_{(-DOX\ si\alpha TAT1\#9)} = 69$; $_{(+DOX\ si\alpha TAT1\#9)} = 67$).

F Representative images of cells stained for Golgi (GM130, orange), F-actin (phalloidin, gray), and DNA (Hoechst, cyan) upon αTAT1 depletion. Scale bar: 10 μm.

G Quantification of Golgi area (Left panel: $n_{(-DOX\ siCtr)} = 177$; $n_{(+DOX\ siCtr)} = 147$; $n_{(-DOX\ si\alpha TAT1\#5)} = 172$; $n_{(+DOX\ si\alpha TAT1\#5)} = 156$; Right panel: $n_{(-DOX\ siCtr)} = 103$; $n_{(+DOX\ siCtr)} = 103$; $n_{(-DOX\ si\alpha TAT1\#9)} = 128$; $n_{(+DOX\ si\alpha TAT1\#9)} = 109$).

H Representative images of cells treated with siRNA against αTAT1, stained for microtubules (α-tubulin, cyan), acetylated tubulin (Ac-tubulin, orange) and DNA (Hoechst, gray) upon nocodazole treatment (Noc, 2 μM). Scale bar: 10 μm.

I Quantification of microtubule numbers ($n_{(+DOX\ siCtr+Noc)} = 145$; $n_{(+DOX\ si\alpha TAT1+Noc)} = 159$).

Data information: For all graphs, error bars represent mean ± SD from three independent experiments. *P*-values are described in the graphs, ns = not significant ($P > 0.05$). The following statistics were applied: one-way ANOVA with Tukey's *post hoc* test for graphs in (E) and (G) and unpaired *t*-test for graph in (I). *n* = number of cells analyzed.
Source data are available online for this figure.

 © 2023 The Authors

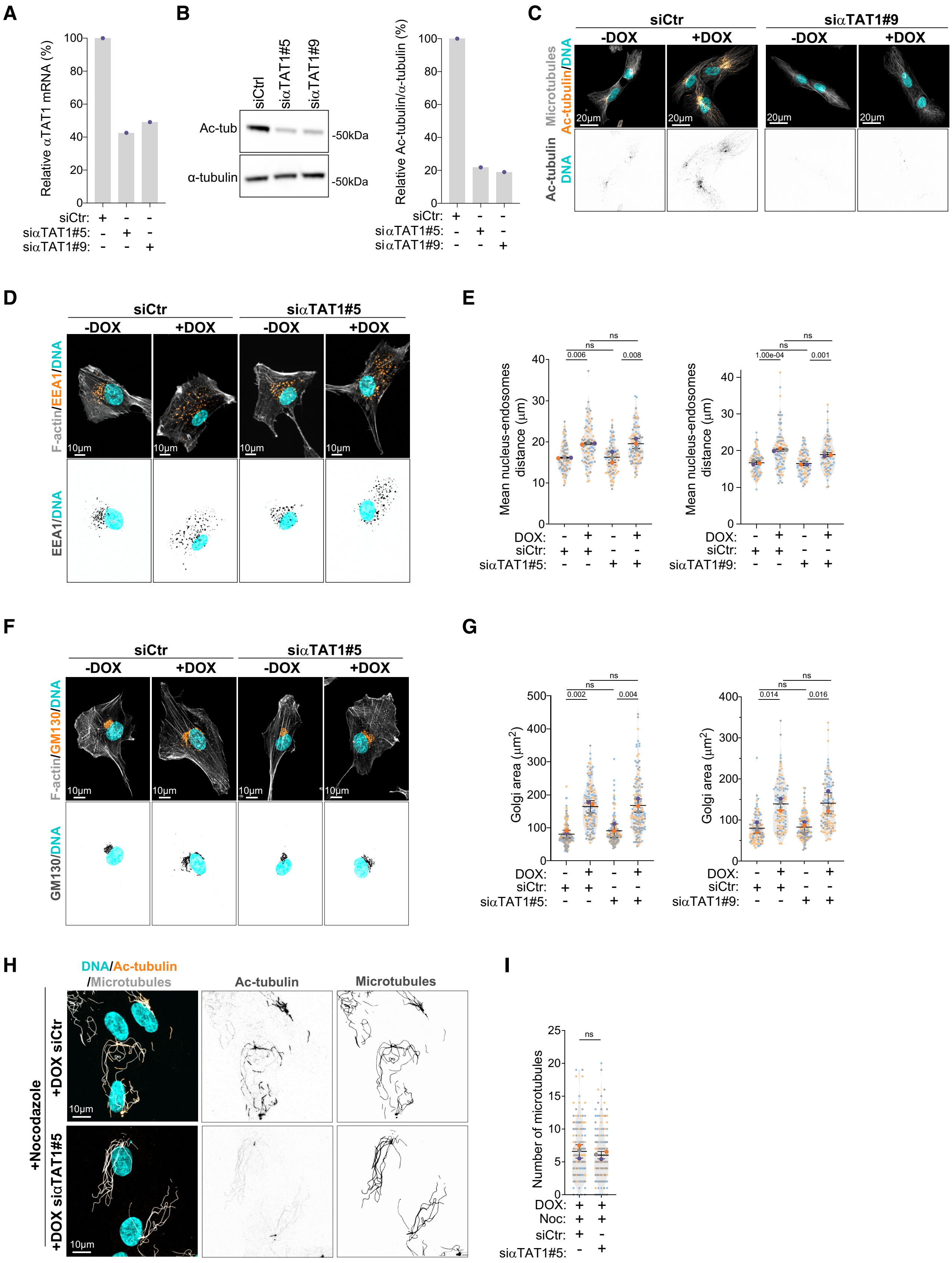
A
Relative αTAT1 mRNA (%)
siCtr:
siαTAT1#5:
siαTAT1#9:
B
siCtrl
siαTAT1#5
siαTAT1#9
Ac-tub
α-tubulin
-50kDa
Relative Ac-tubulin/α-tubulin (%)
C
siCtr
siαTAT1#9
-DOX
+DOX
Microtubules/Ac-tubulin/DNA
Ac-tubulin DNA
20µm
D
siCtr
siαTAT1#5
F-actin/EEA1/DNA
EEA1/DNA
10µm
E
Mean nucleus-endosomes distance (µm)
0.006
0.008
1.00e-04
0.001
ns
DOX:
siCtr:
siαTAT1#5:
siαTAT1#9:
F
F-actin/GM130/DNA
GM130/DNA
G
Golgi area (µm²)
0.002
0.004
0.014
0.016
H
DNA/Ac-tubulin/Microtubules
Ac-tubulin
Microtubules
+Nocodazole
+DOX siCtr
+DOX siαTAT1#5
I
Number of microtubules
Noc:

Figure EV4.

Figure EV5. H_2O_2-treated cells do not display endosome displacement or Golgi dispersion.

A Representative images of cells stained for early endosomes (EEA1, orange), F-actin (phalloidin, gray), and DNA (Hoechst, cyan) treated with H_2O_2. Scale bar: 10 μm.
B Quantification of endosomes-nucleus distance ($n_{(Ctr)} = 82$; $n_{(H2O2)} = 79$).
C Quantification of Golgi area ($n_{(Ctr)} = 151$; $n_{(H2O2)} = 157$).
D Representative images of cells stained for mitochondria (MitoTracker, orange), microtubules (α-tubulin, gray), and DNA (Hoechst, cyan) treated with H_2O_2 (75 μM). Scale bar: 10 μm.
E Representative images of cells stained for acetylated tubulin (Ac-tubulin, orange) and DNA (Hoechst, cyan) treated with Tubacin (5 μM) or overexpressing eGFP-αTAT1 (αTAT1 OE). Scale bar: 10 μm.
F Quantification of acetylated tubulin fluorescence intensity ($n_{(Ctr)} = 29$; $n_{(Tubacin)} = 32$; $n_{(\alpha TAT1\ OE)} = 25$).
G Top: Heat map of α-tubulin distribution in 50 cells from each condition. Bottom: Outline of all cells (based on α-tubulin signal). Cells were superimposed using the center of the nucleus as reference point. Scale bar: 20 μm.
H Table summarizing the effect of different treatments on intracellular organization.

Data information: For all graphs, error bars represent mean ± SD from three independent experiments. *P*-values are described in the graphs, ns = not significant ($P > 0.05$). The following statistics were applied: unpaired *t*-test for graphs in (B) and (C) and one-way ANOVA with Tukey's *post hoc* test for graph in (F). *n* = number of cells analyzed.
Source data are available online for this figure.

 © 2023 The Authors

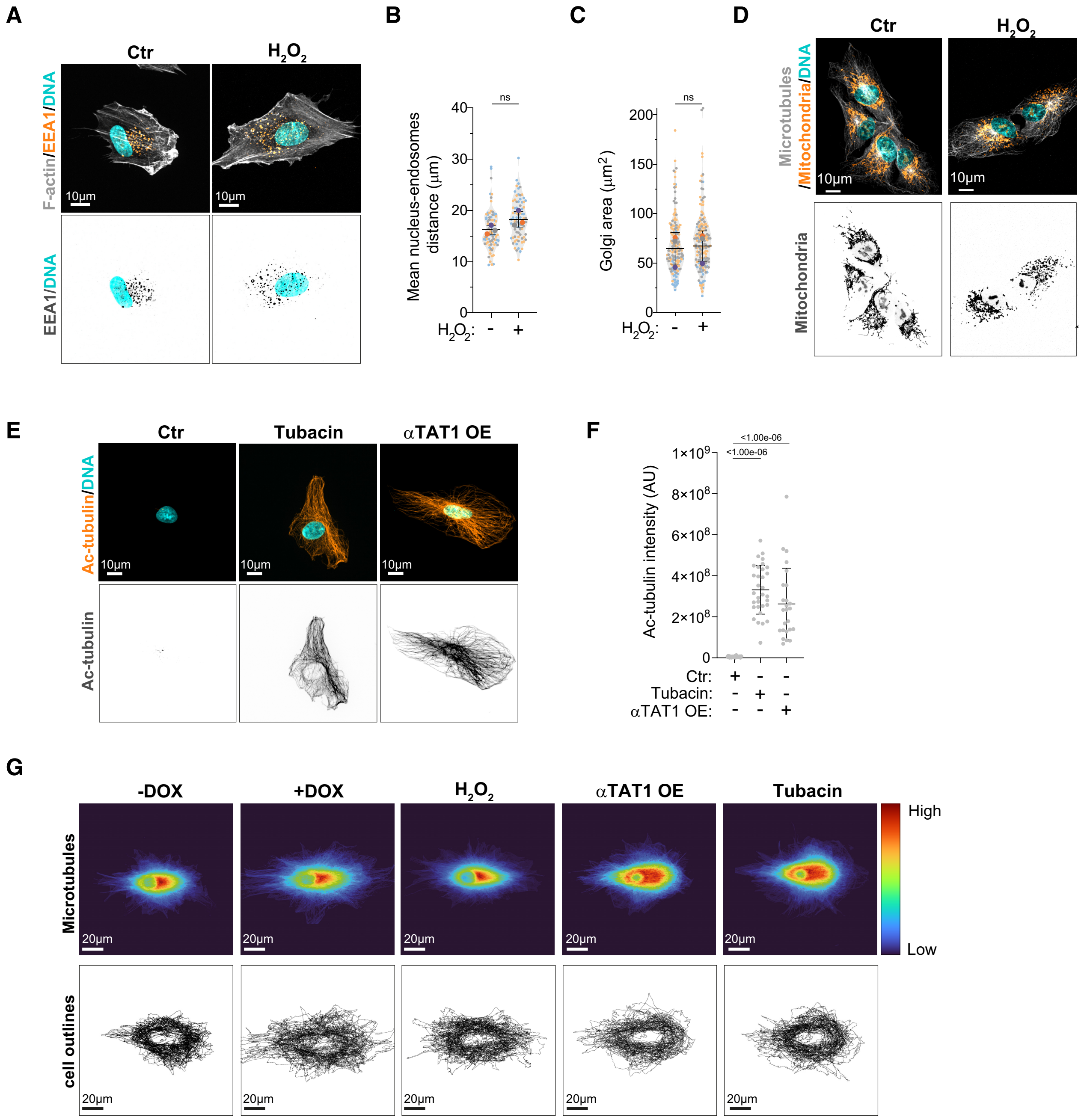

H

	siCtr -DOX	siCtr +DOX	siKIF5B -DOX	siKIF5B +DOX	sip150 -DOX	sip150 +DOX	siαTAT1 -DOX	siαTAT1 +DOX	H_2O_2	Tubacin	αTAT1 OE
**Centrosomes**	–	displaced	*closer to nucleus*	rescued	displaced	displaced	–	rescued	displaced	–	–
**Vimentin**	–	displaced	*closer to nucleus*	rescued	displaced	displaced	–	rescued	displaced	displaced	displaced
**Endosomes**	–	displaced	*closer to nucleus*	rescued	displaced	displaced	–	displaced	–	n.a.	n.a.
**Golgi**	–	displaced	–	n.a.	n.a.	n.a.	–	displaced	–	n.a.	n.a.
**Mitochondria**	–	displaced	–	n.a.	n.a.	n.a.	–	rescued	*fragmented*	displaced	displaced

– *normal positioning*

n.a. *not assessed*

Figure EV5.
