# Supplementary material for: Centrosome amplification fine tunes tubulin acetylation to differentially control intracellular organization
Source: EMBO J. 2023 Jul 5;42(16):e112812. doi: 10.15252/embj.2022112812 (PMC10425843; doi:10.15252/embj.2022112812)
Supplement: Supplementary file 3 — Movie EV1 [file EMBJ-42-e112812-s005.zip › README_Movie EV1.docx]

**Movie EV1 | Time-lapse to track EB3 comets.** Time-lapse imaging using spinning disk confocal microscopy to track EB3-GFP (-DOX, +DOX, H_2_O_2_ and Tubacin treated cells) and EB3-RFP (αTAT1 OE) over 30 seconds. Images were acquired every second. Conditions imaged are as followed: **A.** -DOX, **B.** +DOX, **C.** H_2_O_2_, **D.** Tubacin and **E**. αTAT1 OE. Scale = 10 µm. Note that although we could still identify EB3-comets in αTAT1 OE cells, it also decorates microtubules.
