# Supplementary material for: Centrosome amplification fine tunes tubulin acetylation to differentially control intracellular organization
Source: EMBO J. 2023 Jul 5;42(16):e112812. doi: 10.15252/embj.2022112812 (PMC10425843; doi:10.15252/embj.2022112812)
Supplement: Supplementary file 4 — Movie EV2 [file EMBJ-42-e112812-s009.zip › README_Movie EV2.docx]

**Movie EV2 | Example of reverse polarity microtubules.** Time-lapse imaging using spinning disk confocal microscopy to track EB3-GFP in -DOX cells over 30 seconds. Inset depicts a magnified area of the time-lapse and red arrowheads highlight EB3 comets that do not follow the expected (-) end cell center to (+) end cell periphery polarity. Scale = 10 µm.
