# Supplementary figures and images for: Centrosome amplification fine tunes tubulin acetylation to differentially control intracellular organization
Source: EMBO J. 2023 Jul 5;42(16):e112812. doi: 10.15252/embj.2022112812 (PMC10425843; doi:10.15252/embj.2022112812)

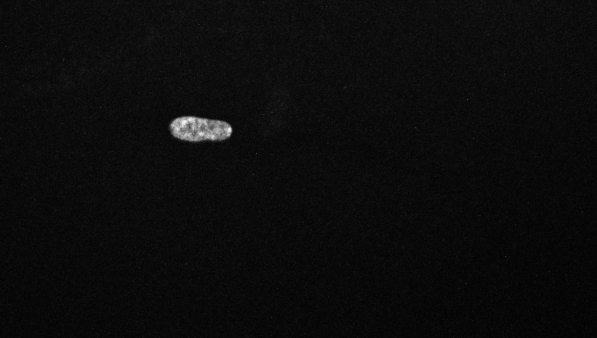

Supplement: Supplementary file 7 — Source Data for Figure 1 [file EMBJ-42-e112812-s006.zip › Figure 1 Source data/Fig 1F image sip150 -DOX.tif]

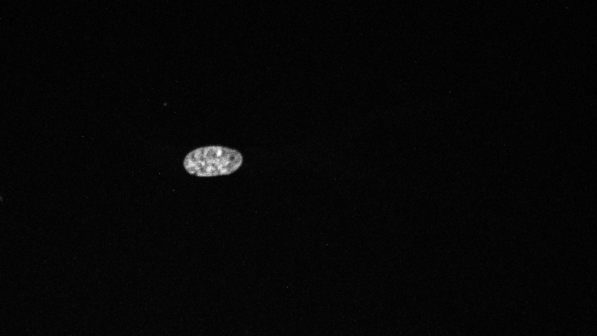

Supplement: Supplementary file 7 — Source Data for Figure 1 [file EMBJ-42-e112812-s006.zip › Figure 1 Source data/Fig 1F image sip150 +DOX.tif]

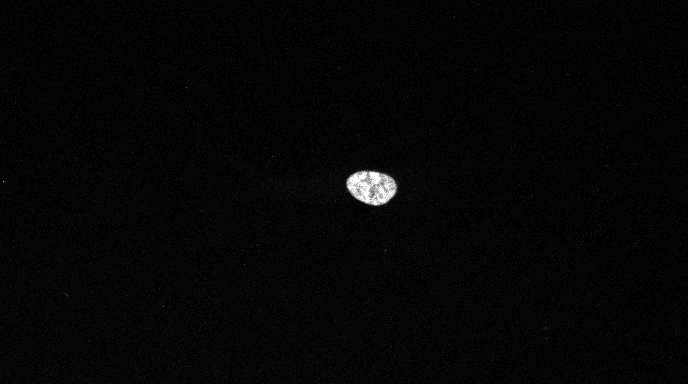

Supplement: Supplementary file 7 — Source Data for Figure 1 [file EMBJ-42-e112812-s006.zip › Figure 1 Source data/Fig 1F image siCtr -DOX.tif]

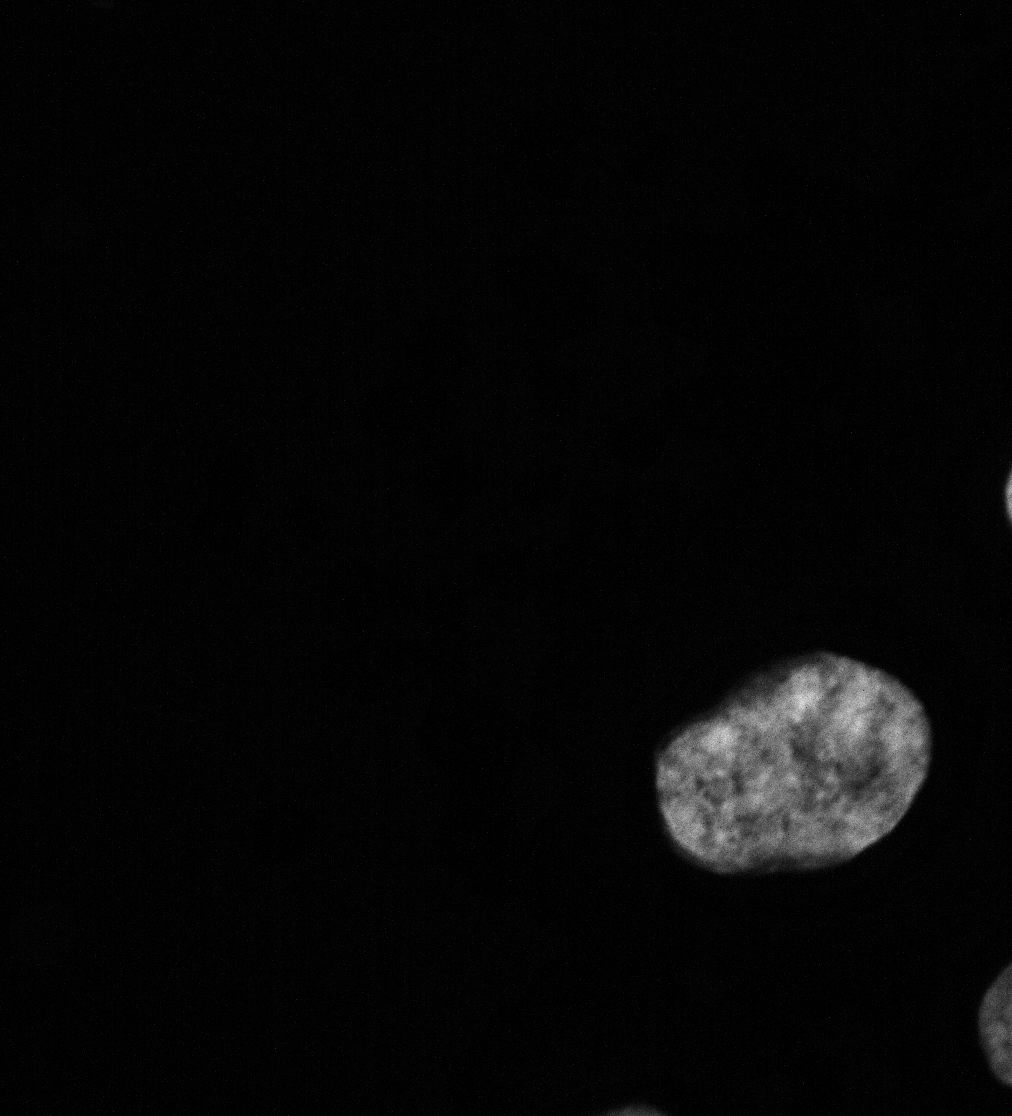

Supplement: Supplementary file 7 — Source Data for Figure 1 [file EMBJ-42-e112812-s006.zip › Figure 1 Source data/Fig 1A image -DOX.tif]

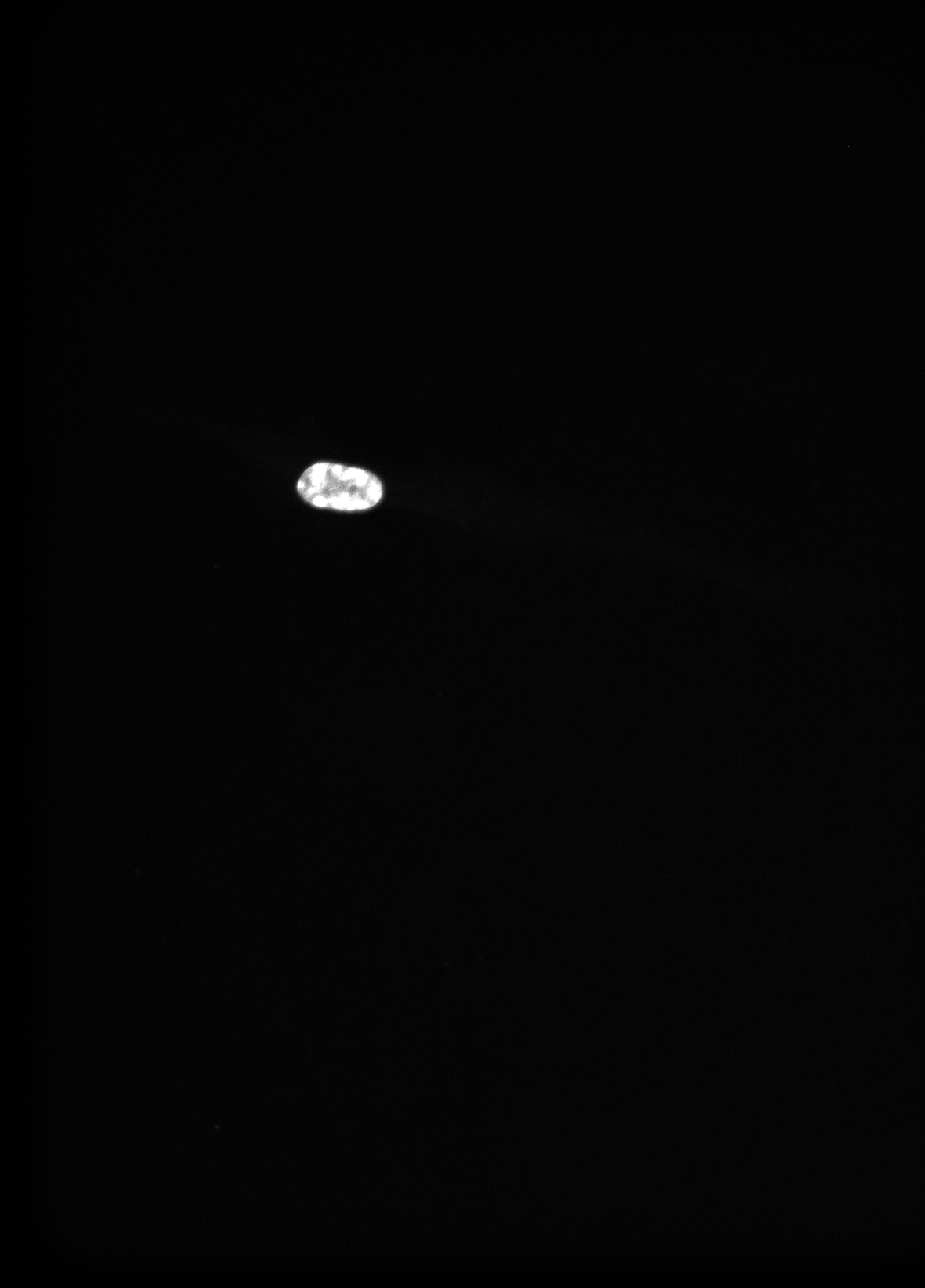

Supplement: Supplementary file 7 — Source Data for Figure 1 [file EMBJ-42-e112812-s006.zip › Figure 1 Source data/Fig 1C image +DOX.tif]

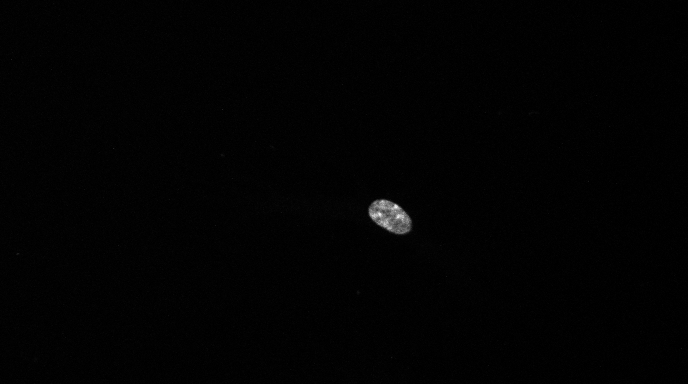

Supplement: Supplementary file 7 — Source Data for Figure 1 [file EMBJ-42-e112812-s006.zip › Figure 1 Source data/Fig 1F image siKIF5B -DOX.tif]

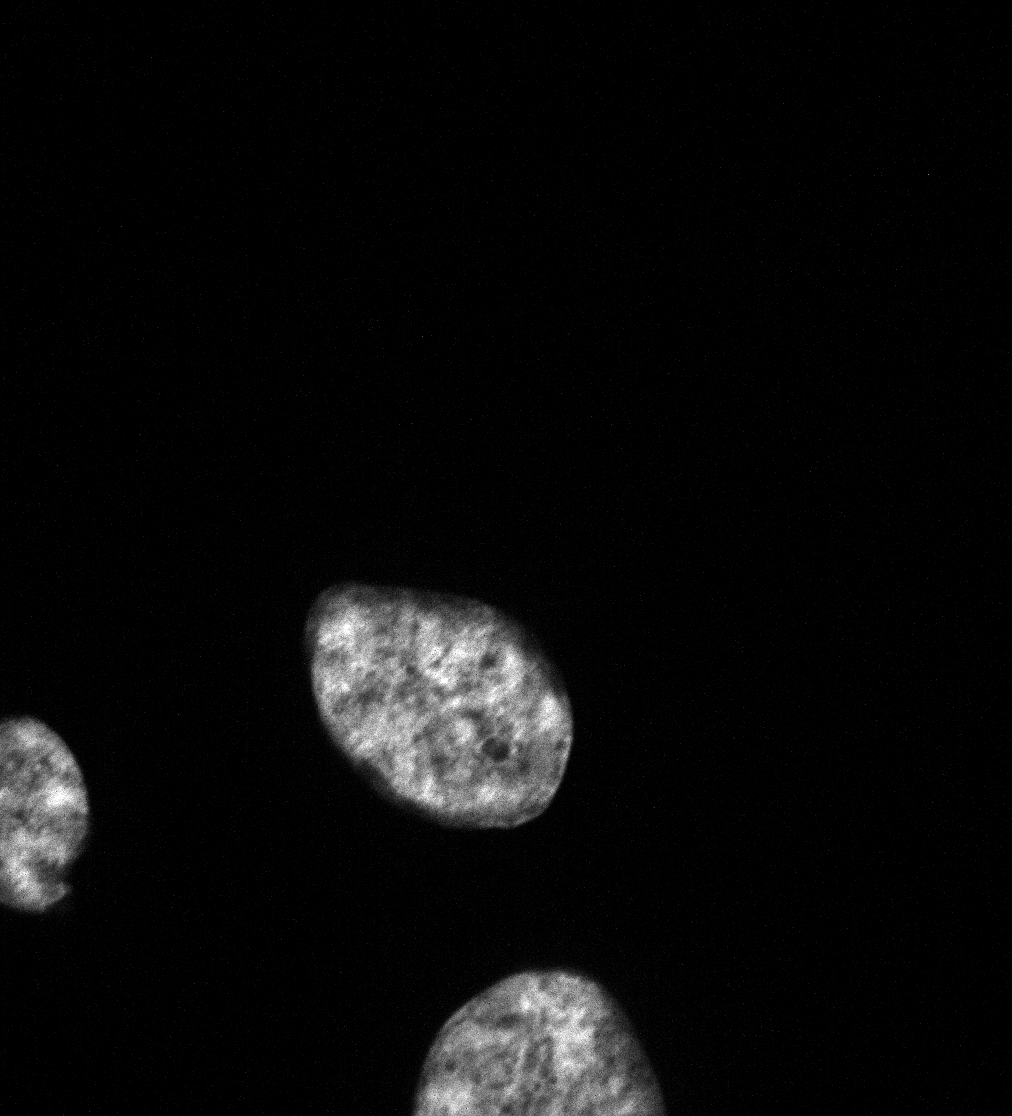

Supplement: Supplementary file 7 — Source Data for Figure 1 [file EMBJ-42-e112812-s006.zip › Figure 1 Source data/Fig 1A image +DOX.tif]

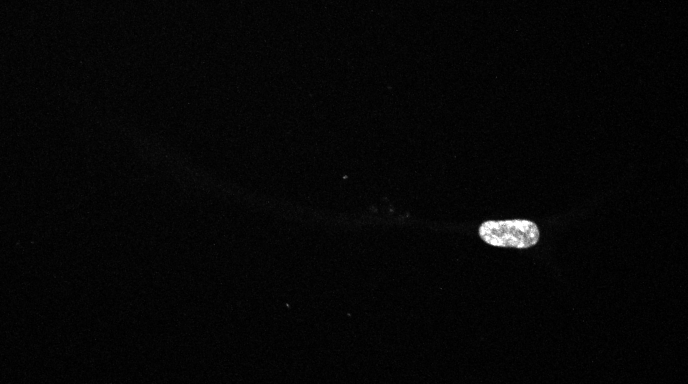

Supplement: Supplementary file 7 — Source Data for Figure 1 [file EMBJ-42-e112812-s006.zip › Figure 1 Source data/Fig 1F image siCtr +DOX.tif]

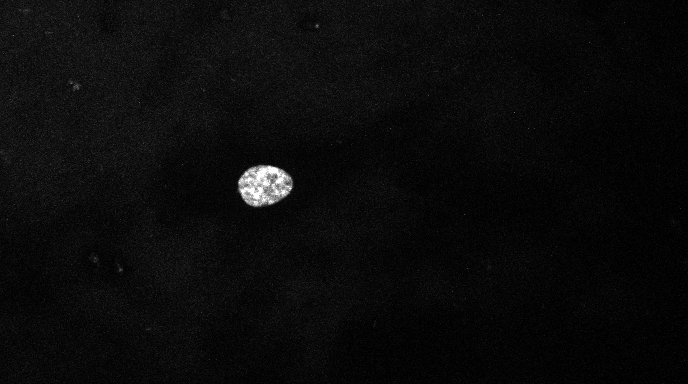

Supplement: Supplementary file 7 — Source Data for Figure 1 [file EMBJ-42-e112812-s006.zip › Figure 1 Source data/Fig 1F image siKIF5B +DOX.tif]

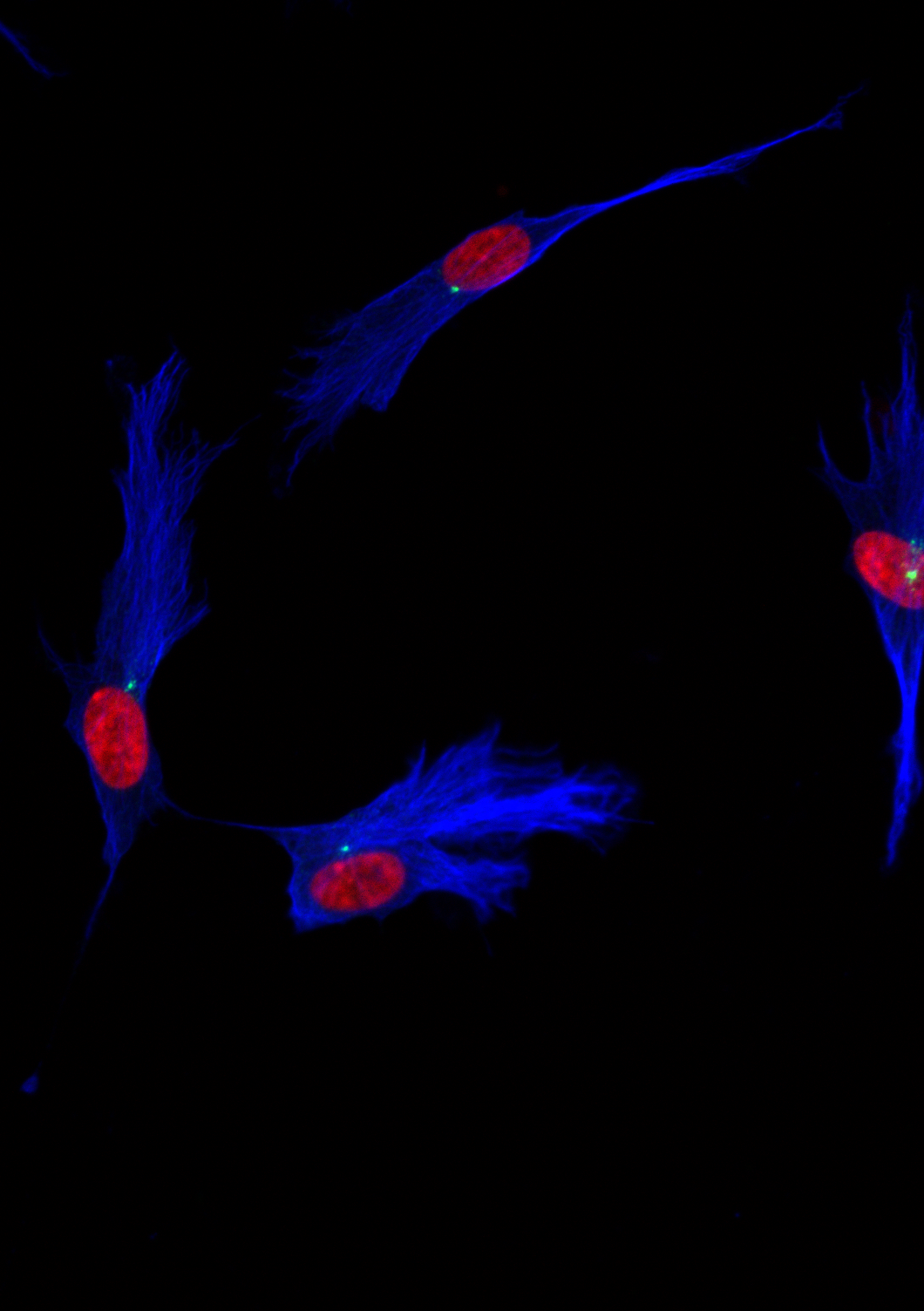

Supplement: Supplementary file 7 — Source Data for Figure 1 [file EMBJ-42-e112812-s006.zip › Figure 1 Source data/Fig 1C image -DOX.tif]

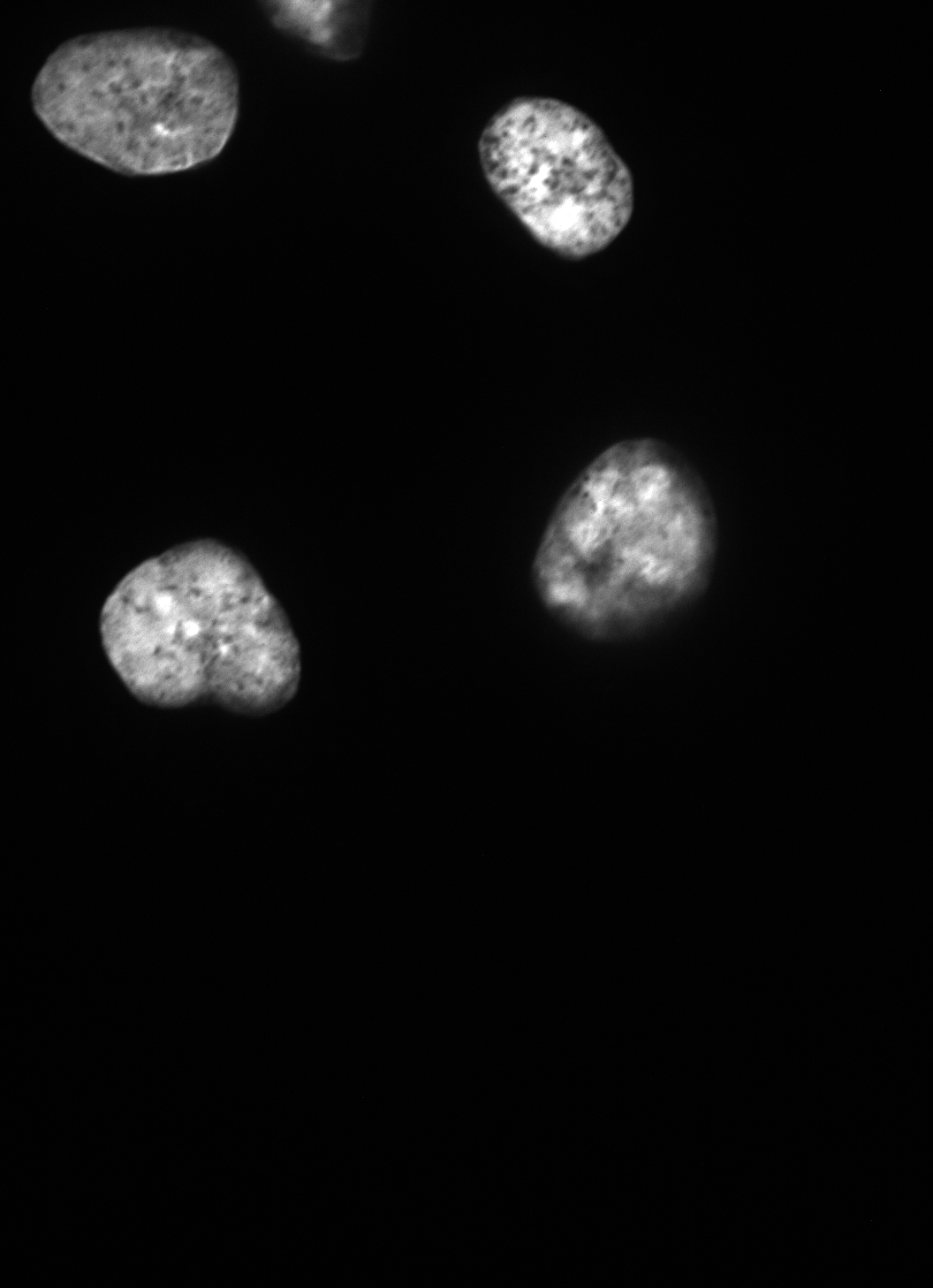

Supplement: Supplementary file 8 — Source Data for Figure 2 [file EMBJ-42-e112812-s003.zip › Figure 2 Source Data /Fig 2G image -DOX.tif]

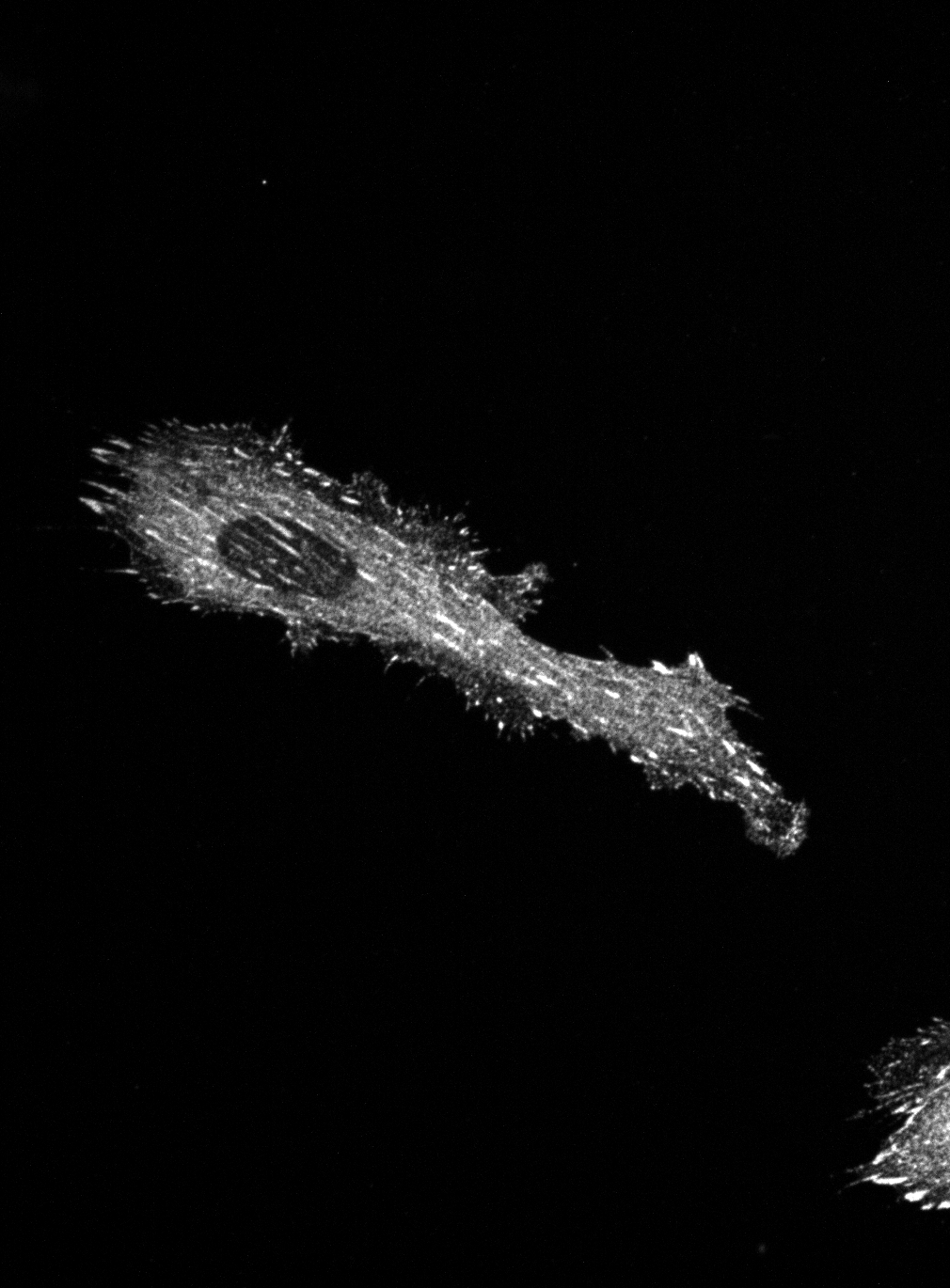

Supplement: Supplementary file 8 — Source Data for Figure 2 [file EMBJ-42-e112812-s003.zip › Figure 2 Source Data /Fig 2D image sip150 -DOX.tif]

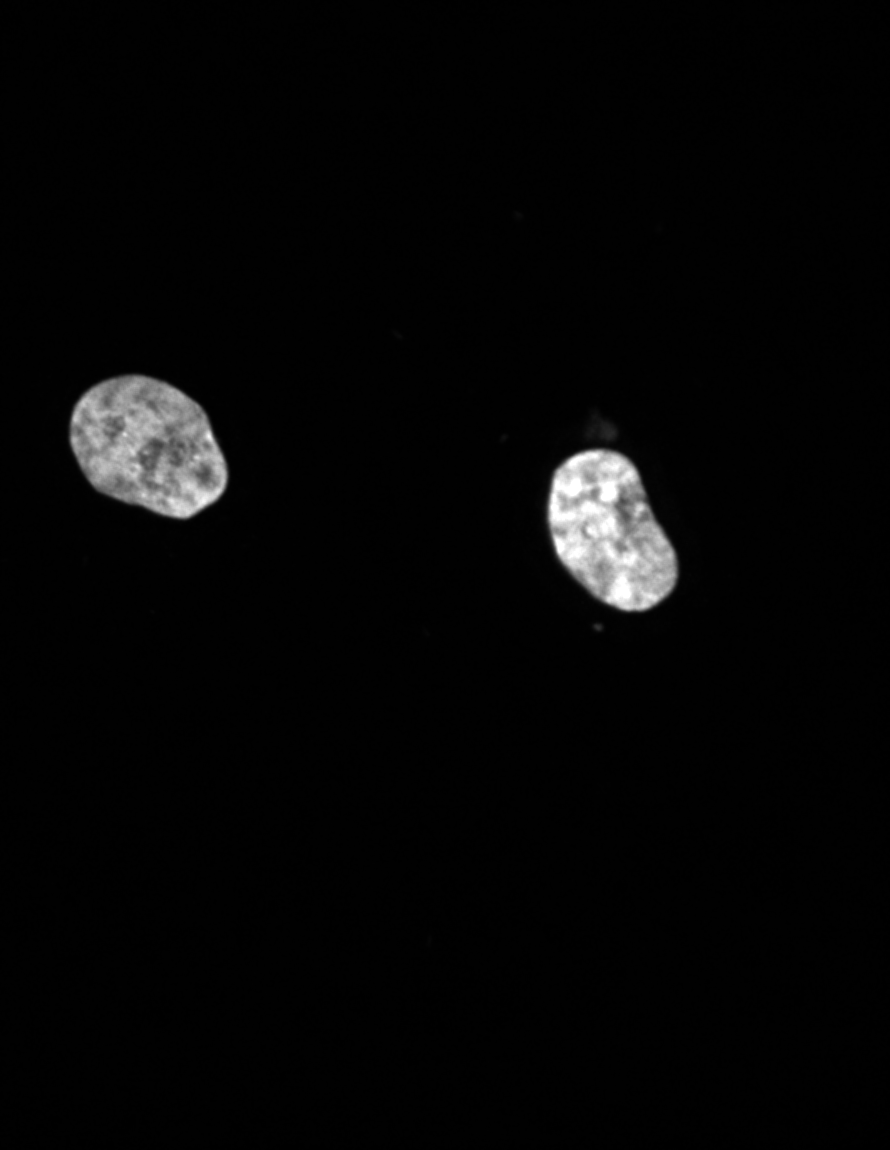

Supplement: Supplementary file 8 — Source Data for Figure 2 [file EMBJ-42-e112812-s003.zip › Figure 2 Source Data /Fig 2A image -DOX.tif]

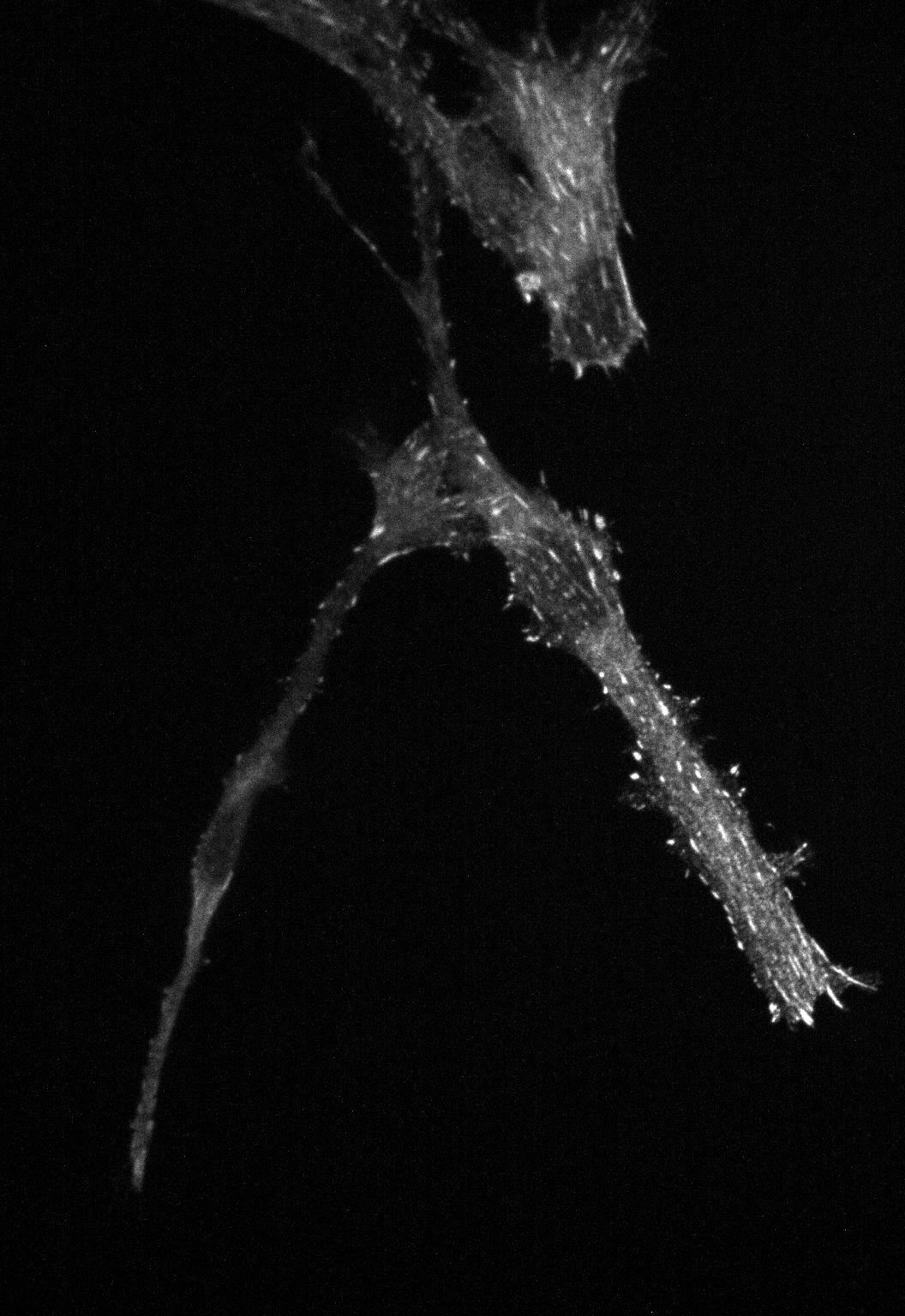

Supplement: Supplementary file 8 — Source Data for Figure 2 [file EMBJ-42-e112812-s003.zip › Figure 2 Source Data /Fig 2D image sip150 +DOX.tif]

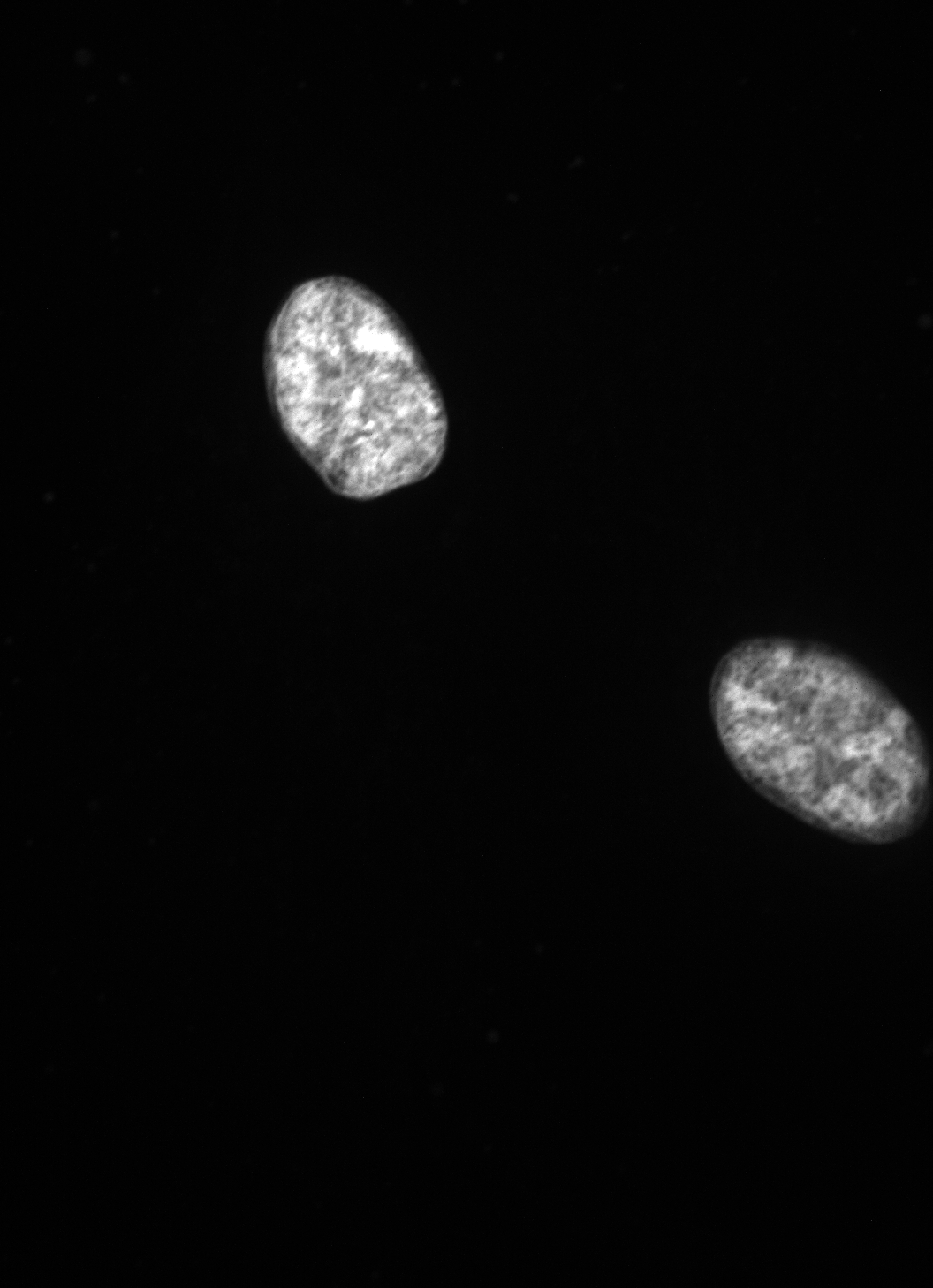

Supplement: Supplementary file 8 — Source Data for Figure 2 [file EMBJ-42-e112812-s003.zip › Figure 2 Source Data /Fig 2G image +DOX.tif]

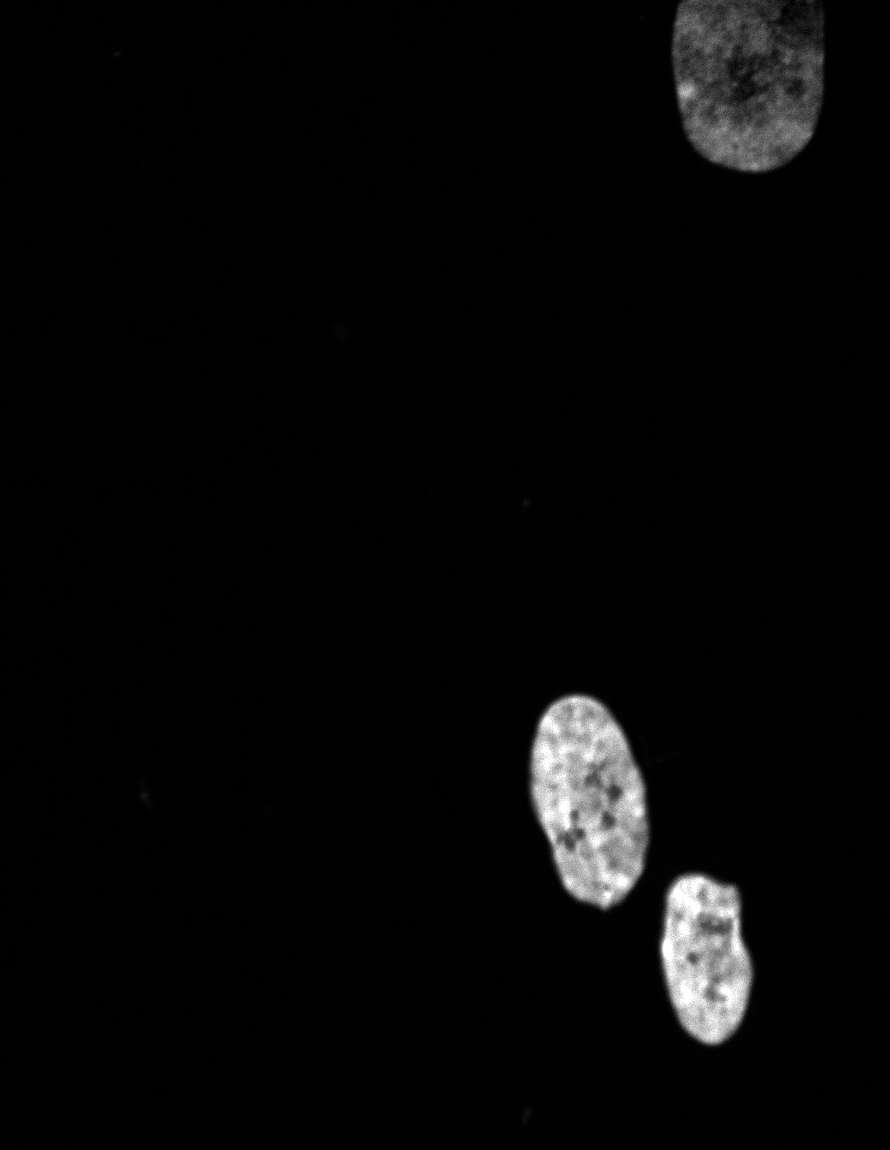

Supplement: Supplementary file 8 — Source Data for Figure 2 [file EMBJ-42-e112812-s003.zip › Figure 2 Source Data /Fig 2A image +DOX.tif]

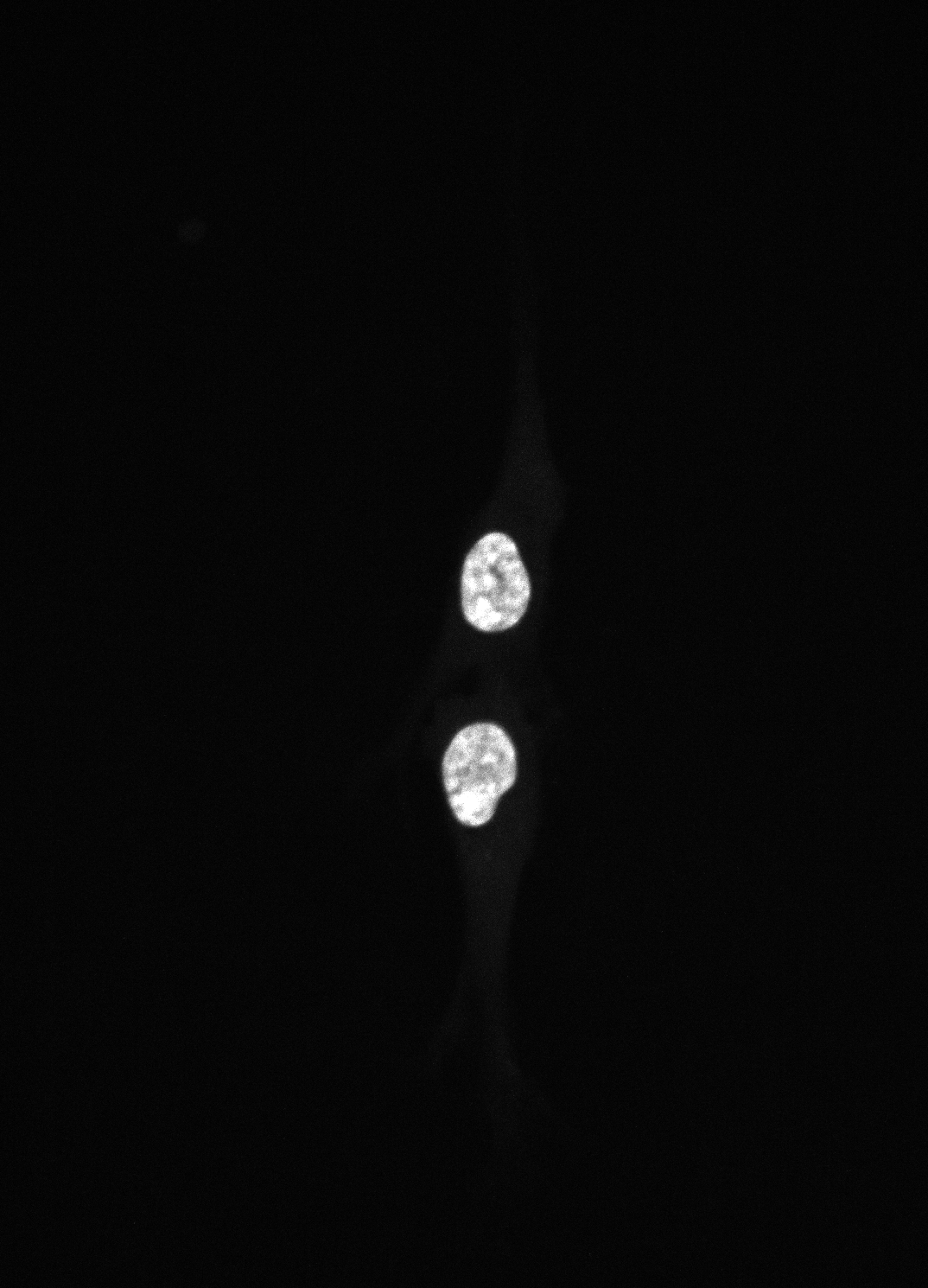

Supplement: Supplementary file 8 — Source Data for Figure 2 [file EMBJ-42-e112812-s003.zip › Figure 2 Source Data /Fig 2I image siKIF5B -DOX.tif]

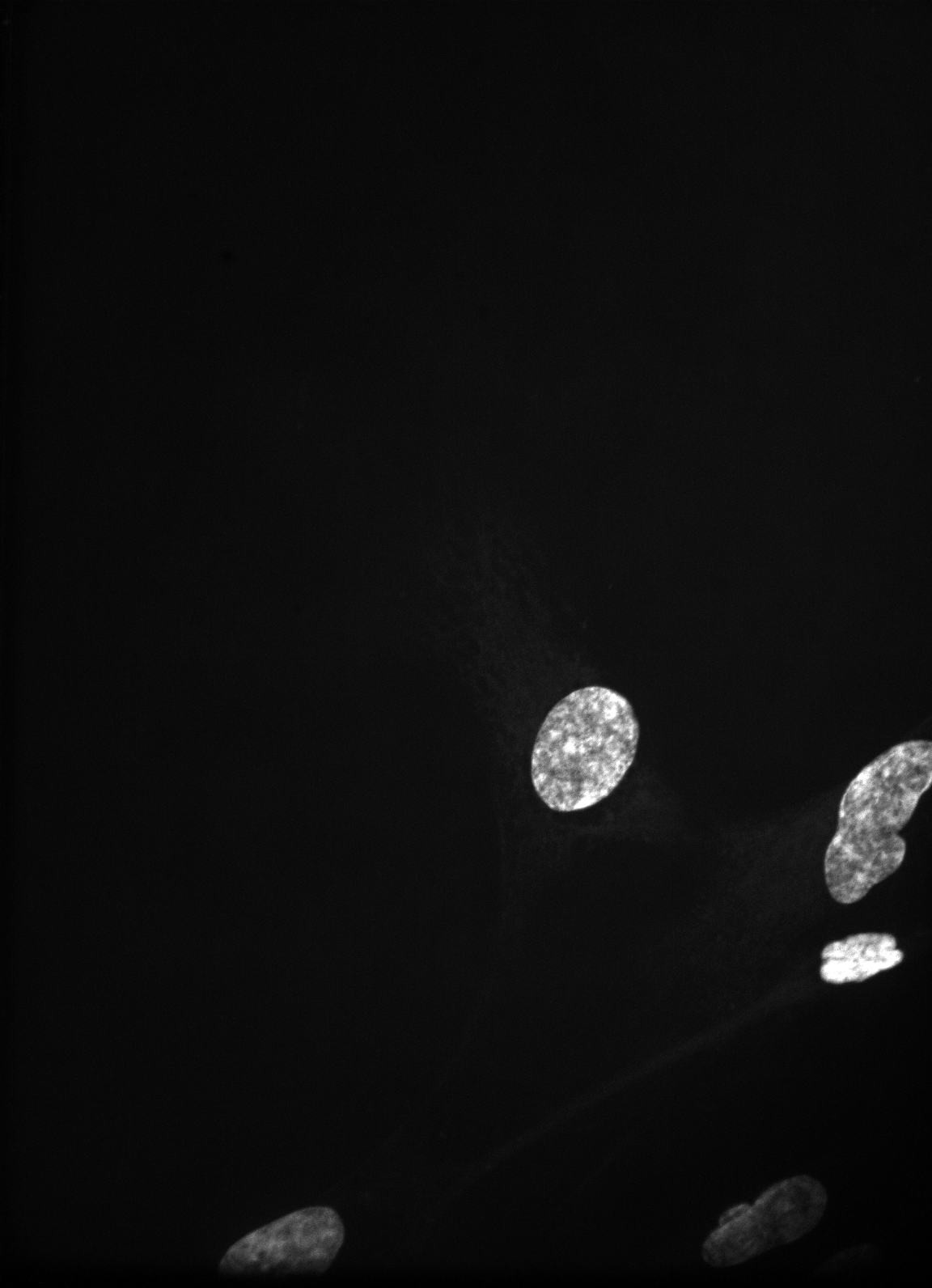

Supplement: Supplementary file 9 — Source Data for Figure 3 [file EMBJ-42-e112812-s014.zip › Figure 3 Source Data/Fig 3A image +DOX.tif]

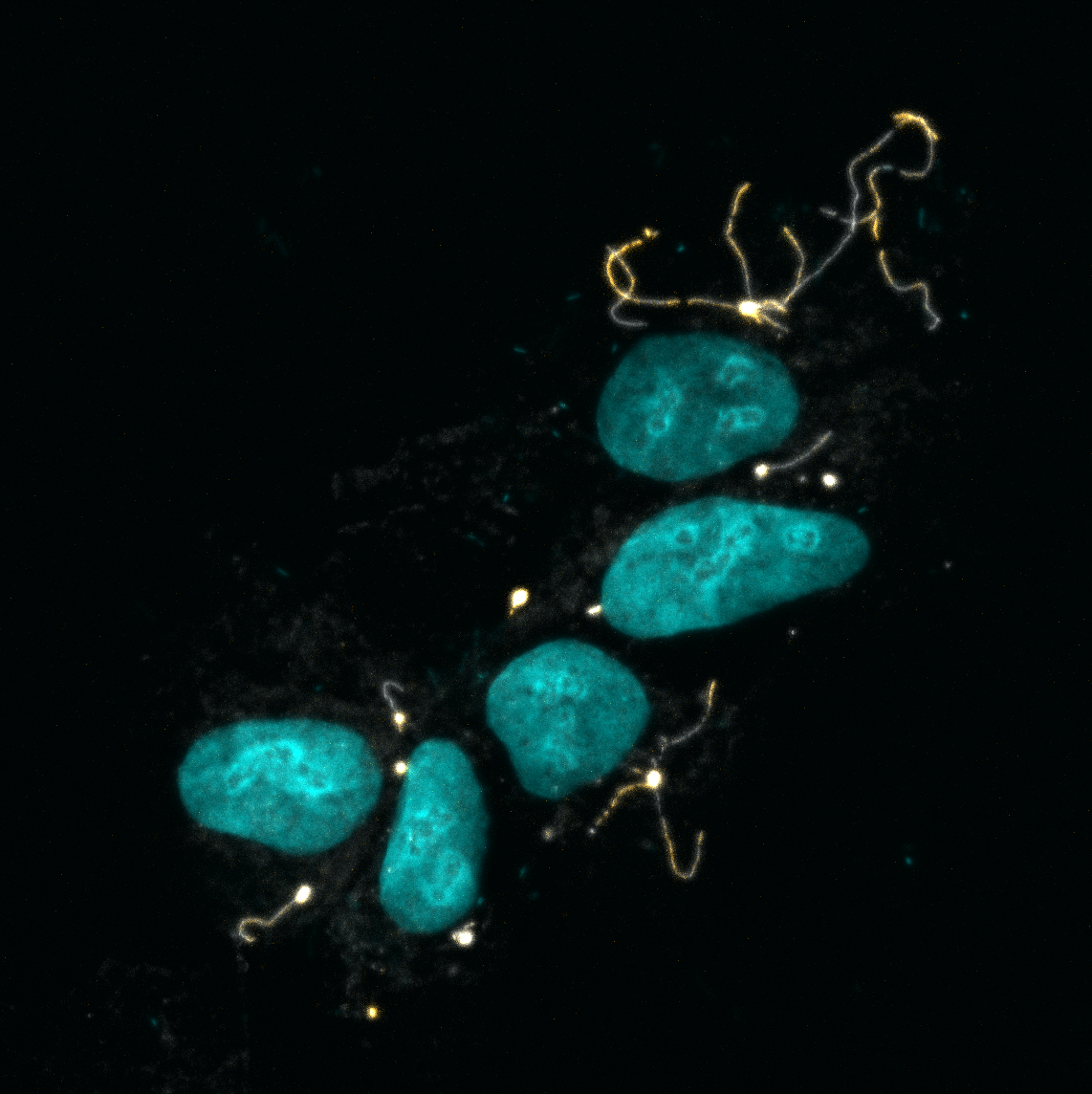

Supplement: Supplementary file 9 — Source Data for Figure 3 [file EMBJ-42-e112812-s014.zip › Figure 3 Source Data/Fig 3D image -DOX.tif]

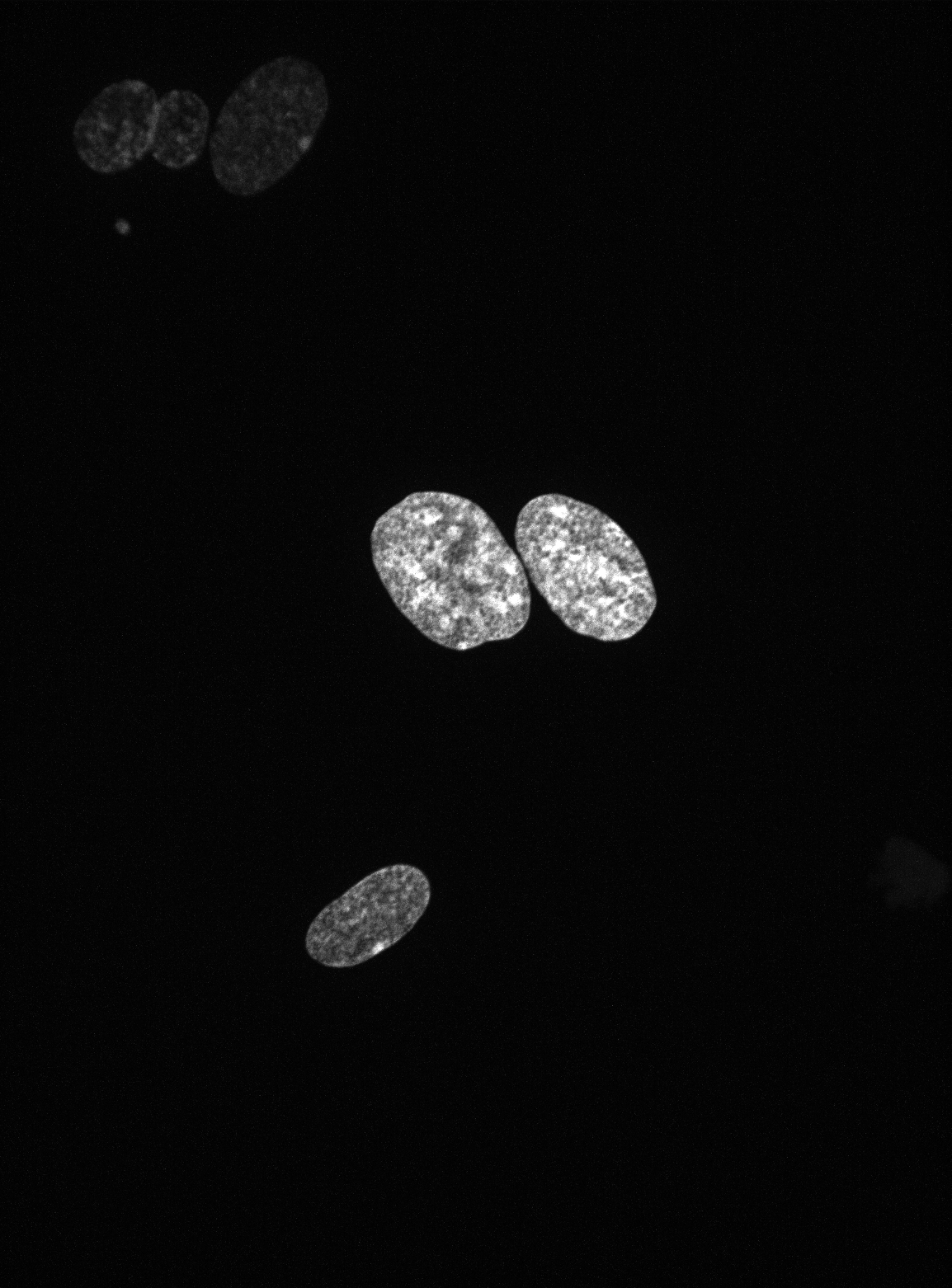

Supplement: Supplementary file 9 — Source Data for Figure 3 [file EMBJ-42-e112812-s014.zip › Figure 3 Source Data/Fig 3G image +DOX.tif]

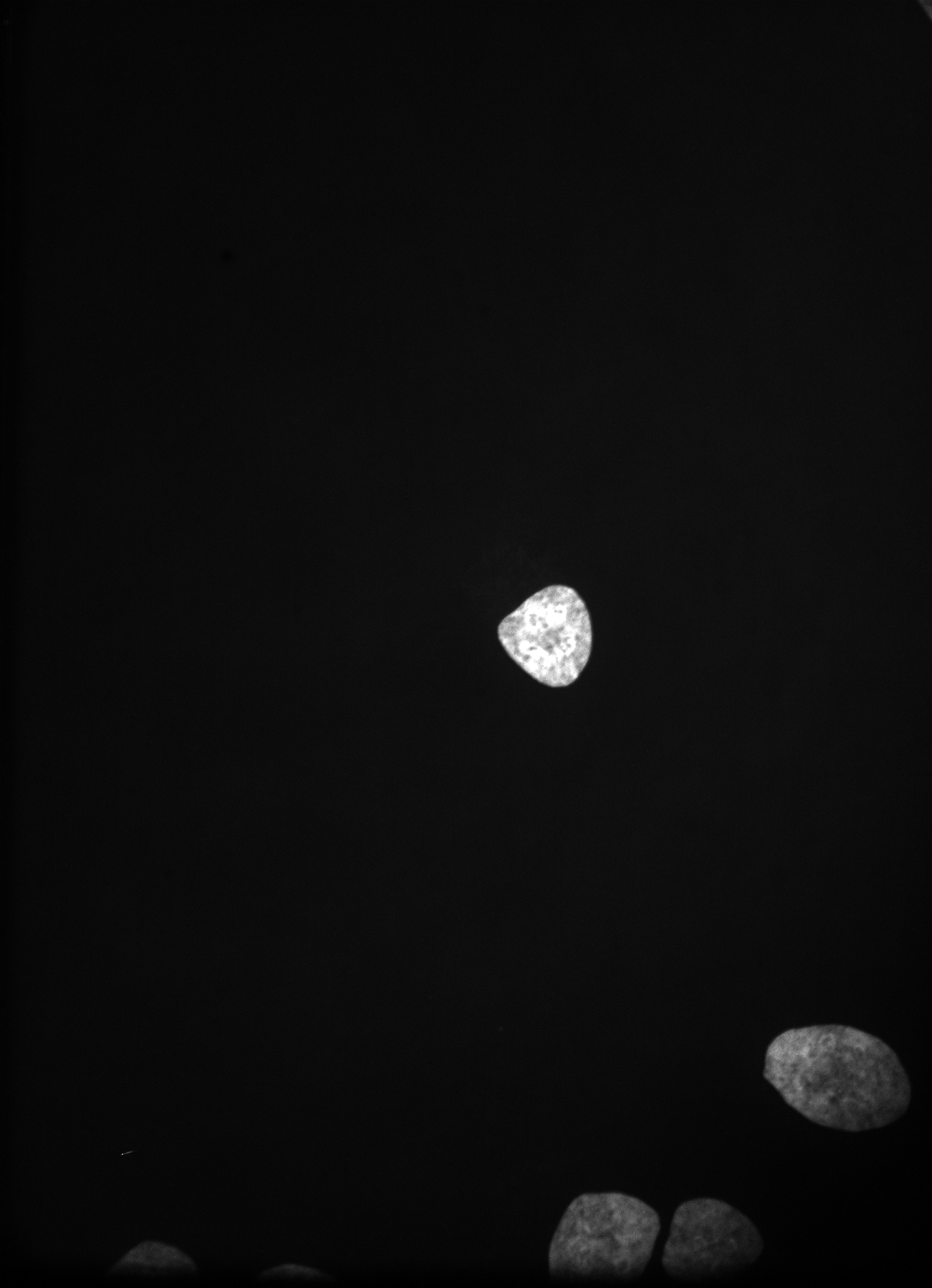

Supplement: Supplementary file 9 — Source Data for Figure 3 [file EMBJ-42-e112812-s014.zip › Figure 3 Source Data/Fig 3A image -DOX.tif]

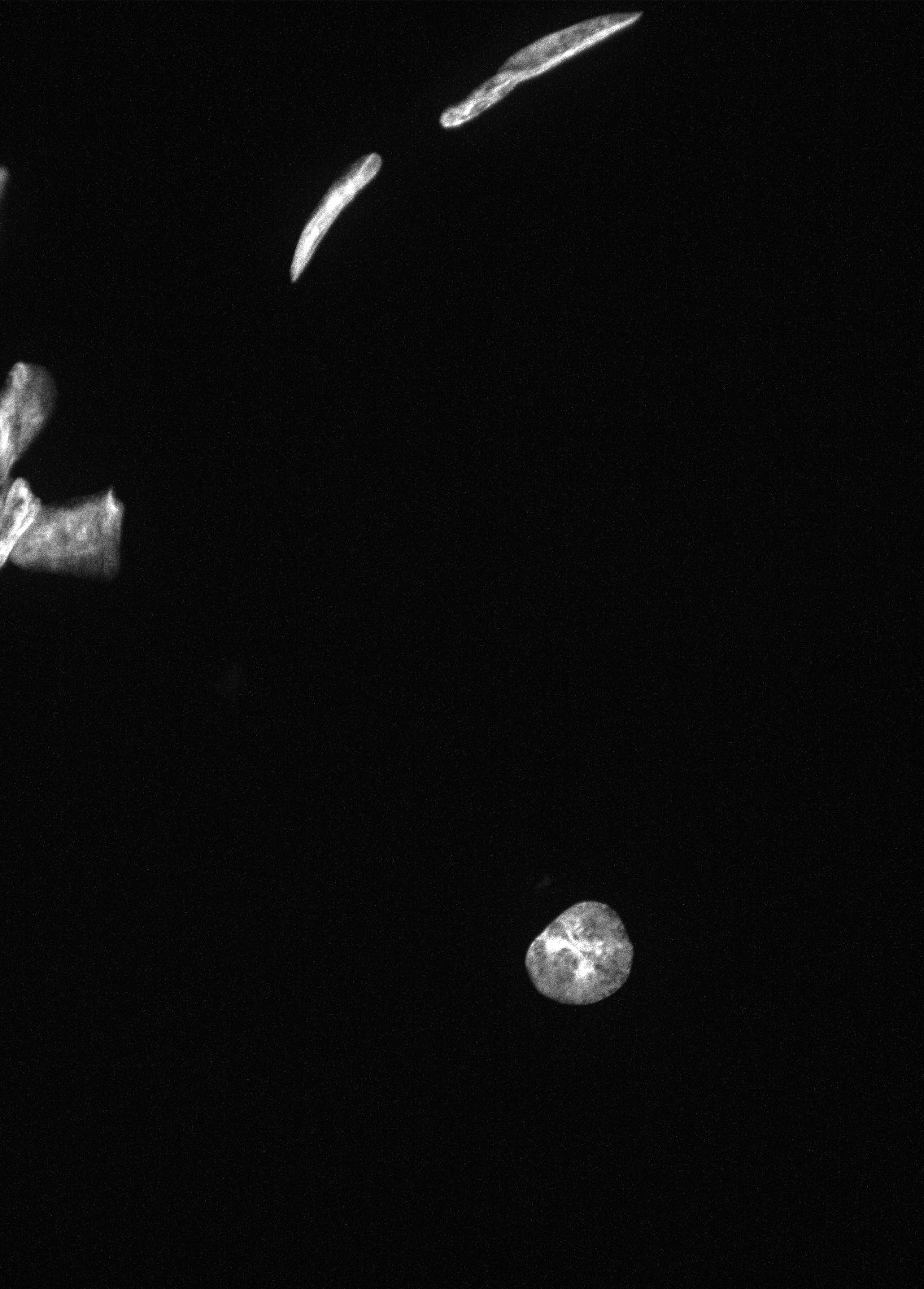

Supplement: Supplementary file 9 — Source Data for Figure 3 [file EMBJ-42-e112812-s014.zip › Figure 3 Source Data/Fig 3G image -DOX.tif]

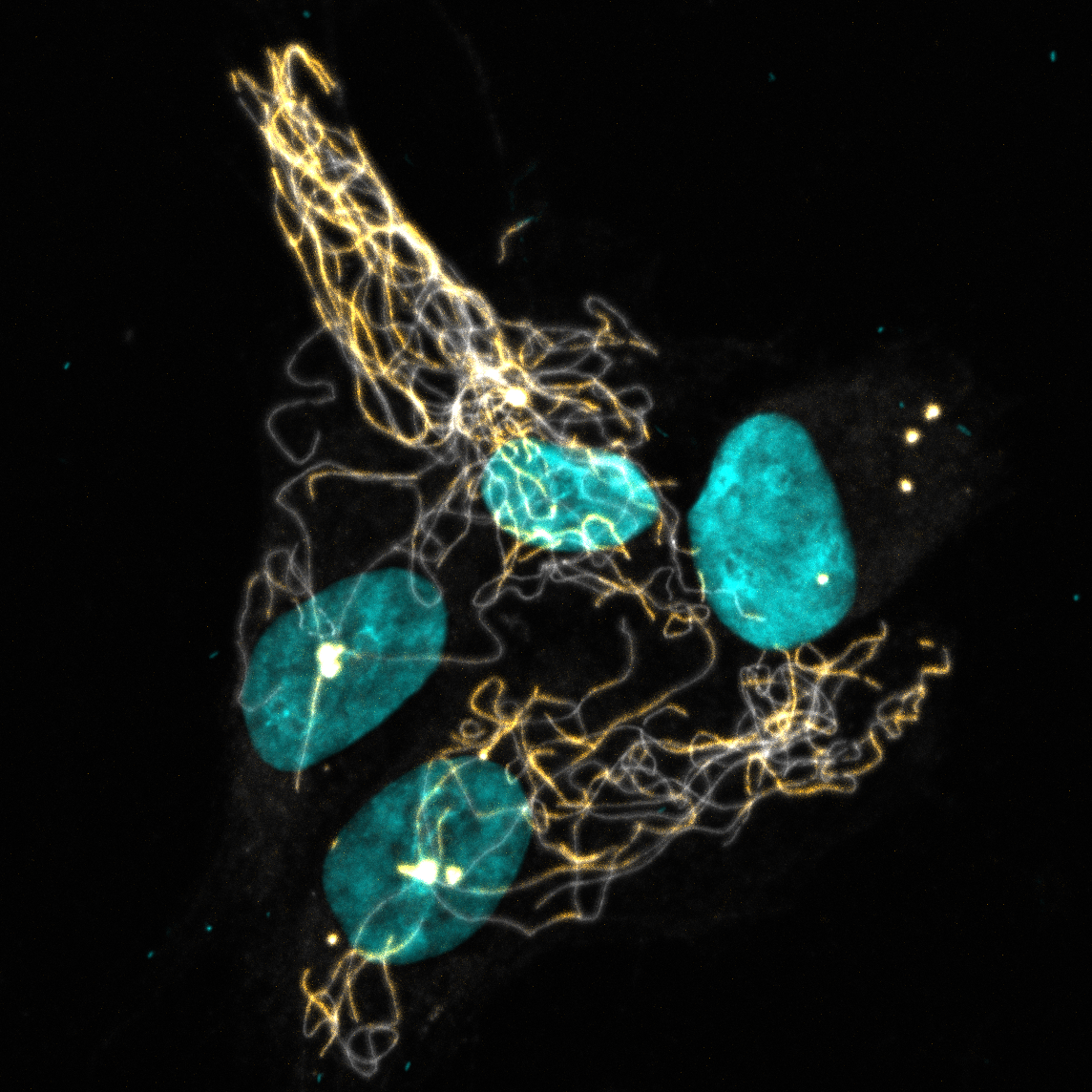

Supplement: Supplementary file 9 — Source Data for Figure 3 [file EMBJ-42-e112812-s014.zip › Figure 3 Source Data/Fig 3D image +DOX.tif]

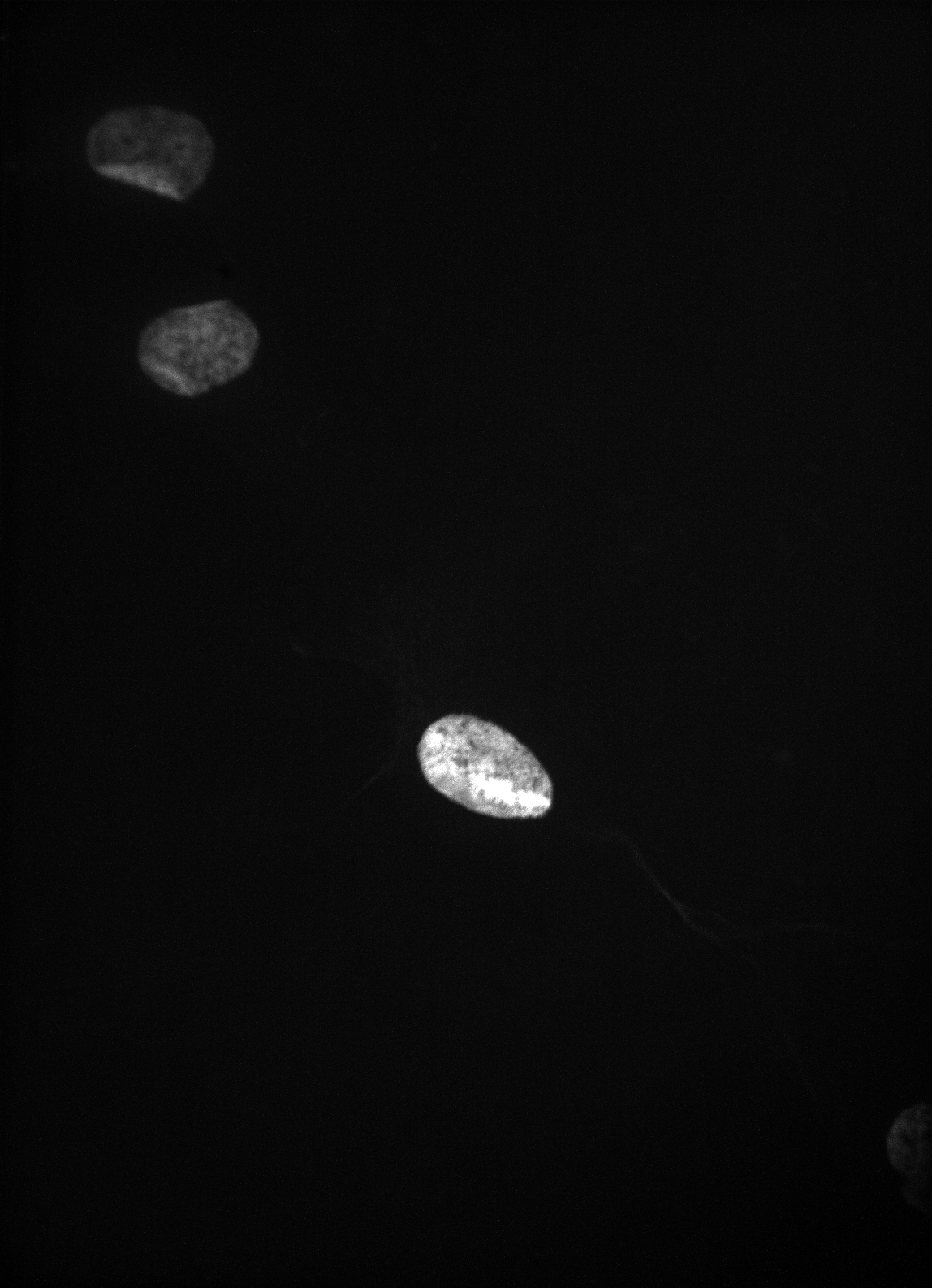

Supplement: Supplementary file 9 — Source Data for Figure 3 [file EMBJ-42-e112812-s014.zip › Figure 3 Source Data/Fig 3G image +DOX +Apocynin.tif]

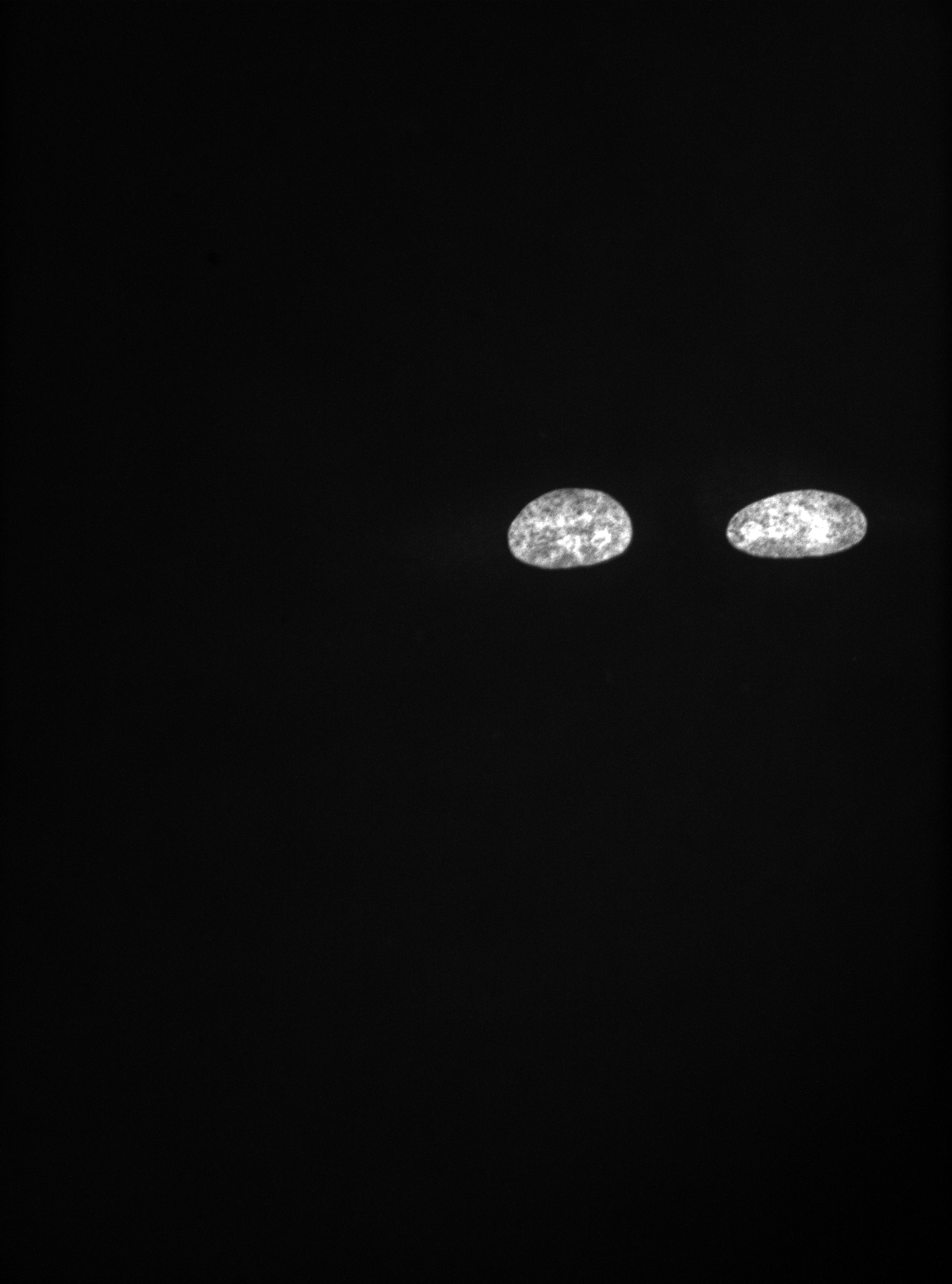

Supplement: Supplementary file 9 — Source Data for Figure 3 [file EMBJ-42-e112812-s014.zip › Figure 3 Source Data/Fig 3G image +H2O2.tif]

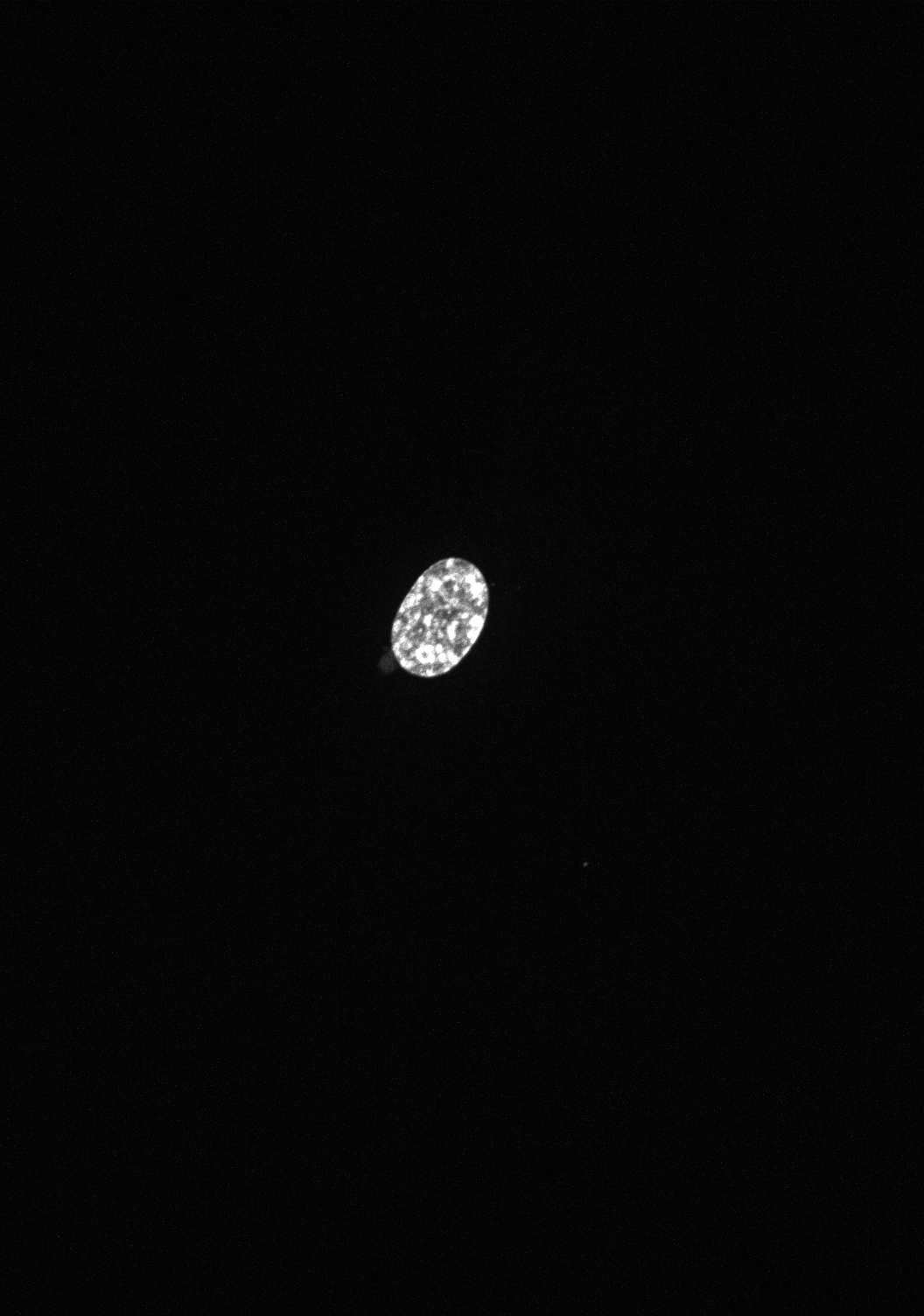

Supplement: Supplementary file 10 — Source Data for Figure 4 [file EMBJ-42-e112812-s013.zip › Figure 4 Source Data/Fig 4E image siATAT1 +DOX.tif]

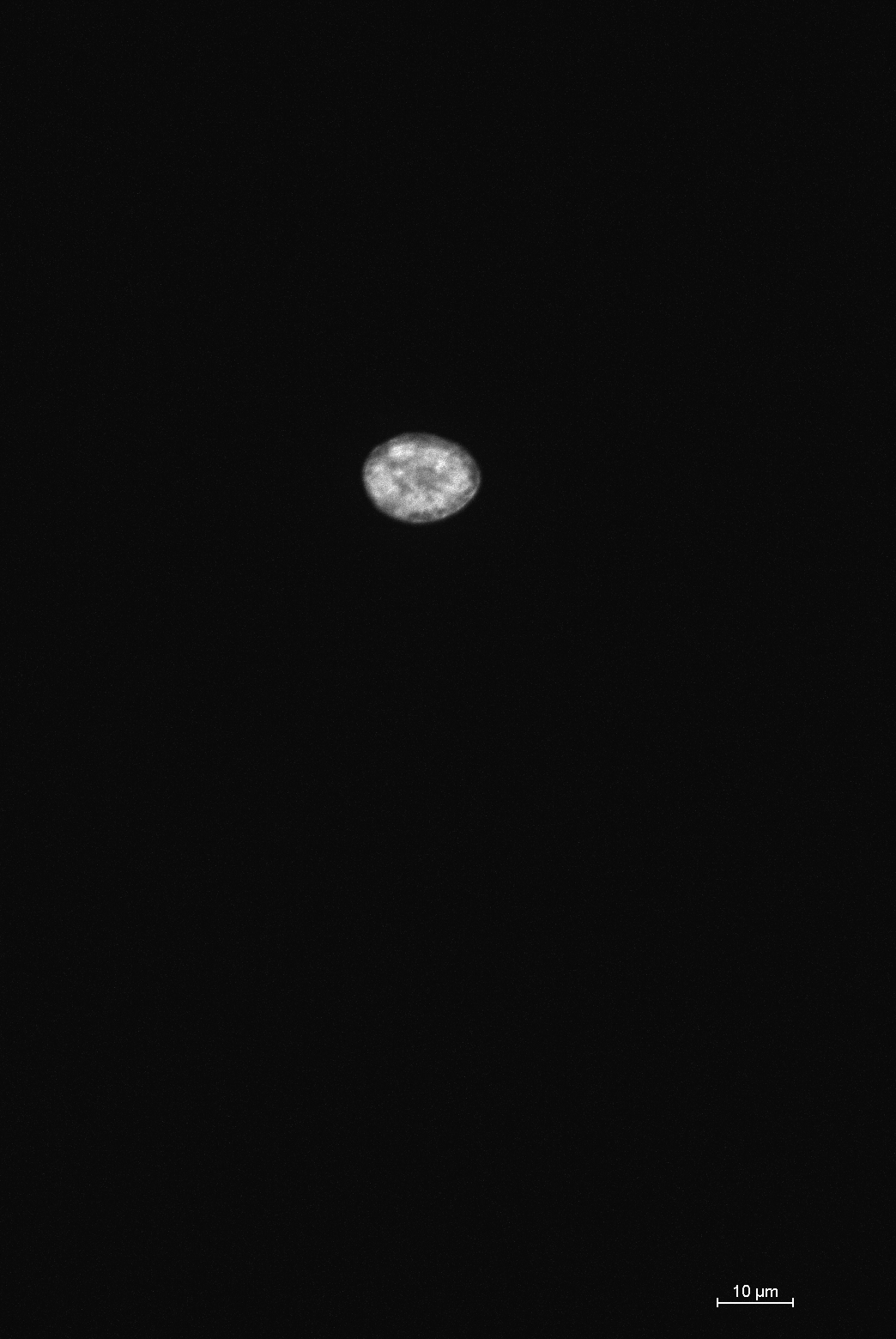

Supplement: Supplementary file 10 — Source Data for Figure 4 [file EMBJ-42-e112812-s013.zip › Figure 4 Source Data/Fig 4C image siCtr +DOX.tif]

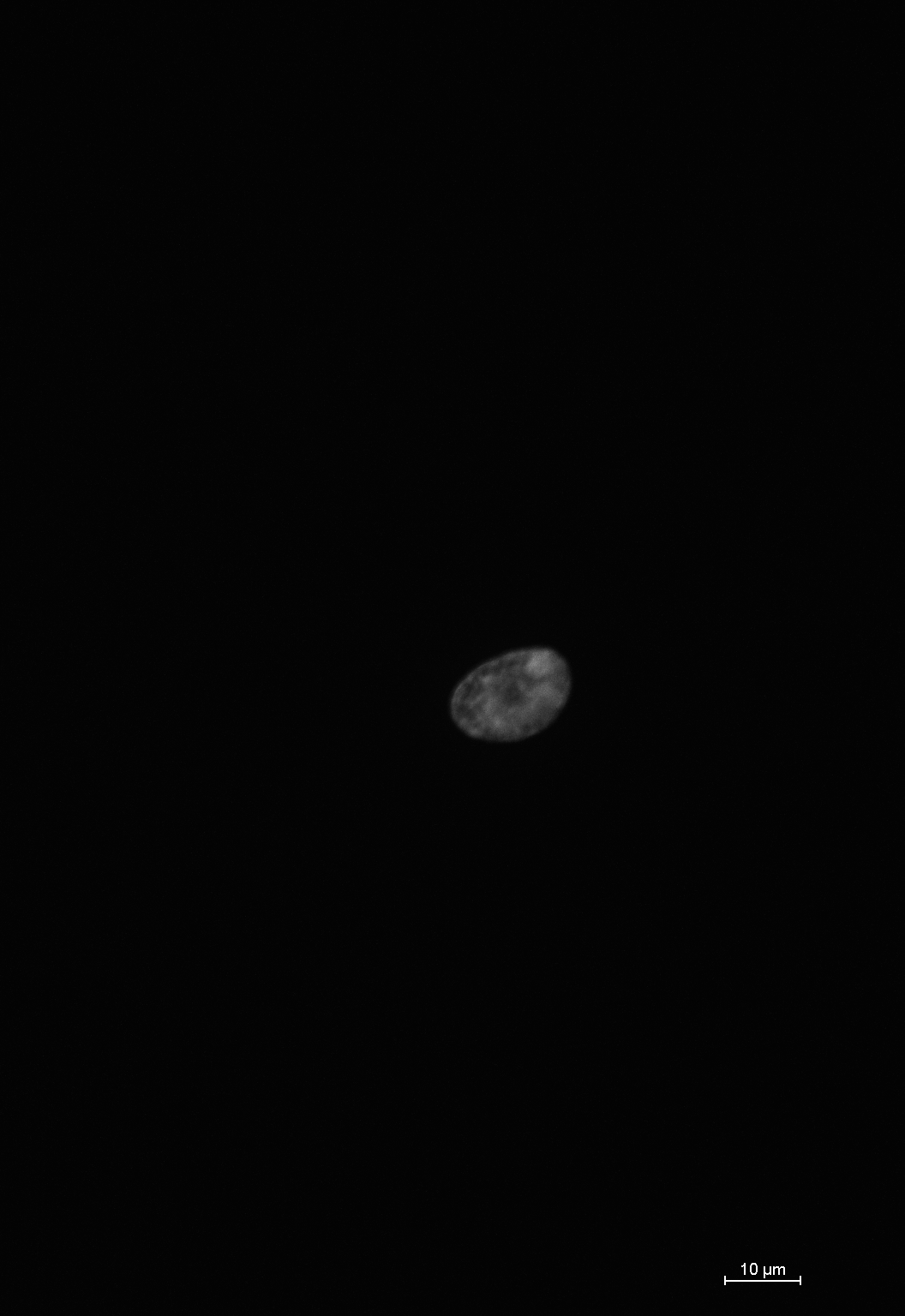

Supplement: Supplementary file 10 — Source Data for Figure 4 [file EMBJ-42-e112812-s013.zip › Figure 4 Source Data/Fig 4A image siATAT1 +DOX.tif]

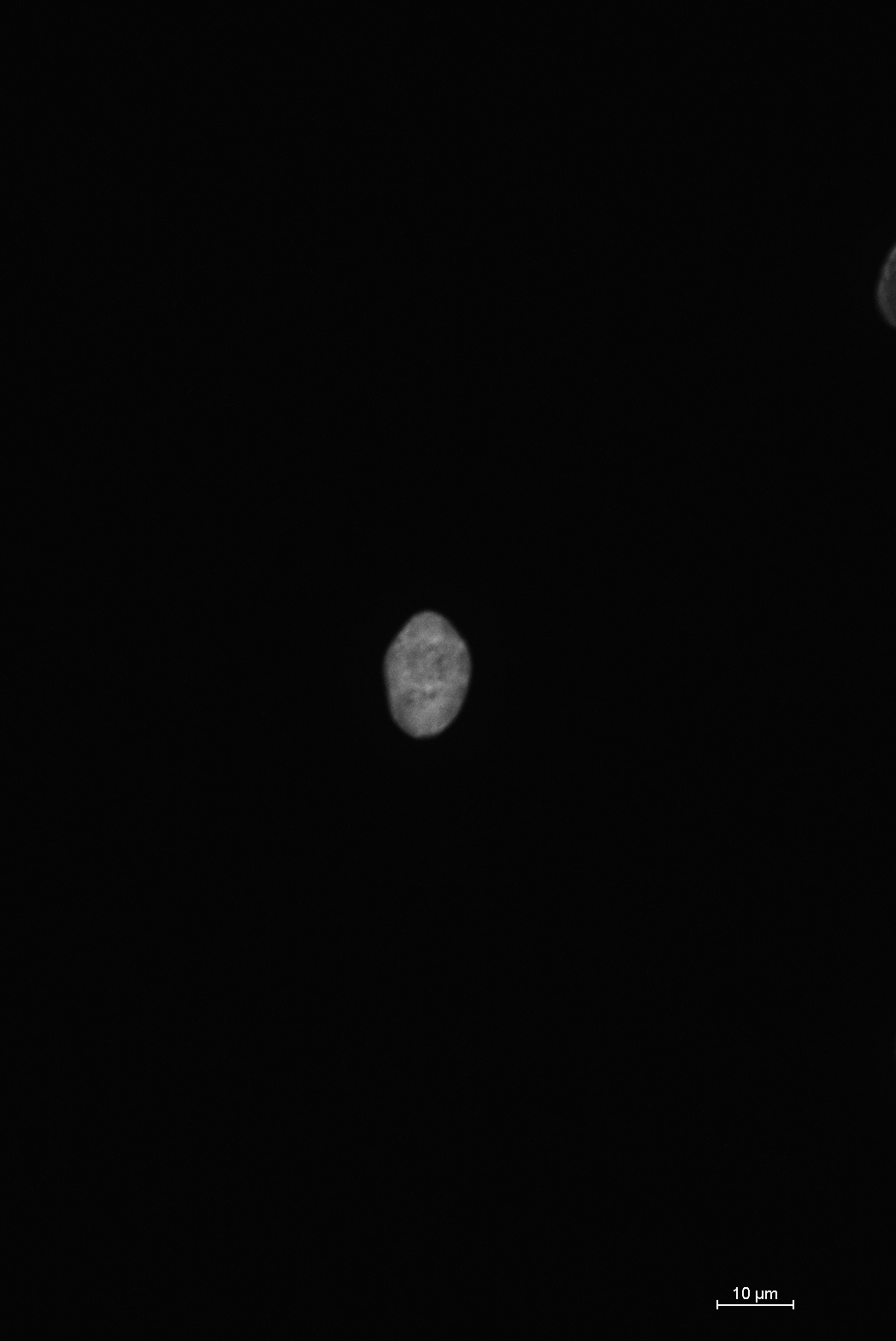

Supplement: Supplementary file 10 — Source Data for Figure 4 [file EMBJ-42-e112812-s013.zip › Figure 4 Source Data/Fig 4C image siATAT1 -DOX.tif]

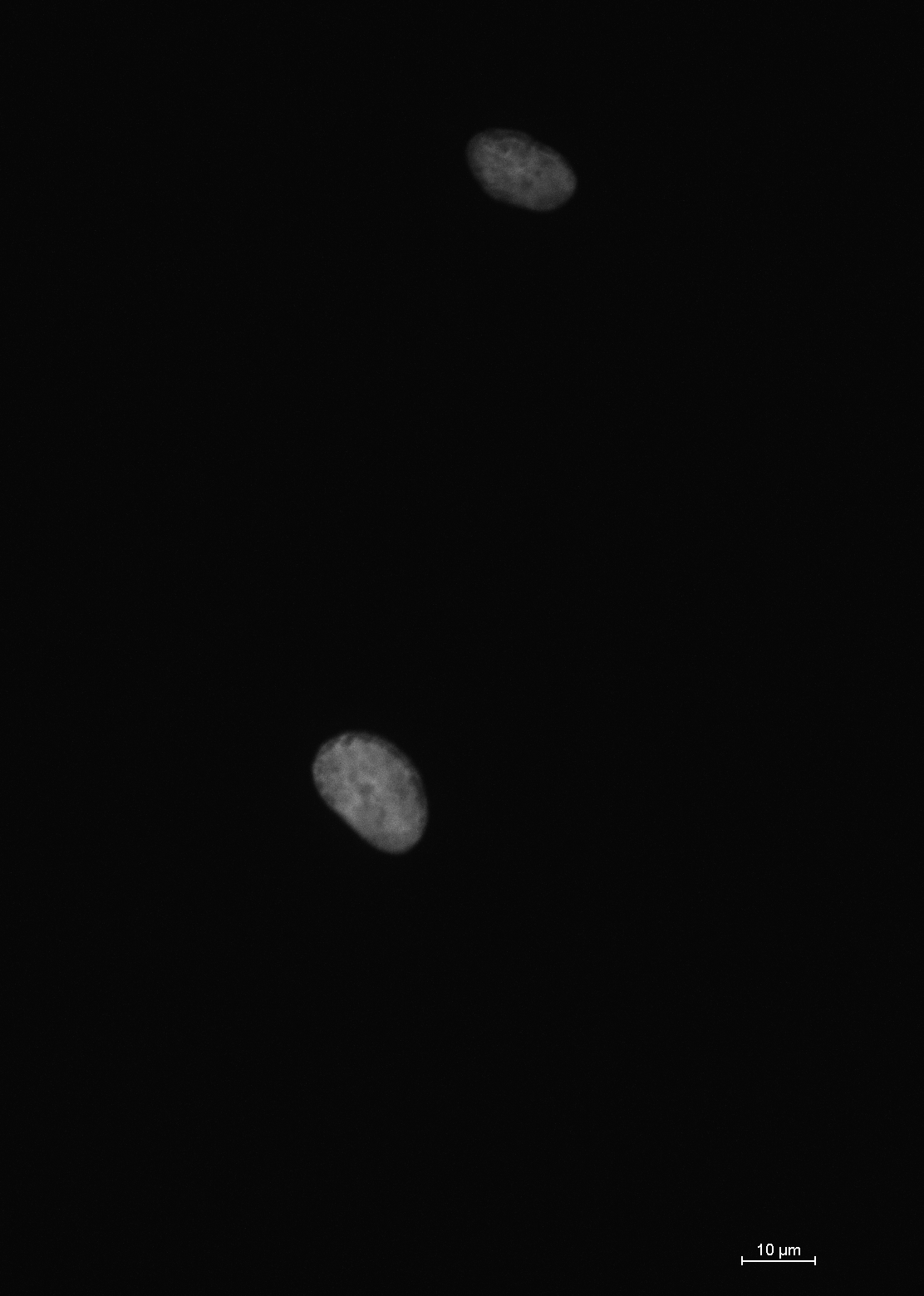

Supplement: Supplementary file 10 — Source Data for Figure 4 [file EMBJ-42-e112812-s013.zip › Figure 4 Source Data/Fig 4C image siCtr -DOX.tif]

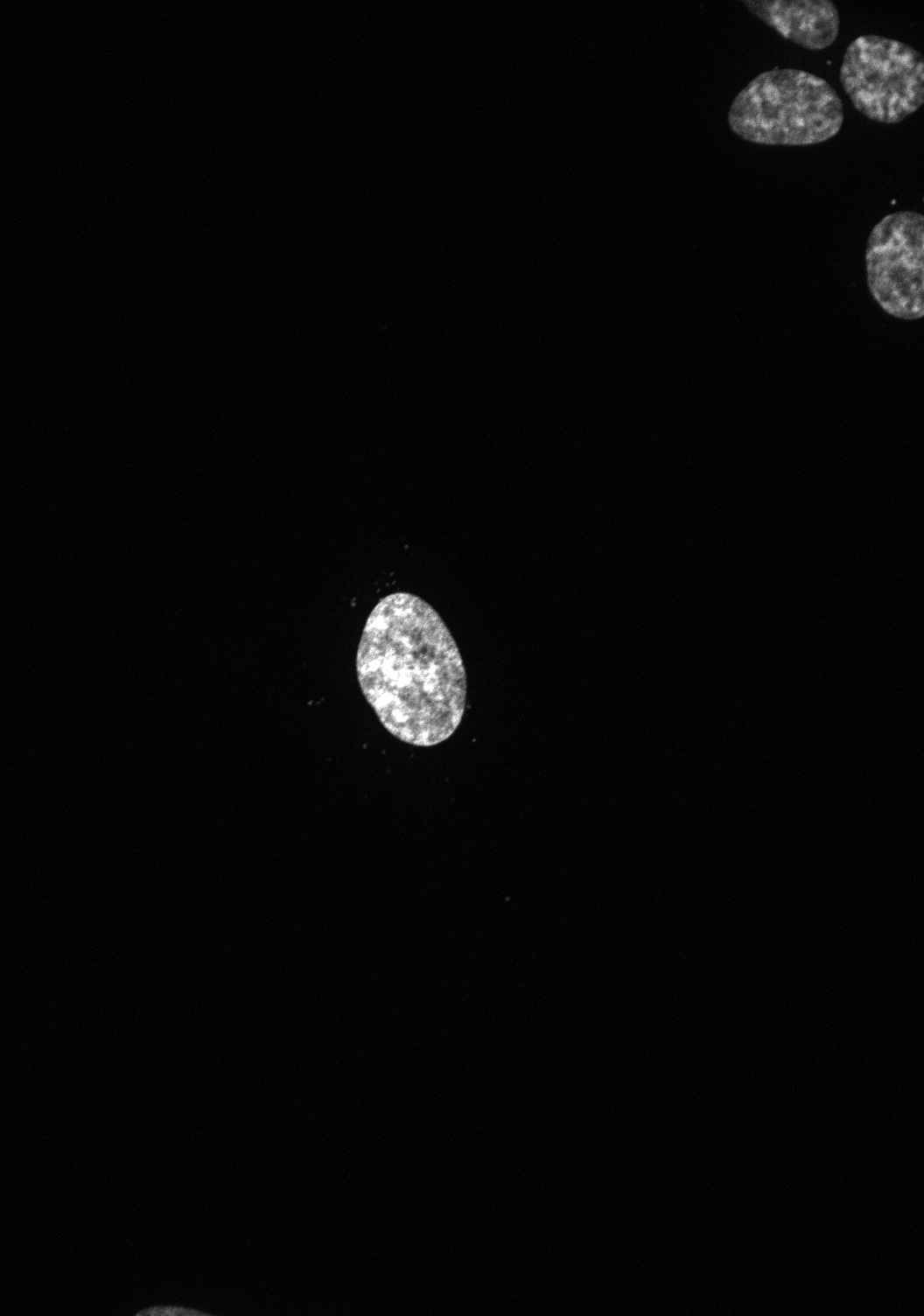

Supplement: Supplementary file 10 — Source Data for Figure 4 [file EMBJ-42-e112812-s013.zip › Figure 4 Source Data/Fig 4E image siATAT1 -DOX.tif]

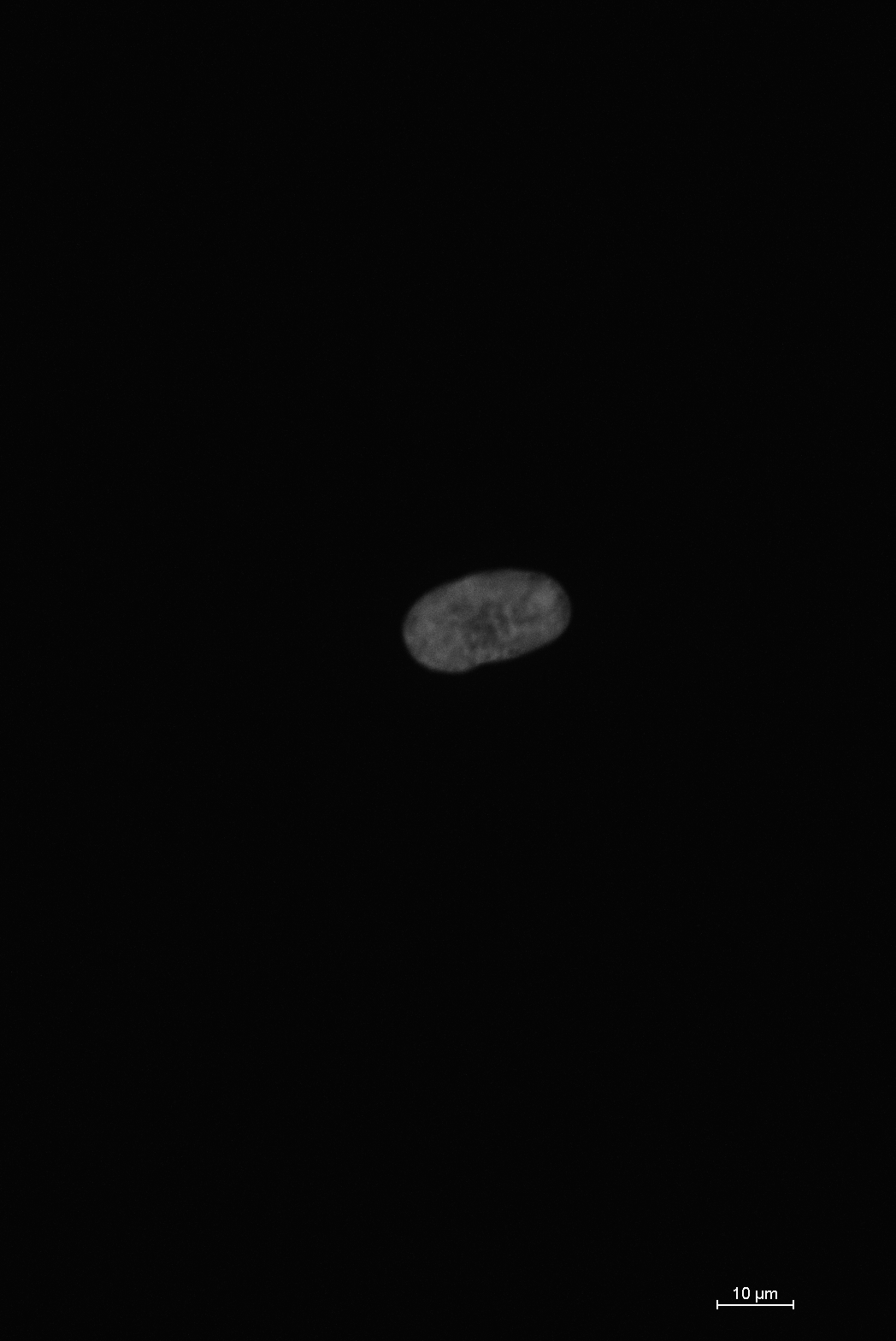

Supplement: Supplementary file 10 — Source Data for Figure 4 [file EMBJ-42-e112812-s013.zip › Figure 4 Source Data/Fig 4C image siATAT1 +DOX.tif]

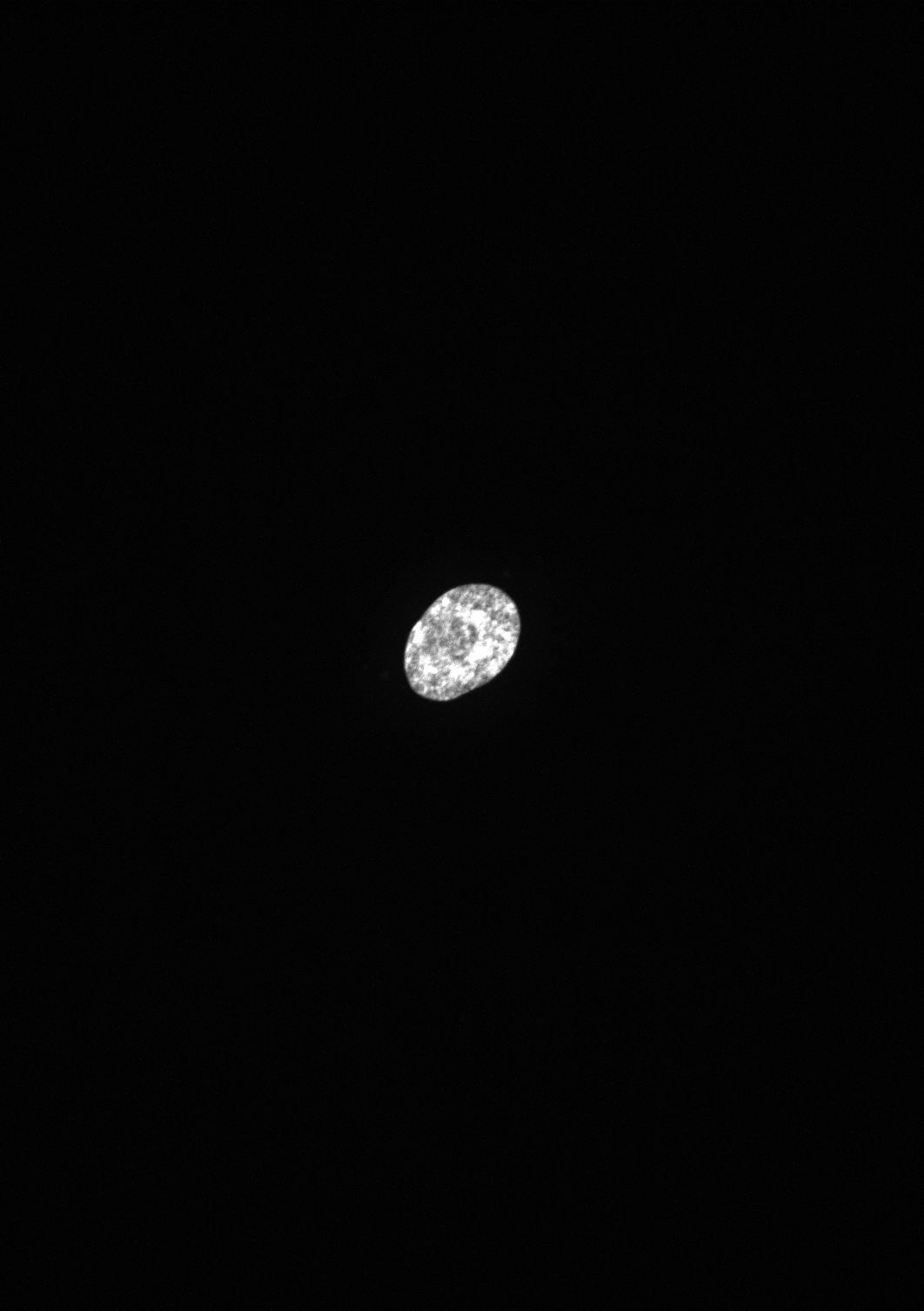

Supplement: Supplementary file 10 — Source Data for Figure 4 [file EMBJ-42-e112812-s013.zip › Figure 4 Source Data/Fig 4E image siCtr -DOX.tif]

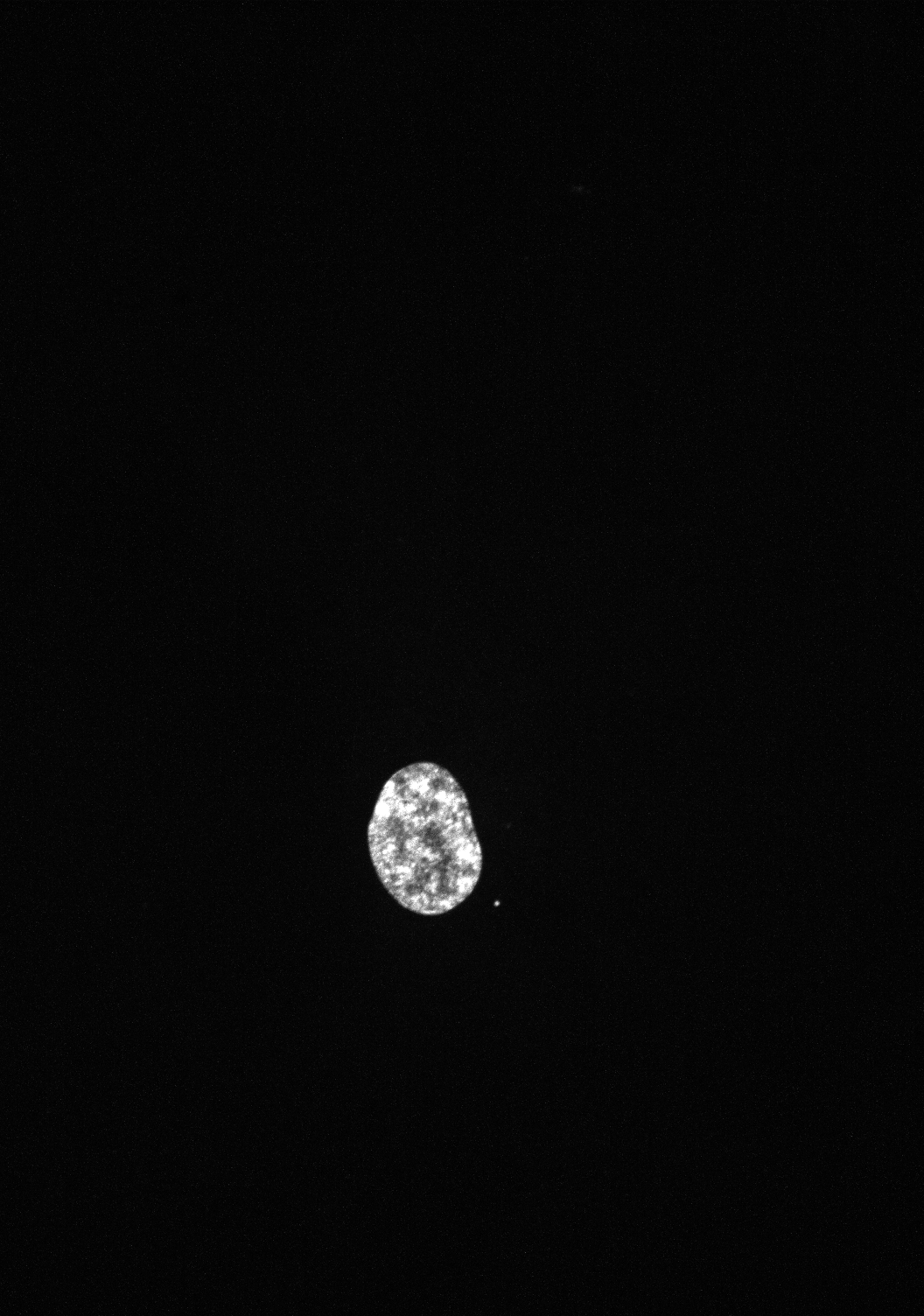

Supplement: Supplementary file 10 — Source Data for Figure 4 [file EMBJ-42-e112812-s013.zip › Figure 4 Source Data/Fig 4E image siCtr +DOX.tif]

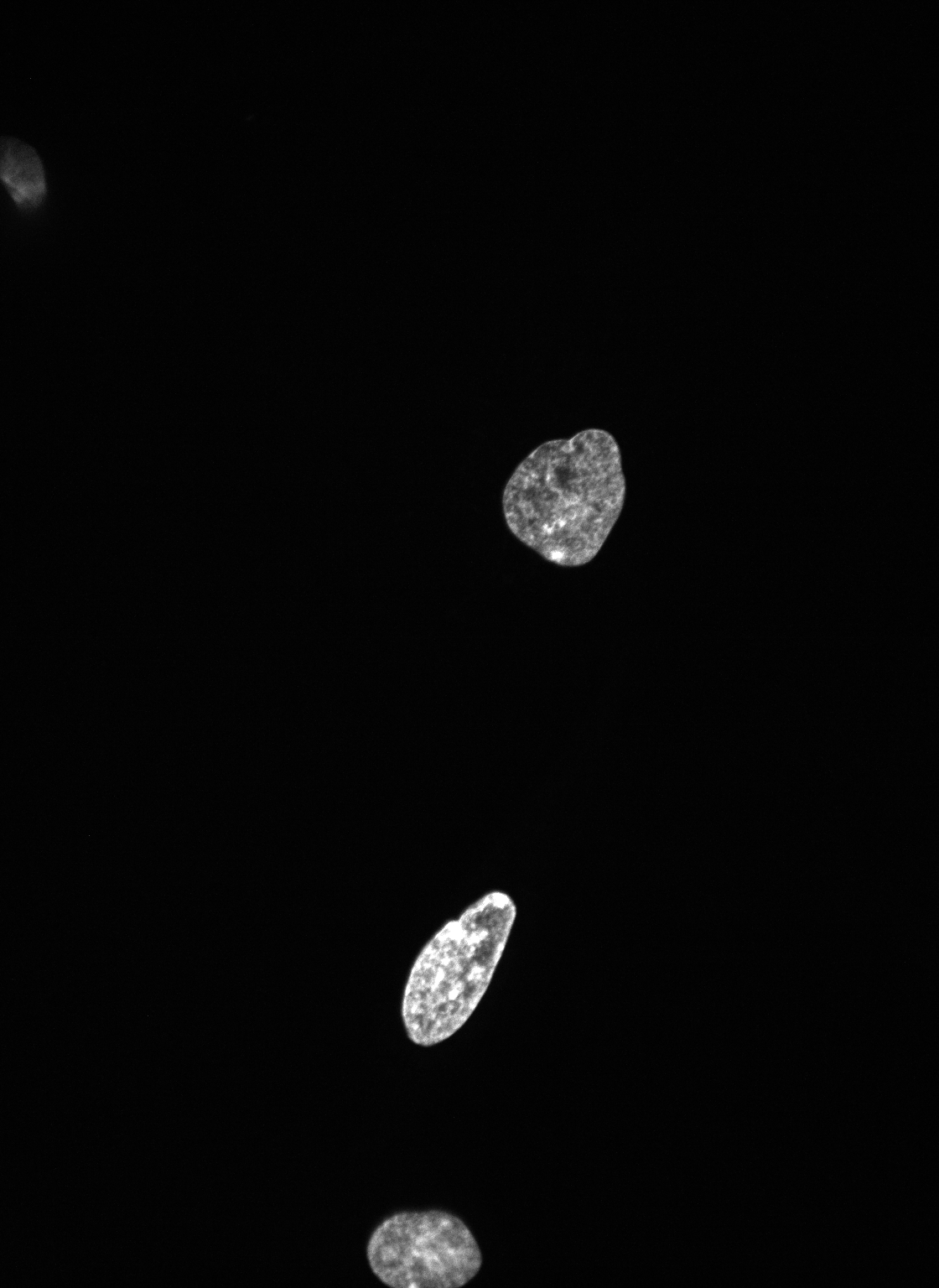

Supplement: Supplementary file 11 — Source Data for Figure 5 [file EMBJ-42-e112812-s010.zip › Figure 5 Source Data/Fig 5I image Ctr.tif]

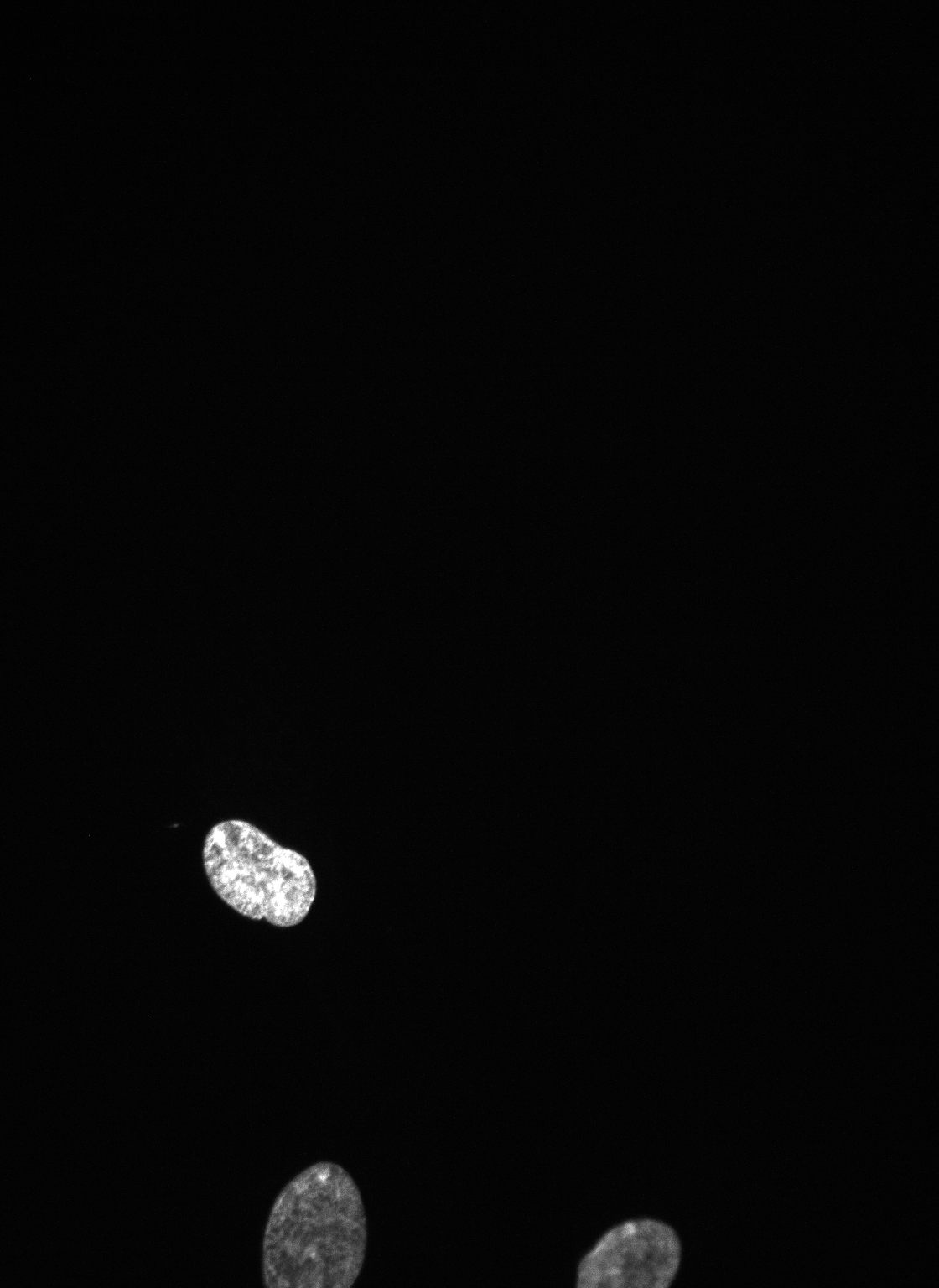

Supplement: Supplementary file 11 — Source Data for Figure 5 [file EMBJ-42-e112812-s010.zip › Figure 5 Source Data/Fig 5E image Tubacin.tif]

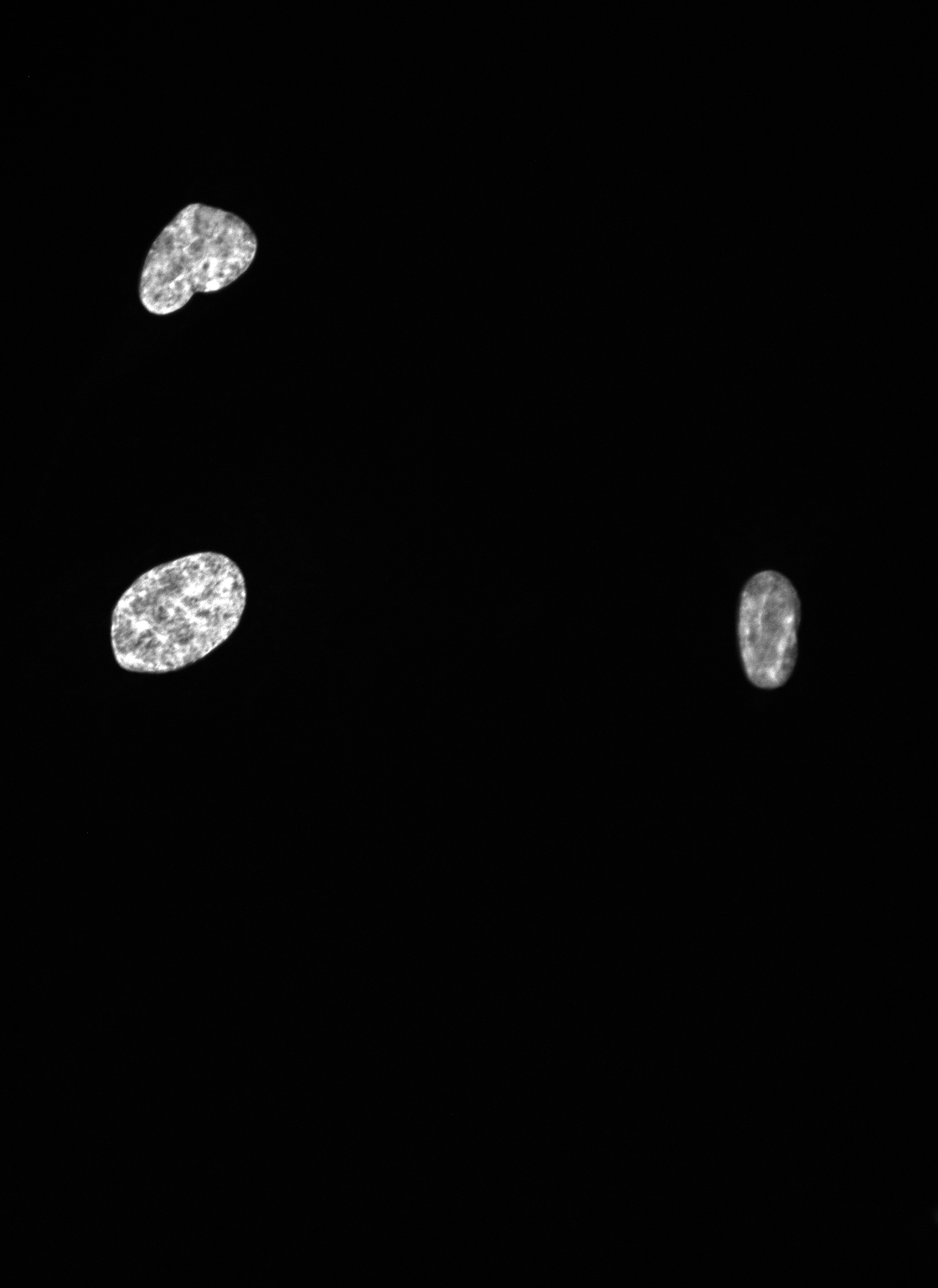

Supplement: Supplementary file 11 — Source Data for Figure 5 [file EMBJ-42-e112812-s010.zip › Figure 5 Source Data/Fig 5I image ATAT1OE.tif]

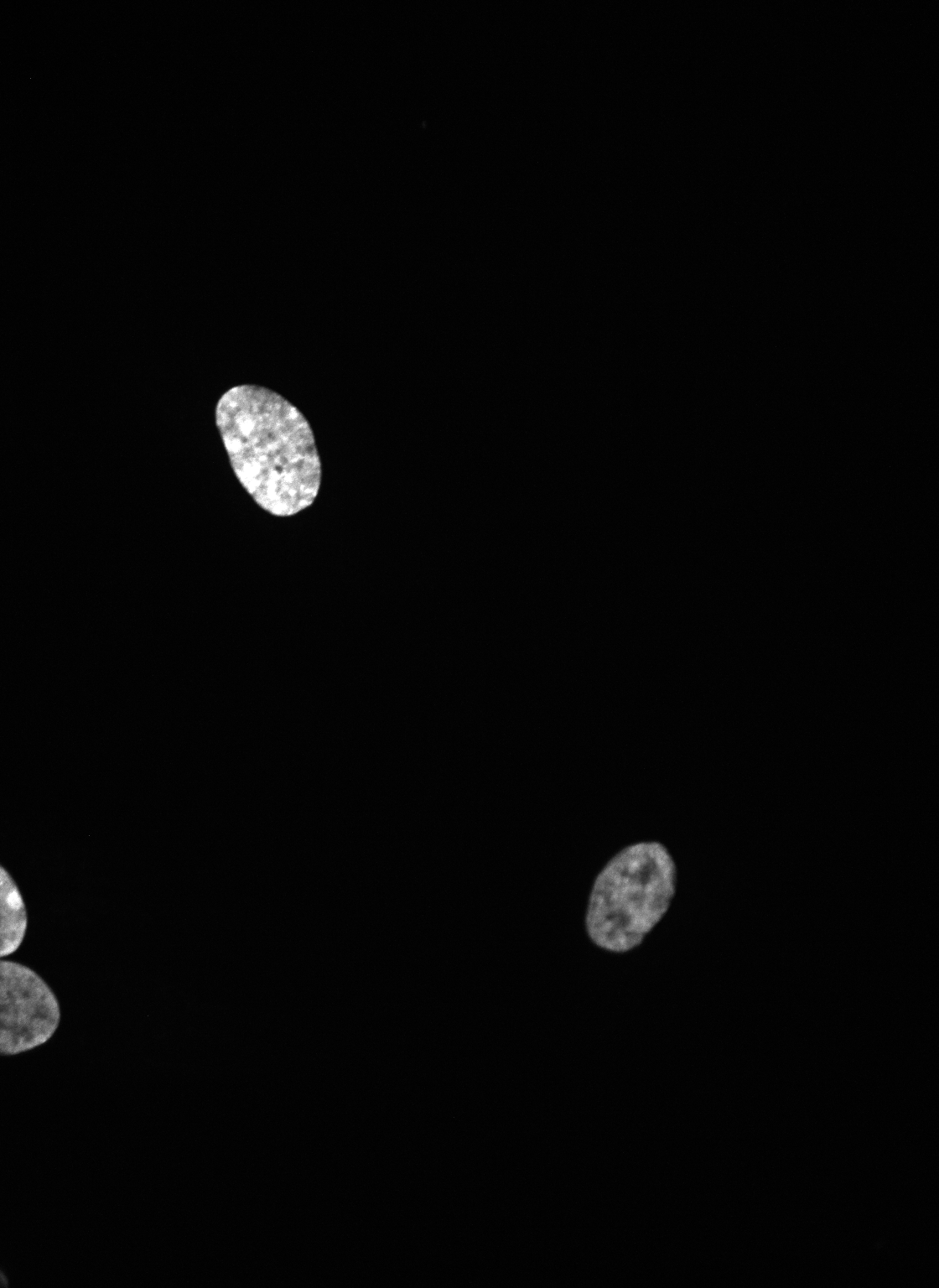

Supplement: Supplementary file 11 — Source Data for Figure 5 [file EMBJ-42-e112812-s010.zip › Figure 5 Source Data/Fig 5G image Ctr.tif]

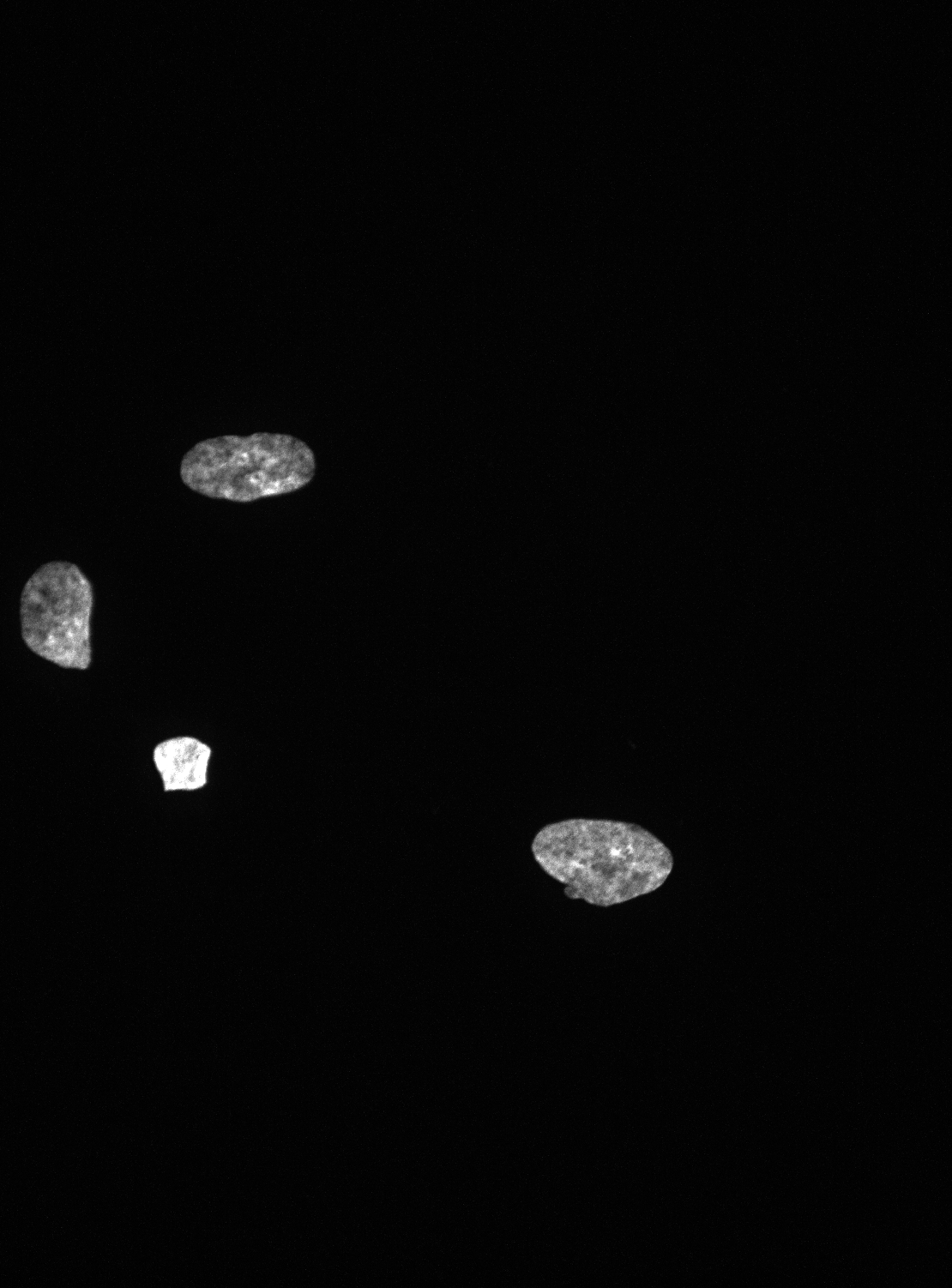

Supplement: Supplementary file 11 — Source Data for Figure 5 [file EMBJ-42-e112812-s010.zip › Figure 5 Source Data/Fig 5C image Ctr.tif]

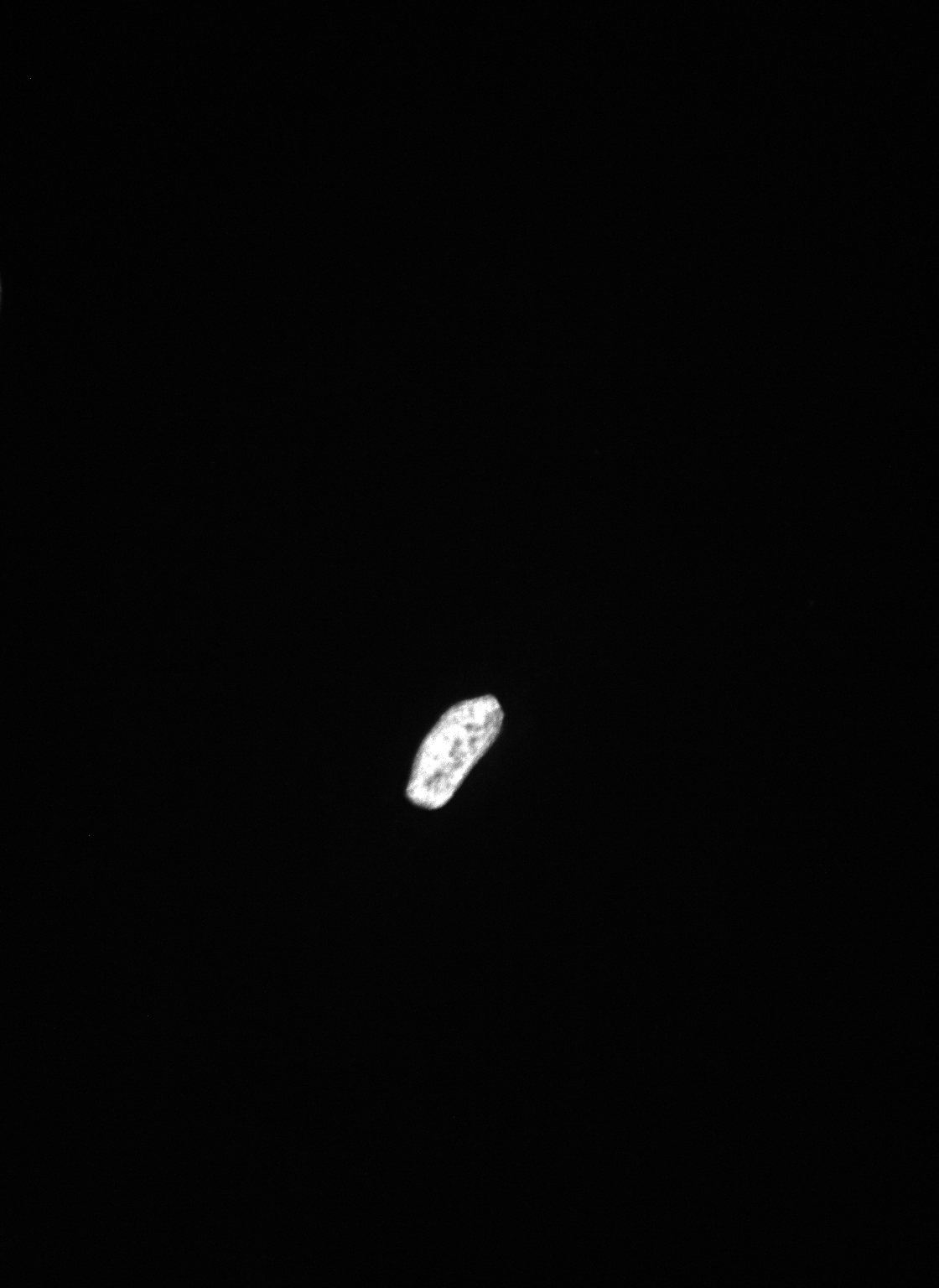

Supplement: Supplementary file 11 — Source Data for Figure 5 [file EMBJ-42-e112812-s010.zip › Figure 5 Source Data/Fig 5G image Tubacin.tif]

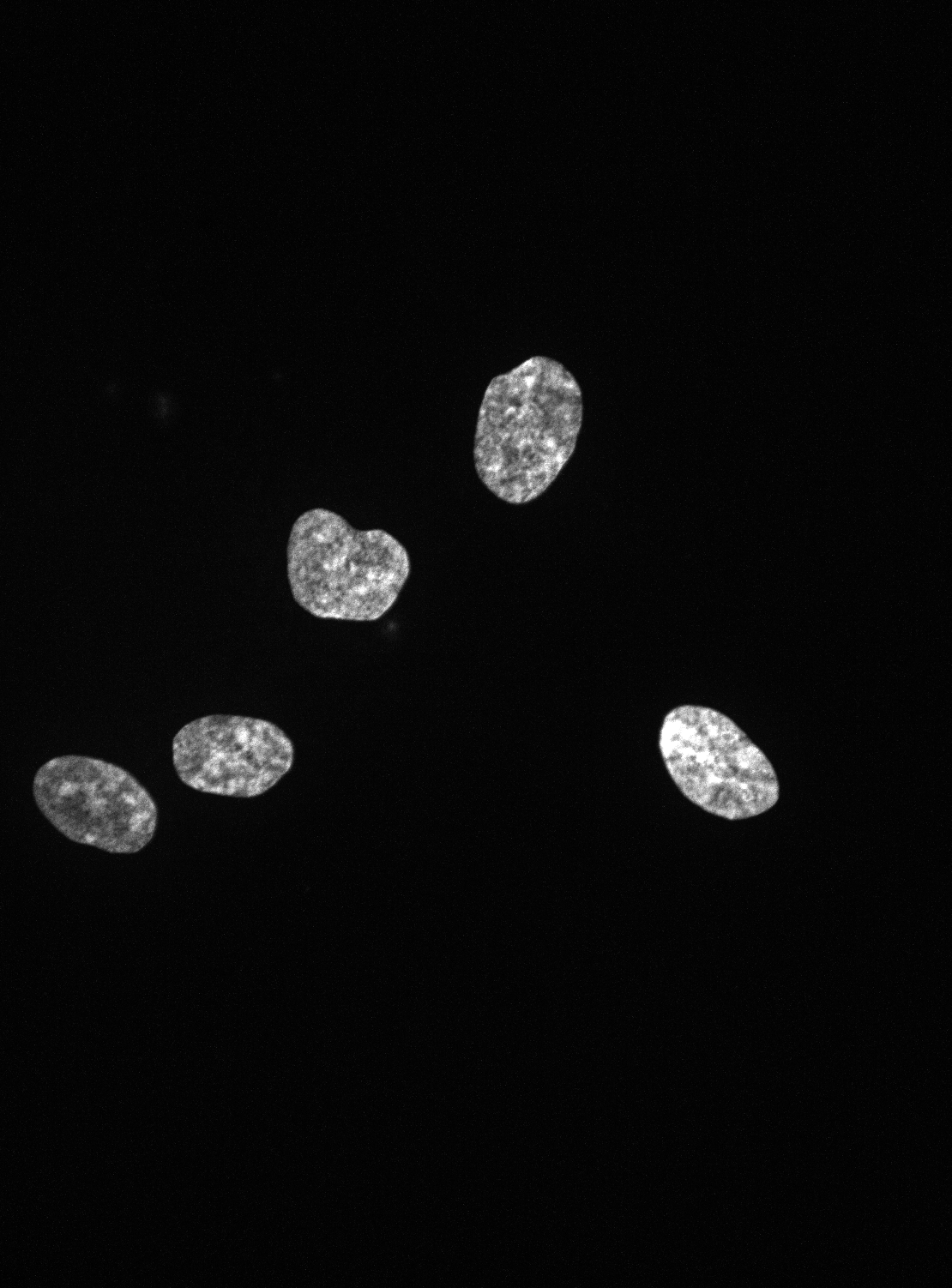

Supplement: Supplementary file 11 — Source Data for Figure 5 [file EMBJ-42-e112812-s010.zip › Figure 5 Source Data/Fig 5A image Ctr.tif]

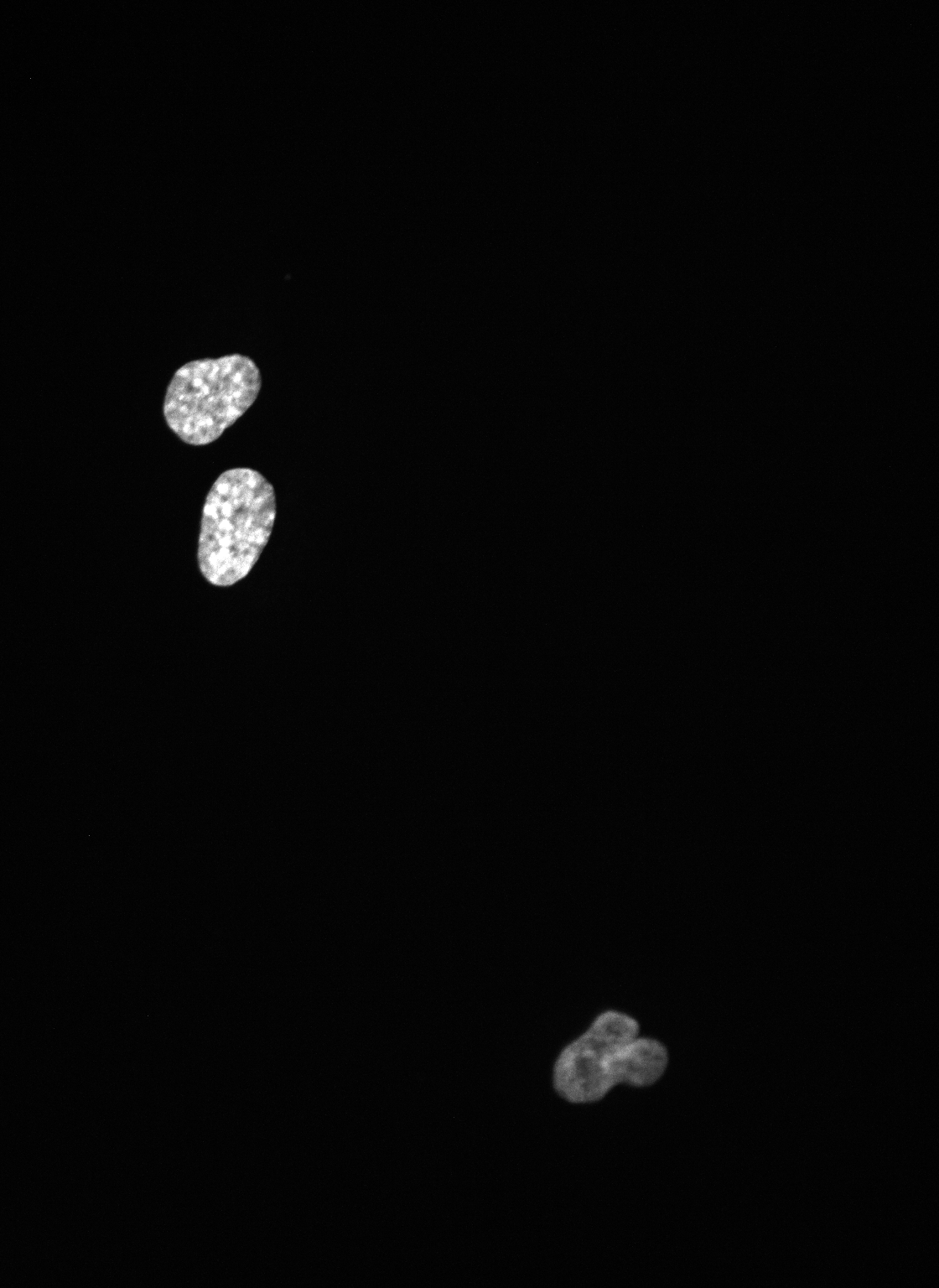

Supplement: Supplementary file 11 — Source Data for Figure 5 [file EMBJ-42-e112812-s010.zip › Figure 5 Source Data/Fig 5G image ATAT1OE.tif]

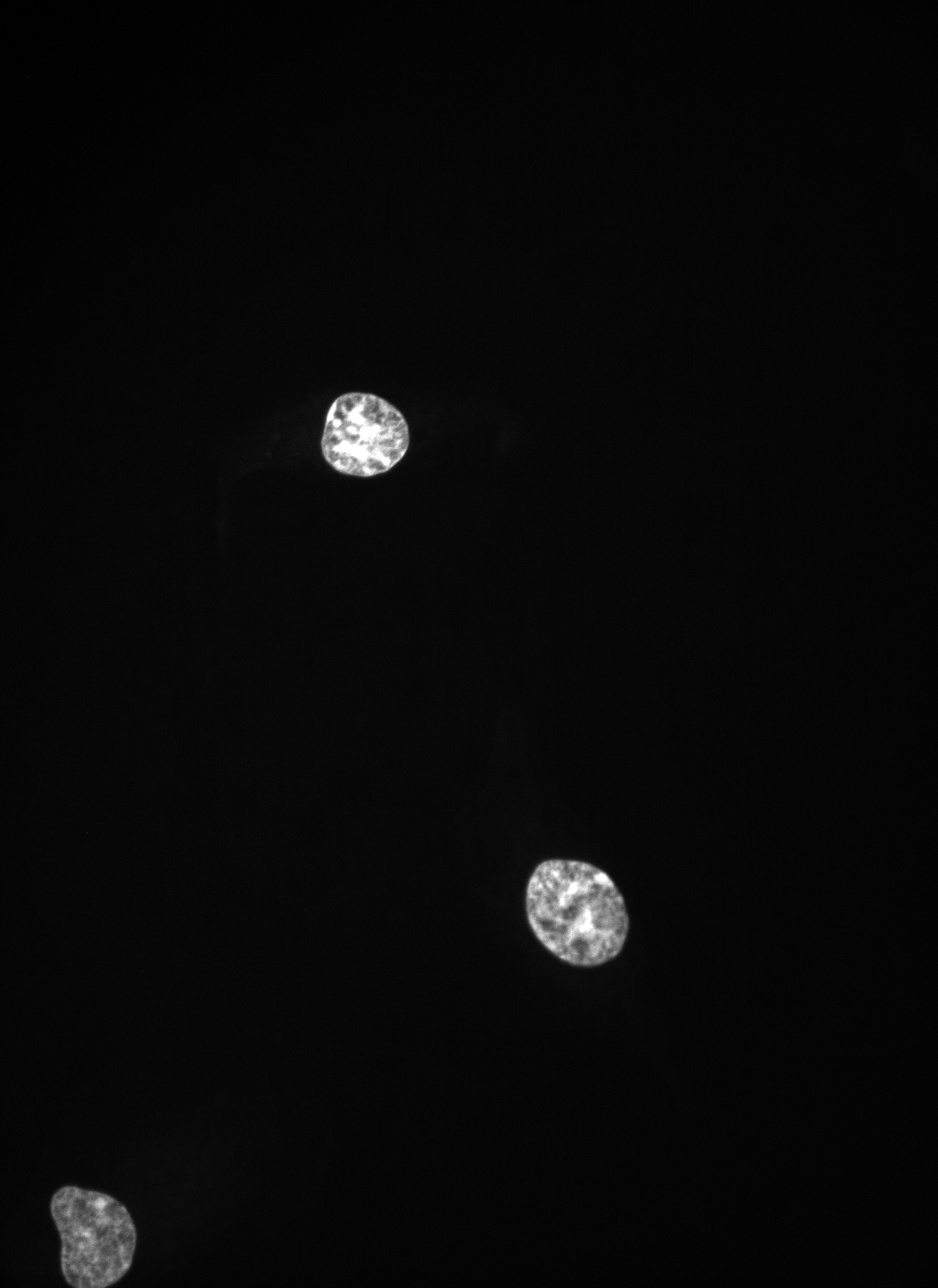

Supplement: Supplementary file 11 — Source Data for Figure 5 [file EMBJ-42-e112812-s010.zip › Figure 5 Source Data/Fig 5E image Ctr.tif]

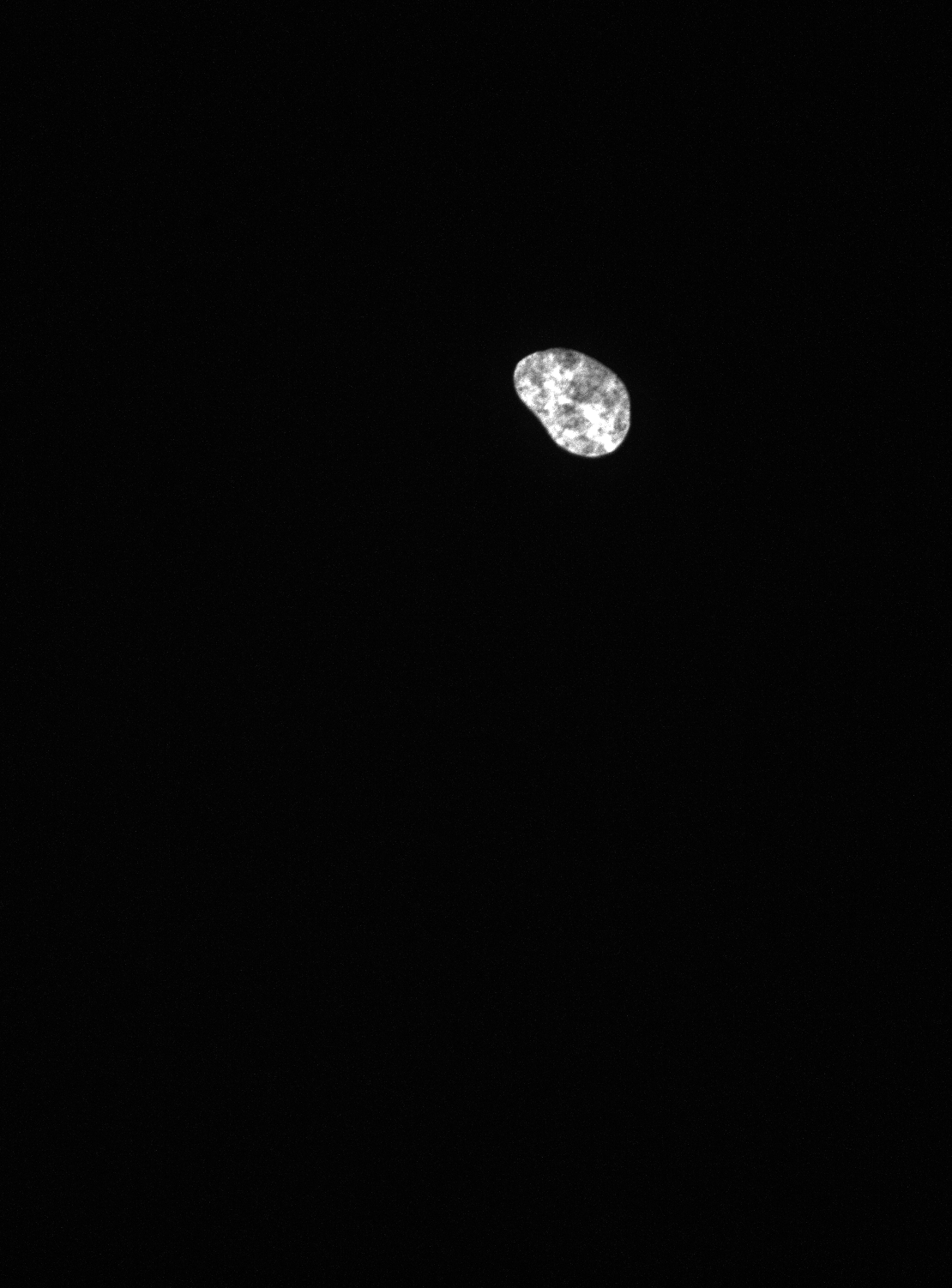

Supplement: Supplementary file 11 — Source Data for Figure 5 [file EMBJ-42-e112812-s010.zip › Figure 5 Source Data/Fig 5C image H2O2.tif]

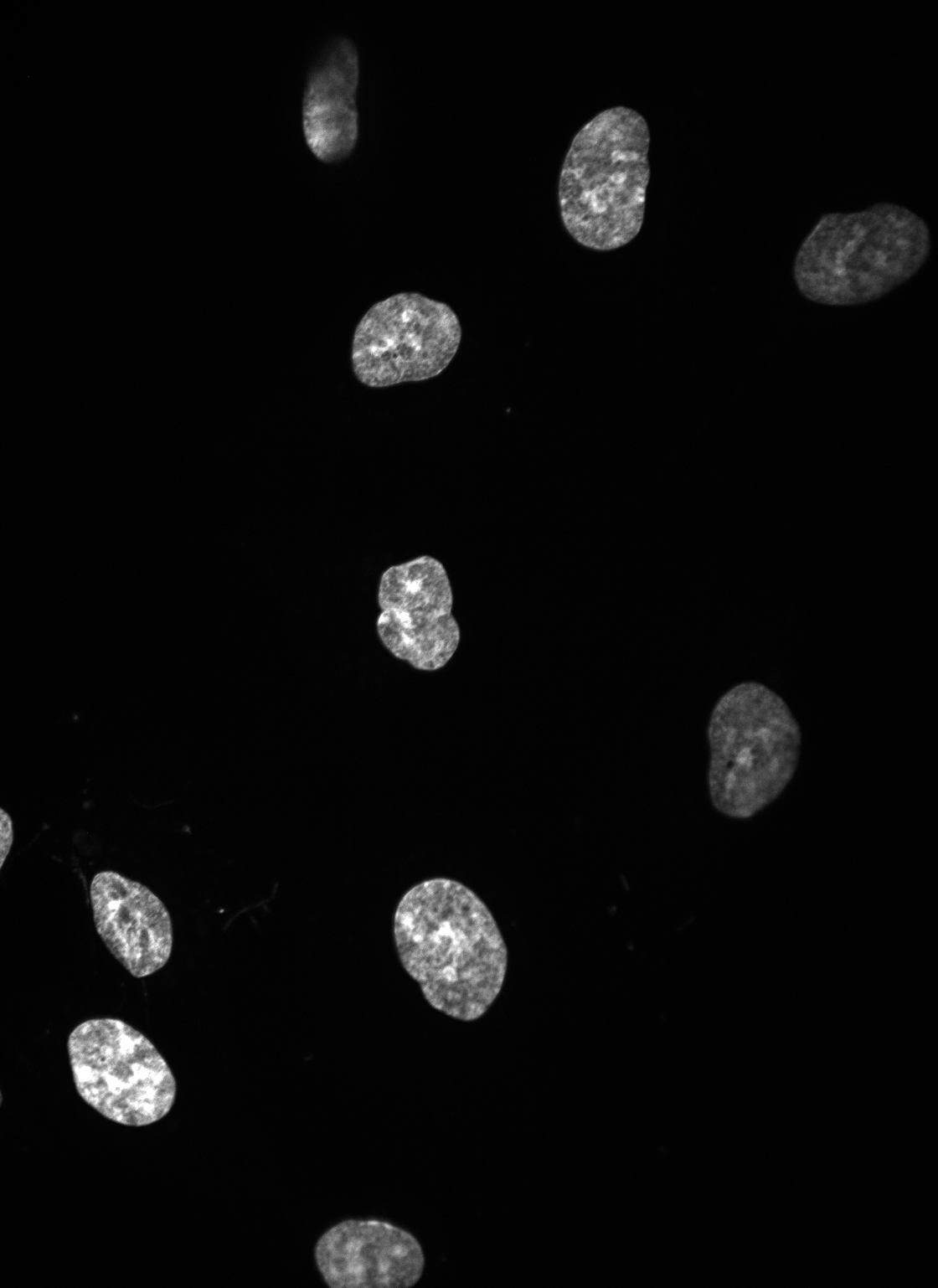

Supplement: Supplementary file 11 — Source Data for Figure 5 [file EMBJ-42-e112812-s010.zip › Figure 5 Source Data/Fig 5I image Tubacin.tif]

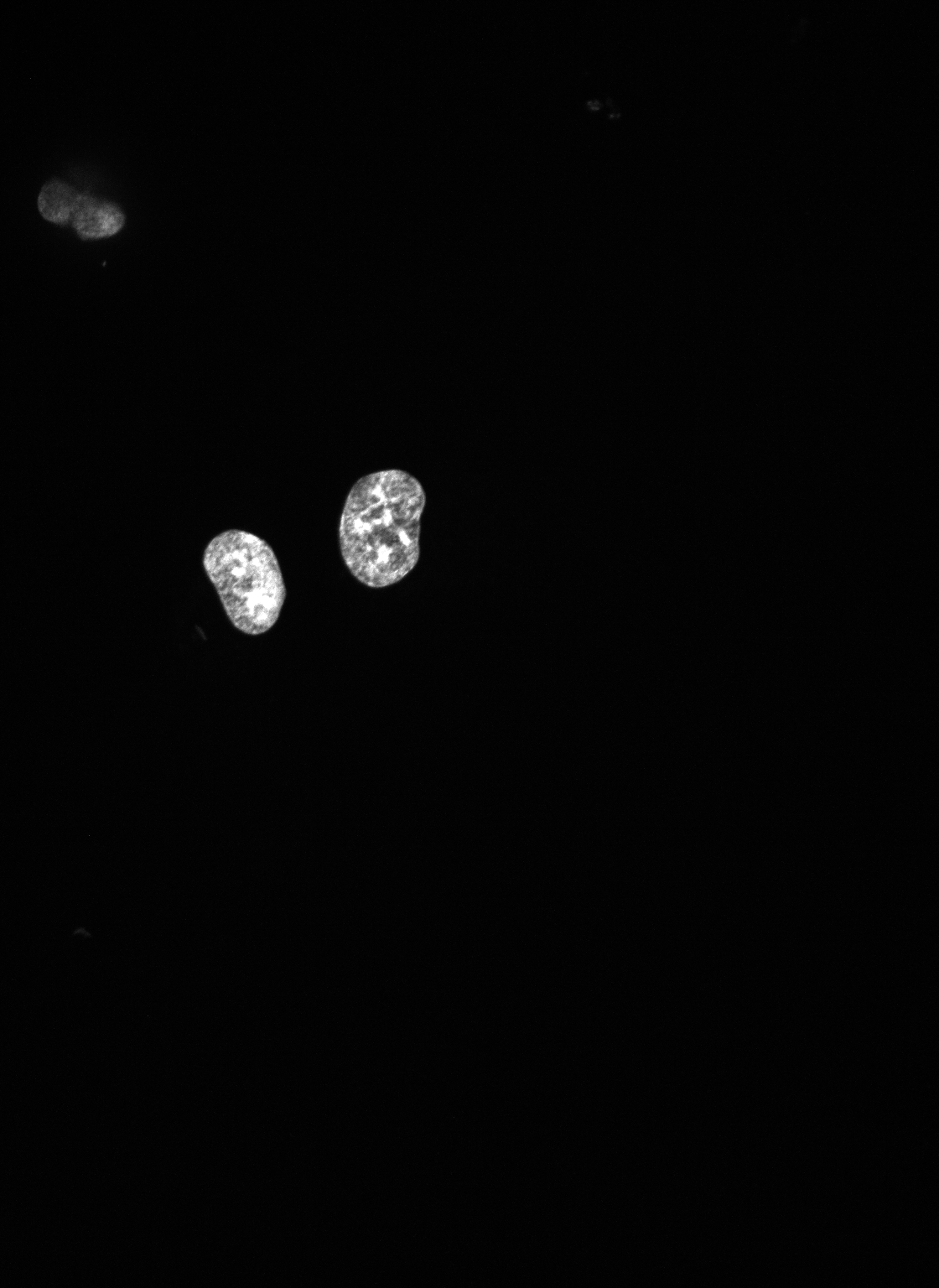

Supplement: Supplementary file 11 — Source Data for Figure 5 [file EMBJ-42-e112812-s010.zip › Figure 5 Source Data/Fig 5E image ATAT1OE.tif]

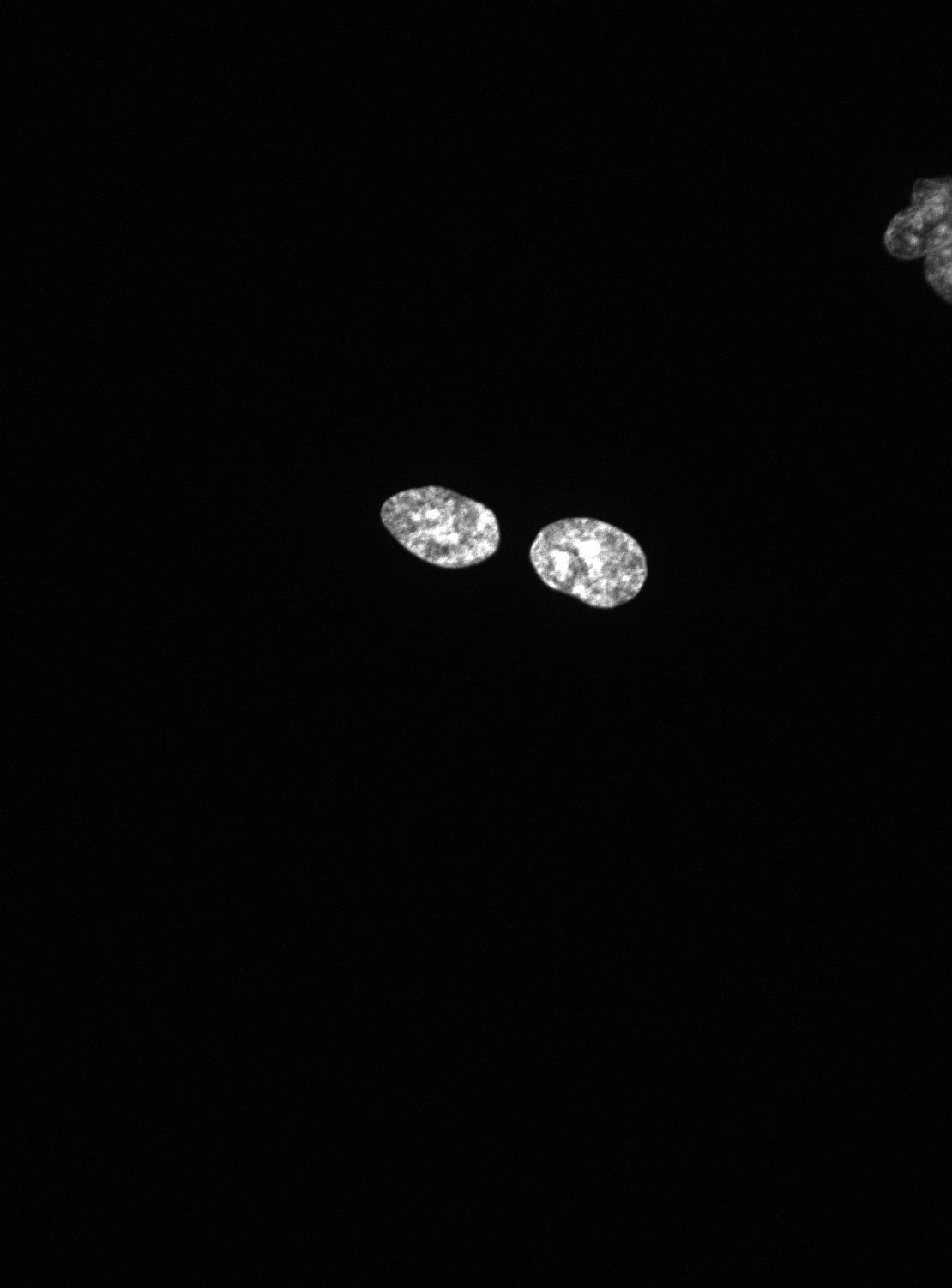

Supplement: Supplementary file 11 — Source Data for Figure 5 [file EMBJ-42-e112812-s010.zip › Figure 5 Source Data/Fig 5A image H2O2.tif]

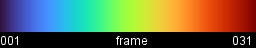

Supplement: Supplementary file 12 — Source Data for Figure 6 [file EMBJ-42-e112812-s001.zip › Figure 6 Source Data/Fig 6E color time scale.png]

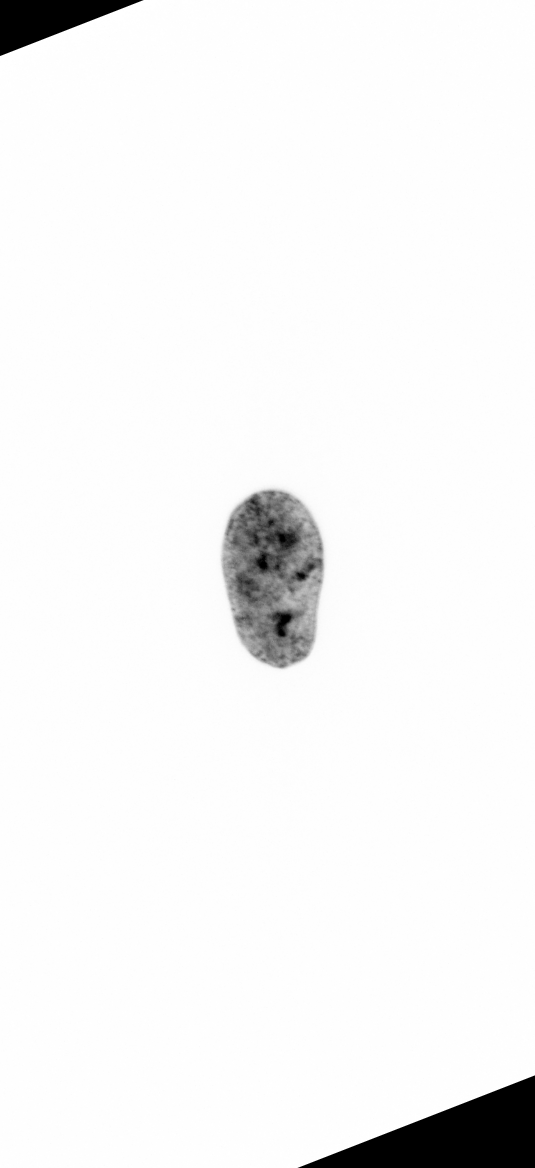

Supplement: Supplementary file 12 — Source Data for Figure 6 [file EMBJ-42-e112812-s001.zip › Figure 6 Source Data/Figure 6C image Tubacin.tif]

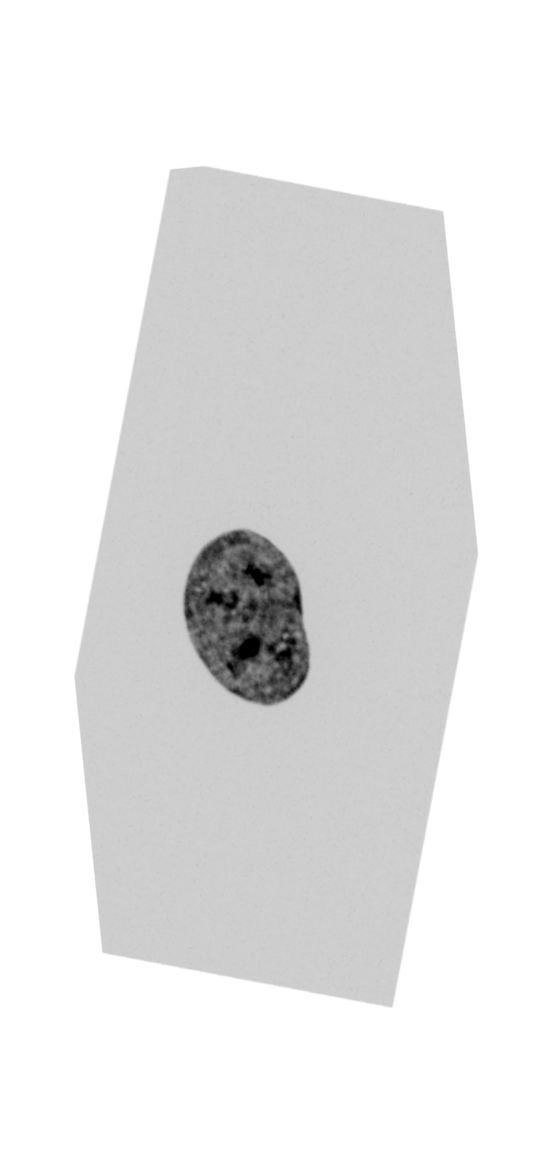

Supplement: Supplementary file 12 — Source Data for Figure 6 [file EMBJ-42-e112812-s001.zip › Figure 6 Source Data/Figure 6C image H2O2.tif]

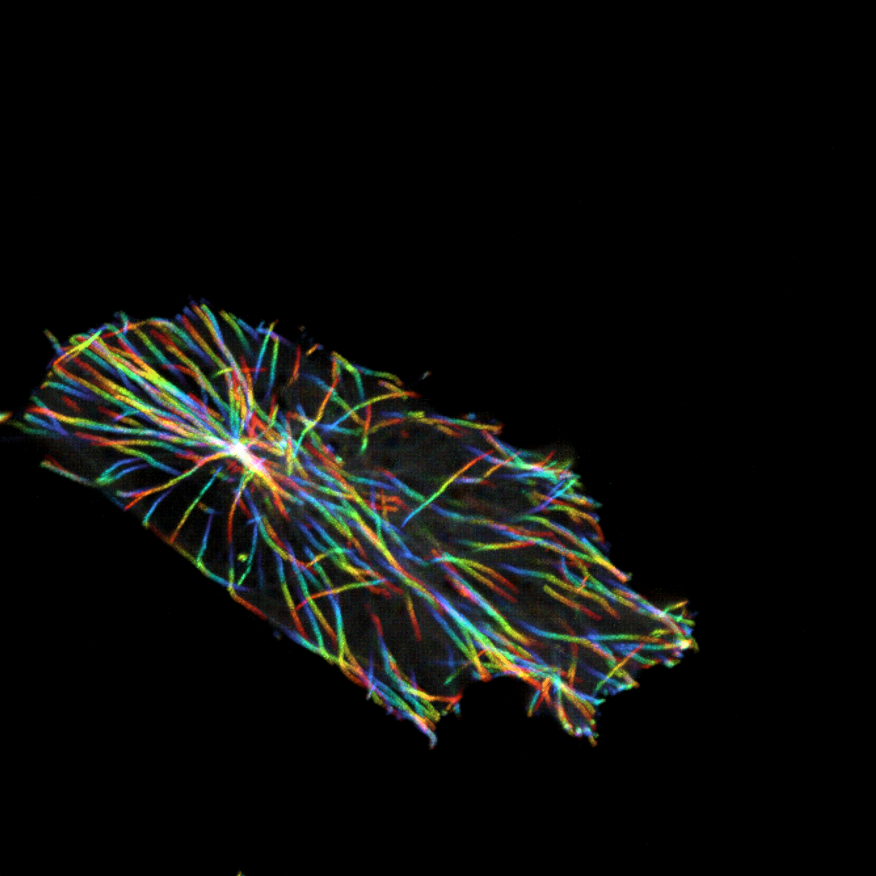

Supplement: Supplementary file 12 — Source Data for Figure 6 [file EMBJ-42-e112812-s001.zip › Figure 6 Source Data/Fig 6E image Tubacin.png]

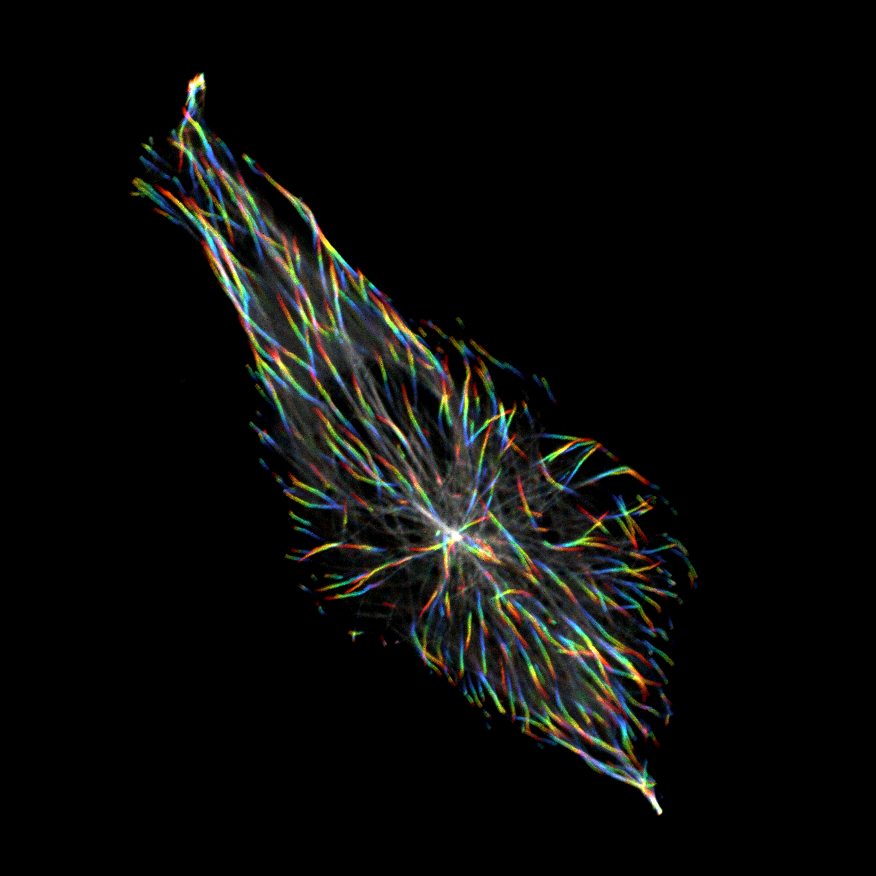

Supplement: Supplementary file 12 — Source Data for Figure 6 [file EMBJ-42-e112812-s001.zip › Figure 6 Source Data/Fig 6E image H2O2.png]

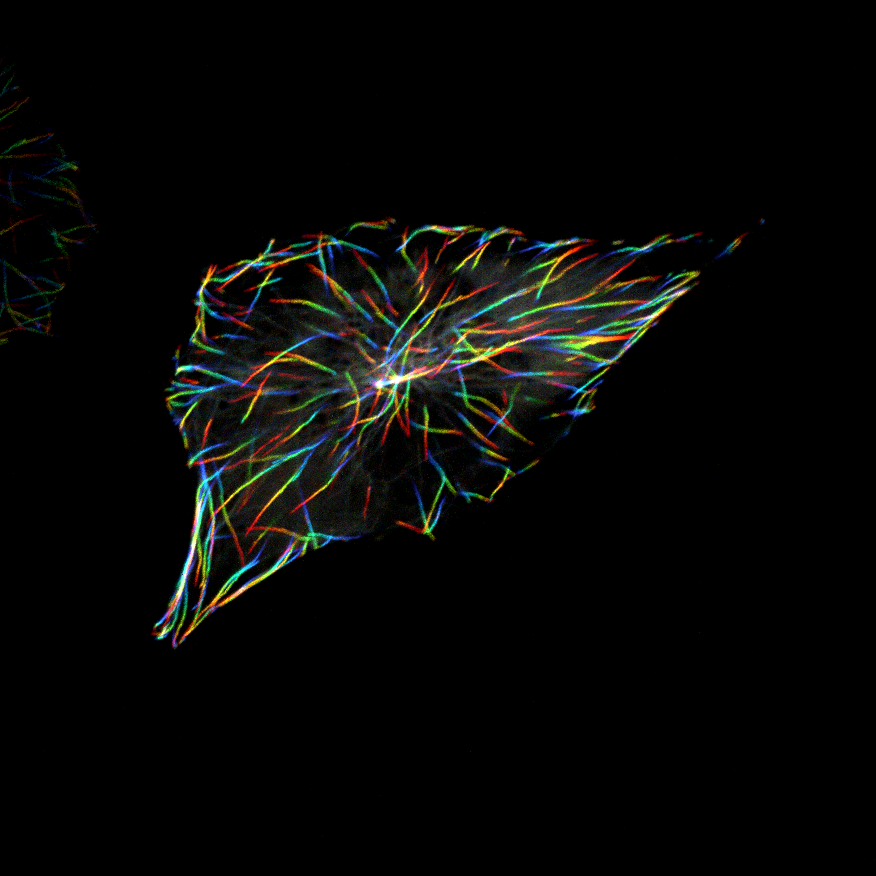

Supplement: Supplementary file 12 — Source Data for Figure 6 [file EMBJ-42-e112812-s001.zip › Figure 6 Source Data/Fig 6E image -DOX.png]

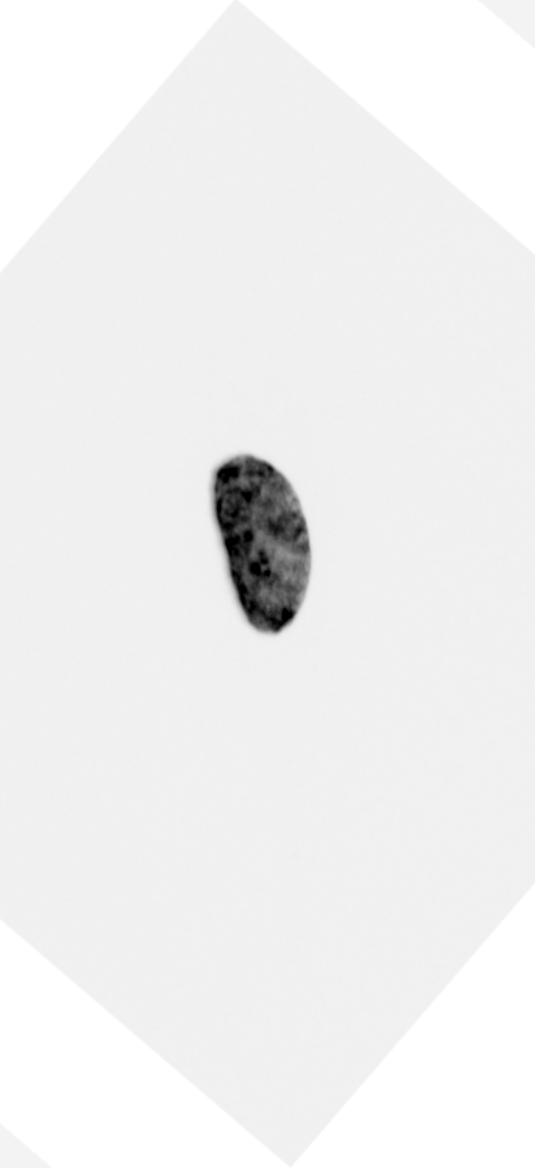

Supplement: Supplementary file 12 — Source Data for Figure 6 [file EMBJ-42-e112812-s001.zip › Figure 6 Source Data/Fig 6C image aTAT1OE.tif]

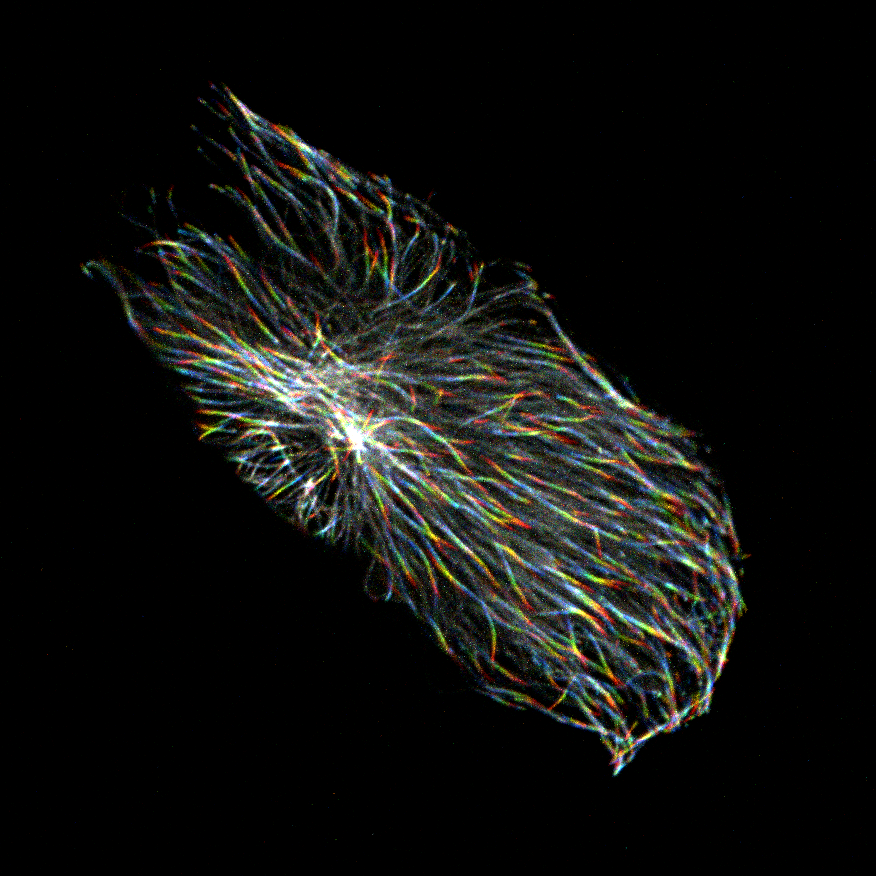

Supplement: Supplementary file 12 — Source Data for Figure 6 [file EMBJ-42-e112812-s001.zip › Figure 6 Source Data/Fig 6E imafe aTAT1 OE.png]

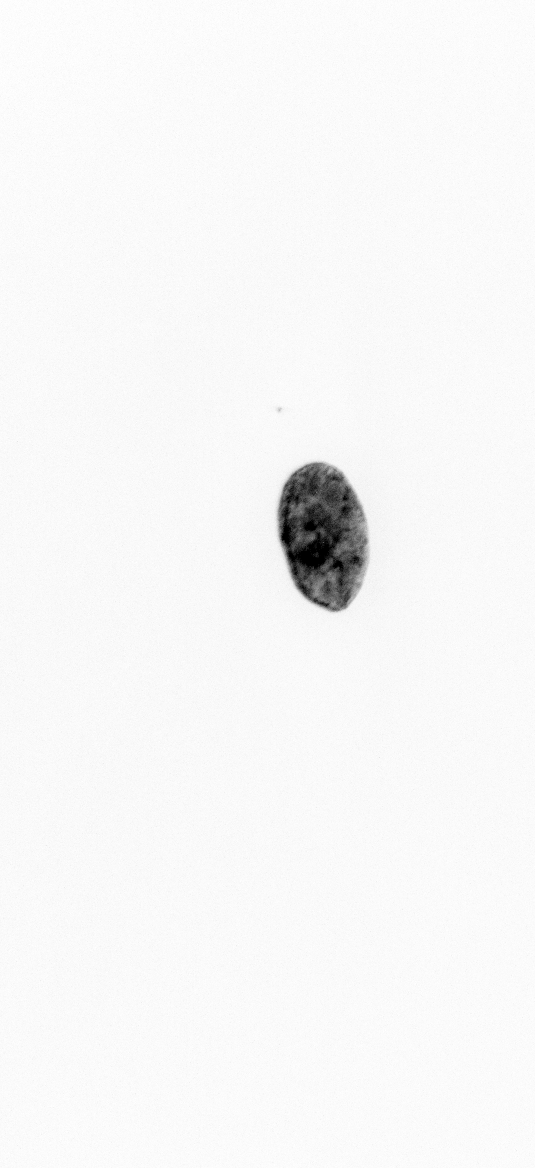

Supplement: Supplementary file 12 — Source Data for Figure 6 [file EMBJ-42-e112812-s001.zip › Figure 6 Source Data/Figure 6C image -DOX.tif]

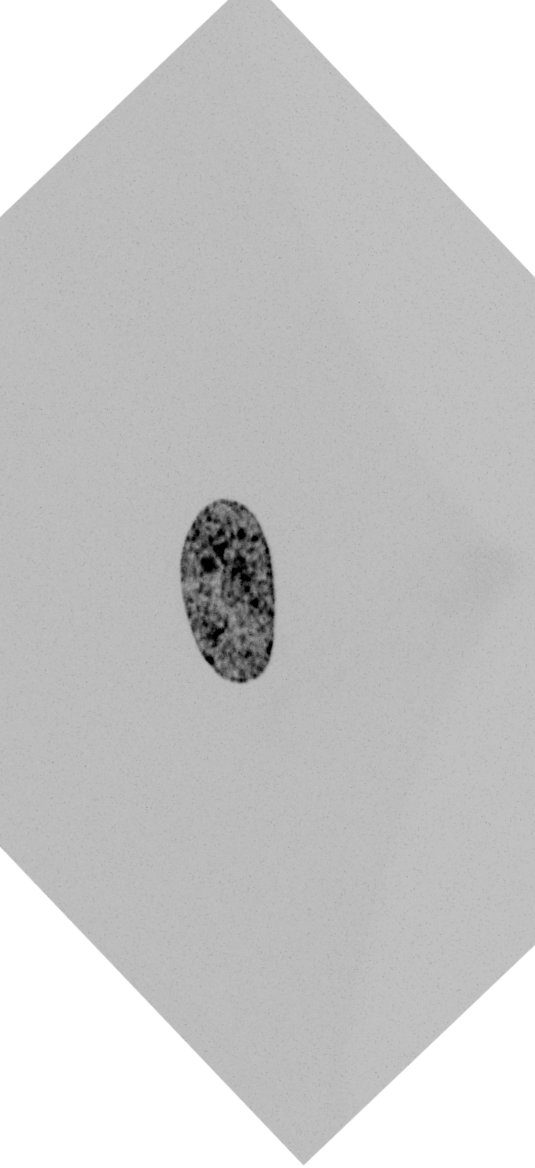

Supplement: Supplementary file 12 — Source Data for Figure 6 [file EMBJ-42-e112812-s001.zip › Figure 6 Source Data/Fig 6C image +DOX.tif]

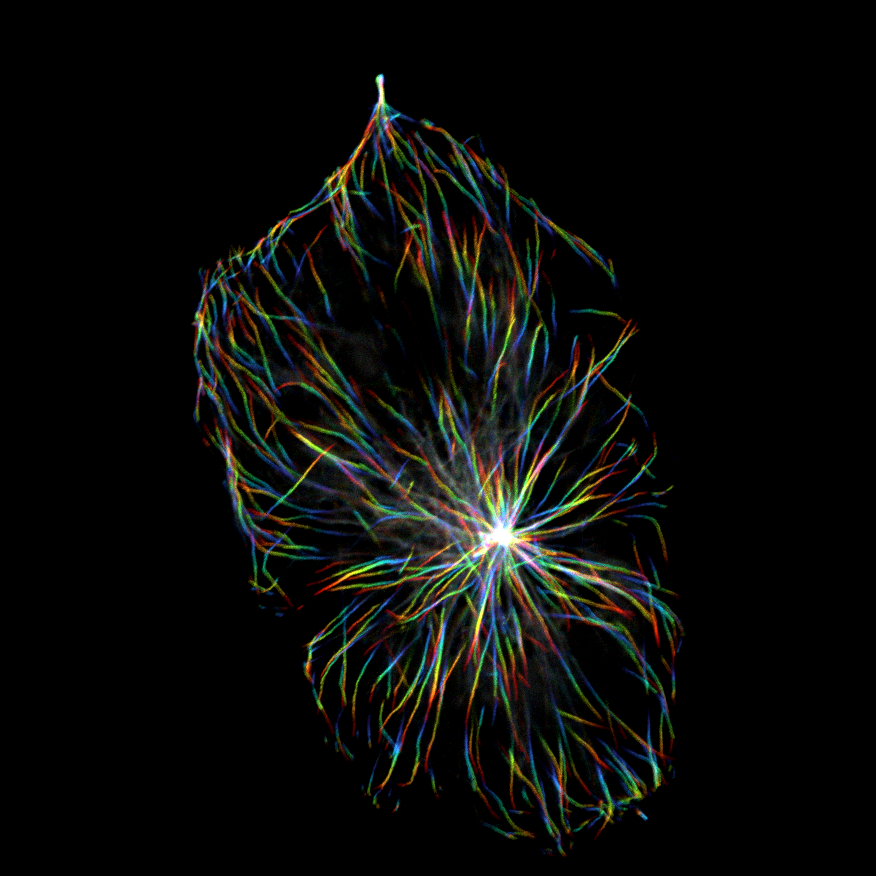

Supplement: Supplementary file 12 — Source Data for Figure 6 [file EMBJ-42-e112812-s001.zip › Figure 6 Source Data/Fig 6E image +DOX.png]

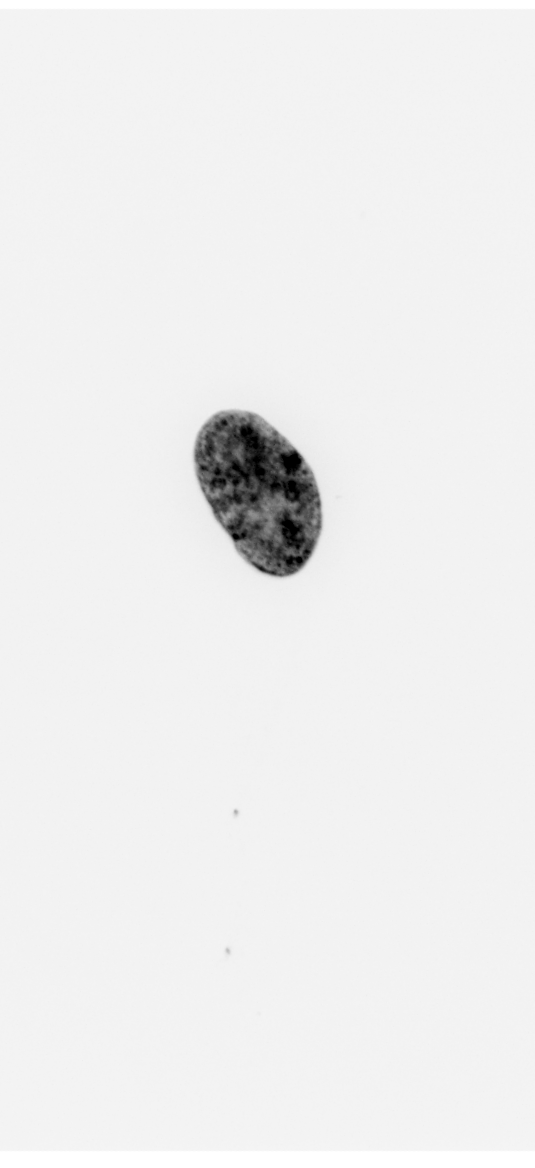

Supplement: Supplementary file 12 — Source Data for Figure 6 [file EMBJ-42-e112812-s001.zip › Figure 6 Source Data/Figure 6D +DOX+Tubacin.tif]

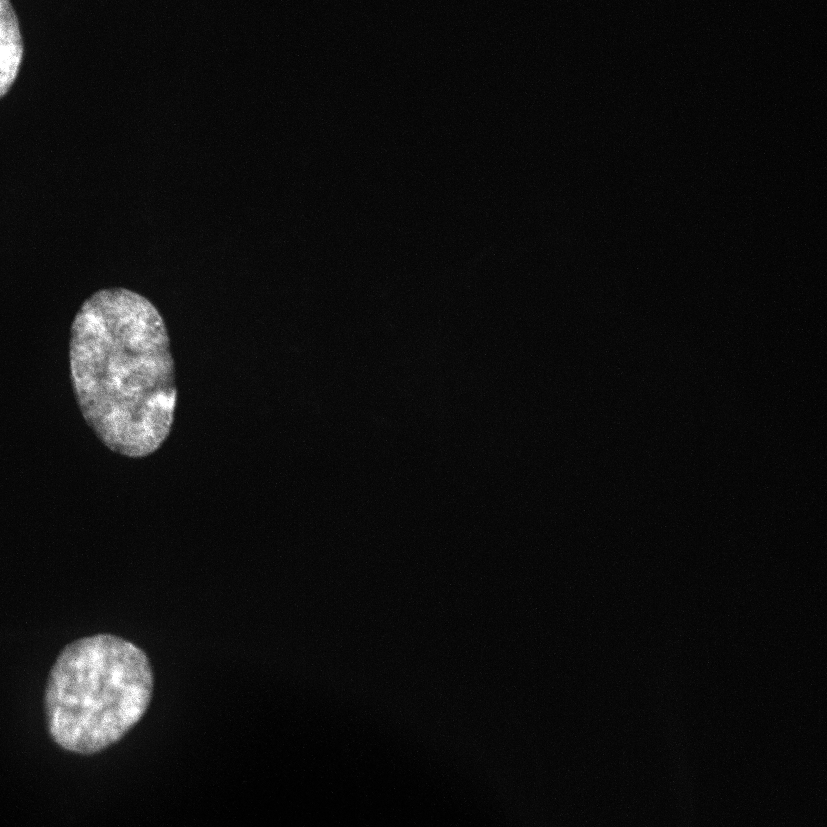

Supplement: Supplementary file 13 — Source Data for Figure 7 [file EMBJ-42-e112812-s002.zip › Figure 7 Source data/Figure 7G image +DOX+Tubacin.tif]

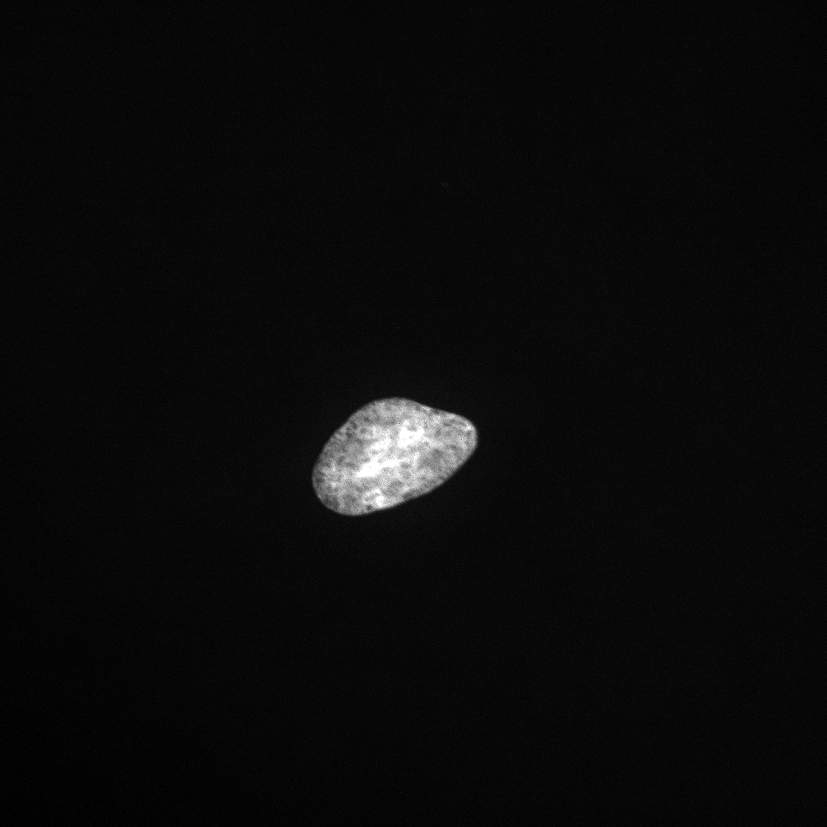

Supplement: Supplementary file 13 — Source Data for Figure 7 [file EMBJ-42-e112812-s002.zip › Figure 7 Source data/Figure 7G image -DOX.tif]

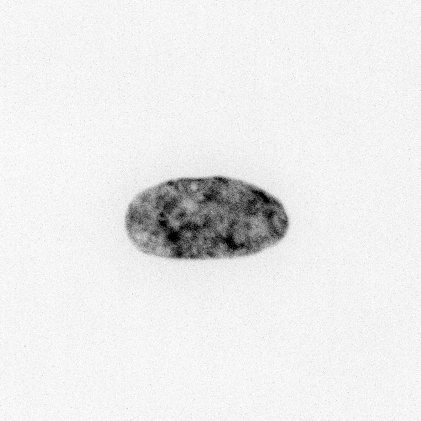

Supplement: Supplementary file 13 — Source Data for Figure 7 [file EMBJ-42-e112812-s002.zip › Figure 7 Source data/Figure 7G image +DOX.tif]
