# Supplementary material for: Centrosome amplification fine tunes tubulin acetylation to differentially control intracellular organization
Source: EMBO J. 2023 Jul 5;42(16):e112812. doi: 10.15252/embj.2022112812 (PMC10425843; doi:10.15252/embj.2022112812)
Supplement: Supplementary file 14 [file EMBJ-42-e112812-s008.pdf]

THE EMBO JOURNAL

Centrosome amplification fine-tunes tubulin acetylation to differentially control intracellular organization

Pedro Monteiro, Bongwhan Yeon, Samuel Wallis, and Susana Godinho
DOI: 10.15252/embj.2022112812

Corresponding author(s): Susana Godinho (s.godinho@qmul.ac.uk)

Review Timeline:

Submission Date:	14th Oct 22
Editorial Decision:	29th Nov 22
Revision Received:	26th Apr 23
Editorial Decision:	30th May 23
Revision Received:	5th Jun 23
Accepted:	14th Jun 23

Editor: Ieva Gailite

Transaction Report:

(Note: With the exception of the correction of typographical or spelling errors that could be a source of ambiguity, letters and reports are not edited. Depending on transfer agreements, referee reports obtained elsewhere may or may not be included in this compilation. Referee reports are anonymous unless the Referee chooses to sign their reports.)

1st Editorial Decision 29th Nov 2022

Dear Susana,

Thank you for submitting your manuscript to The EMBO Journal. I sincerely apologise for the protracted review process due to delayed submission of referee reports. We have now received comments from four reviewers, which are included below for your information.

As you will see from the reports, reviewers #2-#4 find the proposed mechanism of centrosome amplification-induced organelle displacement via regulation of microtubule acetylation interesting. However, reviewers #3 and #4 indicate several issues regarding the depth of analysis, the experimental approach and data analysis that need to be addressed before they can accept publication here. Reviewer #1 is more critical in their assessment, but their comments also highlight the need to further flesh out the reported findings.

In particular, reviewers #3 and #4 find that the contribution microtubule orientation and increased mass would need to be analysed in more detail, as well as request to test alternative approaches for increasing tubulin acetylation. Additionally, reviewers #1 and #3 raise concerns regarding the mode of action of the H2O2 bath treatment and the combined effect of DN KASH2 and centrosome amplification on centrosome attachment to the nucleus.

Based on the overall interest expressed by the reviewers #2-#4, I would like to invite you to address the comments of all reviewers in a revised version of the manuscript. I should add that it is The EMBO Journal policy to allow only a single major round of revision and that it is therefore important to resolve the main concerns at this stage. I think it would be useful to discuss the revision in more detail via email or phone/videoconferencing - please let me know which option you prefer.

We generally allow three months as standard revision time. As a matter of policy, competing manuscripts published during this period will not negatively impact on our assessment of the conceptual advance presented by your study. However, please contact me as soon as possible upon publication of any related work to discuss the appropriate course of action. Should you foresee a problem in meeting this three-month deadline, please let us know in advance to arrange an extension.

When preparing your letter of response to the referees' comments, please bear in mind that this will form part of the Review Process File and will therefore be available online to the community. For more details on our Transparent Editorial Process, please visit our website: https://www.embopress.org/page/journal/14602075/authorguide#transparentprocess. Please also see the attached instructions for further guidelines on preparation of the revised manuscript.

Please let me know if you have any further questions regarding the revision. Thank you for the opportunity to consider your work for publication, and I look forward to receiving the revised manuscript.

Best wishes,

Ieva

Ieva Gailite, PhD
Senior Scientific Editor
The EMBO Journal
Meyerhofstrasse 1
D-69117 Heidelberg
Tel: +4962218891309
i.gailite@embojournal.org

Instructions for preparing your revised manuscript:

Please make sure you upload a letter of response to the referees' comments together with the revised manuscript.

Please also check that the title and abstract of the manuscript are brief, yet explicit, even to non-specialists.

When assembling figures, please refer to our figure preparation guideline in order to ensure proper formatting and readability in print as well as on screen:
https://bit.ly/EMBOPressFigurePreparationGuideline
See also guidelines for figure legends: https://www.embopress.org/page/journal/14602075/authorguide#figureformat

At EMBO Press we ask authors to provide source data for the main manuscript figures. Our source data coordinator will contact

you to discuss which figure panels we would need source data for and will also provide you with helpful tips on how to upload and organize the files.

IMPORTANT: When you send the revision we will require
- a point-by-point response to the referees' comments, with a detailed description of the changes made (as a word file).
- a word file of the manuscript text.
- individual production quality figure files (one file per figure)
- a complete author checklist, which you can download from our author guidelines (https://www.embopress.org/page/journal/14602075/authorguide).
- Expanded View files (replacing Supplementary Information)
Please see out instructions to authors
https://www.embopress.org/page/journal/14602075/authorguide#expandedview

Please remember: Digital image enhancement is acceptable practice, as long as it accurately represents the original data and conforms to community standards. If a figure has been subjected to significant electronic manipulation, this must be noted in the figure legend or in the 'Materials and Methods' section. The editors reserve the right to request original versions of figures and the original images that were used to assemble the figure.

Further information is available in our Guide For Authors: https://www.embopress.org/page/journal/14602075/authorguide

We realize that it is difficult to revise to a specific deadline. In the interest of protecting the conceptual advance provided by the work, we recommend a revision within 3 months (27th Feb 2023). Please discuss the revision progress ahead of this time with the editor if you require more time to complete the revisions.

Referee #1:

The authors investigate the polar organisation of cells treated with a PLK4 inhibitor, which results in centrosomal duplication and find that treated cells translocate their centrosomes (and associated with it a number of organelles) from an area in nuclear proximity to the cell periphery. To understand the mechanism underlying this phenomenon, they follow the concept of a push-pull balance, where the centrosome shifts between perinuclear and peripheral based on a balance between kinesin 1 driven pushing and dynein driven pulling forces. genetic and pharmacological experiments support this idea. The authors then move to the role of microtubule modification in this phenomenon and find that cells with multiplied centrosomes have higher levels of acetylation and increased levels of tubulin. Enhanced acetylation explains some but not other organelle displacement phenotypes that were previously described, potentially linking acetylation and kinesin activity. Finally the authors link the observed phenotypes to altered vimentin distribution and enhanced nuclear deformability, which in turn affects migration in 3D environments.

The main problem with this manuscript is that despite multiple rounds of reading, I cannot understand the causal logic that leads from one experiment to the other. The readout of the experiments is a very crude quantification of organelle or centrosome positioning, while the manipulations are also very generic. The experiments no not directly address the mechanistic basis of the cell biological process.
Some major issues:

1) It is not clear to me how the push-pull concept of centrosome positioning is directly supported by the experiment when the authors disengage microtubules and nucleus using the dominant negative kASH approach. The authors do not even provide a mechanistic framework how this result could support the conclusions. Importantly, no measurements, like e.g. laser ablations, are performed to challenge the concept.
2) The authors take a large leap from centrosome duplications to microtubule acetylation and this step is motivated by the observation that there is more acetylation (but also more tubulin) in the duplicated setting. I cannot understand what justifies this step and how the two phenomena are interlinked. The idea that kinesins mediate this effect is plausible but not directly supported by experiments.
3) Another large leap is the manipulation of acetylation using H_2O_2. This is a completely unspecific approach, which does not allow any conclusions.
4) The obervation that some but not other organelles are influenced by acetylation is interesting but not followed up at all.
5) the link between acetylation, intermediate filaments and nuclear deformability is again not directly addressed.

Referee #2:

This is a very interesting and well-executed paper to study the effects of increased centriole numbers upon cytoskeletal events in RPE-1 cells resulting from elevated levels of Plk4.
The authors find that the amplified centrioles are displaced to wards the periphery of the cell in a manner that requires KIF5B and is associated with an increased acetylation of tubulin. Indeed, depletion of the aTAT1 acetyl transferase prevents the displacement of centrosomes and other organelles, with the exception o the Golgi and endosomes. Finally cells with displaced centrosomes display increased ability of their nuclei to become deformed, facilitating cell migration through small pores.
These are interesting observations, particularly in light of tumor cells to have supernumerary centrosomes. The conclusions are adequately supported by the experimentation and I recommend publication in EMBOJ without the need for revision.

Referee #3:

In this manuscript, Monteiro and co-authors show that centrosome duplication results in a change of organelle positioning and increased levels of K40 acetylated microtubules. The interplay of kinesin-1 and dynein motor complex seem to be important for the organelle displacement. Changing the level of microtubule acetylation by knockdown of αTAT1 or Tubacin treatment had an effect on several organelles and subcellular structures whereas others were not affected. The duplication of centrosomes and organelle displacement influenced nuclear shape and deformability leading to facilitated migration of cells through confined spaces. Altogether the authors propose an interesting process of deregulated organelle localization due to centrosome duplication which is especially interesting in the context of cancer and metastasis. This is a well performed study which in my opinion will be suitable for publication after addressing several points:

Major points:
1. Abstract: "Depletion of a-tubulin acetyltransferase 1 (aTAT1) to block tubulin acetylation, which has no impact on control cells, rescues the displacement of centrosomes, mitochondria and vimentin, but not Golgi or endosomes." In the abstract it is not clear what on what exactly depletion of ATAT1 has no effect. Please rephrase and clarify.

2. Figure 2: In panels I and J the distribution of vimentin is measured in a 3D environment. However in 3D distance is not only in x,y direction. Has the distance in z direction also been considered?

3. Figure 5: The authors show that some organelles are displaced by increased microtubule acetylation independent of centrosome duplication. However, there is no analysis of Tubacin treatment/αTAT1 overexpression vs PLK4 overexpression and therefore centrosome duplication. In order to show that the effects are comparable a quantification of the displacement between these two ways to induce organelle displacement would be beneficial.

4. Figure 6: What happen to the MT polarity (+end, -end localisation)? This would give further information regarding force generation by kinesin and dynein motors and net force outcome.

5. The authors show that increasing tubulin acetylation by overexpression of ATA1 is sufficient to promote organelle displacement independently of amplified centrosomes. I am wondering if increasing acetylation for example by treatment with taxon would lead to a similar outcome.

6. Line 153-156: The authors conclude that after dominant-negative GFPKASH2 expression the interaction of the centrosome with the nucleus is disrupted. The combination of centrosome duplication and dominant-negative GFPKASH2 expression leads to even greater displacement. This raises the question if the centrosomes can still be somehow attached to the nucleus if they are displaced and multiple µm away from the nucleus (Fig. EV1H,I).

7. Lines 356-359: The authors state that they see a polarized distribution of acetylated MTs upon treatment with hydrogen peroxide. On page 8 they state that hydrogen peroxide damages the microtubule lattice which leads to increased acetylation. This raises the question how a bath application of hydrogen peroxide can lead to a polarized distribution of microtubule acetylation which is not addressed by the authors.

8. Statistical analysis: It is not stated how exactly statistical tests are performed. Are they performed on all values or on the means of individual experiments? This is also not clear because in the graphs all single values as well as means of individual experiments are shown. Also, often student's t-tests are used and no information about normal distribution are stated.

Minor points:
9. In some figure panels two channels are both shown in grey scale on the same image. Better use different colours.
10. It it better to be consistent with the colours used for the representative images. For example, in Fig. 2 panel F in the upper pictures microtubules are shown in grey and vimentin in orange. In the lower pictures vimentin is shown in grey. In Fig. 4 panel C in the upper pictures microtubules are shown in grey and vimentin in orange. In the lower pictures vimentin is shown in grey, which is confusing.

11. Line 129-133: Claims about the centrosome nucleus distance due to nocodazole and latrunculin A treatment are made. However, statistical analysis for this is missing. Comparison -Dox, -Noc, -LatA vs -Dox, +Noc/+LatA (Fig. EV1C,D).
12. Statistics for centrosome displacement in control cells after KIF5B or p150 depletion is missing. Comparison of -Dox, -siKIF5B/-sip150 vs -Dox, + siKIF5B/+sip150 (Fig. 1G).
13. Line 175-176: The statistics for the comparison of p150 depletion in control cells compared to doxycyclin treated cells are missing.
14. Line205: figure mentioned should be Fig. 3E,F not EV3
15. Lines 354-355: Please explain to what the levels of acetylated microtubules was compared to.
16. Centrin 2 antibody is missing in the antibody table.
17. Fig. 2: In figure legend for panel C there are words missing (...upon induction of PLK4...). For panel C are the n numbers endosomes or cells?
18. Fig. 3: In panel A the Ac-tubulin picture is shifted down compared to merged picture. Panels I and J are mentioned in the text before E-H (line 197). In panel D statistics are missing. In panels F, G, H the y-axis legend is incomplete (relative intensity?). In figure legend for panel H the word intensity is missing. In description of statistical tests there is a typo: there is no panel K it should be H.
19. Fig. 4: Which siRNA for αTAT1 was used in the representative pictures (panel A, C, E)? In panel D the statistic for siCtr comparison of +Dox and -Dox is missing.
20. Fig. 6: Are the n numbers for this figure cells or microtubules? Please indicate in figure legend
21. Fig. EV1: Statistics for panel I are missing.
22. Fig. EV3: C and D are not mentioned in the manuscript. Please explain what DCF is doing.
23. Fig. EV4: Which siRNA for αTAT1 was used in the representative pictures (panel C, E, G)?
It would be better to keep consistent with the colours used for the representative images. For example, in panel C in the upper pictures EEA1 is shown in orange and F-actin in grey. In the lower pictures EEA1 and DNA is shown in grey, which is confusing. Figure legend for panel C: Is the n number cells or endosomes?
24. Fig. EV5: In panel E the authors show the increased MT acetylation upon Tubacin treatment and αTAT1 overexpression. However, they only show one cell for each condition. A bigger field of view with multiple cells or quantification of the increase would be more convincing. Panel F is not mentioned in the text.

Referee #4:

In this manuscript Monteiro and colleagues demonstrate that overamplification of centrosomes leads to reorganization of organelle positioning inside cells, and that this process is dependent on kinesin-1-mediated transport and polarized distribution of acetylated microtubules towards the cell periphery.

The authors perform a systematic and carefully executed study on the role of centrosome amplification, induced by the overexpression of Plk4, on the re-organisation of the intracellular space. They first demonstrate that centrosome amplification leads to the displacement of the centrosomes in a kinesin-1-depended manner, and that this process induces a global intracellular re-organisation of different organelles. Next, they show that cells with extra centrosomes have increased levels of alpha-tubulin acetylation, suggesting that perhaps this change in microtubule modification could be the cause of the observed re-organisation of the intracellular space. The authors test this hypothesis by experimentally modulating the levels of tubulin acetylation and indeed show that they can reproduce most, however, not all of the re-organisation events induced by centrosome amplification. They also follow up a previous observation that oxidative damage increases tubulin acetylation, and demonstrate that accordingly, applying oxidative damage to cells is sufficient to induce intracellular reorganisation. Because increase of tubulin acetylation alone was not sufficient to induce displacement of centrosomes (while it did induce the reorganisation of most other intracellular components), the authors performed a detailed image analyses of the cells to determine how polarity of acetylation patters could explain the observed effects. Finally, the authors show that following global organelle re-distribution, the nucleus tolerates higher degrees of deformation, thus giving the cells an increased ability to migrate through narrow spaces.

This is a carefully executed, and well-documented study. The manuscript is well-written, and figures are of high quality. In the introduction and discussion section the authors did a great job in introducing the relevant literature, and to thoughtfully discuss their findings in the light of this literature. The study provides a systematic characterisation of the consequences of centrosome overamplification on intracellular organization of organelles, supported by well-executed experimental procedures. The key finding is that tubulin acetylation, a so-far functionally understudied posttranslational modification of microtubules, is induced by centrosome overamplification, and mediates intracellular re-organisation. It is thus an important step toward the functional understanding of the physiological roles of tubulin modification. However, some aspects remain rather descriptive and do not propose a deeper mechanistic explanation of the links between the different phenotypes, for example how tubulin acetylation levels could be modulated by the centrosome amplification. Nevertheless, the findings reported in this manuscript contribute important novel insight into microtubule-based mechanisms of intracellular organisation, which will be key for future research on migration of cancer cells with supernumerary centrosomes. A revised version of this manuscript would thus be a strong candidate for publication in EMBO J.

Major points:
1. Does PLK4-induced centrosome overamplification lead to abnormalities in nucleus shape or presence of micronuclei due to mitotic defects? If yes, were such cells included in the analysis (e.g. for determining nuclear circularity?)

2. Fig. EV1H,I: the authors should test the possibility that the observable further displacement of the centrosome upon detachment of the nucleus could be explained with the greater mass of microtubules, which, as they show, results from having multiple MTOCs? This should be quantified. The authors should also check whether Kif5B levels in cells with normal and supernumerary centrosomes are similar.

3. For the vimentin displacement analysis, was the cell shape taken into account? This must be clarified as in the less polarized cells the distance between the nucleus and the leading edge can be rather small, resulting in overlap in the measured signal. Also, was the overall mass of vimentin different between the conditions, and/or did it correlate to the microtubule mass)?

4. Fig. 3D and EV3B: The authors may consider swapping Fig. 3D and Fig. EV3B. It appears that the current Fig. EV3B provides a stronger support for their conclusion that the distribution of acetylated tubulin does not decrease as dramatically in cells with overamplified centrosomes with increasing distance from the nucleus. In the legend of EV3B the authors should also include information on how many cells, and which regions were analysed for the generation of the curve.
The authors should also verify whether the total tubulin signal increases or remains similar in both conditions, and in the different regions of the cell.

5. Fig. 3J: it appears that the number of individual microtubules was counted. This is not a good way of doing these analyses for several reasons. First, how are single microtubules distinguished in images like those in the bottom row of Fig.3I (+DOX, +Noc), where the network is dense? Secondly, and most importantly, how is microtubule length taken into account? A potential solution is to either quantify microtubule mass, or microtubule length (summing up the length of all microtubules in one cell).

6. Does the analysis of acetylation orientation consider the irregularities in cell shapes, and how much space is there in the rear/front of the cell, especially in the conditions where acetylated tubulin is accumulated? An analysis of acetylated tubulin distribution normalized to total tubulin distribution could reveal if there is any bias in the orientation of the former towards the leading edge.
It is also not clear whether the orientation of the microtubules in respect to their minus- to plus-ends is analysed, or whether only the distribution of the acetylation signal throughout the cell is taken into account.
Another concern in this analysis is that between the normal (-DOX) and increased-acetylation (Tubacin and ATAT1 overexpression) conditions, the amount of acetylated microtubules differs greatly. This may conceal the effects the authors aim to determine, and therefore not allow to draw meaningful conclusions about the polarization of the signal. An additional control the authors may consider to add is overexpression of ATAT1, or Tubacin treatment in cells with amplified centrosomes to check whether this leads to polarization of the highly acetylated microtubule network.

7. Does H_2O_2 treatment in cells with normal centrosomes also lead to higher nucleus deformability and ability to migrate through small pores? From the current data it seems to be the condition that most faithfully replicates the effects of overamplified centrosomes by increasing acetylated tubulin in the orientation of the leading edge, as well as by inducing the displacement of vimentin. Showing that H_2O_2 treatment leads to increased deformability of the nucleus would thus strengthen the author's hypothesis that vimentin displacement contributes to this phenotype.

Minor comments:

1. There are a couple of instances where the authors make imprecise statements regarding the existing literature. This should be carefully revised:
- in the abstract: "Microtubule posttranslational modifications (PTMs) contribute to microtubule diversity and differentially regulate motor-mediated transport." - it would be more appropriate to say "can regulate..." as there is only sparse evidence for this so far.
- The authors write at several instances that acetylation is known to affect kinesin-1 transport. They should make sure to specify that this was observed in one few studies in cells, while in vitro, kinesin-1 was not sensitive to tubulin acetylation. In general, how tubulin acetylation affects kinesin-1 mediated transport remains a controversial issue, which should be clearly stated and discussed. (in abstract, introduction and discussion at different instances)
- line 76: "...PTMs, have been shown to differentially regulate several molecular motors..." this statement should be toned down, and it should be explained that the experiments were done with chemically modified yeast tubulin.

2. In the abstract, the authors propose that "that each organelle must have its own sensing and response mechanism". This is a long shot and even as a hypothesis a bit of an overstatement given the data they present in their paper. They should tone down.

3. Throughout the manuscript, the authors should verify the correct wording of tubulin isotypes (they wrote at several instances "isoforms").

4. In all figures, real p-values instead of stars or n.s. should be shown.

5. Page 5, line 121: there appears to be a typo: is it meant "catalytically INactive", not "active"?

6. There is missing information about the Centrin-2 antibody in the Methods.

7. Legend of Fig. EV1A: the colours of Centrin2 and DNA in the legend are swapped.

8. Fig. 2D: are the images to the same scale? The nuclei in the sip150 condition appear smaller than the siCtrl. Is this a phenotype of sip150 treatment?

9. Fig. 3C and D: was this analysis performed on cells in a 3D matrix? Does it work good for cells that are not so polarized? It would be important to show an example on a real image just below the cartoon.

10. Fig. 3I: did the authors assess whether the microtubules that are nocodazole-resistant are also detyrosinated, especially those that are not positive for acetylation staining?

11. The ROS analysis by live-cell imaging is incorrectly referred to as Fig. EV3E,F in the text (p.8, line 205); it is EV3C,D in the figure. The lack of explanation in the Results of what is "DCF" used for this imaging also creates confusion about this experiment.

12. Line 215: "These results demonstrate that cells with extra centrosomes have increased acetylated microtubules at least in part due to higher levels of total tubulin." This statement is puzzling - do the authors mean to say that there is no specific effect of acetylation at all? Is it all about more microtubules?

13. Fig EV4A,B: is there a reason authors do not show images of their siRNA-treated cells? They show images for every other experiment in this manuscript. Also, how do the authors explain that a 50% reduction of ATAT1 expression is sufficient to abolish acetylation? After all, ATAT1 is an enzyme and should still be able to do its function when overall levels are reduced.

14. In Fig. 5G, how was mitochondria area determined as a fraction of whole cell area - was segmentation used? Also, the labelling of the shown staining is missing for these images.

15. Fig. EV5F: the overview of the phenotypes in this table is very useful, but the H2O2 treatment should be included as well.

16. Page 10, line 251: it seems that author wanted to cite Fig. EV5E, not D - please confirm.

17. Fig.7E,F: please describe how the migration was quantified, and what the different phases mean?

Manuscript EMBOJ-2022-112812

"Centrosome amplification fine-tunes tubulin acetylation to differentially control intracellular organization"

Referee #1:

The authors investigate the polar organisation of cells treated with a PLK4 inhibitor, which results in centrosomal duplication and find that treated cells translocate their centrosomes (and associated with it a number of organelles) from an area in nuclear proximity to the cell periphery. To understand the mechanism underlying this phenomenon, they follow the concept of a push-pull balance, where the centrosome shifts between perinuclear and peripheral based on a balance between kinesin 1 driven pushing and dynein driven pulling forces. genetic and pharmacological experiments support this idea. The authors then move to the role of microtubule modification in this phenomenon and find that cells with multiplied centrosomes have higher levels of acetylation and increased levels of tubulin. Enhanced acetylation explains some but not other organelle displacement phenotypes that were previously described, potentially linking acetylation and kinesin activity. Finally the authors link the observed phenotypes to altered vimentin distribution and enhanced nuclear deformability, which in turn affects migration in 3D environments.

The main problem with this manuscript is that despite multiple rounds of reading, I cannot understand the causal logic that leads from one experiment to the other. The readout of the experiments is a very crude quantification of organelle or centrosome positioning, while the manipulations are also very generic. The experiments no not directly address the mechanistic basis of the cell biological process.

We thank the reviewer for their comments on the manuscript. We hope we have significantly addressed the concerns raised below.

Some major issues:

1) It is not clear to me how the push-pull concept of centrosome positioning is directly supported by the experiment when the authors disengage microtubules and nucleus using the dominant negative kASH approach. The authors do not even provide a mechanistic framework how this result could support the conclusions. Importantly, no measurements, like e.g. laser ablations, are performed to challenge the concept.

We understand this was unclear and not explained well in the original manuscript, we apologise for the confusion this caused. In fact, reviewers #3 and #4 raised similar comments, and we have found during the revision process that our initial description and idea, specifically regarding the role of kinesin-1 when the LINC complex is disrupted, was oversimplified. Our hypothesis was based on the assumption that kinesin-1 mediated pushing forces on centrosomes does not require an association between microtubules and the LINC complex. This assumption was based on recent work from the Rusan lab (Hannaford *et al*, 2022; PMID: 35929834), which showed that, in *Drosophila*, kinesin-1 interacts with PLP (Pericentrin-like protein) allowing centrosomes to move along microtubules. Furthermore, work from the Chavrier lab (Infante et al., 2018; PMID: 29934494) showed that laser ablation of the microtubules that link centrosomes to the nucleus can promote centrosome displacement, although it is unknown which motor protein is responsible for this phenotype. Thus, it is possible that KIF5B's role on driving the movement of centrosomes could be independent of the association between microtubules and the LINC complex. We have tested this idea by depleting

KIF5B in cells with extra centrosomes expressing KASH2 and found that KIF5B depletion is sufficient to prevent centrosome displacement (Rebuttal Figure 1). We have not further validated this with laser ablations, as the reviewer suggested, since these are not easy experiments to perform, but the work performed by the Chavrier's lab suggests that centrosomes would still be displaced upon laser ablation.

These new data are consistent with our initial idea; however, we are aware that it also raises more questions specifically on centrosome displacement that we cannot answer at this time. In the case of other organelles and vimentin tested in our study, kinesin-1 has been shown to associate and drive their transport along microtubules. Because of this, while the new data opens many interesting questions, we do not think it adds enough to our manuscript and conclusions in its current form and we suggest to remove it entirely. We have already removed from this new version, however if the reviewers think it is important to add it back, we are happy to do so.

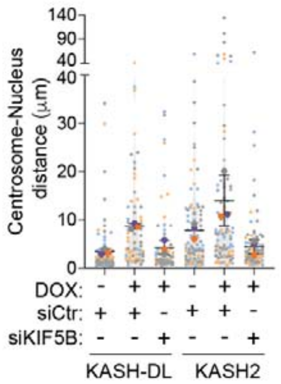

Rebuttal Figure 1. Displacement of centrosomes in cells overexpressing the dominant negative GFP-KASH2. Quantification of centrosome-nucleus distance. Depletion of KIF5B from cells with amplified centrosomes (+DOX) prevents centrosome displacement in cells overexpressing both the control KASH-DL and the dominant negative KASH2 constructs. These data suggest that, even when the LINC complex is disrupted by KASH2 overexpression, KIF5B is still driving centrosome displacement in cells with extra centrosomes. Error bars represent mean ±SD from three independent experiments.

2) The authors take a large leap from centrosome duplications to microtubule acetylation and this step is motivated by the observation that there is more acetylation (but also more tubulin) in the duplicated setting. I cannot understand what justifies this step and how the two phenomena are interlinked. The idea that kinesins mediate this effect is plausible but not directly supported by experiments.

We have tried to improve the rationale of some sections in the revised manuscript. Centrosome positioning, and the position of many organelles/intracellular compartments inside the cell, has been proposed to be regulated by a balance of opposing forces driven by microtubule motors: dynein and plus-end directed kinesins (e.g. kinesin-1). Our data on Figure 1G indeed demonstrates that kinesin-1 is responsible for the displacement of the amplified centrosomes, thus suggesting that something is driving the imbalance of forces that culminates in centrosome displacement. We have added more rationale on this on page 8, lines 182-193. To summarise the key points: 1) Dynein activity does not seem compromised in cells with amplified centrosomes since depletion of KIF5B results in closer localisation of centrosomes, endosomes and vimentin to the nucleus, which is driven by dynein. 2) No differences in the total levels of KIF5B were observed that could change the balance of forces (New Figure EV3A,B). Based on this, we then considered that microtubule PTMs that have been implicated in enhancing motor activity and/or motility, could be responsible for this imbalance and hence the motivation to look at this. It's an educated guess based on what we know from the literature, as referenced in the manuscript. We hope this clarifies our rationale for the reviewer.

We should also note that having more total tubulin does not take way from having more acetylated tubulin. The increase in tubulin that is acetylated is what matters, as this would favour, for example, kinesin-1 motility. Our data on Figure 1G shows that depletion of kinesin-1 prevents centrosome

displacement in cells with amplified centrosomes. Thus, we are unsure what additional evidence the reviewer wants to see? We also would like to point out that kinesins, and in particular kinesin-1, are responsible for the transport of vimentin and mitochondria along microtubules towards cell periphery (plus-end directed motility), which supports our findings.

3) Another large leap is the manipulation of acetylation using H2O2. This is a completely unspecific approach, which does not allow any conclusions.

We agree that H2O2 is not a very specific approach. This is why additional approaches are provided in our manuscript. We induce tubulin acetylation with a well-known HDAC6 inhibitor, Tubacin (although this approach is not the most specific), as well as the overexpression of αTAT1 (αTAT1 does not acetylate histones and no other substrate has been identified). In addition, we also depleted αTAT1 to prevent tubulin acetylation to identify which organelles/intracellular compartments require tubulin acetylation to be displaced. We have provided several orthogonal conditions/experiments in this manuscript, in addition to the fact that some of our data is consistent with what has been already published, and we do not rely solely on H2O2 treatment to make any conclusions, as we hope the reviewer can appreciate.

4) The obervation that some but not other organelles are influenced by acetylation is interesting but not followed up at all.

We appreciate this comment and we also think this is very interesting. But we believe that is out of the scope for this work.

5) the link between acetylation, intermediate filaments and nuclear deformability is again not directly addressed.

We have now provided more data that we hope can address this point. Firstly, we looked at nuclear deformability in cells treated with H2O2, which also increases tubulin acetylation levels, and found that this leads to increase nuclear deformability similar to centrosome amplification. Secondly, we depleted αTAT1 from cells with amplified centrosomes and found that this was sufficient to restore nuclear shape (New Figure 8F). This further supports our hypothesis that tubulin acetylation, likely via changes in kinesin-1 motility (as KIF5B depletion also prevents nuclear deformation, Figure 8E), is important for this phenotype. In addition, we also assessed nuclear translocation through small and large pores in these conditions. Consistent with our data on nuclear deformability, we found that H2O2 treatment facilitates faster nuclear translocation through small pores (5 μm) but has no effect on larger pores (8 μm). Furthermore, αTAT1 depletion in cells with amplified centrosomes does not affect migration in larger pores (8 μm) but prevents faster nuclear translocation in smaller pores (5 μm) (New Figure 8I). Thus, tubulin acetylation facilitates nuclear translocation through confined spaces.

Vimentin depletion has been previously shown to decrease perinuclear stiffness and enhance migration in microchannels with 10-20 μm width (Patteson et al., 2019; PMID: 31721440). This increased nuclear deformation in cells without vimentin leads to nuclear envelope rupture and DNA damage when migrating in confined 3D environments, thus affecting the overall cell viability (Patteson et al., 2019; PMID: 31676718). Similarly, we observe that depletion of vimentin leads to extreme nuclear deformation in cells migrating though 5 μm pore (this was never tested before). The highly deformable nuclei become stuck in these small channels, preventing efficient migration (Rebuttal Figure 2). Thus, it is possible that the displacement of vimentin towards the leading edge, rather than the total loss of vimentin, is important in allowing the nucleus to deform while providing some mechanical protection during confined migration (this is further discussed on page 17, lines 438-453).

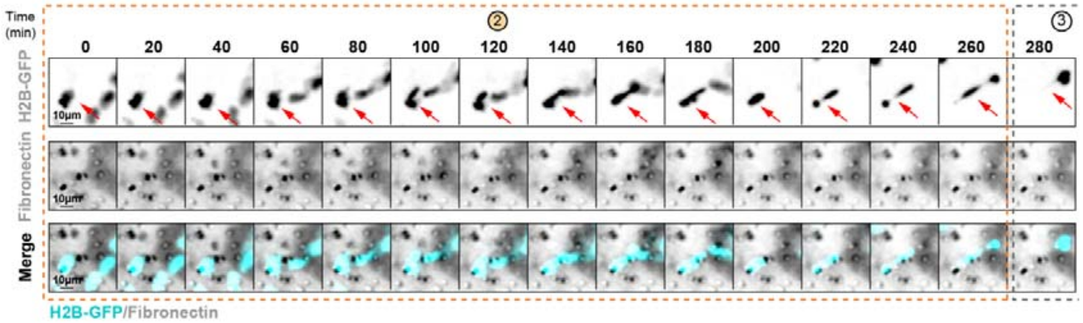

Rebuttal Figure 2. Time lapse of RPE-1 H2B-GFP cells depleted of vimentin migrating through small pores (5 µm). Red arrow indicates a nucleus crossing a small pore. A highly deformed nucleus can be seen at 220-260 minutes due to vimentin depletion. Scale bar = 10 µm.

Referee #2:

This is a very interesting and well-executed paper to study the effects of increased centriole numbers upon cytoskeletal events in RPE-1 cells resulting from elevated levels of Plk4. The authors find that the amplified centrioles are displaced to wards the periphery of the cell in a manner that requires KIF5B and is associated with an increased acetylation of tubulin. Indeed, depletion of the aTAT1 acetyl transferase prevents the displacement of centrosomes and other organelles, with the exception o the Golgi and endosomes. Finally cells with displaced centrosomes display increased ability of their nuclei to become deformed, facilitating cell migration through small pores.
These are interesting observations, particularly in light of tumor cells to have supernumerary centrosomes. The conclusions are adequately supported by the experimentation and I recommend publication in EMBOJ without the need for revision.

We thank the enthusiastic comments from this reviewer about our work!

Referee #3:

In this manuscript, Monteiro and co-authors show that centrosome duplication results in a change of organelle positioning and increased levels of K40 acetylated microtubules. The interplay of kinesin-1 and dynein motor complex seem to be important for the organelle displacement. Changing the level of microtubule acetylation by knockdown of αTAT1 or Tubacin treatment had an effect on several organelles and subcellular structures whereas others were not affected. The duplication of centrosomes and organelle displacement influenced nuclear shape and deformability leading to facilitated migration of cells through confined spaces. Altogether the authors propose an interesting process of deregulated organelle localization due to centrosome duplication which is especially interesting in the context of cancer and metastasis.

We thank the reviewer for their enthusiasm and carefully going through the manuscript. We hope we have significantly improved the manuscript by addressing the points raised below.

This is a well performed study which in my opinion will be suitable for publication after addressing several points:

Major points:

1. Abstract: "Depletion of a-tubulin acetyltransferase 1 (aTAT1) to block tubulin acetylation, which has no impact on control cells, rescues the displacement of centrosomes, mitochondria and vimentin, but not Golgi or endosomes." In the abstract it is not clear what on what exactly depletion of ATAT1 has no effect. Please rephrase and clarify.
We thank the reviewer for their comment and understand why this was not clear. We have now removed *"which has no impact on control cells"* from the abstract since on its own it is confusing, although we think this is a key result. We have now explained this in the main text where we could expand our interpretation, page 10, lines 246-251.

2. Figure 2: In panels I and J the distribution of vimentin is measured in a 3D environment. However in 3D distance is not only in x,y direction. Has the distance in z direction also been considered?
Based on the 3D projection of our images, it is clear to see that on the z-axis cells are very thin, which makes performing analyses in 3D near impossible. This also demonstrates that there is not much space for vimentin to move on the z-axis and thus it is unlikely that this will negatively affect our analyses (Rebuttal Figure 3).

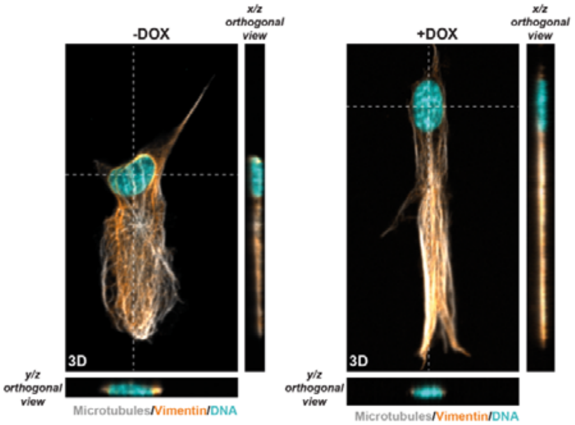

Rebuttal Figure 3. Z-axis projection of cells plated in 3D collagen matrices with normal centrosome number (-DOX) or with extra centrosomes (+DOX). Cells were stained for microtubules (α-tubulin, grey), DNA (cyan) and vimentin (orange). z-axis shows that there is not much space where vimentin could move in 3D.

3. Figure 5: The authors show that some organelles are displaced by increased microtubule acetylation independent of centrosome duplication. However, there is no analysis of Tubacin treatment/αTAT1 overexpression vs PLK4 overexpression and therefore centrosome duplication. In order to show that the effects are comparable a quantification of the displacement between these two ways to induce organelle displacement would be beneficial.
We appreciate the reviewer's comment. We have done these analyses multiple times and although we know the phenotypes are comparable in these conditions, we have now repeated the analyses of the displacement of centrosomes, mitochondria and vimentin in PLK4 OE, Tubacin and αTAT1 OE cells side by side. The new quantifications are provided in Figure 5F,H,J. Apart from centrosome displacement, which we know does not respond to Tubacin treatment or αTAT1 OE, equivalent phenotypes for vimentin and mitochondria displacement are observed in all three conditions.

4. Figure 6: What happen to the MT polarity (+end, -end localisation)? This would give further information regarding force generation by kinesin and dynein motors and net force outcome.
This is an interesting point that we did not consider. Indeed, for our model to work, we assumed that microtubule polarity does not change between conditions and that most microtubules display the characteristic polarity: minus end (cell centre) to plus end (cell periphery). To assess if this is indeed the case for all conditions analysed, we imaged EB3-GFP/EB3-tdTomato expressing RPE-1 cells to track microtubule plus ends and quantified the % of EB3 comets that move towards cell periphery (normal) versus the % of comets that move sideways or towards the cell centre (abnormal). As seen in Fig 6E,F (and Movie 1 and 2), these data clearly demonstrate that

microtubule polarity is unchanged in all conditions, with >97% of all EB3-labelled microtubule +ends moving towards cell periphery in all conditions analysed. This is further explained on page 12, lines 300-305.

5. The authors show that increasing tubulin acetylation by overexpression of ATA1 is sufficient to promote organelle displacement independently of amplified centrosomes. I am wondering if increasing acetylation for example by treatment with taxon would lead to a similar outcome.
The issue with Taxol is that it increases both tubulin acetylation and detyrosination and thus it is not very specific (Rebuttal Figure 4), which is consistent with previous published work (Piperno *et al*, 1987; PMID: 3722261). In addition, Taxol stabilises microtubules, which could also affect all intracellular transport. Thus, while we thank the reviewer for their comment, we have decided to focus on the conditions already in the manuscript, such as Tubacin and αTAT1 overexpression.

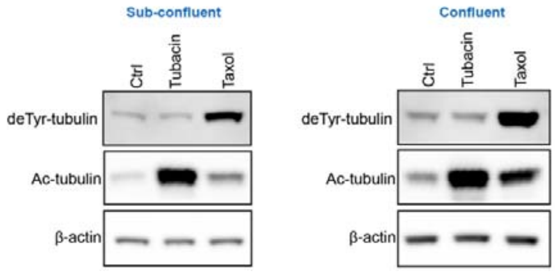

Rebuttal Figure 4. Western blotting analysis of lysates of cells treated with Tubacin (5 μM /4 hrs) and Taxol (10 μM/1hr). We analysed cells plated at sub-confluency as well as confluency stages since we analyse IF images in sub-confluent cells and wanted to check if confluency could affect the results, which does not.

6. Line 153-156: The authors conclude that after dominant-negative GFPKASH2 expression the interaction of the centrosome with the nucleus is disrupted. The combination of centrosome duplication and dominant-negative GFPKASH2 expression leads to even greater displacement. This raises the question if the centrosomes can still be somehow attached to the nucleus if they are displaced and multiple μm away from the nucleus (Fig. EV1H,I).
This comment highlights a part of our manuscript that we now think was confusing and oversimplified. Indeed, we realised, also by taking into account additional comments raised by reviewers #1 and #4, that our initial description and idea about kinesin-1's role in centrosome displacement upon LINC disruption was oversimplified. Our hypothesis was based on the assumption that kinesin-1 pushing forces on the centrosomes do not require an association with the LINC complex. This assumption was based on recent work from the Rusan lab (Hannaford *et al*, 2022; PMID: 35929834), which showed that, in *Drosophila*, kinesin-1 interacts with PLP (Pericentrin-like protein) allowing centrosomes to move along microtubules. Furthermore, work from the Chavrier lab (Infante et al., 2018; PMID: 29934494) showed that laser ablation of the microtubules that link centrosomes to the nucleus can promote centrosome displacement, although it is unknown which motor protein is responsible for this phenotype. Thus, it is possible that KIF5B's role on driving the movement of centrosomes could be independent of the association between microtubules and the LINC complex. We have tested this idea by depleting KIF5B in cells with extra centrosomes expressing KASH2 and found that KIF5B depletion is sufficient to prevent centrosome displacement (Rebuttal Figure 1– *copied below*).

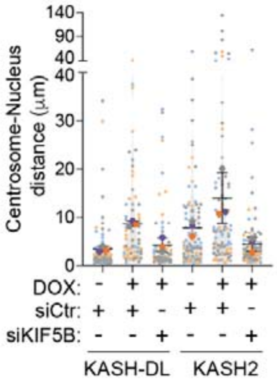

Rebuttal Figure 1. Displacement of centrosomes in cells overexpressing the dominant negative GFP-KASH2. Quantification of centrosome-nucleus distance. Depletion of KIF5B from cells with amplified centrosomes (+DOX) prevents centrosome displacement in cells overexpressing both the control KASH-DL and the dominant negative KASH2 constructs. These data suggest that, even when the LINC complex is disrupted by KASH2 overexpression, KIF5B is still driving centrosome displacement in cells with extra centrosomes. Error bars represent mean ±SD from three independent experiments.

.

These new data are consistent with our initial idea; however, we are aware that it also raises more questions specifically on centrosome displacement that we cannot answer at this time. In the case of other organelles and vimentin tested in our study, kinesin-1 has been shown to associate and drive their transport along microtubules. Because of this, while the new data opens many interesting questions, we do not think it adds enough to our manuscript and conclusions in its current form and we suggest to remove it entirely. We have already removed from this new version, however if the reviewers think it is important to add it back, we are happy to do so.

7. Lines 356-359: The authors state that they see a polarized distribution of acetylated MTs upon treatment with hydrogen peroxide. On page 8 they state that hydrogen peroxide damages the microtubule lattice which leads to increased acetylation. This raises the question how a bath application of hydrogen peroxide can lead to a polarized distribution of microtubule acetylation which is not addressed by the authors.
We appreciate that this was not well explained in the original manuscript. It is important to keep in mind that we use low doses of H_2O_2 because high doses lead to massive tubulin acetylation, as expected due to severe damage, and changes in microtubule architecture (e.g. loss of centrosomal microtubule nucleation; cells look unhappy), as seen in Rebuttal Figure 5. The concentration of H_2O_2 used was optimised to mimic what we see in cells with extra centrosomes (~2-fold increase in the levels of tubulin acetylation). We have now explained this better on page 10, lines 256-258. We do not know why low doses of H_2O_2 lead to polarised acetylated microtubules. One interpretation is that the distribution of acetylated tubulin follows total microtubule distribution (as seen in Figure 7), which is already polarised.

We also discuss why we think tubulin gets acetylated preferentially closer to the centrosomes/perinuclear region. The area around the centrosome is where the highest concentration of 'free' plus ends exist, as microtubules are being nucleated, which provides an entry point for αTAT1 to access the microtubule lumen. It would also be interesting to test if endogenous αTAT1 could associate with the centrosomes, however, current antibodies do not work well in IF or WB. We tried to discuss this in the main text without giving too many speculative hypotheses that we cannot test at the moment (page 9, lines 227-230).

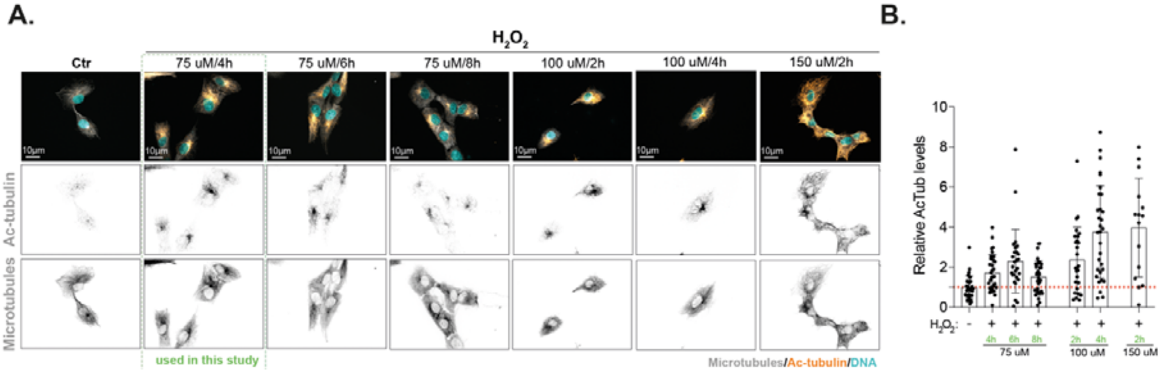

Rebuttal Figure 5. Optimisation of H_2O_2 concentrations. We have tested different H2O2 concentrations and different times to ensure the concentration chosen induces similar levels of tubulin acetylation to centrosome amplification without affecting general microtubule network and cell morphology. **A.** Cells were stained for microtubules (α-tubulin, grey), DNA (cyan) and ac-tubulin (orange). Scale bar = 10um. **B.** Quantification of acetylated tubulin levels in the different conditions.

8. Statistical analysis: It is not stated how exactly statistical tests are performed. Are they performed on all values or on the means of individual experiments? This is also not clear because in the graphs all single values as well as means of individual experiments are shown. Also, often student's t-tests are used and no information about normal distribution are stated.

We thank the reviewer for pointing this out and have clarified this in the methods. All statistical tests were performed using the averages, not on all points, which is regarded to be the correct way of assessing significance without overpowering our data (Lord et al., 2020; PMID: 32346721). The main reason why we use superplots, is that it allows the reader to assess the distribution of all the data, as opposed to averages values only. We always evaluate if the data are normally distributed before performing statistical analyses using the Shapiro-Wilk normality test but this information was missing from the original manuscript. We have added this information to the methods as well. All statistical tests used are additionally stated in the figure legends for all graphs.

Minor points:

9. In some figure panels two channels are both shown in grey scale on the same image. Better use different colours.

We have now changed this so that in the inverted images that contain two channels the DNA is always in cyan to match the merged image. We do think that the inverted grey-scale images work well as they are easier to see and we have changed the way we labelled these images to make them clearer. We hope this addresses the reviewer's concern.

10. It it better to be consistent with the colours used for the representative images. For example, in Fig. 2 panel F in the upper pictures microtubules are shown in grey and vimentin in orange. In the lower pictures vimentin is shown in grey. In Fig. 4 panel C in the upper pictures microtubules are shown in grey and vimentin in orange. In the lower pictures vimentin is shown in grey, which is confusing.

We understand this comment. However, we do think that the inverted grey-scale images are clearer and we would like to be able to keep them as they are. We would like to emphasise that the lower panels are inverted images, not just simply greyscale and are labelled. We have changed the labels in the panels and we hope these are now clearer and less confusing.

11. Line 129-133: Claims about the centrosome nucleus distance due to nocodazole and latrunculin A treatment are made. However, statistical analysis for this is missing. Comparison -Dox, -Noc, -LatA vs -Dox, +Noc/+LatA (Fig. EV1C,D).
We thank the reviewer for highlighting this. We have now added all the stats. We also realised that the statistical test was incorrectly done using on all data points for this particular experiment, instead of the averages, and this has now been corrected as well. The main point we would like to make with these analyses is that, without microtubules, the differences observed in centrosome displacement are lost, while upon actin depolymerization the displacement of centrosomes can still be observed.

12. Statistics for centrosome displacement in control cells after KIF5B or p150 depletion is missing. Comparison of -Dox, -siKIF5B/-sip150 vs -Dox, + siKIF5B/+sip150 (Fig. 1G).
We would like to thank the reviewer again for highlighting this and all stats have now been added. As expected, depletion of KIF5B from control cells leads to a shorter distance between nucleus and centrosomes, while depletion of p150 is sufficient to increase the distance between nucleus and centrosomes. These differences are statistically significant and are in agreement with previous work.

13. Line 175-176: The statistics for the comparison of p150 depletion in control cells compared to doxycyclin treated cells are missing.
The difference between +DOX and -DOX siRNA p150 is not statistically significant. This stat as now been added to graph 2J.

14. Line205: figure mentioned should be Fig. 3E,F not EV3.
In the original manuscript we made a mistake while referencing these panels, as spotted by the reviewer. This particular sentence refers to the ROS data, now described on page 9, line 215, and are now presented in Figure EV3E,F.

15. Lines 354-355: Please explain to what the levels of acetylated microtubules was compared to.
The comparison is made against control, untreated cells. We have now added this to the text, page 16, line 401. We have also added the quantification of tubulin acetylation levels in Tubacin and aTAT1 OE cells to Figure EV5F and described it in the main page 11, lines 264-266.

16. Centrin 2 antibody is missing in the antibody table.
We have now added centrin2 antibody to the table.

17. Fig. 2: In figure legend for panel C there are words missing (...upon induction of PLK4...). For panel C are the n numbers endosomes or cells?
We have corrected this. All *n* numbers refer to cells, we appreciate this was not clear in the original manuscript and we have now added this information to all figure legends as such: *n = number of cells analysed.*

18. Fig. 3: In panel A the Ac-tubulin picture is shifted down compared to merged picture.
Corrected.
Panels I and J are mentioned in the text before E-H (line 197).
Corrected.
In panel D statistics are missing.
This is now Figure EV3D and stats have been added. The differences obtained between -DOX and +DOX analyses are statically significant for ac-tubulin distribution, but not for total tubulin (new graph on the right of Figure EV3D).
In panels F, G, H the y-axis legend is incomplete (relative intensity?).
Corrected.

In figure legend for panel H the word intensity is missing.
Corrected.
In description of statistical tests there is a typo: there is no panel K it should be H.
Corrected.

19. Fig. 4: Which siRNA for αTAT1 was used in the representative pictures (panel A, C, E)?
We used images from siRNA aTAT1#5 as examples but they are representative from all experiments. We have now added this information to the panels.
In panel D the statistic for siCtr comparison of +Dox and -Dox is missing.
This has now been added.

20. Fig. 6: Are the n numbers for this figure cells or microtubules? Please indicate in figure legend.
n = number of cells analysed. This has now been added to all figure legends as explained above.

21. Fig. EV1: Statistics for panel I are missing.
This panel has now been removed from the manuscript.

22. Fig. EV3: C and D are not mentioned in the manuscript.
There was a mistake with the referencing. This has now been corrected and these panels are now referenced in the text on page 9, line 215, and are now presented in Figure EV3E,F.

Please explain what DCF is doing.
We have now added an explanation in the min text, page 9, lines 213-215: " (…) ROS levels, as measured by the levels of Dichlorodihydrofluorescein (DCF) that results from ROS-mediated oxidation of hydrolysed H_2DCFDA." We have also added this information to the methods, page 28, lines 676-678.

23. Fig. EV4: Which siRNA for αTAT1 was used in the representative pictures (panel C, E, G)?
αTAT1#5 was used, now added to all panels.
It would be better to keep consistent with the colours used for the representative images. For example, in panel C in the upper pictures EEA1 is shown in orange and F-actin in grey. In the lower pictures EEA1 and DNA is shown in grey, which is confusing.
We have now modified all images so that DNA is in a different colour (cyan), but we kept inverted images for endosomes. The inverted grey scale images are easier to see, that is why we prefer them. We have changed the labels in the panels to make them clearer. We hope this will satisfy the reviewer.
Figure legend for panel C: Is the n number cells or endosomes?
n = number of cells analysed, now added to all figure legends as explained above.

24. Fig. EV5: In panel E the authors show the increased MT acetylation upon Tubacin treatment and αTAT1 overexpression. However, they only show one cell for each condition. A bigger field of view with multiple cells or quantification of the increase would be more convincing.
We have now added the quantification for this. The data is very robust for these conditions, where we observe a ~50-fold increase in the levels of acetylated tubulin compared to untreated control cells. We appreciate that did not come across by showing one example. This data is now added to Figure EV5F.
Panel F is not mentioned in the text.
This is now panel H, and it is now referenced in the text, page 11, line 273.

Referee #4:

In this manuscript Monteiro and colleagues demonstrate that overamplification of centrosomes leads to reorganization of organelle positioning inside cells, and that this process is dependent on kinesin-1-mediated transport and polarized distribution of acetylated microtubules towards the cell periphery.

The authors perform a systematic and carefully executed study on the role of centrosome amplification, induced by the overexpression of Plk4, on the re-organisation of the intracellular space. They first demonstrate that centrosome amplification leads to the displacement of the centrosomes in a kinesin-1-depended manner, and that this process induces a global intracellular re-organisation of different organelles. Next, they show that cells with extra centrosomes have increased levels of alpha-tubulin acetylation, suggesting that perhaps this change in microtubule modification could be the cause of the observed re-organisation of the intracellular space. The authors test this hypothesis by experimentally modulating the levels of tubulin acetylation and indeed show that they can reproduce most, however, not all of the re-organisation events induced by centrosome amplification. They also follow up a previous observation that oxidative damage increases tubulin acetylation, and demonstrate that accordingly, applying oxidative damage to cells is sufficient to induce intracellular reorganisation. Because increase of tubulin acetylation alone was not sufficient to induce displacement of centrosomes (while it did induce the reorganisation of most other intracellular components), the authors performed a detailed image analyses of the cells to determine how polarity of acetylation patters could explain the observed effects. Finally, the authors show that following global organelle re-distribution, the nucleus tolerates higher degrees of deformation, thus giving the cells an increased ability to migrate through narrow spaces.

This is a carefully executed, and well-documented study. The manuscript is well-written, and figures are of high quality. In the introduction and discussion section the authors did a great job in introducing the relevant literature, and to thoughtfully discuss their findings in the light of this literature. The study provides a systematic characterisation of the consequences of centrosome overamplification on intracellular organization of organelles, supported by well-executed experimental procedures. The key finding is that tubulin acetylation, a so-far functionally understudied posttranslational modification of microtubules, is induced by centrosome overamplification, and mediates intracellular re-organisation. It is thus an important step toward the functional understanding of the physiological roles of tubulin modification. However, some aspects remain rather descriptive and do not propose a deeper mechanistic explanation of the links between the different phenotypes, for example how tubulin acetylation levels could be modulated by the centrosome amplification. Nevertheless, the findings reported in this manuscript contribute important novel insight into microtubule-based mechanisms of intracellular organisation, which will be key for future research on migration of cancer cells with supernumerary centrosomes. A revised version of this manuscript would thus be a strong candidate for publication in EMBO J.

We thank the reviewer for their enthusiasm and carefully going through the manuscript. We hope we addressed the points raised by the reviewer that we believe significantly improved this work.

Major points:

1. Does PLK4-induced centrosome overamplification lead to abnormalities in nucleus shape or presence of micronuclei due to mitotic defects? If yes, were such cells included in the analysis (e.g. for determining nuclear circularity?)
We appreciate this concern. However, in our conditions there are very few cells with micronuclei upon induction of centrosome amplification, which we exclude from our analyses. We have added

this to our methods, page 26, lines 617-618. Regarding nuclear abnormalities, interestingly we did not observe any shape change in cells plated in 2D. It is only when cells are plated in 3D and in a confined space that we observe a decrease in nuclear circularity. We have added the 2D quantification to Figure 8C and explained this in the text, page 13, line 325-326.

2. Fig. EV1H,I: the authors should test the possibility that the observable further displacement of the centrosome upon detachment of the nucleus could be explained with the greater mass of microtubules, which, as they show, results from having multiple MTOCs? This should be quantified.
This is an interesting idea. Unfortunately, we presently do not have a good way to decrease microtubule mass in cells with amplified centrosomes without affecting microtubule PTMs. We have tried to test this in a different way. Our hypothesis is that kinesin-1 could act on microtubules that are not attached to the LINC complex. This was not well explained in the original manuscript and a source of confusion also for reviewers #1 and #3. Our initial hypothesis, which we now think was oversimplified, was based on the assumption that kinesin-1 pushing forces on the centrosomes do not require an association with the LINC complex. This assumption was based on recent work from the Rusan lab (Hannaford *et al*, 2022; PMID: 35929834), which showed that, in *Drosophila*, kinesin-1 interacts with PLP (Pericentrin-like protein) allowing centrosomes to move along microtubules. Furthermore, work from the Chavrier lab (Infante et al., 2018; PMID: 29934494) showed that laser ablation of the microtubules that link centrosomes to the nucleus can promote centrosome displacement, although it is unknown which motor protein is responsible for this phenotype. Thus, it is possible that KIF5B's role on driving the movement of centrosomes could be independent of the association between microtubules and the LINC complex. We have tested this idea by depleting KIF5B in cells with extra centrosomes expressing KASH2 and found that KIF5B depletion is sufficient to prevent centrosome displacement (Rebuttal Figure 1– *copied below*). Thus, we do not think that it is the increased microtubule mass that is pushing the centrosomes away, but rather the motor that is still functional in cells where the LINC complex was disrupted.

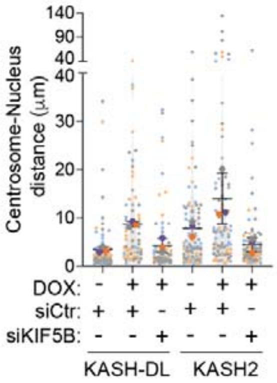

Rebuttal Figure 1. Displacement of centrosomes in cells overexpressing the dominant negative GFP-KASH2. Quantification of centrosome-nucleus distance. Depletion of KIF5B from cells with amplified centrosomes (+DOX) prevents centrosome displacement in cells overexpressing both the control KASH-DL and the dominant negative KASH2 constructs. These data suggest that, even when the LINC complex is disrupted by KASH2 overexpression, KIF5B is still driving centrosome displacement in cells with extra centrosomes. Error bars represent mean ±SD from three independent experiments.

These new data are consistent with our initial idea; however, we are aware that it also raises more questions specifically on centrosome displacement that we cannot answer at this time. In the case of other organelles and vimentin tested in our study, kinesin-1 has been shown to associate and drive their transport along microtubules. Because of this, while the new data opens many interesting questions, we do not think it adds enough to our manuscript and conclusions in its current form and we suggest to remove it entirely. We have already removed from this new version, however if the reviewers think it is important to add it back, we are happy to do so.

The authors should also check whether Kif5B levels in cells with normal and supernumerary centrosomes are similar.

We have quantified KIF5B levels in cells with and without extra centrosomes, in sub confluent and confluent cells, and observed no differences. Data was added to Figure EV3A,B and described in the text, page 8, lines 188-189.

3. For the vimentin displacement analysis, was the cell shape taken into account? This must be clarified as in the less polarized cells the distance between the nucleus and the leading edge can be rather small, resulting in overlap in the measured signal.
Our measurements of vimentin displacement already take into account some component of cell shape, or more precisely size, by assessing the ratio of nuclear versus leading edge vimentin. There is no overlapping signal because we select nuclear region: 15 µm from NE and leading-edge region: 20 µm from LE (Figure 2F). We have also superimposed the outlines of all cells analysed as well as total microtubules, Figure EV5G, which show that there are no major changes in cell polarisation between all conditions. This is now explained in the text, page 11, lines 268-270.
Also, was the overall mass of vimentin different between the conditions, and/or did it correlate to the microtubule mass)?
As can be seen in Rebuttal Figure 6, cells with amplified centrosomes have increased total mass of vimentin, that is correlated with the microtubule mass (Rebuttal Figure 6A). However, Tubacin treated and αTAT1 OE cells have similar vimentin mass to control untreated cells (Rebuttal Figure 6B). Thus, considering that vimentin displacement is observed in cells with amplified centrosomes, treated with Tubacin or overexpressing αTAT1, it can be concluded that changes in the vimentin mass cannot account for the displacement of vimentin observed in these conditions.

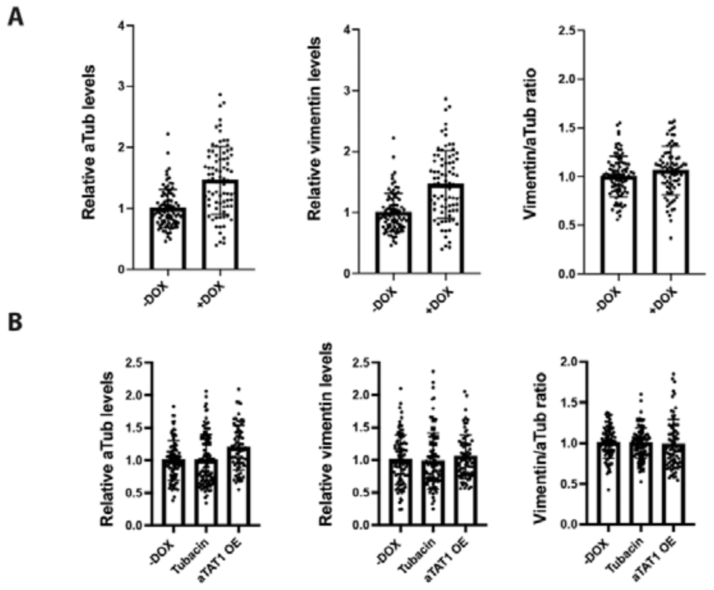

Rebuttal Figure 6. A. Quantification of α-tubulin and vimentin fluorescence intensity in control cells (-DOX) and cells with amplified centrosomes (+DOX). **B.** Quantification of α-tubulin and vimentin fluorescence intensity in control cells (-DOX), cells treated with Tubacin or overexpressing αTAT1. Data from 3 independent experiments.

4. Fig. 3D and EV3B: The authors may consider swapping Fig. 3D and Fig. EV3B. It appears that the current Fig. EV3B provides a stronger support for their conclusion that the distribution of acetylated tubulin does not decrease as dramatically in cells with overamplified centrosomes with increasing distance from the nucleus.
We have now swapped the panels as suggested by the reviewer.
In the legend of EV3B the authors should also include information on how many cells, and which regions were analysed for the generation of the curve.
This is now Figure 3C and numbers have been added to figure legend.
The authors should also verify whether the total tubulin signal increases or remains similar in both conditions, and in the different regions of the cell.

We have now analysed the distribution of total tubulin in -DOX and +DOX cells. Graph in Figure EV3D shows that the α-tubulin signal is comparable between -DOX versus +DOX cells. Thus, the observed differences in the distribution of tubulin acetylation cannot be explained by the loss of α-tubulin signal. This is now explained in the text, page 8, lines 199-200.

5. Fig. 3J: it appears that the number of individual microtubules was counted. This is not a good way of doing these analyses for several reasons. First, how are single microtubules distinguished in images like those in the bottom row of Fig.3I (+DOX, +Noc), where the network is dense? Secondly, and most importantly, how is microtubule length taken into account? A potential solution is to either quantify microtubule mass, or microtubule length (summing up the length of all microtubules in one cell).
We appreciate the concerns raised by the reviewer. However, we believe that the best way to assess stable microtubules is by quantifying the number of microtubules per cell. We have now added additional explanation in the methods on how this is done, page 30, lines 718-719. Specifically, we quantify microtubules that emanate from the centrosomes, and thus we are not over estimating numbers and we are able to distinguish individual microtubules. We also use z-stacks to quantify microtubule number although this is more difficult to appreciate in the images provided. We do not take length into account as the Noc-resistant microtubule are clearly longer and length would be more difficult to quantify since these microtubules can be curved and difficult to disentangle closer to the periphery, hence why we quantify the microtubules nucleated from the centrosomes. However, we have used these images to quantify total α-tubulin intensity as suggested by the reviewer which shows the same result. Data has been added to Figure 3F and discussed in the text, page 8, lines 204-205.

6. Does the analysis of acetylation orientation consider the irregularities in cell shapes, and how much space is there in the rear/front of the cell, especially in the conditions where acetylated tubulin is accumulated? An analysis of acetylated tubulin distribution normalized to total tubulin distribution could reveal if there is any bias in the orientation of the former towards the leading edge.
As shown in New Figure EV5G, there is not significant changes in cell shape in all conditions, and thus we do not believe this will be an issue in our analyses. We also quantified total tubulin distribution as suggested by the reviewer, Figures 6C, 7B and 7D, which shows that there is indeed a correlation between the distribution of acetylated-tubulin and total tubulin. This is now discussed in the text, page 12, lines 290-291.
It is also not clear whether the orientation of the microtubules in respect to their minus- to plus-ends is analysed, or whether only the distribution of the acetylation signal throughout the cell is taken into account.
We have assessed the orientation of microtubules, also suggested by reviewer #3, by using EB3-GFP/EB3-RFP expressing RPE-1 cells to track microtubule plus ends. By doing so, we were able to quantify the % of microtubules that have the expected minus end (centre of the cells) to plus end (cell periphery) polarity. As it can be seen in Figure 6E-F, >97% of all EB3-labelled microtubule +ends move towards the cell periphery in all conditions analysed. This is further explained on page 12, lines 300-305.
Another concern in this analysis is that between the normal (-DOX) and increased-acetylation (Tubacin and ATAT1 overexpression) conditions, the amount of acetylated microtubules differs greatly. This may conceal the effects the authors aim to determine, and therefore not allow to draw meaningful conclusions about the polarization of the signal. An additional control the authors may consider to add is overexpression of ATAT1, or Tubacin treatment in cells with amplified centrosomes to check whether this leads to polarization of the highly acetylated microtubule network.

We thank the reviewer for this interesting idea that could directly test our model. We have now performed the suggested experiment where we treated cells with extra centrosomes (+DOX) with Tubacin. We observed that, when compared with Tubacin treated alone, in +DOX +Tubacin treated cells, a higher frequency of highly polarised microtubules (Figure 6D). *[Please note that we acknowledge that the order that these data is described in the text is not ideal but with the limiting number of supplementary figures allowed we believe this is the best use of space and also makes more sense to have it side by side with the previous experiment]*. In addition, Figure 7E clearly shows that centrosome amplification is sufficient to maintain a polarised microtubule network (both acetylated tubulin and α-tubulin) even when the majority of microtubules are acetylated. More importantly, the same levels of centrosome displacement can be observed in +DOX versus +DOX +Tubacin treated cells (Figure 7G,H). These data strongly support our model and indicates that polarisation of microtubules and tubulin acetylation is key to provide the anisotropic force distribution required for centrosome displacement. This is now discussed in the text, page 12, lines 305-312.

7. Does H_2O_2 treatment in cells with normal centrosomes also lead to higher nucleus deformability and ability to migrate through small pores? From the current data it seems to be the condition that most faithfully replicates the effects of overamplified centrosomes by increasing acetylated tubulin in the orientation of the leading edge, as well as by inducing the displacement of vimentin. Showing that H_2O_2 treatment leads to increased deformability of the nucleus would thus strengthen the author's hypothesis that vimentin displacement contributes to this phenotype.
We agree with the reviewer that this was missing from our original manuscript and was also highlighted by reviewer #1. We have now done the following experiments: 1) we quantified nuclear circularity in cells treated with H_2O_2 and found that indeed, H_2O_2 treated cells have nuclear deformability (New Figure 8F). We also found that depletion of αTAT1 from cells with extra centrosomes rescues the nuclear deformability phenotype, further supporting our model (New Figure 8F). In addition, we also assessed nuclear translocation through small (5 μm) and larger (8 μm) pores in these conditions. Consistent with our data on nuclear deformability, we found that H_2O_2 treatment facilitates faster nuclear translocation through small pores but has no effect on migration through larger pores. Furthermore, αTAT1 depletion in cells with amplified centrosomes does not affect migration in larger pores (8 μm) but prevents faster nuclear translocation in smaller pores (5 μm). Thus, tubulin acetylation facilitates nuclear translocation through confined spaces. This is now described in the text, page 13, lines 334-336 and page 14, lines 348-353.

Minor comments:
1. There are a couple of instances where the authors make imprecise statements regarding the existing literature. This should be carefully revised:
- in the abstract: "Microtubule posttranslational modifications (PTMs) contribute to microtubule diversity and differentially regulate motor-mediated transport." - it would be more appropriate to say "can regulate..." as there is only sparse evidence for this so far.
Corrected.

- The authors write at several instances that acetylation is known to affect kinesin-1 transport. They should make sure to specify that this was observed in one few studies in cells, while in vitro, kinesin-1 was not sensitive to tubulin acetylation. In general, how tubulin acetylation affects kinesin-1 mediated transport remains a controversial issue, which should be clearly stated and discussed. (in abstract, introduction and discussion at different instances).
We appreciate this comment and agree with the reviewer that evidence is still sparse, particularly in cells, which is why we believe our data is very exciting and impactful for the field. We have now tried to emphasise this as suggested by the reviewer throughout the manuscript.

It is clear that depletion of αTAT1, and as a consequence the loss of tubulin acetylation, has little or no effect on control untreated cells in terms of intracellular organisation. This is in contrast to loss of KIF5B, which has a significant effect in control cells (decrease in centrosomes-nucleus and endosome-nucleus distance, and decrease in the distribution ratio of vimentin). We believe that this has made assessing the role of tubulin acetylation more difficult and controversial, mainly because tubulin acetylation levels are very low in normal, unperturbed somatic cells. We have now made this clear in the text, page 17, lines 429-433.

- line 76: "...PTMs, have been shown to differentially regulate several molecular motors..." this statement should be toned down, and it should be explained that the experiments were done with chemically modified yeast tubulin.

Changed to: ”(...) PTMs, *have been shown to differentially regulate several molecular motors in vitro using chemically modified yeast tubulin”*

2. In the abstract, the authors propose that "that each organelle must have its own sensing and response mechanism". This is a long shot and even as a hypothesis a bit of an overstatement given the data they present in their paper. They should tone down.

Changed to “*We propose that tubulin acetylation differentially impacts kinesin-1-mediated organelle displacement, suggesting that how each organelle respond to changes in the microtubule network is different.”*

3. Throughout the manuscript, the authors should verify the correct wording of tubulin isotypes (they wrote at several instances "isoforms").

We thank the reviewer for spotting this, now corrected.

4. In all figures, real p-values instead of stars or n.s. should be shown.

We thank the reviewer for highlighting this, as this practice is part of the style guidelines for authors. We have now added the real p-values to every graph for all statistically significant tests, although we have continued to use n.s. where tests are non-significant, as we believe this enhances readability.

5. Page 5, line 121: there appears to be a typo: is it meant "catalytically INactive", not "active"?

The original text is indeed correct. This specific truncation prevents centrosome localisation of PLK4 while maintaining kinase activity. Thus, our results are not a consequence of non-specific kinase activity. We provided more explanation to make this clearer in the text, page 5, lines 127-129.

6. There is missing information about the Centrin-2 antibody in the Methods.

Now added.

7. Legend of Fig. EV1A: the colours of Centrin2 and DNA in the legend are swapped.

Corrected.

8. Fig. 2D: are the images to the same scale? The nuclei in the sip150 condition appear smaller than the siCtrl. Is this a phenotype of sip150 treatment?

This is not a phenotype of p150 depletion, we have now added better representative images to this panel.

9. Fig. 3C and D: was this analysis performed on cells in a 3D matrix? Does it work good for cells that are not so polarized? It would be important to show an example on a real image just below the cartoon.

Analyses were performed in 2D cells and we have clarified this in the methods section, page 29, line 698. These cells are generally polarised but we have now added an image of a real cell next to the scheme. This is now Figure EV3C.

10. Fig. 3I: did the authors assess whether the microtubules that are nocodazole-resistant are also detyrosinated, especially those that are not positive for acetylation staining?
We have not checked this but it is possible that these are long-lived microtubules and thus may harbour other PTMs. This is something we would like to further explore in future work.

11. The ROS analysis by live-cell imaging is incorrectly referred to as Fig. EV3E,F in the text (p.8, line 205); it is EV3C,D in the figure. The lack of explanation in the Results of what is "DCF" used for this imaging also creates confusion about this experiment.
This is now corrected. We have now added an explanation in the text, page 9, lines 212-215: "(…) ROS levels, as measured by the levels of Dichlorodihydrofluorescein (DCF) that results from ROS-mediated oxidation of hydrolysed H_2DCFDA." We have also added this information to the methods, page 28, lines 676-678.

12. Line 215: "These results demonstrate that cells with extra centrosomes have increased acetylated microtubules at least in part due to higher levels of total tubulin." This statement is puzzling - do the authors mean to say that there is no specific effect of acetylation at all? Is it all about more microtubules?
That is not what we meant. In fact, as we hope the reviewer can appreciate, many of the phenotypes we observed regarding intracellular organisation require tubulin acetylation. Depletion of αTAT1 prevents the displacement of centrosomes, vimentin and mitochondria (Figure 4). Importantly, H2O2 treatment does not change total microtubule mass (Figure 3H,I) but leads to similar increase in tubulin acetylation and displacement phenotypes (Figure 5A-D). Furthermore, Tubacin treatment promotes vimentin and mitochondria displacement (Figure 5F,H) but does not change total microtubule mass (Rebuttal Figure 6).

While we do not know what is causing increased tubulin acetylation in cells with extra centrosomes, one logical explanation is that increased microtubule nucleation that occurs in cells with extra centrosomes (Godinho *et al*., 2014; PMID: 24739973) could lead to increase microtubule acetylation. αTAT1 has to access polymerised microtubules and one possibility entry point is via microtubule plus ends, which are concentrated around the centrosomes. Thus, increased nucleation could indeed increase tubulin acetylation. However, this is presently unknown and it will take much more work to determine what is driving this increase, which we believe is out of the scope of this manuscript. We describe this on page 9, lines 227-230.

13. Fig EV4A,B: is there a reason authors do not show images of their siRNA-treated cells? They show images for every other experiment in this manuscript.
We are not sure if we fully understood this comment. As we hope the reviewer can see, we added multiple examples of cells depleted of αTAT1 to Figures 4A,C,D and EV4D,F. Control cells depleted of αTAT1 are completely normal, nothing abnormal that we can see. If the reviewer is asking specifically about quantifying tubulin acetylation levels by IF in αTAT1-depleted cells, the reason why we showed WB data is because it is already very difficult to detect tubulin acetylation in interphase control cells by IF and the goal of this panel is to demonstrate the efficiency of αTAT1 depletion. We have now added to Figure EV4C images of control and cells with amplified centrosomes depleted of αTAT1 where it is very clear that the levels of acetylated tubulin are very low (page 10, lines 239-240).

Also, how do the authors explain that a 50% reduction of ATAT1 expression is sufficient to abolish acetylation? After all, ATAT1 is an enzyme and should still be able to do its function when overall levels are reduced.

Unfortunately, αTAT1 protein levels could not be assessed since the available antibodies do not work for WB/IF, which would have been the ideal experiment. Thus, it is difficult to infer how much protein is left after depletion. However, the levels of tubulin acetylation in control cells are very low suggesting that even when endogenous αTAT1 is present, most microtubules are not acetylated. However, inhibiting the α-tubulin deacetylase HDAC6 with Tubacin is sufficient to promote massive tubulin acetylation even after 4 hrs of treatment (quantification of ac-tubulin levels upon Tubacin treatment can be seen in Figure EV5F). Therefore, we should perhaps consider that, at least at endogenous levels, not all αTAT1 is active or able to acetylate microtubules, or that the balance is shifted towards HDAC6 activity to prevent higher levels of tubulin acetylation. However, our data clearly shows (both by WB and IF images) that depletion of αTAT1 effectively depleted most ac-tubulin in the cells. Thus, we do not think this is an issue.

14. In Fig. 5G, how was mitochondria area determined as a fraction of whole cell area - was segmentation used? Also, the labelling of the shown staining is missing for these images.

Segmentation was not used; this is now explained in the methods, page 26, lines 630-632. Labelling has been added to the figure.

15. Fig. EV5F: the overview of the phenotypes in this table is very useful, but the H2O2 treatment should be included as well.

Good point. This has been included.

16. Page 10, line 251: it seems that author wanted to cite Fig. EV5E, not D - please confirm.

The reviewer is right, this has now been corrected.

17. Fig.7E,F: please describe how the migration was quantified, and what the different phases mean?

Migration was quantified as the time a nucleus spends crossing pores of different sizes, which we tried to explain in the scheme in Figure 7, now New Figure 8G. We have added a description in Figure 8G of what the different phases are, including phase 2, which is when the nucleus crosses the pores. We added this to figure legend and main text, page 14, lines 343-343. We have also added an explanation how migration was quantified in the methods, page 31, lines 738-740, which was missing in our original version.

1st Revision - Editorial Decision 30th May 2023

Dear Susana,

Thank you for submitting a revised version of your manuscript. Your study has now been seen by two of the original referees, who find that most of their previous concerns have been addressed and are now broadly in favour of the acceptance of the manuscript. There remain only a few mainly editorial points that have to be addressed before I can extend formal acceptance of the manuscript:

1. Please address the comments by referee #4 and add a more fine-tuned discussion of the results.
2. Our publisher has done their pre-publication check on your manuscript. I have attached the file here. Please take a look at the word file and the comments regarding the figure legends and respond to the issues. Please also use this version when you resubmit the revised version.
3. Please submit up to five keywords.
4. Please make sure that the order of the sections in the manuscript is as follows: abstract, introduction, results, discussion, materials & methods, data availability section, acknowledgments, disclosure statement and competing interests, references, main figure legends, tables, expanded figure legends
5. There is a reference to "data not shown" on page 18. According to our policy, which does not permit references to "data not shown", please include this information in the Appendix. Please see also https://www.embopress.org/page/journal/14602075/authorguide#unpublisheddata.
6. Figure panel 6D is not mentioned in the manuscript text.
7. Please check the order of figure callouts: Fig 6F should be called out before Fig 7A-B.
8. We require a Data Availability Section at the end of Materials and Methods. As far as I can see, no data deposition in external databases is needed for this paper. If I am correct, then please state in this section: This study includes no data deposited in external repositories. Further information can be found at https://www.embopress.org/page/journal/14602075/authorguide#dataavailability
9. Please remove movie legends from the manuscript text file and zip together with each movie file. Further information is available here: https://www.embopress.org/page/journal/14602075/authorguide#expandedview
10. Please rename "Conflict of interest" section into "Disclosure and competing interests statement" (further info: https://www.embopress.org/page/journal/14602075/authorguide#conflictsofinterest).
11. We generally encourage publication of source data for electrophoretic gels and blots, with the aim of making primary data more accessible and transparent to the reader. We would need one file per figure (which can be a composite of source data from several panels) in jpg, gif or PDF format, uploaded as "Source data files". The gels should be labeled with the appropriate figure/panel number and should have molecular weight markers; further annotation would clearly be useful but is not essential. These files will be published online with the article as supplementary "Source Data". Please let me know if you have any questions about this policy.
12. Papers published in The EMBO Journal are accompanied online by a 'Synopsis' to enhance discoverability of the manuscript. It consists of A) a short (1-2 sentences) summary of the findings and their significance, B) 3-4 bullet points highlighting key results and C) a synopsis image that is 550x300-600 pixels large (width x height, jpeg or png format). You can either show a model or key data in the synopsis image. Please note that the image size is rather small and that text needs to be readable at the final size. Please send us this information together with the revised manuscript.

Please let me know if you have any questions regarding any of these points. You can use the link below to upload the revised files.

Thank you again for giving us the chance to consider your manuscript for The EMBO Journal. I look forward to receiving the final version.

With best wishes,

Ieva

Ieva Gailite, PhD
Senior Scientific Editor
The EMBO Journal
Meyerhofstrasse 1
D-69117 Heidelberg
Tel: +4962218891309
i.gailite@embojournal.org

Further information is available in our Guide For Authors: https://www.embopress.org/page/journal/14602075/authorguide

We realize that it is difficult to revise to a specific deadline. In the interest of protecting the conceptual advance provided by the work, we recommend a revision within 3 months (28th Aug 2023). Please discuss the revision progress ahead of this time with the editor if you require more time to complete the revisions.

Referee #3:

The authors did a great job by addressing most of my comments in the revised manuscript. Altogether it is a well-executed study with a lot of interesting findings. I would recommend it for publishing.

On a side note, for the point #6, I agree with removing new data from the revised manuscript but it would be great to see point-by-point reply letter published next to the manuscript as these findings, even when they are not fully explored, may be valuable for the field.

Referee #4:

The authors have carefully revised the manuscript and addressed most of the points raised in the first round of review. The novel experiments they have performed, together with the re-writing of the manuscript text have helped to substantially clarify the points that were initially raised. Moreover, they have taken great care to give detailed feedback to the referees' comments, which is much appreciated.

Nevertheless, the revised version still requires some additional revision, in particular to avoid overinterpretation of the results. This problem particularly appears when the authors aim at drawing general conclusions, for instance on a general link between tubulin acetylation and organelle transport by kinesin-1. From a critical reading of the literature, as well as from the data provided in this manuscript, it is apparent that those mechanisms are more complex, and still less well understood than what the authors aim to suggests. To give an example from the authors data: while both vimentin and centrosome displacement can be rescued by reduction of acetylation (siATAT1) and depletion of kinesin-1 (siKIF5B), endosomes remained dispersed regardless of the acetylation state, while the dispersal phenotype was rescued by siKIF5B. Similarly, no centrosome displacement is observed upon acute increase of acetylation. This indicates that the behaviour of KIF5B cannot be fully correlated, nor directly linked, to tubulin acetylation, which however was at places suggested in the introduction.

Moreover, how do the authors explain the fact that kinesin-1 is in some cases dependent on a polarized acetylated microtubule network to generate pushing forces (for centrosome, p. 12 lines 298-301), while in other cases simply acetylation, but not microtubule polarization is required to achieve this (vimentin, mitochondria)? The authors should critically revise their text to make sure that they propose a coherent model for their findings, and avoid overinterpretations and generalisations.

Additionally, there are some minor points the authors should address:

1) Fig. EV1E (sip150 blot): is alpha-tubulin the proper choice for a loading control, considering that the total tubulin levels increase in cells with overamplified centrosomes? Could they clarify whether 15 µg total protein were loaded in each lane of this immunoblot?

2) Fig. 2C: p-value is missing above the PLK4 tested pair.

3) Fig. 2D: the cell contour of the siKIF5B-DOX condition is misaligned.

4) Fig. 8C: how is a ratio of 2 achieved here if 1 is a perfect sphere - is this a mistake or is there an explanation for this? Also, are Fig. 8D and E performed in 3D cultures?

Manuscript EMBOJ-2022-112812 – re-revision

"Centrosome amplification fine-tunes tubulin acetylation to differentially control intracellular organization"

Referee #3:

The authors did a great job by addressing most of my comments in the revised manuscript. Altogether it is a well-executed study with a lot of interesting findings. I would recommend it for publishing.

We thank the reviewer for their support! We are very pleased that we could address the main concerns raised.

On a side note, for the point #6, I agree with removing new data from the revised manuscript but it would be great to see point-by-point reply letter published next to the manuscript as these findings, even when they are not fully explored, may be valuable for the field.

We are happy to have the point-by-point rebuttal letter, including any additional data, to be published alongside this manuscript.

Referee #4:

The authors have carefully revised the manuscript and addressed most of the points raised in the first round of review. The novel experiments they have performed, together with the re-writing of the manuscript text have helped to substantially clarify the points that were initially raised. Moreover, they have taken great care to give detailed feedback to the referees' comments, which is much appreciated.

We thank the reviewer for their comments. We are very happy that we could address the main concerns raised.

Nevertheless, the revised version still requires some additional revision, in particular to avoid overinterpretation of the results. This problem particularly appears when the authors aim at drawing general conclusions, for instance on a general link between tubulin acetylation and organelle transport by kinesin-1. From a critical reading of the literature, as well as from the data provided in this manuscript, it is apparent that those mechanisms are more complex, and still less well understood than what the authors aim to suggests.

We try as much as possible to address this point. We have been careful not to overinterpret our data, something we certainly do not want to do. For example, in the first sentence of our Discussion we say "*Here, we demonstrate that centrosome amplification is sufficient to change intracellular organization, a process that requires kinesin-1 mediated transport and is partly regulated by increased tubulin acetylation*". We have tried to clearly highlight a partial role for tubulin acetylation upfront to explicitly avoid implying a direct role. Based on our data, we put together a model, that we are also careful to describe as a model in our Discussion as well.

To give an example from the authors data: while both vimentin and centrosome displacement can be rescued by reduction of acetylation (siATAT1) and depletion of kinesin-1 (siKIF5B), endosomes

remained dispersed regardless of the acetylation state, while the dispersal phenotype was rescued by siKIF5B.

This specific example the reviewer describes above is already discussed in the manuscript: *"By contrast, depletion of αTAT1 had no significant impact on endosomes and Golgi reorganization, suggesting that other microtubule PTMs and/or adaptor proteins, which link organelles to microtubules, could specifically affect the relocation of these organelles in cells with amplified centrosomes. It is also possible that other plus-end kinesin motors, such as kinesin-3, could transport endosomes independently of tubulin acetylation (Bielska et al, 2014; Wedlich-Soldner et al, 2002)"*. Thus, we specifically state that other mechanisms independent of tubulin acetylation exist that drive the displacement of these compartments. To try to make this point clearer, we have now added the following sentence to the same section, on page 16, lines 408-410:" Importantly, while endosome displacement requires kinesin-1 in cells with amplified centrosomes, this was independent of tubulin acetylation, demonstrating that tubulin acetylation is not a general mechanism to regulate organelle transport. This is consistent with the observation that endosomes do not localize to acetylated microtubules (Friedman *et al.*, 2010)."

Similarly, no centrosome displacement is observed upon acute increase of acetylation. This indicates that the behaviour of KIF5B cannot be fully correlated, nor directly linked, to tubulin acetylation, which however was at places suggested in the introduction.

We want to emphasise that we have in the results section the following sentence: *"Tubulin acetylation has been shown to positively influence kinesin-1 transport in cells, although to date the evidence suggesting that acetylated microtubules promote kinesin-1 transport is still scarce"*. No direct link is trying to be established here.

We completely agree with the reviewer, in fact we dedicated an entire figure to try to understand why acute and unpolarized increased of acetylated microtubules is not sufficient to promote centrosome displacement, which was surprising to us but turned out to be a really exciting part of our work! Clearly this has been missed by the reviewer and we tried to clarify this section, as explained below.

Moreover, how do the authors explain the fact that kinesin-1 is in some cases dependent on a polarized acetylated microtubule network to generate pushing forces (for centrosome, p. 12 lines 298-301), while in other cases simply acetylation, but not microtubule polarization is required to achieve this (vimentin, mitochondria)? The authors should critically revise their text to make sure that they propose a coherent model for their findings, and avoid overinterpretations and generalisations.

We are sorry that the reviewer thinks that we are overinterpreting and generalising our findings. That is not our intention at all and we tried very carefully to avoid this.

Regarding the specific point made above, we indeed provide a model to explain the 'discrepancy' outlined above. In fact, we believe that this 'discrepancy' is one of the most exciting points of this work because it allowed us to re-think how PTMs and motors generate pushing forces and that we should not apply the same model to everything (e.g. to all organelles). We tried to describe our idea with a scheme in Fig 6A and in the results section, but clearly that was not sufficient.

We have now re-written this section, which we hope improves clarity: *"We hypothesized that, in addition to increased levels, the distribution or orientation of acetylated microtubules could differentially impact centrosome positioning. As a single, discrete organelle from where microtubules*

emanate, centrosomes are uniquely surrounded by microtubules and thus we postulated that centrosome displacement would likely be dependent on the distribution or polarization of motor-mediated pushing forces. For example, an isotropic distribution of pushing forces around the centrosome would cancel each other, thereby preventing its displacement (Fig 6A). By contrast, if the microtubule network, and specifically acetylated microtubules, became polarized then this could lead to the anisotropic distributions of forces required to displace the centrosome (Fig 6A). This model could also explain why other intracellular components, such as vimentin and mitochondria, which are usually transported along single microtubules (Friedman *et al.*, 2010; Hookway *et al*, 2015)*, would be less sensitive to the distribution of pushing forces".* This has been highlighted in yellow in the main text.

We have now expanded this part in the Discussion for additional clarity, page 16, lines 427-434: *"These observations led us to propose a model whereby, in addition to increased levels, the distribution of forces, through changes in tubulin acetylation, is required to displace centrosomes. Because microtubules emanate from the centrosomes in all directions, we hypothesized that centrosome displacement is likely to be more sensitive to the distribution of acetylated microtubules and motor-mediated pushing forces. While isotropic distribution of forces would cancel each other and block centrosome displacement, an anisotropic distribution of forces, promoted by the polarized distribution of acetylated microtubules, would lead to centrosome displacement. According to this, displacement of intracellular components that move along single or bundled microtubules could be much less sensitive to the distribution or polarization of pushing forces."* This has been highlighted in yellow in the main text.

Additionally, there are some minor points the authors should address:

1) Fig. EV1E (sip150 blot): is alpha-tubulin the proper choice for a loading control, considering that the total tubulin levels increase in cells with overamplified centrosomes? Could they clarify whether 15 µg total protein were loaded in each lane of this immunoblot?

We appreciate this point. However, we have never detected an increase in α-tubulin expression in cells with extra centrosomes. Due to how tightly tubulin expression is regulated we doubt that this is the case. What we have observed is increased polymerized tubulin by IF. Nevertheless, we would like to stress that the point of this specific WB is to show the efficacy of p150 depletion by siRNA, which we clearly demonstrate. Thus, the data would be unchanged by using a different loading control.
As written in the methods, for all WB, 15ug of protein was laded per lane.

2) Fig. 2C: p-value is missing above the PLK4 tested pair.

We thank the reviewer for spotting this. Now added (p=0.0002).

3) Fig. 2D: the cell contour of the siKIF5B-DOX condition is misaligned.

Corrected.

4) Fig. 8C: how is a ratio of 2 achieved here if 1 is a perfect sphere - is this a mistake or is there an explanation for this? Also, are Fig. 8D and E performed in 3D cultures?

Well spotted and thank you for highlighting this. The way we calculate the nucleus aspect ratio is by dividing the nucleus width by the length, as indicated in Fig 8A. However, in order to maintain 1 as

the maximum value (1=perfect circle) we always divided the lowest by the largest value, since nucleus orientation is not important. The analyses of the data in Fig 8D were not performed this way, hence values above 1. We have now re-analysed these experiments and plotted the data to match the other analyses. The result remains unchanged. We also added this description to the methods, which was missing.

2nd Revision - Editorial Decision **14th Jun 2023**

Dear Susana,

Thank you for submitting the final revision of your manuscript. I am now pleased to inform you that your manuscript has been accepted for publication in the EMBO Journal.

Please note that it is EMBO Journal policy for the transcript of the editorial process (containing referee reports and your response letter) to be published as an online supplement to each paper. If you do NOT want this, you will need to inform the Editorial Office via email immediately. More information is available here: https://www.embopress.org/page/journal/14602075/authorguide#transparentprocess

Your manuscript will be processed for publication in the journal by EMBO Press. Manuscripts in the PDF and electronic editions of The EMBO Journal will be copy edited, and you will be provided with page proofs prior to publication. Please note that supplementary information is not included in the proofs.

You will be contacted by Wiley Author Services to complete licensing and payment information. The required 'Page Charges Authorization Form' is available here: https://www.embopress.org/pb-assets/embo-site/tej_apc.pdf - please download and complete the form and return to embopressproduction@wiley.com

EMBO Press participates in many Publish and Read agreements that allow authors to publish Open Access with reduced/no publication charges. Check your eligibility: https://authorservices.wiley.com/author-resources/Journal-Authors/open-access/affiliation-policies-payments/index.html

Should you be planning a Press Release on your article, please get in contact with embojournal@wiley.com as early as possible, in order to coordinate publication and release dates.

If you have any questions, please do not hesitate to call or email the Editorial Office. Thank you for this contribution to The EMBO Journal and congratulations on a successful publication!

Best wishes,

Ieva

Ieva Gailite, PhD
Senior Scientific Editor
The EMBO Journal
Meyerhofstrasse 1
D-69117 Heidelberg
Tel: +4962218891309
i.gailite@embojournal.org

EMBO Press Author Checklist

Corresponding Author Name: Susana A. Godinho	
Journal Submitted to: EMBO JOURNAL	
Manuscript Number:	

USEFUL LINKS FOR COMPLETING THIS FORM
The EMBO Journal - Author Guidelines
EMBO Reports - Author Guidelines
Molecular Systems Biology - Author Guidelines
EMBO Molecular Medicine - Author Guidelines

Reporting Checklist for Life Science Articles (updated January
This checklist is adapted from Materials Design Analysis Reporting (MDAR) Checklist for Authors. MDAR establishes a minimum set of requirements in transparent reporting in the life sciences (see Statement of Task: 10.31222/osf.io/9sm4x). Please follow the journal's guidelines in preparing your
Please note that a copy of this checklist will be published alongside your article.

Abridged guidelines for figures
1. Data
The data shown in figures should satisfy the following conditions:
- the data were obtained and processed according to the field's best practice and are presented to reflect the results of the experiments in an accurate and unbiased manner.
- ideally, figure panels should include only measurements that are directly comparable to each other and obtained with the same assay.
- plots include clearly labeled error bars for independent experiments and sample sizes. Unless justified, error bars should not be shown for technical
- if n<5, the individual data points from each experiment should be plotted. Any statistical test employed should be justified.
- Source Data should be included to report the data underlying figures according to the guidelines set out in the authorship guidelines on Data

2. Captions
Each figure caption should contain the following information, for each panel where they are relevant:
- a specification of the experimental system investigated (eg cell line, species name).
- the assay(s) and method(s) used to carry out the reported observations and measurements.
- an explicit mention of the biological and chemical entity(ies) that are being measured.
- an explicit mention of the biological and chemical entity(ies) that are altered/varied/perturbed in a controlled manner.
- the exact sample size (n) for each experimental group/condition, given as a number, not a range;
- a description of the sample collection allowing the reader to understand whether the samples represent technical or biological replicates (including how many animals, litters, cultures, etc.).
- a statement of how many times the experiment shown was independently replicated in the laboratory.
- definitions of statistical methods and measures:
  - common tests, such as t-test (please specify whether paired vs. unpaired), simple χ2 tests, Wilcoxon and Mann-Whitney tests, can be unambiguously identified by name only, but more complex techniques should be described in the methods section;
  - are tests one-sided or two-sided?
  - are there adjustments for multiple comparisons?
  - exact statistical test results, e.g., P values = x but not P values < x;
  - definition of 'center values' as median or average;
  - definition of error bars as s.d. or s.e.m.

Please complete ALL of the questions below.
Select "Not Applicable" only when the requested information is not relevant for your study.

Materials

Newly Created Materials	Information included in the manuscript?	In which section is the information available? (Reagents and Tools Table, Materials and Methods, Figures, Data Availability Section)
New materials and reagents need to be available; do any restrictions apply?	Not Applicable	

Antibodies	Information included in the manuscript?	In which section is the information available? (Reagents and Tools Table, Materials and Methods, Figures, Data Availability Section)
For **antibodies** provide the following information:<br>- Commercial antibodies: RRID (if possible) or supplier name, catalogue number and or/clone number<br>- Non-commercial: RRID or citation	Yes	Methods section, antibody table

DNA and RNA sequences	Information included in the manuscript?	In which section is the information available? (Reagents and Tools Table, Materials and Methods, Figures, Data Availability Section)
**Short novel DNA or RNA including primers, probes**: provide the sequences.	Yes	Methods section, Primers and siRNA tables

Cell materials	Information included in the manuscript?	In which section is the information available? (Reagents and Tools Table, Materials and Methods, Figures, Data Availability Section)
**Cell lines:** Provide species information, strain. Provide accession number in repository **OR** supplier name, catalog number, clone number, and/**OR** RRID.	Yes	Methods section, cell culture description
**Primary cultures:** Provide species, strain, sex of origin, genetic modification status.	Not Applicable	
Report if the cell lines were recently **authenticated** (e.g., by STR profiling) and tested for mycoplasma contamination.	Yes	Methods section, cell culture description

Experimental animals	Information included in the manuscript?	In which section is the information available? (Reagents and Tools Table, Materials and Methods, Figures, Data Availability Section)
**Laboratory animals or Model organisms:** Provide species, strain, sex, age, genetic modification status. Provide accession number in repository **OR** supplier name, catalog number, clone number, **OR** RRID.	Not Applicable	
**Animal observed in or captured from the field:** Provide species, sex, and age where possible.	Not Applicable	
Please detail **housing and husbandry conditions**.	Not Applicable	

Plants and microbes	Information included in the manuscript?	In which section is the information available? (Reagents and Tools Table, Materials and Methods, Figures, Data Availability Section)
**Plants:** provide species and strain, ecotype and cultivar where relevant, unique accession number if available, and source (including location for collected wild specimens).	Not Applicable	
**Microbes:** provide species and strain, unique accession number if available, and source.	Yes	Methods section, Plasmids description

Human research participants	Information included in the manuscript?	In which section is the information available? (Reagents and Tools Table, Materials and Methods, Figures, Data Availability Section)
If collected and within the bounds of privacy constraints report on age, sex and gender or ethnicity for all study participants.	Not Applicable	

Core facilities	Information included in the manuscript?	In which section is the information available? (Reagents and Tools Table, Materials and Methods, Figures, Data Availability Section)
If your work benefited from core facilities, was their service mentioned in the acknowledgments section?	Yes	Acknowledgements section

Design

Study protocol	Information included in the manuscript?	In which section is the information available? (Reagents and Tools Table, Materials and Methods, Figures, Data Availability Section)

If study protocol has been **pre-registered, provide DOI in the manuscript**. For clinical trials, provide the trial registration number **OR** cite DOI.	Not Applicable	
Report the **clinical trial registration number** (at ClinicalTrials.gov or equivalent), where applicable.	Not Applicable	

Laboratory protocol	Information included in the manuscript?	In which section is the information available? (Reagents and Tools Table, Materials and Methods, Figures, Data Availability Section)
Provide DOI OR other citation details if **external detailed step-by-step protocols** are available.	Yes	References for specific protocols are added to the methods section

Experimental study design and statistics	Information included in the manuscript?	In which section is the information available? (Reagents and Tools Table, Materials and Methods, Figures, Data Availability Section)
Include a statement about **sample size** estimate even if no statistical methods were used.	Yes	Sample size is added to all figure legends
Were any steps taken to minimize the effects of subjective bias when allocating animals/samples to treatment (e.g. **randomization procedure**)? If yes, have they been described?	Not Applicable	
Include a statement about **blinding** even if no blinding was done.	Yes	
Describe **inclusion/exclusion criteria** if samples or animals were excluded from the analysis. Were the criteria pre-established?<br><br>If sample or data points were omitted from analysis, report if this was due to attrition or intentional exclusion and provide justification.	Yes	Methods section, Quantification of indirect immunofluorescence images
For every figure, are **statistical tests** justified as appropriate? Do the data meet the assumptions of the tests (e.g., normal distribution)? Describe any methods used to assess it. Is there an estimate of variation within each group of data? Is the variance similar between the groups that are being statistically compared?	Yes	All statiscal methods are added to figure legends and desccribed in the methods.

Sample definition and in-laboratory replication	Information included in the manuscript?	In which section is the information available? (Reagents and Tools Table, Materials and Methods, Figures, Data Availability Section)
In the figure legends: state number of times the experiment was **replicated** in laboratory.	Yes	Figure legends
In the figure legends: define whether data describe **technical or biological replicates**.	Yes	Figure legends

Ethics

Ethics	Information included in the manuscript?	In which section is the information available? (Reagents and Tools Table, Materials and Methods, Figures, Data Availability Section)
Studies involving **human participants**: State details of **authority granting ethics approval** (IRB or equivalent committee(s), provide reference number for approval.	Not Applicable	
Studies involving **human participants**: Include a statement confirming that **informed consent** was obtained from all subjects and that the experiments conformed to the principles set out in the WMA Declaration of Helsinki and the Department of Health and Human Services Belmont Report.	Not Applicable	
Studies involving **human participants:** For publication of **patient photos**, include a statement confirming that consent to publish was obtained.	Not Applicable	
Studies involving experimental **animals**: State details of **authority granting ethics approval** (IRB or equivalent committee(s), provide reference number for approval. Include a statement of compliance with ethical regulations.	Not Applicable	
Studies involving **specimen and field samples:** State if relevant **permits** obtained, provide details of authority approving study; if none were required, explain why.	Not Applicable	

Dual Use Research of Concern (DURC)	Information included in the manuscript?	In which section is the information available? (Reagents and Tools Table, Materials and Methods, Figures, Data Availability Section)
Could your study fall under dual use research restrictions? Please check biosecurity documents and list of **select agents and toxins** (CDC): https://www.selectagents.gov/sat/list.htm	Not Applicable	
If you used a select agent, is the security level of the lab appropriate and reported in the manuscript?	Not Applicable	
If a study is subject to dual use research of concern regulations, is the name of the **authority granting approval and reference number** for the regulatory approval provided in the manuscript?	Not Applicable	

Reporting

The MDAR framework recommends adoption of discipline-specific guidelines, established and endorsed through community initiatives. Journals have their own policy about requiring specific guidelines and recommendations to complement MDAR.

Adherence to community standards	Information included in the manuscript?	In which section is the information available? (Reagents and Tools Table, Materials and Methods, Figures, Data Availability Section)
State if relevant guidelines or checklists (e.g., **ICMJE, MIBBI, ARRIVE, PRISMA**) have been followed or provided.	Not Applicable	
For **tumor marker prognostic studies,** we recommend that you follow the **REMARK** reporting guidelines (see link list at top right). See author guidelines, under 'Reporting Guidelines'. Please confirm you have followed these guidelines.	Not Applicable	
For **phase II and III randomized controlled trials**, please refer to the **CONSORT** flow diagram (see link list at top right) and submit the CONSORT checklist (see link list at top right) with your submission. See author guidelines, under 'Reporting Guidelines'. Please confirm you have submitted this list.	Not Applicable	

Data Availability

Data availability	Information included in the manuscript?	In which section is the information available? (Reagents and Tools Table, Materials and Methods, Figures, Data Availability Section)
Have **primary datasets** been deposited according to the journal's guidelines (see 'Data Deposition' section) and the respective accession numbers provided in the Data Availability Section?	Not Applicable	
Were **human clinical and genomic datasets** deposited in a public access-controlled repository in accordance to ethical obligations to the patients and to the applicable consent agreement?	Not Applicable	
Are **computational models** that are central and integral to a study available without restrictions in a machine-readable form? Were the relevant accession numbers or links provided?	Not Applicable	
If publicly available data were reused, provide the respective **data citations in the reference list**.	Not Applicable	
